# Decay of the Weyl curvature in expanding black hole cosmologies

**DOI:** 10.1007/s40818-022-00125-6

**Published:** 2022-05-04

**Authors:** Volker Schlue

**Affiliations:** School of Mathematics & Statistics, The University of Melbourne, Parkville, VIC 3010 Australia

## Abstract

This paper is motivated by the non-linear stability problem for the expanding region of Kerr de Sitter cosmologies in the context of Einstein’s equations with positive cosmological constant. We show that under dynamically realistic assumptions the conformal Weyl curvature of the spacetime decays towards future null infinity. More precisely we establish decay estimates for Weyl fields which are (i) uniform (with respect to a global time function) (ii) optimal (with respect to the rate) and (iii) consistent with a global existence proof (in terms of regularity). The proof relies on a geometric positivity property of compatible currents which is a manifestation of the global redshift effect capturing the expansion of the spacetime.

## Introduction

In this paper we are interested in solutions to Einstein’s field equations with a positive cosmological constant $\Lambda >0$,

1.1 $$\begin{aligned} {{\,\mathrm{Ric}\,}}(g)=\Lambda g\,. \end{aligned}$$

These equations — in fact a more general form including sources of matter — were proposed by Einstein to model the universe in the large [18], $({\mathcal {M}},g)$ being an unknown $3+1$ dimensional Lorentzian manifold which represents the geometry of space-time.^1^ The simplest solutions to (1.1) have non-trivial topology, and are not asymptotically flat: The Einstein universe (which contains a homogeneous fluid) is topologically a cylinder ${\mathbb {S}}^3\times {\mathbb {R}}$, and thus represents a *closed* universe. While Einstein’s solution is *static* (in time), de Sitter found a solution to the vacuum equations (1.1) shortly after the cosmological constant was introduced [15], which is *expanding*:^2^ The de Sitter space-time can be embedded as a (time-like) hyperboloid *H* in 5-dimensional Minkowski space $({\mathbb {R}}^{4+1},m)$, with metric *h* simply induced by the ambient metric *m*. Its geometric properties are relevant to this paper,^3^ and are discussed in detail in [39].

**Fig. 1:**
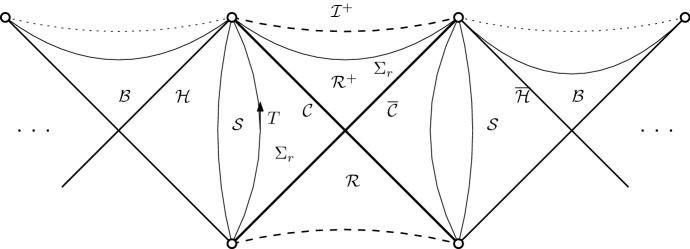
Penrose diagram of Schwarzschild de Sitter geometry

A model of a *black hole* in an expanding universe is provided by the Schwarzschild de Sitter geometry [29, 43] discussed in Section 3. It is a solution to (1.1) with less symmetries than the de Sitter solution but still spherically symmetric, which means that the metric takes the form:

1.2 $$\begin{aligned} g={\mathop {g}\limits ^{{\scriptscriptstyle Q}}}+r^2{\mathop {\gamma }\limits ^{\circ }}\end{aligned}$$

where ${\mathop {g}\limits ^{{\scriptscriptstyle Q}}}$ is a Lorentzian metric on a $1+1$-dimensional manifold *Q*, $r:Q\rightarrow (0,\infty ), q\mapsto r(q)$ is the radius of a sphere $q\in Q$, and ${\mathop {\gamma }\limits ^{\circ }}$ the standard metric on ${\mathbb {S}}^2$. Its causal geometry is best understood if we depict the level sets of *r* in *Q* while keeping the null lines of ${\mathop {g}\limits ^{{\scriptscriptstyle Q}}}$ at $45^\circ$, namely in the form of the Penrose diagram of Fig. 1.

**Fig. 2:**
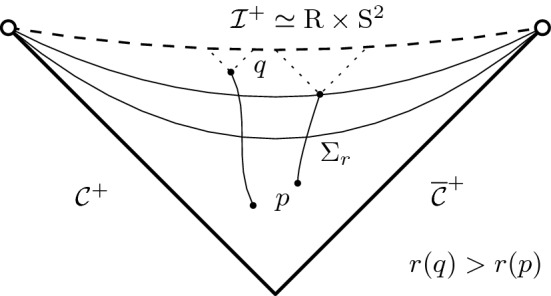
Expanding region of a Schwarzschild de Sitter cosmology

On Schwarzschild de Sitter spacetime we distinguish between the black hole region ${\mathcal {B}}$, the stationary black hole exterior ${\mathcal {S}}$, and the cosmological region ${\mathcal {R}}$. The stationary region ${\mathcal {S}}$ has a time-like Killing vectorfield *T*, and is bounded by an event horizon ${\mathcal {H}}$ towards the interior at $r={r_{{\mathcal {H}}}}$, and a *cosmological horizon* ${\mathcal {C}}$ towards the exterior at $r={r_{{\mathcal {C}}}}$. Beyond ${\mathcal {C}}$ lies the cosmological region, whose future component ${\mathcal {R}}^+$ we depict separately in Fig. 2. ${\mathcal {R}}^+$ is bounded to the past by the null hypersurfaces ${\mathcal {C}}^+\cup \bar{{\mathcal {C}}}^+$, and foliated by the level sets $\Sigma _r$ of *r*:

1.3 $$\begin{aligned} {\mathcal {R}}=\bigcup _{r> {r_{{\mathcal {C}}}}}\Sigma _r \end{aligned}$$

Each leaf $\Sigma _r$ is topologically a cylinder ${\mathbb {S}}^2\times {\mathbb {R}}$, and a *spacelike* hypersurface for $r> {r_{{\mathcal {C}}}}$. Since *r* is increasing along any future-directed causal curve in ${\mathcal {R}}^+$ we also call this region *expanding*. It is future geodesically complete, yet has the property that any two observers are eventually causally disconnected.^4^ This has the consequence that the “ideal boundary at infinity” ${\mathcal {I}}^+$ is a *spacelike* surface.^5^ (${\mathcal {I}}^+$ is not part of the spacetime, but it is intrinsically a cylinder ${\mathbb {R}}\times {\mathbb {S}}^2$ and can be thought of as attached to the spacetime in the topology of the Penrose diagram.) Finally ${\mathcal {B}}$ is referred to as the black hole region, because it lies in the complement of the past of ${\mathcal {I}}^+$.

The maximal extension of the Schwarzschild de Sitter spacetime consists of an infinite chain of black hole regions ${\mathcal {B}}$, separated by exteriors ${\mathcal {S}}$ to the future and past of which lie the cosmological regions ${\mathcal {R}}$. We shall restrict attention to a given cosmological region, and its adjacent black hole exteriors, up to the event horizons; in particular the interior of the black hole is not considered here.^6^

**Fig. 3:**
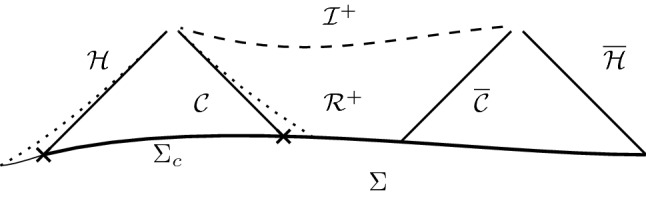
Cauchy problem in the domain of dependence of $\Sigma$

While all classical solutions to (1.1) referred to here were found explicitly, a natural question to ask from the evolutionary point of view is the following:

*Is the picture of Fig.* 3*dynamically stable?*

*In other words, does a perturbation of Schwarzschild de Sitter data on a Cauchy hypersurface* $\Sigma$ *give rise to a maximal development* ${\mathcal {D}}$ *with similar features? In particular does* ${\mathcal {D}}$ *contain a* *future geodesically complete* *region* ${\mathcal {R}}$ *with* *spacelike* *boundary* ${\mathcal {I}}^+$ *at infinity, relative to which* ${\mathcal {D}}$ *contains a black hole region* ${\mathcal {B}}$. *Moreover, is the black hole exterior* ${\mathcal {S}}=I^-({\mathcal {C}})\cap I^-({\mathcal {H}})$ — *where* ${\mathcal {C}}$ *and* ${\mathcal {H}}$ *are defined to be the future boundary of the past of* ${\mathcal {B}}$ *and* $\mathcal {I^+}$, *respectively — asymptotically* *stationary*?

In view of the domain of dependence property of solutions to (1.1) the *stability of the black hole exterior* ${\mathcal {S}}$ can be treated *independently* of the cosmological region ${\mathcal {R}}$ (and the black hole interior ${\mathcal {B}}$). Indeed, since ${\mathcal {S}}$ is contained in the domain of dependence of a compact subset $\Sigma _c\subset \Sigma$, the behavior of the solution in ${\mathcal {S}}$ is not influenced by data in the complement of $\Sigma _c$. In a remarkable series of papers [24–27, 42] Hintz and Vasy have recently proven that solutions to the Cauchy problem for (1.1) arising from a perturbation of Schwarzschild de Sitter data on $\Sigma _c$ *converge exponentially fast to a member of the Kerr de Sitter family* on ${\mathcal {S}}\cup {\mathcal {H}}\cup {\mathcal {C}}$, (and in particular become stationary).^7^

The Kerr de Sitter geometry — given by an explicit 2-parameter family of *axi-symmetric* solutions to (1.1), containing Schwarzschild de Sitter as a subfamily — plays a central role for the understanding of solutions in ${\mathcal {S}}$. Indeed, the result of Hintz and Vasy shows that they parametrize all possible final states for the evolution of perturbations of Schwarzschild de Sitter data, *in the domain bounded by the event horizon* ${\mathcal {H}}$ *and the cosmological horizon* ${\mathcal {C}}$. We will see that for the evolution beyond the cosmological horizon this explicit family of solutions does *not* play an equally prominent role.

**Fig. 4:**
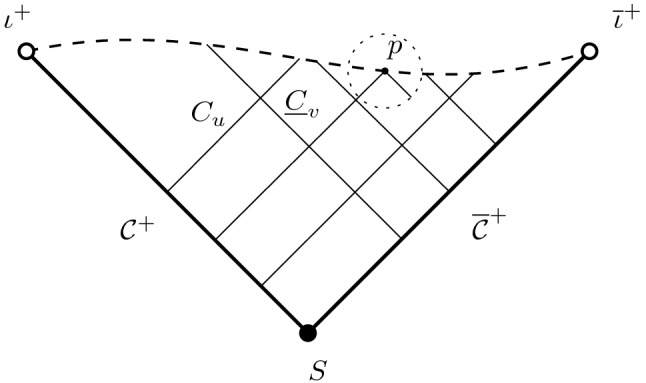
Goursat problem from the cosmological horizons

The problem that motivates this paper is then the following:

*Consider the characteristic initial value problem (or* *Goursat* *problem) for* (1.1) *with data on the (future geodesically complete) cosmological horizons* ${\mathcal {C}}\cup \bar{{\mathcal {C}}}$, *cf. Fig.* 4. *Suppose the characteristic data converges exponentially fast to the geometry induced by a Kerr de Sitter horizon, then is the maximal development* *future geodesically complete*, *and can future null infinity* ${\mathcal {I}}^+$ *be attached at infinity as a* *spacelike* *surface in a suitably regular manner?*

In *spherical symmetry* the analogue of this problem has been addressed in [11] in the context of the Einstein-Maxwell-Scalar field system with $\Lambda >0$. In this paper we approach the above problem *without any symmetries*, but we restrict ourselves to the *linear* analysis of the Bianchi equations, and do not yet pose initial data on the cosmological horizons, but instead on a *spacelike* hypersurface (arbitrarily close to the cosmological horizons) in the cosmological region.

Note that the assumption — that the data be exponentially decaying to a Kerr de Sitter geometry along the cosmological horizons — is justified by virtue of the result of Hintz and Vasy [27].^8^ However, also note that in the discussion of our expectation for the asymptotics no reference is made to the Kerr de Sitter solution.

In [38] I have considered a linear model problem, namely the corresponding Cauchy problem for the linear wave equation

1.4 $$\begin{aligned} \Box _g\psi =0 \end{aligned}$$

on a fixed Kerr de Sitter background $({\mathcal {M}},g)$. It was shown that any solution to (1.4) arising from finite energy data on $\Sigma$, remains globally bounded yet has a limit $\psi ^+$ on ${\mathcal {I}}^+$ which as a function on the standard cylinder ${\mathbb {R}}\times {\mathbb {S}}^2$ has finite energy. Moreover, if exponential decay is assumed along the cosmological horizons (which in this setting is justified by the results of Dyatlov [16, 17]), then this “rescaled” energy of $\psi ^+$ on ${\mathcal {I}}^+$ decays towards time-like infinity $\iota ^+$, but still need not vanish globally on ${\mathcal {I}}^+$. This means that even in the context of the linear theory, *there is a non-trivial degree of freedom at infinity*. Finally, the results in [38] depend by no means on the symmetries of Kerr de Sitter geometry, and have been proven therein for a large class of spacetimes without any symmetries near the Schwarzschild de Sitter cosmology.

The intuition gained in the linear problem tells us that *in the context of the fully non-linear problem we cannot expect convergence to a member of the Kerr de Sitter family, but merely a “nearby” geometry, which is however a priori unknown.* In fact, the setting to be presented in this paper is consistent with the asymptotic geometry to differ from Kerr de Sitter — indeed *de Sitter* spacetime — even at the “leading order”.^9^

This view is echoed in an instructive series of papers by Ashtekar, Bonga and Kesavon [1–3]. In [1] it is argued that future null infinity ${\mathcal {I}}^+$ cannot be conformally flat in the presence of gravitational waves.^10^ They show in particular that the condition that ${\mathcal {I}}^+$ be intrinsically conformally flat (as it is for Schwarzschild de Sitter geometry) would suppress “half” of the gravitational degrees of freedom. This is consistent with the setting in this paper, where will will allow the spheres foliating ${\mathcal {I}}^+$ to be *not perfectly round*.^11^

Now we will treat Einstein’s equations not as a system of wave equations for the metric, but rather using the *electromagnetic analogy*, which has been employed so successfully in the seminal work of Christodoulou and Klainerman [10]. In other words, we use that (1.1) imply the homogeneous contracted Bianchi equations for the Riemann curvature tensor *R*:

1.5 $$\begin{aligned} {{\,\mathrm{div}\,}}R = {{\,\mathrm{curl}\,}}{{\,\mathrm{Ric}\,}}=0 \end{aligned}$$

The Riemann curvature however — in the role of the Faraday tensor *F* — is not a suitable quantity to consider in this setting, and cannot be expected to decay; indeed the de Sitter solution is a *constant curvature space*. In this work we pass from the Riemannian curvature *R* to the *conformal Weyl curvature* *W*, which for any solution to (1.1) is related to *R* by^12^

1.6 $$\begin{aligned} W(X,Y,U,V)=R(X,Y,U,V)+\frac{\Lambda }{3}\Bigl [g(X,V) g(Y,U)-g(X,U)g(Y,V)\Bigr ]\nonumber \\ \end{aligned}$$

and thus also satisfies

1.7 $$\begin{aligned} {{\,\mathrm{div}\,}}W = 0\,. \end{aligned}$$

The conformal Weyl curvature is *the* prototypical Weyl field *W* in the sense of [10] and its algebraic properties allow us to construct energies using the *Bel-Robinson tensor* *Q*(*W*), which can be viewed as a generalisation of the energy-momentum tensor of electromagnetic theory. A key advantage of this approach is then that certain methods developed for the treatment of the linear equation (1.4) — in particular our understanding of the decay mechanism for solution to (1.4) in the cosmological region — carry over to the study of solutions to (1.7). We will elaborate on this in more detail in Section 1.4.

In this paper we will treat the first part of the global non-linear stability problem, as formulated above as a characteristic initial value problem for the cosmological region. Namely following the strategy laid out in [10], we will make certain assumptions on the metric *g*, and the connection coefficients,^13^ and then prove a non-trivial statement for the Weyl curvature. Independently in a second part we will prove that *assuming the bounds on the Weyl curvature* all assumptions that were made here on the metric and the connection coefficients can be derived by a suitable gauge choice on the cosmological horizons.^14^ In the context of an overarching bootstrap argument, it remains to show that the argument closes under the assumption of *small initial data* on the cosmological horizons^15^ to yield a full existence result.

A significant challenge of this part lies in *identifying* a set of assumptions which are on one hand sufficiently general to encompass the actual dynamics of the metric under the evolution of (1.1) (too restrictive assumptions would be inconsistent, and have no chance of being recovered), while on the other hand sufficiently restrictive for the decay mechanism to come into play. The latter is predominantly the *expansion*, which can be captured adequately on the level of *mean curvatures*.

Informally speaking, we establish the following:

*The conformal Weyl curvature decays uniformly in the cosmological region* ${\mathcal {R}}$, *provided the metric and connection coefficients satisfy a set of assumptions which capture in particular the expansion of the spacetime.*

In Schwarzschild de Sitter spacetime the Weyl curvature has only one non-vanishing component

1.8 $$\begin{aligned} \rho [W] =-\frac{2m}{r^3} \end{aligned}$$

where *m* is a constant, the mass of the black hole; cf. Section 2, 3.

“Decay”, and its “uniformity” refer to a parameter like *r* in the Schwarzschild de Sitter example, but as we shall see even the definition of a suitable time function

$$\begin{aligned}r:{\mathcal {R}}\rightarrow (0,\infty )\end{aligned}$$

is non-trivial. The reason the definition of *r* is a non-trivial question is that we subject it to the following two requirements:

- The function *r* is a time-function on ${\mathcal {R}}$ — i.e. it is strictly increasing along any future-directed time-like curve — and the zero level set of 1/*r* can be identified with ${\mathcal {I}}^+$.
- The function *r* is defined by a purely geometric construction, and fully determined by gauge choices on ${\mathcal {C}}\cup \overline{{\mathcal {C}}}$.

In [39] it is shown in particular that — in the context of using optical functions as the purely geometric means by which *r* is defined — the first requirement is not stable at all under perturbations of the gauge choices on ${\mathcal {C}}\cup \overline{{\mathcal {C}}}$; see Section 1.1 for a qualitative discussion. (The paper [39] then also identifies a useful criterion for both requirements to be satisfied, and gives a construction of non-trivial optical functions, and hence time functions in a simplified setting.) In this paper we essentially assume that both requirements are fulfilled, and we will then establish that under our assumptions *all* components of the Weyl curvature decay at precisely the rate indicated in (1.8).

In Section 1.1 we discuss the basic difficulties related to a suitable choice of coordinates which covers the domain of development, and correctly parametrizes future null infinity.^16^ In Section 1.2 we highlight some of the assumptions made in this paper, in particular we identify a suitable notion of expansion at the level of mean curvatures. Then we proceed to a more precise statement of the result in Section 1.3, and discuss some of the ideas and difficulties of the proof in Section 1.4. Finally, we discuss the relation of this result to earlier work, in particular Friedrich’s proof of the stability of the de Sitter solution, in Section 1.6.

### Basic Difficulties

In view of the expectation that the geometry of a dynamical solution to (1.1) in the cosmological region *does not globally converge to a member of the Kerr de Sitter family, but merely to a “nearby geometry” which is a priori unknown*, the choice of suitable coordinate system — which covers the entire region of existence — is non-trivial.

Consider for example any given coordinate system $(x^a;y^A)$ on ${\mathcal {R}}\subset {\mathcal {Q}}$ for the Schwarzschild de Sitter solution. A naïve approach would be to formulate the assumptions on the metric in this coordinate system, and try to establish the decay with respect to a parameter $r=r(x^a)$ formally defined as in the Schwarzschild de Sitter geometry. However, such an approach turns out to be *inconsistent*, the reason being that the surface $r=\infty$ thus defined does not coincide with the true future boundary found in evolution.

To overcome this problem we work in a *double null gauge*,^17^ namely coordinates $(u,v;\vartheta ^1,\vartheta ^1)$ such that the level sets of *u*, *v* are null hypersurfaces whose intersections $S_{u,v}$ are diffeomorphic to ${\mathbb {S}}^2$. This choice is natural for the treatment of a characteristic initial value problem, and it allows us in particular to introduce the function *r* as the *area radius* of the spheres of intersection $(S_{u,v},g\!\!\!/)$,

1.9 $$\begin{aligned} 4\pi r^2(u,v)=\int _{S_{u,v}}\mathrm {d}\mu _{g\!\!\!/}\,. \end{aligned}$$

It is then tempting to define future null infinity ${\mathcal {I}}^+$ as the collection of spheres with infinite area radius. However, a general double null foliation still allows considerable freedom in the choice of the spheres of intersection, and it can happen that a sphere with infinite area radius is in fact partly contained in the spacetime. In such a scenario future null infinity is not correctly identified by the set of points where $r=\infty$.

This subtle yet imporant point is proven in Section 4 of [39], where we give an explicit construction of a double null foliation of de Sitter space which illustrates this phenomenon:

*There exist double null foliations of de Sitter space such that the union of all spheres with area radius* $r\in ({r_{{\mathcal {C}}}},\infty )$ *is contained, but does not exhaust the cosmological region.*

**Fig. 5:**
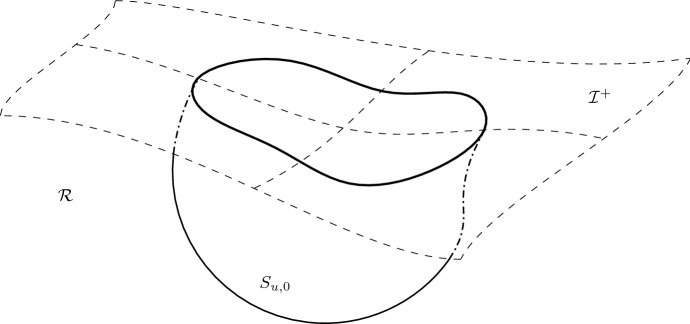
Sphere with infinite area radius partially contained in the spacetime

In fact, these examples are constructed as explicit solutions to the eikonal equation on de Sitter spacetime (*H*, *h*),

1.10 $$\begin{aligned} h(\nabla u,\nabla u)=0 \end{aligned}$$

with the property that the level sets $C_u$ intersect the cosmological horizon ${\mathcal {C}}$ in a small ellipsoidal deformation $C_u\cap {\mathcal {C}}$ of the round sphere, yet the “corresponding sphere near infinity” — namely the intersection $S_{u,0}=C_u\cap {\underline{C}}_0$ with a fixed “incoming” null hypersurface ${\underline{C}}_0$ — is only partially contained in *H*. In these examples, $S_{u,0}$ first “touches infinity at a point” and then gradually “disappears on annular regions” as *u* varies, see Fig. 5.

We expect this to be the generic behavior, and it is important to note that our assumptions on the foliations to be discussed in Section 1.2 rule out such behavior of the spheres near infinity.^18^ In fact, the assumptions of Section 1.2 ensure that the spheres of the foliation indeed exhaust the expanding region; see [39].

Furthermore, in [39] we show — in the case of a fixed de Sitter spacetime with identically vanishing Weyl curvature — that *using a final gauge choice* a global double null foliation *can be constructed*, which has all the properties assumed in Section 1.2. This is achieved by a global analysis of the *null structure equations* on de Sitter space, which arise in the decomposition of (1.1) in double null coordinates $(u,v;\vartheta ^A)$, where *u*, and *v* satisfy (1.10); see Section 5 in [39].

### Assumptions on the foliation

Consider a $3+1$-dimensional Lorentzian manifold $({\mathcal {M}},g)$ with past boundary ${\mathcal {C}}\cup \overline{{\mathcal {C}}}$, two null hypersurfaces ${\mathcal {C}}$ and $\overline{{\mathcal {C}}}$ intersecting in a sphere *S*, whose null geodesic generators have no future end points. We think of initial data prescribed along ${\mathcal {C}}$ and $\overline{{\mathcal {C}}}$, and of ${\mathcal {R}}=J^+({\mathcal {C}}\cup \overline{{\mathcal {C}}})$ — the “cosmological region” — as its future development.

Consider further a double null foliation of ${\mathcal {R}}$ by null hypersurfaces $C_u$, and ${\underline{C}}_v$, namely the level sets of functions

1.11 $$\begin{aligned} u:{\mathcal {R}}\longrightarrow (0,\infty )\qquad v:{\mathcal {R}}\longrightarrow (0,\infty ) \end{aligned}$$

satisfying the eikonal equations

1.12 $$\begin{aligned} g(\nabla u,\nabla u)=0\qquad g(\nabla v,\nabla v)=0 \end{aligned}$$

which are increasing towards the future, such that ${\mathcal {C}}={\underline{C}}_0$, $\overline{{\mathcal {C}}}=C_0$, and

1.13 $$\begin{aligned} \Bigl (S_{u,v}=C_u\cap {\underline{C}}_v ,g\!\!\!/= g|_{\mathrm {T}S_{u,v}}\Bigr ) \end{aligned}$$

is diffeomorphic to ${\mathbb {S}}^2$. Following the conventions in [8] we define

1.14 $$\begin{aligned} L^\prime =-2\mathrm {d}u^\sharp \qquad {\underline{L}}^\prime =-2\mathrm {d}v^\sharp \end{aligned}$$

to be the null geodesic normals, and $\Omega$ to be the null lapse:

1.15 $$\begin{aligned} \Omega =\sqrt{\frac{2}{-g({\underline{L}}^\prime ,L^\prime )}} \end{aligned}$$

#### Remark 1.1

The reader may find it useful to refer in parallel to Section 3 where all of the following geometric quantities are computed for the Schwarzschild de Sitter spacetime in spherically symmetric double null foliations.

Then normalised null normals are given by

1.16 $$\begin{aligned} \hat{{\underline{L}}}=\Omega {\underline{L}}^\prime \qquad \hat{{L}}=\Omega L^\prime \end{aligned}$$

and used to define the null second fundamental forms of the spheres $S_{u,v}$ as surfaces embedded in $C_u$, and ${\underline{C}}_v$ respectively:

1.17 $$\begin{aligned} \chi (X,Y)=g(\nabla _X\hat{{L}}, Y)\qquad {\underline{\chi }}(X,Y)=g(\nabla _X\hat{{\underline{L}}}, Y)\qquad (X,Y\in \mathrm {T}S_{u,v}) \end{aligned}$$

The null expansions, namely the traces ${{\,\mathrm{tr}\,}}{\underline{\chi }}$, and ${{\,\mathrm{tr}\,}}\chi$ (with respect to $g\!\!\!/$), measure pointwise the change of the area element $\mathrm {d}\mu _{g\!\!\!/}$, in the null directions $\hat{{\underline{L}}}$, and $\hat{{L}}$, respectively. Our first main assumption is that “the cosmological region is expanding”: 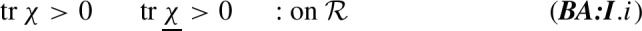 The positivity of the null expansions alone is not enough, and we will assume that they are always close to the “geodesic accelerations” $2{\hat{{\underline{\omega }}}}$, and $2{\hat{\omega }}$ defined by

1.18 $$\begin{aligned} \nabla _{\hat{{L}}}\hat{{L}}={\hat{\omega }}\hat{{L}}\qquad \nabla _{\hat{{\underline{L}}}}\hat{{\underline{L}}}={\hat{{\underline{\omega }}}}\hat{{\underline{L}}}\end{aligned}$$

Equivalently, they are given by

1.19 $$\begin{aligned} {\hat{\omega }}=\hat{{L}}\log \Omega \qquad {\hat{{\underline{\omega }}}}=\hat{{\underline{L}}}\log \Omega \end{aligned}$$

and our second assumption is that for some constant $C_0>0$: 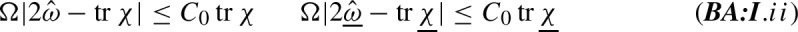


Our third assumption is crucially related to the discussion in Section 1.1, and amounts to the condition that the null expansions are pointwise close to their spherical averages

1.20 $$\begin{aligned} \overline{\Omega {{\,\mathrm{tr}\,}}\chi }=\frac{1}{4\pi r^2}\int _{S_{u,v}}\Omega {{\,\mathrm{tr}\,}}\chi \mathrm {d}\mu _{g\!\!\!/} \qquad \overline{\Omega {{\,\mathrm{tr}\,}}{\underline{\chi }}}=\frac{1}{4\pi r^2}\int _{S_{u,v}}\Omega {{\,\mathrm{tr}\,}}{\underline{\chi }}\mathrm {d}\mu _{g\!\!\!/}\,. \end{aligned}$$

We require that for some constant $C_0>0$
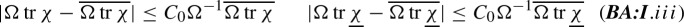


Finally we assume for the remaing connection coefficients, namely the trace-free parts of the null second fundamental forms above, and the torsion

1.21 $$\begin{aligned} \zeta (X)=\frac{1}{2}g(\nabla _X\hat{{L}},\hat{{\underline{L}}})\qquad (X,Y\in \mathrm {T}S_{u,v}) \end{aligned}$$

that 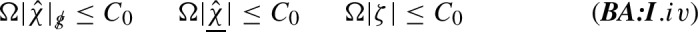


The assumptions (***BA:I***) are $\mathrm {L}^\infty$-bounds on $S_{u,v}$. They already allow us to prove that the $\mathrm {L}^2(\Sigma _r)$- norm of the Weyl curvature decays; see (1.23) for the definition of the spacelike hypersurface $\Sigma _r$, and Section 4.3 for proof of the decay of the Weyl curvature flux through $\Sigma _r$. However, here we seek to prove decay of the Weyl curvature in $\mathrm {L}^4(S_{u,v})$. This requires us to make additional assumptions on the *derivatives of the connection coefficients*. These assumptions, schematically bounds on 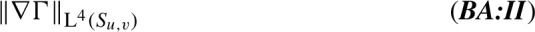 are too numerous and technical to state conveniently here and will instead be collected under the label (***BA:II***) below. They will allow us to prove that all tangential derivatives of *W* to $\Sigma _r$ decay in $\mathrm {L}^2(\Sigma _r)$.

Finally, several assumptions will be needed to deduce the decay rates of the energy and for the application of the elliptic theory in Sections 6, 7. Most importantly, 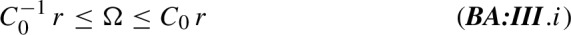 and the remaining assumptions will be collected under the label (***BA:III***).

The complete list of assumptions is given collectively in Appendix B.

*We emphasize that none of the assumptions make explicit reference to the Schwarzschild de Sitter geometry, and capture only some of the features that the relevant “nearby” geometries have in common.* We expect the results of this paper to be an integral part of a general existence theorem for the problem discussed above which in particular would provide as a Corollary non-trivial examples of spacetimes satisfying these assumptions.

In the coordinates $(u,v;\vartheta ^1,\vartheta ^2)$ thus introduced^19^ the spacetime metric takes the form

1.22 $$\begin{aligned} g=-2\Omega ^2\bigl (\mathrm {d}u\otimes \mathrm {d}v+\mathrm {d}v\otimes \mathrm {d}u\bigr )+g\!\!\!/_{AB}\bigl (\mathrm {d}\vartheta ^A-b^A\mathrm {d}v\bigr )\otimes \bigl (\mathrm {d}\vartheta ^B-b^B\mathrm {d}v\bigr )\qquad \end{aligned}$$

In the special case that $b^A=0$, $g\!\!\!/=r^2{\mathop {\gamma }\limits ^{\circ }}$, and $\Omega$ is independent of $\vartheta ^A$, the metric reduces to the spherically symmetric form (1.2). In Section 3 we will discuss various specific choices of the functions *u*, *v* on ${\mathcal {Q}}$ for the Schwarzschild de Sitter metric, and the associated values of the connection coefficients above.

In Section 2.5 we will discuss yet another form of the metric adapted to the decomposition relative to the level sets of the area radius

1.23 $$\begin{aligned} \Sigma _r=\Bigl \{ q\in {\mathcal {R}}: r(q)=r \Bigr \}\,. \end{aligned}$$

By (${\varvec{BA:I}}.i$) these hypersurfaces are always *spacelike*, and the area radius plays the role of a time-function:

1.24 $$\begin{aligned} g=-\phi ^2\mathrm {d}r^2+{\overline{g}}_{r} \end{aligned}$$

where ${\overline{g}}_{r}$ denotes the induced Riemannian metric on $\Sigma _r$.^20^ While this form of the metric is advantageous in some parts of this paper,^21^ and of some importance for the discussion of the asymptotics, we have chosen the double null gauge as the underlying differential structure, because it will allow us to formulate all assumptions that fix the gauge and specify the initial data only on ${\mathcal {C}}\cup \overline{{\mathcal {C}}}$. Moreover, unlike in “non-local” gauges such as “maximal” gauges which fix the mean curvature of the surfaces $\Sigma _r$, the double null gauge allows us to localise any argument to the domain of dependence of any subset of $\Sigma _r$; this will be of importance for the recovery of the assumptions made in this Section.

### Main result

We are interested in the “cosmological region” ${\mathcal {R}}$ to the future of the cosmological horizons ${\mathcal {C}}\cup \overline{{\mathcal {C}}}$; see Fig. 4. Above we have introduced the 2-dimensional closed Riemannian manifolds $(S_{u,v},g\!\!\!/)\subset {\mathcal {R}}$ as the intersections of “ingoing” and “outgoing” null hypersurfaces ${\underline{C}}_v$, and $C_u$, the leaves of a double null foliation of ${\mathcal {R}}$ discussed in Section 1.2. Each sphere $S_{u,v}$ has area $4\pi r^2(u,v)$ as discussed in Section 1.1, and we shall now introduce a dimensionless $\mathrm {L}^p$- norm for tensors $\theta$ on the spheres:

1.25 $$\begin{aligned} \Vert \!\!\!\!-\theta \Vert \!\!\!\!-_{\mathrm {L}^{p}(S)}:=\biggl (\frac{1}{4\pi r^2}\int _{S_{u,v}}|\theta |_{g\!\!\!/}^p\mathrm {d}\mu _{g\!\!\!/}\biggr )^\frac{1}{p} \end{aligned}$$

#### Theorem

Let $({\mathcal {M}},g)$ be a $3+1$-dimensional Lorentzian manifold, ${\mathcal {R}}\subset {\mathcal {M}}$ a domain with past boundary ${\mathcal {C}}\cup \overline{{\mathcal {C}}}$, where ${\mathcal {C}}$ and $\overline{{\mathcal {C}}}$ are future geodesically complete null hypersurfaces intersecting in a sphere $S_\circ$ diffeomorphic to ${\mathbb {S}}^2$. Assume that *g* can be expressed globally on ${\mathcal {R}}$ in the form (1.22) and that the double null foliation of ${\mathcal {R}}$ satisfies the assumptions (**BA:I**-**BA:III**) (as listed in Appendix B).

Suppose the Weyl field *W* is a solution to the Bianchi equations (1.7) and $W\in H^1(\Sigma _{r_\circ })$ where $\Sigma _{r_\circ }\subset {\mathcal {R}}$ is a level set of *r*. Then *W* obeys

1.26 $$\begin{aligned} \Vert \!\!\!\!-W \Vert \!\!\!\!-_{\mathrm {L}^{4}(S)} \le \frac{C}{r^{3}} \Vert W \Vert _{\mathrm {H}^1(\Sigma _{r_\circ })}\qquad \text {: on }{\mathcal {R}} \end{aligned}$$

where $C>0$ only depends on the constants in (**BA:I**-**BA:III**).

#### Remark 1.2

The main estimate could alternatively be stated as

$$\begin{aligned}\Vert W\Vert _{\mathrm {H}^1(\Sigma _r)}^2 := \int _{\Sigma _r}|W|^2+r^2|{\overline{\nabla }}W|\lesssim \frac{1}{r^3}\Vert W\Vert _{\mathrm {H}^1(\Sigma _{r_\circ })}^2\,.\end{aligned}$$

The estimate (1.26) then follows from the Sobolev embedding $W_1^2(\Sigma _r)\hookrightarrow L^6(\Sigma _r)$ which we discuss in Section 7 in the present setting under the assumptions (**BA:I**-**BA:III**). The Sobolev inequality on $\Sigma _r$ is derived from the isoperimetric Sobolev inequality on the spheres $S_{u,v}$, in the course of which we show that the trace operator from $W_1^2(\Sigma _r)\rightarrow L^4(S_{u,v})$ is bounded, which explains the decay statement is in $\mathrm {L}^4$.

#### Remark 1.3

Recall that for the example of Schwarzschild de Sitter spacetime the Weyl curvature satisfies (1.8). In terms of the decay *rate* the Theorem thus states the optimal decay of *all* components of the conformal curvature.^22^ In terms of *regularity*, we do not estimate the curvature in $\mathrm {L}^\infty (S_{u,v})$ (which would require assumptions on the connection coefficients at second order of differentiablity). However, it suffices to control the curvature merely in $\mathrm {L}^4(S_{u,v})$ to obtain a existence for the Einstein equations (1.1).^23^

#### Remark 1.4

A *localised version* of the result holds: Let $\Sigma =\Sigma _{r_0}\cap \{u\le u_0\}\cap \{v\le v_0\}$ be a “segment” of a level set $r=r_0$, and $W\in H^1(\Sigma )$, then (1.26) holds for all $S=S_{u,v}$ *in the domain of dependence of* $\Sigma$, namely $u\le u_0$, $v\le v_0$.

In this paper we do not yet give an existence proof of solutions to (1.1) in ${\mathcal {R}}$. The theorem establishes what we expect to be the first part of a larger *bootstrap*, or *continuous induction* argument. The task of the second part is to establish the converse, namely that *assuming* $\mathrm {L}^4$-bounds on the Weyl curvature, we seek to *prove* the $\mathrm {L}^\infty$, and $\mathrm {L}^4$-estimates (***BA***) on the connection coefficients, *under suitable assumptions on the characterisitc initial data*. In a simplified and restricted — yet very instructive — setting, this is achieved in [39]; see Section 1.5 for further comments.

#### Remark 1.5

The assumptions on the initial data in the Theorem are made on the spacelike hypersurface $\Sigma _{r_0}$. In the context of the non-linear problem described on page 5 geometric initial data is prescribed on the characteristic null hypersurfaces ${\mathcal {C}}\cup \overline{{\mathcal {C}}}$. Proceeding similarly to Chapter 2 in [8] we can derive from the null constraint equations — and now using the exponential decay of the energy density as defined in (2.70) in [8] — the precise behaviour of all geometric along the cosmological horizons, and we expect that analogously to the scalar case — see Section 4.2 in [38] — the “local redshift effect” (due to the positive “surface gravity”) can be exploited to prove a local existence result up to $\Sigma _{r_0}$, such that all decay properties towards $\iota ^+$ are inherited. Besides a direct application to the Weyl curvature, the above theorem may also be applied to Weyl fields corresponding to suitable renormalisations of the Weyl curvature. These constructions are independent of the results in this paper.

### Comments on the proof

The proof is largely a treatment of the Bianchi equations (1.7) for the Weyl curvature:

1.27 $$\begin{aligned} \nabla _\alpha W^\alpha _{\beta \gamma \delta }=0 \end{aligned}$$

In analogy to Maxwell’s theory, one can construct an energy-momentum tensor *Q*(*W*), the Bel-Robinson tensor

1.28 $$\begin{aligned} Q[W]_{\alpha \beta \gamma \delta }= W_{\alpha \rho \gamma \sigma }W_{\beta \delta }^{\rho \sigma }+{}^*W_{\alpha \rho \gamma \sigma }{}^*W_{\beta \delta }^{\rho \sigma } \end{aligned}$$

which has the well-known properties — see for example Chapter 7.1 in [10] — that it is *positive* when evaluated on any causal future-directed vectors at a point (Proposition 4.2 in [9]), and *divergence-free* if *W* is a solution to (1.27) (Proposition 4.4 in [9]):

1.29 $$\begin{aligned} \nabla ^\alpha Q[W]_{\alpha \beta \gamma \delta }=0\,. \end{aligned}$$

This allows us define *energy currents* with the help of “multiplier vectorfields” *X*, *Y*, *Z*,

1.30 $$\begin{aligned} P[W]^{(X,Y,Z)}_\alpha =-Q[W]_{\alpha \beta \gamma \delta }X^\beta Y^\gamma Z^\delta \end{aligned}$$

which give rise to *energy identities*; see derivations in Section 4.1. An important example in the context of this paper are energy identities on space-time domains bounded by the level sets $\Sigma _r$:

1.31 $$\begin{aligned} {\mathcal {D}}=\bigcup _{r_1\le r \le r_2}\Sigma _r \end{aligned}$$

The usefulness of the resulting identity,

1.32 $$\begin{aligned}&\int _{\Sigma _{r_2}}Q(n,X,Y,Z)\mathrm {d}\mu _{{\overline{g}}_{r_2}}+ \int _{{\mathcal {D}}}{{\,\mathrm{div}\,}}(-P)[W]^{(X,Y,Z)}\mathrm {d}\mu _{g}\nonumber \\&\quad = \int _{\Sigma _{r_1}}Q(n,X,Y,Z)\mathrm {d}\mu _{{\overline{g}}_{r_1}} \end{aligned}$$

where *n* denotes the (future directed) unit normal to $\Sigma _r$, depends crucially on the properties of the “divergence term”, or “bulk term” on ${\mathcal {D}}$. By (1.29) the integrand is given purely by a contraction of *Q*(*W*) with the “deformation tensor” of the multiplier vectorfields:

1.33 $$\begin{aligned} {}^{(X)}\pi ={\mathcal {L}}_X g \end{aligned}$$

In fact, since *Q*(*W*) is *trace-free* (and symmetric) in all indices, only the “trace-free part” of these tensors appear:

1.34 $$\begin{aligned} {}^{(X)}{\hat{\pi }}={}^{(X)}\pi -\frac{1}{4}g{{\,\mathrm{tr}\,}}{}^{(X)}\pi \end{aligned}$$

In Section 4.2 we construct a multiplier vectorfield *M* and prove that the associated divergence terms have *a sign* which yields by (1.32) a *monotone* energy. In fact, we will use

1.35 $$\begin{aligned} M=\frac{1}{2}\frac{1}{\Omega }\Bigl (\hat{{\underline{L}}}+\hat{{L}}\Bigr ) \end{aligned}$$

to prove in Section 4.3 that the current

1.36 $$\begin{aligned} P[W]^{M}_\alpha :=P[W]^{(M,M,M)}_\alpha \end{aligned}$$

has the following positivity property:

*Suppose the connection coefficients satisfy the assumptions* (***BA:I***). *Then for any solution* *W* *to* (1.27):

1.37 $$\begin{aligned} \int _{{\mathcal {D}}}{{\,\mathrm{div}\,}}(-P)[W]^{M}\mathrm {d}\mu _{g} \ge \int _{r_1}^{r_2}\frac{6}{r}\int _{\Sigma _r}Q[W](n,M,M,M)\mathrm {d}\mu _{{\overline{g}}_{r_1}} \mathrm {d}r \end{aligned}$$

The existence of such a positive current is obviously intimately related to the assumption (${\varvec{BA:I}}.i$),^24^ and the numerical prefactor in the above inequality is important, because it will directly translate into the decay rate of the energy associated to *M*.

This part of the argument is in close analogy to my treatment of linear waves on Kerr de Sitter cosmologies in [38]; cf. discussion in Section 4.2. The inequality (1.37) lends itself to the interpretation that *M* captures the classical *redshift effect* in the cosmological region,^25^ in the language of “compatible currents”; we will thus often refer to *M* as the “global redshift vectorfield”.

In the context of the linear wave equation on Schwarzschild de Sitter spacetimes, the treatment of *higher order* energies, and pointwise estimates is a trivial extension of the “global redshift estimate” because the tangent space to $\Sigma _r$ is in this case spanned by Killing vectorfields. Indeed the commutation of (1.4) with the generators of the spherical isometries of $S_{u,v}$, and of the translational isometry *T* along $\Sigma _r$ immediately gives the desired higher order energy estimates in [38]. In the present context, however, this approach is not very fruitful, because even if we were to construct generators $\Omega _{(i)}$ and *T* of “spherical” and “translational” actions on $\Sigma _r$, these actions cannot be expected to generate *asymptotic symmetries* (as in [10]; because unlike in the asymptotically flat case future null infinity may not possess *any* symmetries).

In our approach then we use the global redshift vectorfield also as a *commutator*.^26^

In general, the conformal properties of the Bianchi equations — see e.g. Chapter 12.1 in [8] — allow us to define a “modified Lie derivative” $\tilde{{\mathcal {L}}}_{X}W$ with respect to any vectorfield *X* of a solution *W* to (1.27) which satisfies the *inhomogeneous* Bianchi equations

1.38 $$\begin{aligned} \nabla ^\alpha \bigl (\tilde{{\mathcal {L}}}_{X}W\bigr )_{\alpha \beta \gamma \delta }={}^{(X)}J(W)_{\beta \gamma \delta } \end{aligned}$$

where ${}^{(X)}J(W)$ is a “Weyl current” which can be expressed in terms of contractions of ${}^{(X)}{\hat{\pi }}$ with $\nabla W$, and contractions of $\nabla {}^{(X)}{\hat{\pi }}$ with *W*; see e.g. Proposition 12.1 in [8]. In the presence of an inhomogeneity in the Bianchi equations, the divergence-free property of the associated Bel-Robinson tensor fails, and (1.29) is replaced by

1.39 $$\begin{aligned} \begin{aligned} \nabla ^\alpha Q[\tilde{{\mathcal {L}}}_{X}W]_{\alpha \beta \gamma \delta }=&W_{\beta \delta }^{\mu \nu }{}^{(X)}J_{\mu \gamma \nu }(W)+ W_{\beta \gamma }^{\mu \nu }{}^{(X)}J_{\mu \delta \nu }(W)\\&+{}^*W_{\beta \delta }^{\mu \nu }{}^{(X)}J^*_{\mu \gamma \nu }(W)+{}^*W_{\beta \gamma }^{\mu \nu }{}^{(X)}J^*_{\mu \delta \nu }(W)\,. \end{aligned} \end{aligned}$$

Consequently the energy identity derived from the current

1.40 $$\begin{aligned} P[\tilde{{\mathcal {L}}}_{X}W]^{M}_\alpha =-Q[\tilde{{\mathcal {L}}}_{X}W]_{\alpha \beta \gamma \delta }M^\beta M^\gamma M^\delta \end{aligned}$$

contains an additional “divergence term” of the form

1.41 $$\begin{aligned} \int _{{\mathcal {D}}}({{\,\mathrm{div}\,}}Q[\tilde{{\mathcal {L}}}_{X}W])(M,M,M)\mathrm {d}\mu _{g}\,. \end{aligned}$$

A major difficulty in the proof lies in showing that this contribution to the “bulk term” can *also* be arranged to have a sign.

As already indicated above, one could choose $X=M$, but this choice does not succeed.

#### Remark 1.6

While it is possible to show that (under suitable assumptions) “at the highest order of derivatives” the current (1.40) has the following positivity property:

1.42 $$\begin{aligned}&\int _{{\mathcal {D}}}({{\,\mathrm{div}\,}}Q[\tilde{{\mathcal {L}}}_{M}W])(M,M,M)\mathrm {d}\mu _{g} \nonumber \\&\quad \ge \int _{r_1}^{r_2}\frac{4}{r}\int _{\Sigma _r}Q[\tilde{{\mathcal {L}}}_{M}W](n,M,M,M)\mathrm {d}\mu _{{\overline{g}}_{r}} \mathrm {d}r+\int _{{\mathcal {D}}}{\mathcal {E}}\mathrm {d}\mu _{g} \end{aligned}$$

However, the “lower order terms” contained in the “error” ${\mathcal {E}}$ — which are on the level of *W* and can in principle be controlled by the energy associated to $P^M[W]$ — do not decay fast enough towards ${\mathcal {I}}^+$, for this energy estimate to give the *rate of decay* of the energy associated to $P^M[\tilde{{\mathcal {L}}}_{M}W]$ that would be required (in the application of the Sobolev inequality) to prove the $\mathrm {L}^4$-bound stated in the theorem.

The treatment of the “commutation”, or “first order energy” thus becomes the most complex part of this paper, because it requires us not only to find a sign in the divergence, but also to exhibit *various cancellations in the lower order terms*.^27^ This is the reason why we are forced to compute very carefully most terms contained in (1.41), including the signs and prefactors.^28^

It turns out that a suitable choice of a commutation vectorfield is given by

1.43 $$\begin{aligned} X=\Omega ^2 M_q \end{aligned}$$

where

1.44 $$\begin{aligned} M_q=\frac{1}{2}\frac{1}{\Omega }\Bigl (q\hat{{\underline{L}}}+q^{-1}\hat{{L}}\Bigr )\,,\qquad q= \sqrt{\frac{\overline{\Omega {{\,\mathrm{tr}\,}}\chi }}{\overline{\Omega {{\,\mathrm{tr}\,}}{\underline{\chi }}}}}\,. \end{aligned}$$

One can think of the map $M\mapsto M_q$ as induced by a Lorentz transformation with the effect of aligning $M_q$ with the normal *n* to $\Sigma _r$. The final commutation vectorfield *X*, subsequently denoted by *N*, is then obtained from $M_q$ by scaling with the weight $\Omega ^2$.

In Section 5 we shall prove the following:

*Suppose the connection coefficients satisfy the assumptions* (***BA:I***) *and* (***BA:II***). *Then there exists a constant* $C>0$, *such that for any solution* *W* *to* (1.27):

1.45 $$\begin{aligned} \int _{{\mathcal {D}}}{{\,\mathrm{div}\,}}(-P)[\tilde{{\mathcal {L}}}_{N}W]^{M_q}\,\mathrm {d}\mu _{g}\ge & {} \int _{r_1}^{r_2}\frac{6}{r}\int _{\Sigma _r}Q[\tilde{{\mathcal {L}}}_{N}W](n,M_q,M_q,M_q)\mathrm {d}\mu _{{\overline{g}}_{r_1}} \mathrm {d}r\nonumber \\&\quad -\,\int _{r_1}^{r_2}\frac{C}{r}\int _{\Sigma _r}\frac{1}{\Omega } Q[W](n,M_q,M_q,M_q)\mathrm {d}\mu _{{\overline{g}}_{r}}\mathrm {d}r\nonumber \\&\quad -\,\int _{r_1}^{r_2}\frac{C}{r} \int _{\Sigma _r}\frac{1}{\Omega ^2}|{\overline{\nabla }}W |^2\mathrm {d}\mu _{{\overline{g}}_{r}}\mathrm {d}r \end{aligned}$$

In this estimate we have achieved that the “error” (second term on the r.h.s.) is small — because $\Omega ^{-1}$ is small — and controlled by the energy associated to $P[\tilde{{\mathcal {L}}}_{N}W]^{M_q}$ and $P[W]^{M_q}$, up to terms which only involve *tangential derivatives* to $\Sigma _r$ (third term). Next we prove that the latter can be controlled by the first two terms, but it is again a non-trivial statement that in this estimate no “lower order terms” appear, which would obstruct the “redshift” gained with the positivity of the first term on the r.h.s. of (1.45).

The “electromagnetic decomposition” of a Weyl field *W* relative to $\Sigma _r$ — much like the decomposition of the Faraday tensor *F* in electric and magnetic fields *E*, and *H* relative to a given frame of reference — recast the Bianchi equations in a system akin to Maxwell’s equations:

1.46a $$\begin{aligned} {\overline{{{\,\mathrm{div}\,}}}}E= & {} H\wedge k \qquad \widehat{{\mathcal {L}}_nH}+{\overline{{{\,\mathrm{curl}\,}}}}E+\frac{1}{2}k\times H={\overline{\nabla }}\log \phi \wedge E \end{aligned}$$

1.46b $$\begin{aligned} {\overline{{{\,\mathrm{div}\,}}}}H= & {} -E\wedge k\qquad \widehat{{\mathcal {L}}_nE}-{\overline{{{\,\mathrm{curl}\,}}}}H+\frac{1}{2}k\times E=-{\overline{\nabla }}\log \phi \wedge H \end{aligned}$$

In Section 6 we derive an elliptic estimate for this Hodge system on $\Sigma _r$^29^ that allows us to control all tangential derivatives to $\Sigma _r$ by the energy associated to $P[\tilde{{\mathcal {L}}}_{N}W]^{(M_q)}$. Here it is essential that the commutator vectorfield *N* has been aligned with the normal *n* to $\Sigma _r$, and carries a weight that leads again to exact cancellations with the “lower order terms” (on the level of the second fundamental form *k* of $\Sigma _r$) present in (1.46).

*Suppose the assumptions* (***BA:I***) *and* (***BA:III***) *hold. Then there exists a constant* $C>0$ *such that for all solutions to* (1.46),

1.47 $$\begin{aligned} \int _{\Sigma _r} \frac{1}{\Omega ^2}|{\overline{\nabla }}W|^2 \mathrm {d}\mu _{{\overline{g}}_{r}}\le & {} C \int _{\Sigma _r}\frac{1}{\Omega } Q[\tilde{{\mathcal {L}}}_{N}W](n,M_q,M_q,M_q)\mathrm {d}\mu _{{\overline{g}}_{r}}\nonumber \\&\quad +\,C\int _{\Sigma _r}\frac{1}{\Omega }Q[W](n,M_q,M_q,M_q)\mathrm {d}\mu _{{\overline{g}}_{r}} \end{aligned}$$

In conclusion, we obtain under the assumptions (***BA:I-III***), stated with a slight abuse of notation and freely using (${\varvec{BA:III}}.i$), that

1.48 $$\begin{aligned} \int _{\Sigma _r}|W |^2 + r^2 |{\overline{\nabla }}W |^2 \mathrm {d}\mu _{{\overline{g}}_{r}}\lesssim \frac{1}{r^{3}} \end{aligned}$$

which then easily implies the statement of the theorem by a Sobolev trace inequality on $\Sigma _r$ which we discuss in Section 7.

### Further comments on the assumptions

We outline briefly how some of the assumptions (***BA:I***) made in this paper are recovered and refer to [39] for a more elaborate discussion in a simplified setting. Recall the task is here to prove the estimates $({{\varvec{BA}}})$ under suitable assumptions on the initial data, using the bounds on the Weyl curvature established in this paper. In [39] this is carried out in a drastically “simplified” setting where the Weyl curvature not only decays, but vanishes identically.^30^ It is also “restricted” in the sense that the initial data on one of the cosmological horizons is shear-free.^31^

Among the most important assumptions are (${\varvec{BA:I}}.i$), (${\varvec{BA:I}}.ii$) and (${{\textbf {BA:I}}.vi}^\prime$). Note that while $2\omega$ and $\Omega {{\,\mathrm{tr}\,}}\chi$ are both linearly growing in *r*, their difference is assumed to be bounded. To prove this, one considers the difference of the propagation equations for $2\omega$, and $\Omega {{\,\mathrm{tr}\,}}\chi$, namely the equations for ${\underline{L}}(2\omega )$ and ${\underline{L}}(\Omega {{\,\mathrm{tr}\,}}\chi )$ which are at the level of curvature, and one finds using the Einstein equations (1.1) that^32^

1.49 $$\begin{aligned} {\underline{L}}\bigl (2\omega -\Omega {{\,\mathrm{tr}\,}}\chi \bigr ) = -2\Omega ^2\Bigl (2\rho [W]-\frac{1}{2}\bigl ({\underline{\chi }},\chi )+{{\,\mathrm{div}\,}}\!\!\!\!\!/\,\eta +|\eta |^2 -2(\eta ,{\underline{\eta }})+|{\underline{\eta }}|^2+\frac{\Lambda }{3}\Bigr )\nonumber \\ \end{aligned}$$

Now in [39] we define the *mass aspect function* by

1.50 $$\begin{aligned} \mu :=-\rho [W]+\frac{1}{2}({\hat{\chi }},{\hat{{\underline{\chi }}}})-{{\,\mathrm{div}\,}}\!\!\!\!\!/\,\eta = K-{{\,\mathrm{div}\,}}\!\!\!\!\!/\,\eta +\frac{1}{4}{{\,\mathrm{tr}\,}}\chi {{\,\mathrm{tr}\,}}{\underline{\chi }}-\frac{\Lambda }{3} \end{aligned}$$

where in the second equality we have used the Gauss equation (2.43) which relates the Gauss curvature *K* of $(S_{u,v},g\!\!\!/)$ to the ambient Weyl curvature. Therefore

1.51 $$\begin{aligned} {\underline{L}}\bigl (2\omega -\Omega {{\,\mathrm{tr}\,}}\chi \bigr ) = -2\Omega ^2\Bigl (\rho [W]-\mu +|\eta |^2 -2(\eta ,{\underline{\eta }})+|{\underline{\eta }}|^2-\frac{1}{4}{{\,\mathrm{tr}\,}}\chi {{\,\mathrm{tr}\,}}{\underline{\chi }}+\frac{\Lambda }{3}\Bigr ),\nonumber \\ \end{aligned}$$

thus reducing the boundedness of $2\omega -\Omega {{\,\mathrm{tr}\,}}\chi$ to decay properties of $\rho [W]$, and $\mu$, and also $\eta$, ${\underline{\eta }}$, and $(\Lambda /3-{{\,\mathrm{tr}\,}}\chi {{\,\mathrm{tr}\,}}{\underline{\chi }}/4)$. The mass aspect function plays an equally central role as in [10]; (cf. Section 5.3 in [39]): It satisfies a “good” propagation equation in the sense that one can hope to prove that $\mu ={\mathcal {O}}(r^{-3})$, provided various final gauge choices are satisfied. Once such a bound on $\mu$ is obtained, the idea is to view the definition of (1.50) as part of an elliptic system on $S_{u,v}$ for the torsion $\eta$:

1.52a $$\begin{aligned} {{\,\mathrm{div}\,}}\!\!\!\!\!/\,\eta&=-\rho [W]+\frac{1}{2}({\hat{\chi }},{\hat{{\underline{\chi }}}})-\mu \end{aligned}$$

1.52b $$\begin{aligned} {{\,\mathrm{curl}\,}}\!\!\!\!\!/\,\eta&= -\frac{1}{2}{\hat{\chi }}\wedge {\hat{{\underline{\chi }}}}+\sigma [W] \end{aligned}$$

The assumption (${{\textbf {BA:I}}.vi}^\prime$) on $\eta$ can then be recovered using elliptic estimates — which hold under sufficient control on the isoperimetric constant of $(S_{u,v},g\!\!\!/)$ — and the established bounds on the Weyl curvature; (cf. Section 6, 7 in [39]).

A key remaining difficulty in the recovery is the above mentioned “final gauge choice”, which needs to be addressed in order to be able to choose various boundary values for the propagation equations of the structure coefficients. More precisely, given a spacetime domain ${\mathcal {D}}=\cup _{r_1\le r\le r_2}\Sigma _r=\cup _{r_1\le r(u,v)\le r_1}S_{u,v}$ it involves the geometric construction of a family of new spheres $S_{u,v}'$ in ${\mathcal {D}}$, such that $\mu$, ${{\,\mathrm{tr}\,}}\chi$, and ${{\,\mathrm{tr}\,}}\chi$ assume specific values; for a solution to this problem in a different setting see [28].

### Relation to earlier work

We have already mentioned the work of Hintz and Vasy on the non-linear stability of the Kerr-de Sitter family in the black hole exterior on the domain bounded by the cosmological horizon [27].^33^ Another important result to be mentioned in this context is the work of Friedrich on the stability of the de Sitter spacetime [20]. We will discuss here briefly its relevance to the stability problem for *Schwarzschild-*de Sitter cosmologies. Finally we will mention the work of Ringström [33], and Rodnianski and Speck [34, 40, 41].

**Fig. 6:**
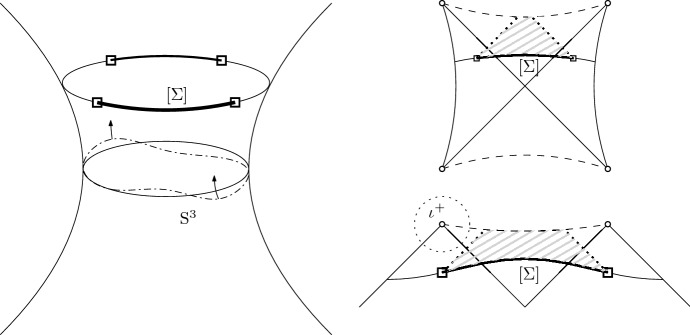
*Bottom right:* Truncation $[\Sigma ]$ of Cauchy hypersurface $\Sigma$ away from the event horizons, and domain of dependence of $[\Sigma ]$ (shaded). *Top right:* $[\Sigma ]$ viewed as “perturbation” of a segment of a hypersurface in de Sitter spacetime. *Left:* Sketch of initial data for Friedrich’s theorem (dash-dotted); Initial data corresponding to $[\Sigma ]$ as a subset of ${\mathbb {S}}^3$ (bold, between “truncation spheres” depicted as squares).

In [20] Friedrich proved that the future development of Cauchy data on ${\mathbb {S}}^3$ is *geodesically complete*, provided the initial data is “a small perturbation” of the datum induced by the de Sitter solution. Now with regard to the initial data induced by a Schwarzschild de Sitter solution, a Cauchy hypersurface $\Sigma$ as in Fig. 3*cannot* be expressed as a “perturbation” of de Sitter data. However, a *truncation* $[\Sigma ]$ of $\Sigma$ *away from the event horizons* could be viewed as a perturbation of a suitable “segment” of de Sitter data, at least for small mass $0<m\ll 1/(3\sqrt{\Lambda })$; see Fig. 6 (right). It is plausible that the resulting data on $[\Sigma ]\simeq [\delta ,\pi -\delta ]\times {\mathbb {S}}^2$ can be glued to the “spherical caps” $[0,\delta ]\times {\mathbb {S}}^2$ of an ${\mathbb {S}}^3$, to obtain an admissible initial data set for [20]; see Fig. 6 (left).^34^ The result of Friedrich would then yield a geodesically complete spacetime, which agrees with the future development of $\Sigma$ *on the domain of dependence* of $[\Sigma ]$; see Fig. 6 (shaded). Thus [20] could be used to show the stability of regions ${\mathcal {D}}_F$ realized as the past of spatially compact segments $\Sigma _c^+$ of ${\mathcal {I}}^+$,

1.53 $$\begin{aligned} {\mathcal {D}}_F\subset I^-(\Sigma ^+_c)\qquad \Sigma _c^+\subset {\mathcal {I}}^+\simeq {\mathbb {R}}\times {\mathbb {S}}^2 \end{aligned}$$

Notably, such an argument cannot achieve a stability statement “in a neighborhood of time-like infinity $\iota ^+$;” see Fig. 6.

Another approach to implement the conformal method in the Schwarzschild de Sitter setting has been pursued in [21]. There it is found that initial data for the conformal field equations on a $\Sigma _r$ hypersurface can only be constructed under strong fall off assumptions to trivial data which amount to excluding the points $\iota ^+$ from the analysis. This still yields a “localised” result in the sense of Remark 1.4, and gives an interesting discussion of the geometric data on ${\mathcal {I}}^+$.

While the results in [20] are closely related to the conformal properties of (1.1), and achieve a global existence result by a reduction to a “local in time” problem, Ringström provided a treatment of the “Einstein-non-linear scalar field system” — which includes the Einstein vacuum equations with positive cosmological constant as a special case — that reproves the results in [20], without resorting to a “conformal compactification”, and without specific reference to the topology of the initial data [33]. In fact, the set-up in [33] exploits a causal feature of “accelerated expansion” already evident from the Penrose diagram of de Sitter spacetime, cf. Fig. 6 (right): Consider a spacelike Cauchy hypersurface $\Sigma$ in de Sitter spacetime, let $[\Sigma ]\subset {\mathcal {R}}$ be a truncation of $\Sigma$ contained in the expanding region, and $p\in [\Sigma ]$. Choose $R>0$ such that $B_{4R}(p)\subset [\Sigma ]$, then the future of $B_R(p)$ is contained in the domain of dependence of $B_{4R}(p)$, $I^+(B_R(p))\subset {\mathcal {R}}\setminus I^+(\Sigma \setminus B_{4R}(p))$, provided $\Sigma$ is “at sufficiently late time”, e.g.  if $\min _{[\Sigma ]} r\gg {r_{{\mathcal {C}}}}$. This allows Ringström to prove “global in time” results, from “local in space” assumptions on the inital data, which cover in particular perturbations of the de Sitter solution, but are not restricted to the ${\mathbb {S}}^3$ topology. Notably, the “asymptotic expansions” of Theorem 2 in [33] show the existence of “asymptotic functional degrees of freedom”, namely that the solution converges to a metric which after rescaling by the expected behavior in time differs from the rescaled de Sitter metric, even at the leading order parametrized by a free “profile” function.^35^

This paper does not yet give a full global existence theorem for solutions to (1.1) on the level of [20, 27, 33]. It does however accomplish what one expects to be an essential step towards the stability of the expanding region of *Schwarzschild-* de Sitter cosmologies: We show that the Weyl curvature decays under sufficiently general assumptions — roughly corresponding to Part II of the original proof of the non-linear stability of Minkowski space [10].

The underlying decay mechanism — namely the expansion of spacetime — has also played a prominent role in the work of Speck on Friedman-Lemaître-Robertson-Walker cosmologies: They were shown to be future stable in [23, 34, 40] as solutions to the Euler-Einstein system, and it was observed in particular that the “de Sitter”-like expansion prevents the formation of shocks in relativistic fluids (with linear barotropic equation of state) [41]. For stiff fluids Rodnianski and Speck also showed stable “big bang” singularity formulation in the past [35, 36]. Some elements of their proof — in particular the existence of a monotone energy at the level of the commuted equations, the resulting smallness of the Weyl curvature, and the functional degrees of freedoms associated to all possible “end states” — bear some resemblance to the approach pursued in this paper.^36^

Finally while we do use [8] as our primary reference throughout for quoting various formulas related to the double null formalism, many of the central propositions in particular related to the construction of currents of course first appeared in [10] and [9].

## Einstein’s equations with cosmological constant

The Einstein vacuum equations with positive cosmological constant $\Lambda >0$ are

2.1 $$\begin{aligned} {{\,\mathrm{Ric}\,}}(g)=\Lambda g \end{aligned}$$

### Weyl curvature

Presently we shall focus on the conformal decomposition of the curvature tensor of a $3+1$-dimensional spacetime manifold, which plays an important role in this context.

Recall the *Schouten* tensor

2.2 $$\begin{aligned} P_{\alpha \beta }=\frac{1}{2}\Bigl [{{\,\mathrm{Ric}\,}}_{\alpha \beta }-\frac{R}{6}g_{\alpha \beta }\Bigr ] \end{aligned}$$

where *R* denotes the scalar curvature; see e.g. [19]. We observe that for any solution to (2.1) the Schouten tensor is simply

2.3 $$\begin{aligned} P_{\alpha \beta }=\frac{\Lambda }{6}g_{\alpha \beta } \end{aligned}$$

The *Weyl* curvature *W*, in general, is defined by [19]

2.4 $$\begin{aligned} W_{\alpha \beta \mu \nu }=R_{\alpha \beta \mu \nu }+\Bigl [P_{\beta \mu }g_{\alpha \nu }+P_{\alpha \nu }g_{\beta \mu }-P_{\beta \nu }g_{\alpha \mu }-P_{\alpha \mu }g_{\beta \nu }\Bigr ] \end{aligned}$$

which for solutions to (2.1) then reduces to:

2.5 $$\begin{aligned} W_{\alpha \beta \mu \nu }=R_{\alpha \beta \mu \nu }+\frac{\Lambda }{3}\Bigl [g_{\alpha \nu } g_{\beta \mu }-g_{\alpha \mu }g_{\beta \nu }\Bigr ] \end{aligned}$$

Note that *W* has the same algebraic symmetries as the curvature tensor *R*, and in addition is totally trace-free. We shall thus proceed in Section 2.2 with the null decompositon of the *Weyl* curvature.

### Null decomposition of the Weyl curvature

We have already referred to the symmetries of the Weyl curvature. We note that the Weyl curvature (2.5) is a “Weyl field” in the sense of Chapter 12 in [8]: It is anti-symmetric in the first two and last two indices, and satisfies the cyclic identity:

2.6 $$\begin{aligned} W_{\alpha [\beta \mu \nu ]}=0 \end{aligned}$$

Moreover, the Weyl curvature satisfies the trace conditon:

2.7 $$\begin{aligned} g^{\alpha \mu }W_{\alpha \beta \mu \nu }={{\,\mathrm{Ric}\,}}_{\beta \nu }+\frac{\Lambda }{3}\Bigl [g_{\beta \nu }-4g_{\beta \nu }\Bigr ]=0 \end{aligned}$$

The dual of *W* is defined by (as we know, left and right duals coincide)

2.8 $$\begin{aligned} W_{\alpha \beta \mu \nu }^*=\frac{1}{2}W_{\alpha \beta }^{\gamma \delta }\epsilon _{\gamma \delta \mu \nu } \end{aligned}$$

There are 10 algebraically independent components of a Weyl field. Let $(e_A:A=1,2;e_3,e_4)$ be an orthonormal null frame field. Then the 2-covariant tensorfields

2.9 $$\begin{aligned} {\underline{\alpha }}_{AB}[W]=W_{A3B3} \quad \alpha _{AB}[W]=W_{A4B4} \end{aligned}$$

account for 2 components each, because they are symmetric and trace-free:

2.10 $$\begin{aligned} g^{AB}{\underline{\alpha }}_{AB}=g^{AB}W_{A3B3}=g^{\mu \nu }W_{\mu 3\nu 3}=0 \end{aligned}$$

Also the 1-forms

2.11 $$\begin{aligned} {\underline{\beta }}_A[W]=\frac{1}{2}W_{A334}\quad \beta _A[W]=\frac{1}{2}W_{A434} \end{aligned}$$

account for 2 components each, which leaves us with 2 functions

2.12 $$\begin{aligned} \rho [W]=\frac{1}{4}W_{3434}\quad \sigma [W] \epsilon _{AB}=\frac{1}{2}W_{AB34} \end{aligned}$$

Note that with (2.5) we have

2.13 $$\begin{aligned} {\underline{\alpha }}_{AB}[W]= & {} R_{A3B3}\qquad \alpha _{AB}[W]=R_{A4B4} \end{aligned}$$

2.14 $$\begin{aligned} {\underline{\beta }}_A[W]= & {} R_{A334}\qquad \beta _A[W]=R_{A434} \end{aligned}$$

2.15 $$\begin{aligned} \rho [W]= & {} \frac{1}{4}R_{3434}+\frac{\Lambda }{3}\qquad \sigma [W]\epsilon _{AB}=\frac{1}{2} R_{AB34} \end{aligned}$$

Here we used $g_{33}=g_{3A}=g_{44}=g_{4A}=0$, and $g_{34}=-2$.

Thus the only component that differs from the corresponding null decompositon of the curvature tensor *R* (which is *only* a Weyl field in the case $\Lambda =0$) is $\rho$.

Note that $\sigma [W]$ can equally be defined by

2.16 $$\begin{aligned} \begin{aligned} \sigma [W]&=\rho [{}^*W]=\frac{1}{4}{}^*W_{3434}=\frac{1}{4}W_{34}^{\alpha \beta }\epsilon _{\alpha \beta 34}\\&=\frac{1}{4}W_{34}^{12}+\frac{1}{4}W_{34}^{21}(-1)=\frac{1}{2}R_{3412} \end{aligned} \end{aligned}$$

The remaining components of *W* are expressed as, cf. (12.34) in [8],

2.17a $$\begin{aligned} W_{A3BC}&=g_{AB}{\underline{\beta }}_C[W]-g_{AC}{\underline{\beta }}_B[W]=\frac{1}{2}g_{AB}R_{C334}-\frac{1}{2}g_{AC}R_{B334} \end{aligned}$$

2.17b $$\begin{aligned} W_{A4BC}&=-g_{AB}\beta _C[W]+g_{AC}\beta _B[W]=-\frac{1}{2}g_{AB}R_{C434}+\frac{1}{2}g_{AC}R_{B434} \end{aligned}$$

2.17c $$\begin{aligned} W_{A3B4}&=-\rho [W]g_{AB}+\sigma [W]\epsilon _{AB}=-\frac{1}{4}R_{3434}\, g_{AB}-\frac{\Lambda }{3}g_{AB}+\frac{1}{2}R_{AB34} \end{aligned}$$

2.17d $$\begin{aligned} W_{ABCD}&=-\rho [W]\epsilon _{AB}\epsilon _{CD}=-\Bigl (\frac{1}{4}R_{3434}+\frac{\Lambda }{3}\Bigr )\bigl (g_{AC}g_{BD}-g_{AD}g_{BC}\bigr ) \end{aligned}$$

We also use the notation ${}^*{\underline{\alpha }}$, ${}^*{\underline{\beta }}$ for the left duals of ${\underline{\alpha }}$ and $\alpha$, respectively, which are related to null decomposition of ${}^*W$ according to (12.35) in [8]:

2.18 $$\begin{aligned} {}^*{\underline{\alpha }}(W)={\underline{\alpha }}({}^*W)\qquad {}^*{\underline{\beta }}(W)={\underline{\beta }}({}^*W)\,. \end{aligned}$$

In terms of an orthonormal frame $(e_A:A=1,2)$:

2.19 $$\begin{aligned} {}^*{\underline{\alpha }}_{AB}= \epsilon \!\!/_{A}^{\sharp C}\alpha _{CB}\qquad {}^*{\underline{\beta }}_A=\epsilon \!\!/_A^{\sharp B}\beta _B \end{aligned}$$

where $2\epsilon \!\!/_{AB}=\epsilon _{AB34}$. Analogous definitions apply to the left duals of $\alpha$, and $\beta$. We note that as ${\underline{\alpha }}$, and $\alpha$, the 2-covariant tensorfields ${}^*{\underline{\alpha }}$, and ${}^*\alpha$ are symmetric and trace-free.

### Bianchi identities

Recall that in general the curvature tensor *R* satisfies the Bianchi identities:

2.20 $$\begin{aligned} \nabla _{\mu }R^\alpha _{\beta \nu \lambda }+\nabla _\nu R^\alpha _{\beta \lambda \mu }+\nabla _\lambda R^\alpha _{\beta \mu \nu }=0 \end{aligned}$$

which, by setting $\alpha =\nu$ and summing, yields the contracted Bianchi identies:

2.21 $$\begin{aligned} \nabla _\alpha R^\alpha _{\beta \lambda \mu }=\nabla _\lambda R_{\mu \beta }-\nabla _\mu R_{\lambda \beta } \end{aligned}$$

Schematically, these equations say

2.22 $$\begin{aligned} {{\,\mathrm{div}\,}}{{\,\mathrm{Riem}\,}}={{\,\mathrm{curl}\,}}{{\,\mathrm{Ric}\,}}\end{aligned}$$

But here, of course, for any solution to the vacuum equations with positive cosmological constant, ${{\,\mathrm{Ric}\,}}(g)=\Lambda g$, and

2.23 $$\begin{aligned} \nabla _\lambda g_{\mu \beta }=0 \end{aligned}$$

by metric compatibility of the connection. Thus, as in the case $\Lambda =0$,

2.24 $$\begin{aligned} \nabla _\alpha R^{\alpha }_{\beta \nu \lambda }=0 \end{aligned}$$

This implies now that for a solution to (2.1) also the Weyl curvature is divergence free:

2.25 $$\begin{aligned}&W^\alpha _{\beta \mu \nu }=R^\alpha _{\beta \mu \nu }+\frac{\Lambda }{3}\Bigl [\delta ^\alpha _{\nu } g_{\beta \mu }-\delta ^\alpha _{\mu }g_{\beta \nu }\Bigr ] \end{aligned}$$

2.26 $$\begin{aligned}&\nabla _\alpha W^\alpha _{\beta \mu \nu }=\frac{\Lambda }{3}\Bigl [\nabla _\nu g_{\beta \nu }-\nabla _\mu g_{\beta \nu }\Bigr ]=0 \end{aligned}$$

With the same formula, (2.25), we see that the “Bianchi identity” (2.20) is also true for the Weyl curvature:

2.27 $$\begin{aligned} \nabla _{\mu }W^\alpha _{\beta \nu \lambda }+\nabla _\nu W^\alpha _{\beta \lambda \mu }+\nabla _\lambda W^\alpha _{\beta \mu \nu }=0 \end{aligned}$$

or, for short, the homogeneous Bianchi equations hold:

2.28 $$\begin{aligned} \nabla _{[\mu }W_{\nu \lambda ]\beta }^{\alpha }=0 \end{aligned}$$

This is consistent with general principles, according to which the equation (2.28), for the Weyl field *W*, also written as

2.29 $$\begin{aligned} D W =0 \end{aligned}$$

is *equivalent* to

2.30 $$\begin{aligned} D {}^*W=0\,, \end{aligned}$$

by the symmetries of a Weyl field, which are precisely the equations (2.26).

Now we are in the situation where we have a Weyl field *W* satisfying (2.26):

2.31 $$\begin{aligned} \nabla _\alpha W^\alpha _{\beta \mu \nu }=0 \end{aligned}$$

Therefore by Proposition 12.4 in [8] the null decomposition of (2.31) takes precisely the form given therein. In other words, the Bianchi equations are *verbatim* those of the vacuum equations in the case $\Lambda =0$, with the understanding that the null components refer to the null decomposition of the Weyl curvature tensor.

### Double null gauge

We follow the conventions of Chapter 1 in [8], for the definition of the double null foliation, and all associated geometric quantities.

We have already introduced in Section 1.2 the *optical functions* *u*, *v* as solutions to the eikonal equations (1.12) such that the surfaces of intersection of the level sets of *u*, *v*, (1.13), are spheres diffeomorphic to ${\mathbb {S}}^2$. Null geodesic normals ${\underline{L}}^\prime$, and $L^\prime$ are introduced as in (1.14), whose components in any coordinate system are

2.32 $$\begin{aligned} {L^\prime }^\mu =-2(g^{-1})^{\mu \nu }\partial _\nu u \qquad {{\underline{L}}^\prime }^\mu =-2(g^{-1})^{\mu \nu }\partial _\nu v \end{aligned}$$

and with their help we have introduced the null lapse function $\Omega$ in (1.15). Note that with

2.33 $$\begin{aligned} {\underline{L}}=\Omega ^2{\underline{L}}^\prime \qquad L=\Omega ^2L^\prime \end{aligned}$$

we have

2.34a $$\begin{aligned} {\underline{L}}u= & {} 1\qquad {\underline{L}}v=0 \end{aligned}$$

2.34b $$\begin{aligned} L u= & {} 0\qquad L v=1 \end{aligned}$$

and

2.35 $$\begin{aligned} g(L,{\underline{L}})=-2\Omega ^2 \end{aligned}$$

Moreover, local coordinates on $S_{u,v}$ are introduced as in Chapter 1.4 in [8]: We choose coordinates $(\vartheta ^1,\vartheta ^2)$ on $S_{0,0}$, which are then transported first along the geodesics generated by ${\underline{L}}^\prime$ on ${\underline{C}}$, and then along the null geodesics generated by $L^\prime$ on $C_u$. In this “canonical coordinate system” the metric takes the form (1.22).

#### Area radius

Recall that we have already introduced in (1.9) the area radius *r*(*u*, *v*) of $S_{u,v}$. Since, by definition

2.36 $$\begin{aligned} 4\pi r^2(u,v)=\int _{S_{u,v}}\mathrm {d}\mu _{g\!\!\!/}=\int _{S_{u,0}}\Phi _v^*\mathrm {d}\mu _{g\!\!\!/} \end{aligned}$$

where $\Phi _v$ is the 1-parameter group generated by *L*, we have

2.37a $$\begin{aligned} D (4\pi r^2)=\int _{S_{u,s}}\Omega {{\,\mathrm{tr}\,}}\chi \mathrm {d}\mu _{g\!\!\!/} \end{aligned}$$

where by definition $Df=Lf$ for any function *f*, and thus

2.37b $$\begin{aligned} Dr= & {} \frac{r}{2}\overline{\Omega {{\,\mathrm{tr}\,}}\chi } \end{aligned}$$

2.37c $$\begin{aligned} {\underline{D}}r= & {} \frac{r}{2}\overline{\Omega {{\,\mathrm{tr}\,}}{\underline{\chi }}} \end{aligned}$$

where ${\overline{\cdot }}$ denotes the average of a function on the sphere

2.38 $$\begin{aligned} {\overline{f}}(u,v) :=\frac{1}{4\pi r^2(u,v)}\int _{S_{u,v}}f(u,v,\vartheta ^1,\vartheta ^2)\mathrm {d}\mu _{g\!\!\!/}(\vartheta ^1,\vartheta ^2)\,. \end{aligned}$$

#### Optical structure coefficients

We have already introduced the normalised null normals $(\hat{{\underline{L}}},\hat{{L}})$ in (1.16), and the null structure coefficients ${\hat{{\underline{\omega }}}}$, and ${\hat{\omega }}$ in (1.18). The null normals $({\underline{L}},L)$ defined in (2.33) then satisfy

2.39 $$\begin{aligned} \nabla _L L=2\omega L\qquad \nabla _{{\underline{L}}}{\underline{L}}=2{\underline{\omega }}{\underline{L}}\end{aligned}$$

where

2.40a $$\begin{aligned} \omega= & {} D\log \Omega \qquad {\underline{\omega }}={\underline{D}}\log \Omega \end{aligned}$$

2.40b $$\begin{aligned} {\hat{\omega }}= & {} \frac{1}{\Omega }\omega \qquad {\hat{{\underline{\omega }}}}=\frac{1}{\Omega }{\underline{\omega }}\,. \end{aligned}$$

The null second fundamental forms $\chi$ and ${\underline{\chi }}$ are defined in (1.17), and its trace-free parts are:

2.41 $$\begin{aligned} {\hat{\chi }}=\chi -\frac{1}{2}g\!\!\!/{{\,\mathrm{tr}\,}}\chi \qquad {\hat{{\underline{\chi }}}}={\underline{\chi }}-\frac{1}{2}g\!\!\!/{{\,\mathrm{tr}\,}}{\underline{\chi }}\,. \end{aligned}$$

We frequently adopt the musical notation (${}^\sharp$) to indicate “raising” an index with $g\!\!\!/$, for instance:

2.42 $$\begin{aligned} ({\hat{\chi }}^{\sharp \sharp })^{AB}=(g\!\!\!/^{-1})^{AC}(g\!\!\!/^{-1})^{BD}{\hat{\chi }}_{CD} \end{aligned}$$

We record here for future reference the Gauss equation of the embedding of $S_{u,v}$ in the spacetime, which relates the Gauss curvature *K* of $(S_{u,v},g\!\!\!/)$ to (the component $\rho$ of) the ambient Weyl curvature *W*:

2.43 $$\begin{aligned} K+\frac{1}{4}{{\,\mathrm{tr}\,}}\chi {{\,\mathrm{tr}\,}}{\underline{\chi }}-\frac{\Lambda }{3}=-\rho [W]+\frac{1}{2}({\hat{\chi }},{\hat{{\underline{\chi }}}}) \end{aligned}$$

In addition to the torsion $\zeta$ defined in (1.21), one may also define a notion of torsion with respect to the null geodesic normals:

2.44 $$\begin{aligned} \eta (X)=\frac{\Omega ^2}{2}g(\nabla _XL^\prime ,{\underline{L}}^\prime )\qquad {\underline{\eta }}(X)=\frac{\Omega ^2}{2}g(\nabla _X{\underline{L}}^\prime ,L^\prime )\qquad :X\in T_{S_{u,v}} \end{aligned}$$

which is related to $\zeta$ via

2.45 $$\begin{aligned} \eta =\zeta +\mathrm {d}\!\!\!/\log \Omega \qquad {\underline{\eta }}=-\zeta +\mathrm {d}\!\!\!/\log \Omega \,. \end{aligned}$$

With the above notation for the structure coefficients we can then express the frame relations as follows:

2.46a $$\begin{aligned} \nabla _X\hat{{L}}= & {} -\zeta (X)\hat{{L}}+\chi ^\sharp \cdot X\,,\qquad \nabla _X\hat{{\underline{L}}}=\zeta (X)\hat{{\underline{L}}}+{\underline{\chi }}^\sharp \cdot X\qquad : X\in \mathrm {T}S_{u,v}\qquad \quad \end{aligned}$$

2.46b $$\begin{aligned} \nabla _{\hat{{L}}}\hat{{\underline{L}}}= & {} -{\hat{\omega }}\hat{{\underline{L}}}+2{\underline{\eta }}^\sharp \,,\qquad \nabla _{\hat{{\underline{L}}}}\hat{{L}}=-{\hat{{\underline{\omega }}}}\hat{{L}}+2\eta ^\sharp \,, \end{aligned}$$

where again $\eta ^\sharp$ and ${\underline{\eta }}^\sharp$ denote the ($S_{u,v}$-tangent) vectorfields corresponding to the 1-forms $\eta$ and ${\underline{\eta }}$, respectively: ${\underline{\eta }}^{\sharp A}=g\!\!\!/^{AB}{\underline{\eta }}_B$, $\eta ^{\sharp A}=g\!\!\!/^{AB}\eta _B$, where $e_A:A=1,2$ is an arbitrary basis for $\mathrm {T}S_{u,v}$, and moreover

2.47 $$\begin{aligned} \chi ^\sharp \cdot e_A= \chi _A^{\sharp C} e_C= (g\!\!\!/^{-1})^{CB}\chi _{AB}e_C \end{aligned}$$

For detailed discussion of the formulas see (1.69, 1.79, 1.86, 1.87) in [8].

### Areal time function

Besides the double null gauge, which is particularly suited for the characteristic initial value problem, other gauges typically involve the choice of a *time function*. This concept appears here naturally in the form of the area radius which is increasing towards to future — this is one manifestation of the expansion of the cosmological region. However, while we do use the decomposition of the Einstein equations relative to a given time-function, we do not impose an equation on its level sets, such as in [10], or [35, 36]; here the time function is chosen once the double null foliation is fixed.

Given an “areal time function” *r*, we define

2.48 $$\begin{aligned} V^\mu =-g^{\mu \nu }\partial _\nu r \end{aligned}$$

and the associated lapse function by

2.49 $$\begin{aligned} \phi =\frac{1}{\sqrt{-g(V,V)}} \end{aligned}$$

Then the unit normal to the level sets of *r*, $\Sigma _r$ is

2.50 $$\begin{aligned} n=\phi V \end{aligned}$$

In the following it will be useful to express these in terms of quantities associated to the double null foliation:

#### Lemma 2.1

The lapse function of the foliation by level sets $\Sigma _r$ is

2.51 $$\begin{aligned} \phi =\frac{2}{r}\frac{\Omega }{\sqrt{\overline{\Omega {{\,\mathrm{tr}\,}}\chi }\,\overline{\Omega {{\,\mathrm{tr}\,}}{\underline{\chi }}}}} \end{aligned}$$

and the normal *n* to each leaf $\Sigma _r$ is given by

2.52 $$\begin{aligned} n=\frac{1}{2}\Bigl (q\hat{{\underline{L}}}+q^{-1} \hat{{L}})\qquad \text {where } q:=\sqrt{\frac{\overline{\Omega {{\,\mathrm{tr}\,}}\chi }}{\overline{\Omega {{\,\mathrm{tr}\,}}{\underline{\chi }}}}} \end{aligned}$$

#### Proof

Here we need explicit expressions for the components of the inverse:

2.53 $$\begin{aligned} \begin{aligned} g^{-1}&=-\frac{1}{2}\hat{{\underline{L}}}\otimes \hat{{L}}-\frac{1}{2}\hat{{L}}\otimes \hat{{\underline{L}}}+\sum _{A=1}^2 E_A\otimes E_A\\&=-\frac{1}{2}\frac{1}{\Omega ^2}{\underline{L}}\otimes L-\frac{1}{2}\frac{1}{\Omega ^2} L\otimes {\underline{L}}+\sum _{A=1}^2E_A\otimes E_A \end{aligned} \end{aligned}$$

so in particular

2.54 $$\begin{aligned} g^{uv}=-\frac{1}{2}\frac{1}{\Omega ^2}\qquad g^{uA}=-\frac{1}{2}\frac{1}{\Omega ^2}b^A\qquad g^{vA}=0\,. \end{aligned}$$

This yields

2.55 $$\begin{aligned} V^u= & {} -g^{uv}\partial _v r=\frac{1}{4}\frac{1}{\Omega ^2}r\overline{\Omega {{\,\mathrm{tr}\,}}\chi }\qquad V^v=\frac{1}{4}\frac{1}{\Omega ^2}r\,\overline{\Omega {{\,\mathrm{tr}\,}}{\underline{\chi }}} \end{aligned}$$

2.56 $$\begin{aligned} V^A= & {} -g^{Au}\partial _u r=\frac{1}{4}\frac{1}{\Omega ^2}b^A r\,\overline{\Omega {{\,\mathrm{tr}\,}}{\underline{\chi }}} \end{aligned}$$

or

2.57 $$\begin{aligned} V=\frac{r}{4}\frac{1}{\Omega }\Bigl (\overline{\Omega {{\,\mathrm{tr}\,}}\chi }\,\hat{{\underline{L}}}+\overline{\Omega {{\,\mathrm{tr}\,}}{\underline{\chi }}}\, \hat{{L}}\Bigr ) \end{aligned}$$

This implies

2.58 $$\begin{aligned} g(V,V)=-\frac{r^2}{4}\frac{1}{\Omega ^2}\,\overline{\Omega {{\,\mathrm{tr}\,}}\chi }\,\overline{\Omega {{\,\mathrm{tr}\,}}{\underline{\chi }}} \end{aligned}$$

and thus the statement of the Lemma. $\square$

#### Induced metric

Let us discuss here the metric on $\Sigma _r$, in particular as *r* tends to infinity. On $\Sigma _r$ we may use $(u,\vartheta ^1,\vartheta ^2)$ as coordinates. Recall from Lemma 2.1 the expression for the normal to $\Sigma _r$, and that in general ${\overline{g}}_{r}$, the induced metric on $\Sigma _r$ is given by

2.59 $$\begin{aligned} {\overline{g}}_{r}(X,Y)=g(\Pi X,\Pi Y)\qquad \Pi X=X+g(n,X)n\,. \end{aligned}$$

##### Lemma 2.2

The metric on $\Sigma _r$ in $(u,\vartheta ^1,\vartheta ^2)$ coordinates, takes the form

2.60 $$\begin{aligned} {\overline{g}}_{r}=q^{-2}\Omega ^2\mathrm {d}u^2+g\!\!\!/_{AB}\mathrm {d}\vartheta ^A\mathrm {d}\vartheta ^B \end{aligned}$$

and the volume form on $\Sigma _r$ is

2.61 $$\begin{aligned} \mathrm {d}\mu _{{\overline{g}}_{r}}=q^{-1}\Omega \,\mathrm {d}u\wedge \mathrm {d}\mu _{g\!\!\!/}=q^{-1}\Omega \sqrt{\det g\!\!\!/}\,\mathrm {d}u \wedge \mathrm {d}\vartheta ^1\wedge \mathrm {d}\vartheta ^2 \end{aligned}$$

##### Proof

Since

2.62 $$\begin{aligned} \partial _u={\underline{L}}=\Omega \hat{{\underline{L}}}\end{aligned}$$

we have

2.63 $$\begin{aligned} \Pi \partial _u=\frac{1}{2}{\underline{L}}-\frac{1}{2}q^{-2}L \end{aligned}$$

and so

2.64 $$\begin{aligned} ({\overline{g}}_{r})_{uu}=q^{-2}\Omega ^2\,. \end{aligned}$$

Moreover,

2.65 $$\begin{aligned}&({\overline{g}}_{r})_{u\vartheta ^A}=g(\Pi \frac{\partial }{\partial u},\frac{\partial }{\partial \vartheta ^A})=0 \end{aligned}$$

2.66 $$\begin{aligned}&({\overline{g}}_{r})_{\vartheta ^A\vartheta ^B}=g\!\!\!/_{AB} \end{aligned}$$

$$\square $$

##### Remark 2.3

There appears no “shift” in the induced metric, because with our present choice the angular coordinates are Lie transported along the ingoing null geodesics.

#### Second fundamental forms

Following the discussion of the first fundamental form, ${\overline{g}}_{r}$, we now turn to the second fundamental form $k_r$ of $\Sigma _r$.

Recall the *Codazzi equations*:

2.67 $$\begin{aligned} ({\overline{\nabla }}_Xk)(Y,Z)-({\overline{\nabla }}_Y k)(X,Z)=R(Z,n,X,Y) \end{aligned}$$

where *X*, *Y*, *Z* are tangent to $\Sigma _r$.

We use a “convenient frame”: $(E_0=n,E_i)$ where $g(E_i,E_j)=\delta _{ij}$ and $[\phi n,E_i]=0$.^37^ Then

2.68 $$\begin{aligned} g_{00}=-1\quad g_{i0}=0 \quad g_{ij}=({\overline{g}}_{r})_{ij} \end{aligned}$$

2.69 $$\begin{aligned} \frac{\partial {\overline{g}}_{ij}}{\partial r}=2\phi k_{ij} \end{aligned}$$

and

2.70a $$\begin{aligned}&{\overline{\nabla }}_i k_{jm}-{\overline{\nabla }}_j k_{im}=R_{m0ij} \end{aligned}$$

2.70b $$\begin{aligned}&{\overline{\nabla }}^m k_{jm}-{\overline{\nabla }}_j{{\,\mathrm{tr}\,}}k={{\,\mathrm{Ric}\,}}_{0j}=\Lambda g_{0j}=0 \end{aligned}$$

Moreover the *Gauss equations* are:

2.71a $$\begin{aligned}&{\overline{R}}_{minj}+k_{mn}k_{ij}-k_{mj}k_{ni}=R_{minj} \end{aligned}$$

2.71b $$\begin{aligned}&{\overline{{{\,\mathrm{Ric}\,}}}}_{ij}+{{\,\mathrm{tr}\,}}k k_{ij}-k_{i}^{m}k_{mj}={{\,\mathrm{Ric}\,}}_{ij}+R_{0i0j} \end{aligned}$$

2.71c $$\begin{aligned}&{\overline{R}}+({{\,\mathrm{tr}\,}}k)^2-|k|^2=R+2{{\,\mathrm{Ric}\,}}_{00}=2\Lambda \end{aligned}$$

The “acceleration of the normal lines” is given by $\nabla _n n={\overline{\nabla }}\log \phi$. In particular if $\phi$ is constant on $\Sigma _r$ the normal lines are geodesics parametrized by arc length. The second variation equation reads in the above frame:

2.72a $$\begin{aligned} \frac{\partial k_{ij}}{\partial r}= & {} {\overline{\nabla }}^2_{ij}\phi +\phi \Bigl \{-R_{i0j0}+k_{i}^{m}k_{mj}\Bigr \} \end{aligned}$$

2.72b $$\begin{aligned} \frac{\partial k_{ij}}{\partial r}= & {} {\overline{\nabla }}^2_{ij}\phi +\phi \Bigl \{ -{\overline{{{\,\mathrm{Ric}\,}}}}_{ij}-{{\,\mathrm{tr}\,}}k k_{ij}+2k_{i}^{m}k_{mj}+\Lambda g_{ij}\Bigr \} \end{aligned}$$

and therefore

2.73 $$\begin{aligned} \begin{aligned} \frac{\partial {{\,\mathrm{tr}\,}}k}{\partial r}=&\frac{\partial }{\partial r}\Bigl (\bigl ({\overline{g}}^{-1}\bigr )^{ij}k_{ij}\Bigr )=\bigl ({\overline{g}}^{-1}\bigr )^{ij}\frac{\partial k_{ij}}{\partial r}-2\phi k^{ij}k_{ij}\\ =&{\overline{\triangle }}\phi -\phi \Bigl ( {\overline{R}}+({{\,\mathrm{tr}\,}}k)^2-3\Lambda \Bigr )={\overline{\triangle }}\phi +\phi \Bigl (\Lambda -|k|^2\Bigr )\,. \end{aligned} \end{aligned}$$

Finally we note the associated connection coefficients:

2.74a $$\begin{aligned} \Gamma _{00}^0= & {} 0\quad \Gamma _{00}^i={\overline{\nabla }}^i\log \phi \end{aligned}$$

2.74b $$\begin{aligned} \Gamma _{i0}^0= & {} 0\quad \Gamma _{i0}^j=k_i^j \end{aligned}$$

2.74c $$\begin{aligned} \Gamma _{0i}^0= & {} {\overline{\nabla }}_i\log \phi \quad \Gamma _{0i}^j=k_i^j \end{aligned}$$

2.74d $$\begin{aligned} \Gamma _{ij}^0= & {} k_{ij}\quad \Gamma _{ij}^m={\overline{\Gamma }}_{ij}^m \end{aligned}$$

For a detailed derivation of these formulas see for example Chapter 4 in [37].

## Schwarzschild de Sitter cosmology

In this Section we briefly discuss some aspects of the geometry of the Schwarzschild de Sitter solution [29, 43]. Its global geometry — as depicted in the Penrose diagram of Fig. 1 — has already been discussed in Section 3 of [38]; cf. [22].

Here we are mainly interested in the values of the structure coefficients for different choices of double null foliations, which has partly motivated our assumptions in Section 1.2. We restrict ourselves to the cosmological region ${\mathcal {R}}$, and *spherically symmetric* foliations.

The formulas derived in this section are not used in the remainder of this paper, but they give the reader the opportunity to familiarize themselves with the Schwarzschild de Sitter solution in double null gauge. We discuss in particular the gauge freedom in the class of spherically symmetric foliations,^38^ from which the reader can see that our assumptions do not single out a specific choice of double null coordinates.

### General properties

The Schwarzschild de Sitter spacetime is a *spherically symmetric* solution to (1.1), and distinguishes itself from de Sitter solution by the presence of a mass $m>0$. The manifold is ${\mathcal {Q}}\times {\mathbb {S}}^2$, and the metric *g* takes the form (1.2). Moreover — as we have seen in Section 1.2 — in double null coordinates the metric takes the form (1.22), which simply reduces to

3.1 $$\begin{aligned} g=-4\Omega ^2\mathrm {d}u\mathrm {d}v +r^2{\mathop {\gamma }\limits ^{\circ }}\end{aligned}$$

The mass *m*, representing the “mass energy contained in a sphere” $S_{u,v}$, can be defined unambiguously in spherical symmetry as a function $m:{\mathcal {Q}}\rightarrow [0,\infty )$ satisfying

3.2 $$\begin{aligned} 1-\frac{2m(u,v)}{r}-\frac{\Lambda r^2}{3}=-\frac{1}{\Omega ^2}\frac{\partial r}{\partial u}\frac{\partial r}{\partial v} \end{aligned}$$

In *vacuum*, the Einstein equations (1.1) then imply that *m* is a constant, (which parametrizes this 1-parameter family of solutions.) This allows us further to pass from the unknown $r:{\mathcal {Q}}\rightarrow (0,\infty )$ to the “Regge-Wheeler coordinate”

3.3 $$\begin{aligned} {r_*}=\int \frac{\mathrm {d}r}{1-\frac{2m}{r}-\frac{\Lambda r^2}{3}} \end{aligned}$$

which by virtue of (1.1) satisfies the simple p.d.e.

3.4 $$\begin{aligned} \frac{\partial ^2 {r_*}}{\partial u \partial v}=0 \end{aligned}$$

The various double null coordinates discussed below can be thought of as different choices of functions *f*, *g* appearing in the general solution ${r_*}(u,v)=f(u)+g(v)$ of (3.4), and constants of integration in (3.3).

Let us also note that the polynomial in *r* on the l.h.s. of (3.2) has three real distinct roots $\overline{r_{{\mathcal {C}}}},{r_{{\mathcal {H}}}},{r_{{\mathcal {C}}}}$ provided $0<m<1/(3\sqrt{\Lambda })$, the two positive ones ${r_{{\mathcal {H}}}}$ and ${r_{{\mathcal {C}}}}$ coinciding with the event, and cosmological horizons ${\mathcal {H}}$, and ${\mathcal {C}}$, respectively, (where $\partial _u r=0$, or $\partial _vr=0$, by the equation). In the following we are only interested in charts covering the cosmological region, and horizons, namely the domain $r\ge {r_{{\mathcal {C}}}}$.

### Eddington-Finkelstein gauge

In “Eddington-Finkelstein” coordinates we choose

3.5 $$\begin{aligned} {r_*}=u+v=-\int _r^{\infty }\frac{1}{\frac{\Lambda r^2}{3}+\frac{2m}{r}-1}\mathrm {d}r \end{aligned}$$

and thus cover ${\mathcal {R}}^+$ by

3.6 $$\begin{aligned} {\mathcal {R}}^+=I^+({\mathcal {C}}^+\cup \overline{{\mathcal {C}}}^+) = \Bigl \{ (u,v,\vartheta ^1,\vartheta ^2) : u+v < 0 \Bigr \}\,. \end{aligned}$$

Note that the cosmological horizons ${\mathcal {C}}^+\cup \overline{{\mathcal {C}}}^+$ at ${r_*}=-\infty$, are *not* covered by this chart, but strictly only its future; moreover future null infinity ${\mathcal {I}}^+$ can be identified with the surface $u+v=0$. In these coordinates the metric takes the form

3.7 $$\begin{aligned} g=-4\Bigl (\frac{\Lambda r^2}{3}+\frac{2m}{r}-1\Bigr )\mathrm {d}u\mathrm {d}v+r^2{\mathop {\gamma }\limits ^{\circ }}_{AB}\mathrm {d}\vartheta ^A\vartheta ^B \end{aligned}$$

and we note specifically

3.8 $$\begin{aligned} \frac{\partial r}{\partial u}=\frac{\partial r}{\partial v}=\Omega ^2=\frac{\Lambda }{3}r^2+\frac{2m}{r}-1 \end{aligned}$$

With the definitions of the null normals $(\hat{{\underline{L}}},\hat{{L}})$ of Section 1.2 it is then straight-forward to verify that^39^

3.9a $$\begin{aligned} \omega= & {} \frac{\Lambda }{3}r-\frac{1}{2}\frac{2m}{r^2} \end{aligned}$$

3.9b $$\begin{aligned} {\hat{\omega }}= & {} \frac{1}{\Omega }\partial _v\log \Omega =\frac{1}{2}\frac{1}{\Omega }\Bigl (2\frac{\Lambda }{3}r-\frac{2m}{r^2}\Bigr )\longrightarrow \sqrt{\frac{\Lambda }{3}}\quad (r\rightarrow \infty ) \end{aligned}$$

3.9c $$\begin{aligned} {\hat{{\underline{\omega }}}}= & {} {\hat{\omega }}\end{aligned}$$

and

3.10a $$\begin{aligned} \chi _{AB}= & {} g(\nabla _{e_A}\hat{{L}},e_B)=\frac{\Omega }{r}g\!\!\!/_{AB} \end{aligned}$$

3.10b $$\begin{aligned} {\hat{\chi }}= & {} 0\qquad {{\,\mathrm{tr}\,}}\chi =\frac{2}{r}\Omega \longrightarrow 2\sqrt{\frac{\Lambda }{3}}\qquad (r\rightarrow \infty ) \end{aligned}$$

3.10c $$\begin{aligned} {\hat{{\underline{\chi }}}}= & {} 0\qquad {{\,\mathrm{tr}\,}}{\underline{\chi }}=\frac{2}{r}\Omega \end{aligned}$$

The Gauss equation (2.43) now allows us to calculate the $\rho$ component of the Weyl curvature: Since the spheres $S_{u,v}$ are *round*, we have $K=r^{-2}$, and we obtain with (3.10) that

3.11 $$\begin{aligned} \rho [W]=\frac{\Lambda }{3}-K-\frac{1}{4}{{\,\mathrm{tr}\,}}\chi {{\,\mathrm{tr}\,}}{\underline{\chi }}=\frac{1}{r^2}\Bigl (\frac{\Lambda }{3}r^2-1-\Omega ^2\Bigr )=-\frac{2m}{r^3}\,. \end{aligned}$$

(This shows in particular that the mass $m\ne 0$ is the obstruction to conformal flatness.) Moreover, since $\alpha [W]={\underline{\alpha }}[W]=\beta [W]={\underline{\beta }}[W]=\zeta [W]=0$ in spherical symmetry symmetry, and thus also $\sigma [W]=0$, we have also proven (1.8). In summary , $\rho [W]$, ${\hat{\omega }}$, ${\hat{{\underline{\omega }}}}$, and ${{\,\mathrm{tr}\,}}\chi$, ${{\,\mathrm{tr}\,}}{\underline{\chi }}$ are the only non-vanishing null structure components for the Schwarzschild de Sitter solution.

### Gauge transformations and “regular” coordinates

The choice of null coordinates in Section 3.2 has a shortcoming: the coordinates do not extend to the cosmological horizons. While Eddington-Finkelstein coordinates provide a natural notion of “retarded and advanced time”, we will now discuss coordinates which extend beyond the cosmological horizons. The following discussion highlights in particular the gauge dependence of the structure coefficients, and is relevant for the dynamical problem.

#### Kruskal coordinates

Let us denote the Eddington-Finkelstein coordinates of Section 3.2 by $({u_*},{v_*})$. Then “Kruskal coordinates” $(u_{{\mathcal {K}}},v_{{\mathcal {K}}})$ are obtained with the following transformation:

3.12 $$\begin{aligned} u_{{\mathcal {K}}}=e^{2\kappa _{\mathcal {C}}{u_*}}\qquad v_{{\mathcal {K}}}=e^{2\kappa _{\mathcal {C}}{v_*}} \end{aligned}$$

In these coordinates *r*(*u*, *v*) is implicitly given by

3.13 $$\begin{aligned} u_{{\mathcal {K}}}v_{{\mathcal {K}}}=(r-{r_{{\mathcal {C}}}})(r-{r_{{\mathcal {H}}}})^{-\alpha _{{\mathcal {H}}}}(r+|\overline{r_{{\mathcal {C}}}}|)^{-\overline{\alpha _{{\mathcal {C}}}}} \end{aligned}$$

where $\alpha _{{\mathcal {H}}},\overline{\alpha _{{\mathcal {C}}}}>0$ are positive exponents (depending on $\Lambda$, *m*) satisfying $\alpha _{{\mathcal {H}}}+\overline{\alpha _{{\mathcal {C}}}}=1$; cf. (3.16) in [38]. In particular, in these coordinates the cosmological horizons $\overline{{\mathcal {C}}}^+$, and ${\mathcal {C}}^+$, are at $u_{{\mathcal {K}}}=0$, and $v_{{\mathcal {K}}}=0$, respectively, and the future boundary $r=\infty$ lies on the *hyperbola* $u_{{\mathcal {K}}}v_{{\mathcal {K}}}=1$. The metric takes the form (3.1) where $\Omega$ is *non-degenerate* on the ${\mathcal {C}}\cup \overline{{\mathcal {C}}}$; in fact

3.14 $$\begin{aligned} \Omega ^2_{{\mathcal {K}}}=\frac{1}{4}\frac{\Lambda }{3}\kappa _{\mathcal {C}}^{-2}\frac{(r-{r_{{\mathcal {H}}}})^{1+\alpha _{{\mathcal {H}}}}(r+|\overline{r_{{\mathcal {C}}}}|)^{1+\overline{\alpha _{{\mathcal {C}}}}}}{r}\longrightarrow \frac{1}{4}\frac{\Lambda }{3}\kappa _{\mathcal {C}}^{-2}r^2\qquad (r\rightarrow \infty )\qquad \quad \end{aligned}$$

and

3.15 $$\begin{aligned} \frac{\partial r}{\partial u_{{\mathcal {K}}}}=2\kappa _{\mathcal {C}}\Omega ^2_{{\mathcal {K}}}v_{{\mathcal {K}}}\qquad \frac{\partial r}{\partial v_{{\mathcal {K}}}}=2\kappa _{\mathcal {C}}\Omega ^2_{{\mathcal {K}}}u_{{\mathcal {K}}}\end{aligned}$$

where $\kappa _{\mathcal {C}}>0$ is the surface gravity of the cosmological horizons; see Section 3 of [38] for derivations. It is then straight-forward to calculate that *in this gauge*,

3.16a $$\begin{aligned} \chi _{AB}= & {} g(\nabla _{e_A}\hat{{L}},e_B)=\frac{1}{\Omega _{{\mathcal {K}}}}\frac{1}{r}\partial _{v_{{\mathcal {K}}}} r\, g_{AB}=\frac{2}{r}\kappa _{\mathcal {C}}\Omega _{{\mathcal {K}}} u_{{\mathcal {K}}}\, g_{AB}\,, \end{aligned}$$

3.16b $$\begin{aligned} {\hat{\chi }}= & {} 0\qquad {{\,\mathrm{tr}\,}}\chi =4\kappa _{\mathcal {C}}\frac{\Omega _{{\mathcal {K}}} u_{{\mathcal {K}}}}{r} \end{aligned}$$

3.16c $$\begin{aligned} {{\,\mathrm{tr}\,}}\chi= & {} 0 \quad \text {on } u_{{\mathcal {K}}}=0\,,\quad {{\,\mathrm{tr}\,}}\chi \rightarrow 2\sqrt{\frac{\Lambda }{3}}u_{{\mathcal {K}}}\qquad \text { as } r\rightarrow \infty \,. \end{aligned}$$

Similarly,

3.17a $$\begin{aligned} {\hat{{\underline{\chi }}}}= & {} 0\qquad {{\,\mathrm{tr}\,}}{\underline{\chi }}=4\kappa _{\mathcal {C}}\frac{\Omega _{{\mathcal {K}}}v_{{\mathcal {K}}}}{r} \end{aligned}$$

3.17b $$\begin{aligned} {{\,\mathrm{tr}\,}}{\underline{\chi }}= & {} 0 \quad \text {on } v_{{\mathcal {K}}}=0\,,\qquad {{\,\mathrm{tr}\,}}{\underline{\chi }}\rightarrow 2\sqrt{\frac{\Lambda }{3}}v_{{\mathcal {K}}}\quad \text { as } r\rightarrow \infty \,. \end{aligned}$$

We also calculate

3.18a $$\begin{aligned} {\hat{\omega }}=\frac{1}{\Omega _{{\mathcal {K}}}}\frac{\partial }{\partial v_{{\mathcal {K}}}}\log \Omega _{{\mathcal {K}}}=\frac{1}{4\kappa _{\mathcal {C}}\Omega _{{\mathcal {C}}} v_{{\mathcal {K}}}}\Bigl [2\frac{\Lambda }{3}r-\frac{2m}{r^2}-2\kappa _{\mathcal {C}}\Bigr ] \!\longrightarrow \!\frac{1}{v_{{\mathcal {K}}}}\sqrt{\frac{\Lambda }{3}}\quad \text {as } r\!\rightarrow \!\infty \nonumber \\ \end{aligned}$$

and ${\hat{\omega }}=0$ on $u_{{\mathcal {K}}}=0$. Similarly,

3.18b $$\begin{aligned} {\hat{{\underline{\omega }}}}=\frac{1}{4\kappa _{\mathcal {C}}\Omega _{{\mathcal {K}}} u_{{\mathcal {K}}}}\Bigl [2\frac{\Lambda }{3}r-\frac{2m}{r^2}-2\kappa _{\mathcal {C}}\Bigr ]\rightarrow \frac{1}{u_{{\mathcal {K}}}}\sqrt{\frac{\Lambda }{3}}\qquad \text {as } r\rightarrow \infty \end{aligned}$$

#### “Initial data” gauge

We give an example of a double null system which retains “retarded time” *u* of “Eddington-Finkelstein type” along ${\mathcal {C}}^+$, and “advanced time” *v* of “Eddington-Finkelstein type” along $\overline{{\mathcal {C}}}^+$, yet is regular at the past horizons. It is trivially obtained by “patching” the above coordinate systems, but its features are worth studying, because it mimics a suitable gauge choice for the characteristic initial value problem.

Let us define

3.19 $$\begin{aligned} {\underline{u}}= {\left\{ \begin{array}{ll} u_{{\mathcal {K}}}-1 &{} {u_*}<0\\ 2\kappa _{\mathcal {C}}{u_*}&{}{u_*}>0 \end{array}\right. } \qquad {\underline{v}}= {\left\{ \begin{array}{ll} v_{{\mathcal {K}}}-1 &{} {v_*}<0\\ 2\kappa _{\mathcal {C}}{v_*}&{}{v_*}>0 \end{array}\right. } \end{aligned}$$

Then

3.20 $$\begin{aligned} {\mathop {g}\limits ^{{\mathcal {Q}}}}= {\left\{ \begin{array}{ll} -4\Omega ^2_{{\mathcal {K}}}\mathrm {d}{\underline{u}}\mathrm {d}{\underline{v}}&{} :{u_*}<0,{v_*}<0\\ -4\Omega ^2_{{\mathcal {K}}}u_{{\mathcal {K}}}\mathrm {d}{\underline{u}}\mathrm {d}{\underline{v}}=:-4\Omega _{(u)}^2\mathrm {d}{\underline{u}}\mathrm {d}{\underline{v}}&{}:{u_*}>0,{v_*}<0\\ -4\Omega ^2_{{\mathcal {K}}}v_{{\mathcal {K}}}\mathrm {d}{\underline{u}}\mathrm {d}{\underline{v}}=:-4\Omega _{(v)}^2\mathrm {d}{\underline{u}}\mathrm {d}{\underline{v}}&{}:{v_*}>0,{u_*}<0\\ \end{array}\right. } \end{aligned}$$

where

3.21a $$\begin{aligned} \Omega _{(u)}^2= & {} \Omega _{{\mathcal {K}}}^2u_{{\mathcal {K}}}=\frac{1}{4\kappa _{\mathcal {C}}^2}\frac{1}{v}\Bigl [\frac{\Lambda r^2}{3}+\frac{2m}{r}-1\Bigr ] \end{aligned}$$

3.21b $$\begin{aligned} \Omega _{(v)}^2= & {} \Omega ^2_{{\mathcal {K}}}v_{{\mathcal {K}}}=\frac{1}{4\kappa _{\mathcal {C}}^2}\frac{1}{u}\Bigl [\frac{\Lambda r^2}{3}+\frac{2m}{r}-1\Bigr ] \end{aligned}$$

This means that *in this gauge*, along ${\mathcal {C}}^+$, for ${u_*}>0$, the null lapse behaves like

3.22 $$\begin{aligned} \Omega |_{{\mathcal {C}}^+}=\frac{1}{2\kappa _{\mathcal {C}}}\cdot c(\Lambda ,m)\cdot e^{\kappa _{\mathcal {C}}{u_*}} \end{aligned}$$

and along the null infinity, for ${u_*}>0$,

3.23 $$\begin{aligned} \Omega ^2|_{{\mathcal {I}}^+}=\frac{1}{4\kappa _{\mathcal {C}}^2}\cdot e^{2\kappa _{\mathcal {C}}{u_*}}\cdot \frac{\Lambda }{3}r^2 \end{aligned}$$

Let us calculate the structure coefficients in the region ${u_*}>0$; (the region ${v_*}>0$ is entirely analogous). In the same way as in Section 3.2, we find, relative to the normalised frame,

3.24 $$\begin{aligned} \hat{{\underline{L}}}=\frac{1}{\Omega _{(u)}}\frac{\partial }{\partial {\underline{u}}}\qquad \hat{{L}}= \frac{1}{\Omega _{(u)}}\frac{\partial }{\partial {\underline{v}}} \end{aligned}$$

that

3.25a $$\begin{aligned} \begin{aligned} {\underline{\chi }}_{AB}&=\frac{1}{\Omega _{(u)}}\frac{1}{r}\partial _{{\underline{u}}}r\, g_{AB}=\frac{1}{\Omega _{(u)}}\frac{1}{r}\frac{1}{2\kappa _{\mathcal {C}}}\Omega _*^2\,g_{AB}\\&=\Omega _{(u)}\frac{2\kappa _{\mathcal {C}}}{r}v_{{\mathcal {K}}}\,g_{AB}=\frac{1}{\Omega _{(u)}}\frac{1}{2\kappa _{\mathcal {C}}}\frac{1}{r}\Bigl (\frac{\Lambda }{3}r^2+\frac{2m}{r}-1\Bigr )g_{AB} \end{aligned} \end{aligned}$$

or

3.25b $$\begin{aligned} {\hat{{\underline{\chi }}}}=0\qquad {{\,\mathrm{tr}\,}}{\underline{\chi }}=\frac{1}{\Omega _{(u)}}\frac{1}{\kappa _{\mathcal {C}}r}\Bigl (\frac{\Lambda }{3}r^2+\frac{2m}{r}-1\Bigr ) \end{aligned}$$

so that

3.25c $$\begin{aligned}&{{\,\mathrm{tr}\,}}{\underline{\chi }}|_{{\mathcal {C}}^+}=0 \end{aligned}$$

3.25d $$\begin{aligned}&{{\,\mathrm{tr}\,}}{\underline{\chi }}\longrightarrow 2\cdot e^{-\kappa _{\mathcal {C}}{u_*}}\cdot \sqrt{\frac{\Lambda }{3}}\qquad (r\rightarrow \infty ) \end{aligned}$$

Similarly, we find

3.26a $$\begin{aligned} \chi _{AB}= & {} \frac{1}{\Omega _{(u)}}\frac{1}{r}\partial _{v_{{\mathcal {K}}}}r g_{AB}=\frac{2\kappa _{\mathcal {C}}}{r}\Omega _{(u)} g_{AB} \end{aligned}$$

3.26b $$\begin{aligned} {\hat{\chi }}= & {} 0\qquad {{\,\mathrm{tr}\,}}\chi =\frac{4\kappa _{\mathcal {C}}}{r}\Omega _{(u)} \end{aligned}$$

in particular

3.26c $$\begin{aligned}&{{\,\mathrm{tr}\,}}\chi |_{{\mathcal {C}}^+}=c(\Lambda ,m)\cdot e^{\kappa _{\mathcal {C}}{u_*}} \end{aligned}$$

3.26d $$\begin{aligned}&{{\,\mathrm{tr}\,}}\chi \longrightarrow 2\sqrt{\frac{\Lambda }{3}}\cdot e^{\kappa _{\mathcal {C}}{u_*}}\qquad (r\rightarrow \infty ) \end{aligned}$$

It remains to calculate the values of ${\hat{\omega }}$, ${\hat{{\underline{\omega }}}}$ in this gauge. We find

3.27a $$\begin{aligned} {\hat{{\underline{\omega }}}}= & {} \frac{1}{2}\frac{1}{\Omega _{(u)}^3}\partial _{{\underline{u}}}\Omega _{(u)}^2=\frac{1}{4\kappa _{\mathcal {C}}\Omega _{(u)}}\Bigl (2\frac{\Lambda }{3}r-\frac{2m}{r^2}\Bigr ) \end{aligned}$$

3.27b $$\begin{aligned} {\hat{\omega }}= & {} \frac{1}{2}\frac{1}{\Omega _{(u)}^3}\frac{\partial \Omega ^2_{{\mathcal {K}}}}{\partial v_{{\mathcal {K}}}}u_{{\mathcal {K}}}=\kappa _{\mathcal {C}}\Omega _{(u)}\Bigl (\frac{1+\alpha _{{\mathcal {H}}}}{r-{r_{{\mathcal {H}}}}}+\frac{1+\overline{\alpha _{{\mathcal {C}}}}}{r+|\overline{r_{{\mathcal {C}}}}|}-\frac{1}{r}\Bigr ) \end{aligned}$$

and in particular

3.28a $$\begin{aligned} {\hat{{\underline{\omega }}}}|_{{\mathcal {C}}^+}= & {} c(\Lambda ,m)e^{-\kappa _{\mathcal {C}}{u_*}}\,,\qquad {\hat{{\underline{\omega }}}}\longrightarrow \sqrt{\frac{\Lambda }{3}}\cdot e^{-\kappa {u_*}}\quad (r\rightarrow \infty ) \end{aligned}$$

3.28b $$\begin{aligned} {\hat{\omega }}|_{{\mathcal {C}}^+}= & {} c(\Lambda ,m)\cdot e^{\kappa _{\mathcal {C}}{u_*}}\,,\qquad {\hat{\omega }}\longrightarrow \sqrt{\frac{\Lambda }{3}}\cdot e^{\kappa _{\mathcal {C}}{u_*}}\quad (r\rightarrow \infty ) \end{aligned}$$

#### Gauge invariance

In view of the assumptions on the structure coefficients outlined in Section 1.2, we discuss the gauge -dependence and -invariance of the relevant quantities for the Schwarzschild-de Sitter example.

In Table 1 we summarize the asymptotics towards null infinity of the values of the connection coefficients for the Schwarzschild de Sitter metric in the gauges discussed above.

**Table 1:** Schwarzschild de Sitter values in Eddington-Finkelstein, Kruskal and “Initial data” gauges, asymptotically towards null infinity

Gauge	*Edd.-Finkelstein*	*Kruskal*	“*Initial data*”
$\Omega ^2$	$\frac{\Lambda }{3}r^2$	$\frac{1}{4}\frac{\Lambda }{3}\frac{1}{\kappa _{\mathcal {C}}^2} r^2$	$\frac{1}{4}\frac{\Lambda }{3}\frac{1}{\kappa _{\mathcal {C}}^2} e^{2\kappa _{\mathcal {C}}{u_*}} r^2$
${{\,\mathrm{tr}\,}}\chi$	$2\sqrt{\frac{\Lambda }{3}}$	$2\sqrt{\frac{\Lambda }{3}}e^{2\kappa _{\mathcal {C}}{u_*}}$	$2\sqrt{\frac{\Lambda }{3}}e^{\kappa _{\mathcal {C}}{u_*}}$
${{\,\mathrm{tr}\,}}{\underline{\chi }}$	$2\sqrt{\frac{\Lambda }{3}}$	$2\sqrt{\frac{\Lambda }{3}}e^{2\kappa _{\mathcal {C}}{v_*}}$	$2\sqrt{\frac{\Lambda }{3}}e^{-\kappa _{\mathcal {C}}{u_*}}$
${\hat{\omega }}$	$\sqrt{\frac{\Lambda }{3}}$	$\sqrt{\frac{\Lambda }{3}}e^{-2\kappa {v_*}}$	$\sqrt{\frac{\Lambda }{3}}e^{\kappa _{\mathcal {C}}{u_*}}$
${\hat{{\underline{\omega }}}}$	$\sqrt{\frac{\Lambda }{3}}$	$\sqrt{\frac{\Lambda }{3}}e^{-2\kappa _{\mathcal {C}}{u_*}}$	$\sqrt{\frac{\Lambda }{3}}e^{-\kappa _{\mathcal {C}}{u_*}}$
*q*	1	$e^{2\kappa _{\mathcal {C}}{u_*}}$	$e^{\kappa _{\mathcal {C}}{u_*}}$

Note that each quantity, $\Omega$, ${\hat{\omega }}$, ${\hat{{\underline{\omega }}}}$, ${{\,\mathrm{tr}\,}}\chi$, ${{\,\mathrm{tr}\,}}{\underline{\chi }}$, has the same asymptotics in *r* (*towards* null infinity) in all gauges, but different behavior in ${u_*}$ *along* null infinity. In particular note that $\Omega$ differs by a prefactor even at the leading order.

Nonetheless — since $\lim _{r\rightarrow \infty } ({u_*}+{v_*})|_{\Sigma _r}=0$ — we have in all gauges

3.29 $$\begin{aligned} \lim _{r\rightarrow \infty }\frac{1}{4}{{\,\mathrm{tr}\,}}\chi {{\,\mathrm{tr}\,}}{\underline{\chi }}=\frac{\Lambda }{3}\end{aligned}$$

in agreement with the Gauss equation (2.43). Moreover, we have in all gauges,

3.30 $$\begin{aligned} \lim _{r\rightarrow \infty } |2{\hat{\omega }}-{{\,\mathrm{tr}\,}}\chi |= 0 \qquad \lim _{r\rightarrow \infty } |2{\hat{{\underline{\omega }}}}-{{\,\mathrm{tr}\,}}{\underline{\chi }}|= 0 \,. \end{aligned}$$

and

3.31 $$\begin{aligned} \lim _{r\rightarrow \infty } |q {{\,\mathrm{tr}\,}}{\underline{\chi }}- q^{-1}{{\,\mathrm{tr}\,}}\chi |=0 \end{aligned}$$

In fact, we have here in all gauges

3.32 $$\begin{aligned} \lim _{r\rightarrow \infty } q {{\,\mathrm{tr}\,}}{\underline{\chi }}= \lim _{r\rightarrow \infty } q^{-1}{{\,\mathrm{tr}\,}}\chi =2\sqrt{\frac{\Lambda }{3}}\,. \end{aligned}$$

## Global redshift effect

This Section contains a central part of this paper: We will prove a non-trivial bound for the Weyl curvature in spacetimes that satisfy our assumptions. This is achieved by means of energy estimates for the Bianchi equations, which are recalled in some generality in Section 4.1. In Section 4.2 we will construct a suitable “multiplier vectorfield” whose associated energy is “redshifted”, or “damped” in a fashion that is related to the expansion of the spacetime. This approach will then be further developed in Section 5 to obtain also bounds on the derivatives of the Weyl curvature.

### Energy identity

Let us recall that the conformal curvature tensor *W* satisfies the contracted Bianchi equations (1.27). The Bel-Robinson tensor *Q*(*W*) defined by (1.28) is symmetric and trace-free *in all indices*; cf. Proposition 12.5 in [8]. Moreover it is non-negative when evaluated on future-directed causal vectors.

We have — by Proposition 12.6 in [8], as a consequence of (1.27) — that *Q*(*W*) is divergence-free, see (1.29). In particular, if we define the energy current *P* associated to *W* as in (1.30) then it follows from (1.29) that

4.1 $$\begin{aligned} \nabla ^\alpha P[W]_\alpha ^{(X,Y,Z)}= & {} -\frac{1}{2}Q[W]_{\alpha \beta \gamma \delta }{}^{(X)}\pi ^{\alpha \beta } Y^\gamma Z^\delta -\frac{1}{2}Q[W]_{\alpha \beta \gamma \delta } X^\beta {}^{(Y)}\pi ^{\alpha \gamma } Z^\delta \nonumber \\&-\frac{1}{2}Q[W]_{\alpha \beta \gamma \delta }X^\beta Y^\gamma {}^{(Z)}\pi ^{\alpha \delta } \end{aligned}$$

In view of the trace-free property of *Q*[*W*] it is actually only the trace-free part of the deformation tensor that enters here:

4.2 $$\begin{aligned}&{}^{(X)}{\hat{\pi }}={}^{(X)}\pi -\frac{1}{4}({{\,\mathrm{tr}\,}}{}^{(X)}\pi )\,g \end{aligned}$$

4.3 $$\begin{aligned}&{}^{(X)}\pi ={\mathcal {L}}_X g \quad {}^{(X)}\pi _{\mu \nu }=\nabla _\mu X_\nu +\nabla _\nu X_\mu \end{aligned}$$

4.4 $$\begin{aligned} Q[W]_{\alpha \beta \gamma \delta }{}^{(X)}\pi ^{\alpha \beta } Y^\gamma&Z^\delta =Q[W]_{\alpha \beta \gamma \delta }{}^{(X)}{\hat{\pi }}^{\alpha \beta } Y^\gamma Z^\delta \end{aligned}$$

Using also the symmetry with respect to any index we finally obtain:

4.5 $$\begin{aligned} \nabla ^\alpha P[W]_\alpha ^{(X,Y,Z)}=-\frac{1}{2}Q[W]_{\alpha \beta \gamma \delta }\Bigl ({}^{(X)}{\hat{\pi }}^{\alpha \beta } Y^\gamma Z^\delta +{}^{(Y)}{\hat{\pi }}^{\alpha \beta } X^\gamma Z^\delta +{}^{(Z)}{\hat{\pi }}^{\alpha \beta } X^\gamma Y^\delta \Bigr )\nonumber \\ \end{aligned}$$

We defined *P* as a 1-form. Let ${}^*P$ be the dual of the corresponding vectorfield $P^\sharp$, which is a 3-form:

4.6 $$\begin{aligned} {}^*P_{\nu \kappa \lambda }=P^\mu \epsilon _{\mu \nu \kappa \lambda } \end{aligned}$$

Here $\epsilon =\mathrm {d}\mu _{g}$ is the volume form of *g*. The exterior derivative of ${}^*P$ is a 4-form, and hence must be proportional to the volume form:

4.7 $$\begin{aligned} \mathrm {d}{}^*P= f\mathrm {d}\mu _{g} \end{aligned}$$

Moreover,

4.8 $$\begin{aligned} f=-{}^*\mathrm {d}{}^*P=\nabla ^\mu P_\mu \end{aligned}$$

so we can conclude

4.9 $$\begin{aligned} \mathrm {d}{}^*P=\nabla ^\mu P_\mu \,\mathrm {d}\mu _{g}\,. \end{aligned}$$

This implies, that integrated on any spacetime region ${\mathcal {D}}$, we have by virtue of Stokes theorem,

4.10 $$\begin{aligned} \int _{\partial {\mathcal {D}}}{}^*P=\int _{{\mathcal {D}}}\mathrm {d}{}^*P=\int _{{\mathcal {D}}}\nabla ^\mu P_\mu \mathrm {d}\mu _{g} \end{aligned}$$

**Fig. 7:**
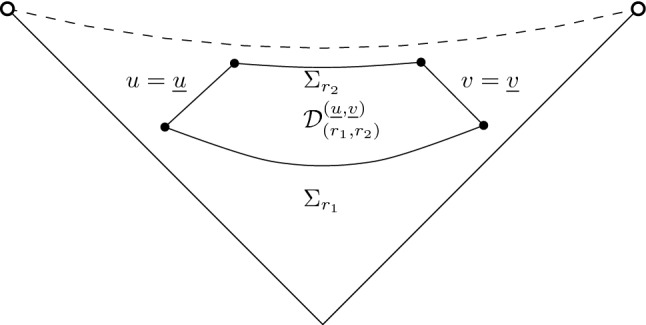
Spacetime domain used in energy identities

Let the domain ${\mathcal {D}}$ be as in Figure 7, namely

4.11 $$\begin{aligned} {\mathcal {D}}^{({\underline{u}},{\underline{v}})}_{(r_1,r_2)}=\bigcup _{r_1\le r \le r_2}\Sigma _r \bigcap \{u\le {\underline{u}}\} \bigcap \{v\le {\underline{v}}\} \end{aligned}$$

so that

4.12 $$\begin{aligned} \partial {\mathcal {D}}=\Sigma _{r_1}^c\cup C_{{\underline{u}}}^c \cup \Sigma _{r_2}^c \cup {\underline{C}}_{{\underline{v}}}^c \end{aligned}$$

where superscript *c* denotes that these surfaces are appropriately “capped”. We have

4.13 $$\begin{aligned} \int _{\Sigma _{r_2}^c} {}^*P=\int _{\Sigma _{r_2}^c} P^n\mathrm {d}\mu _{{\overline{g}}_{r_2}}=\int _{\Sigma _{r_2}^c} -g(P,n)\mathrm {d}\mu _{{\overline{g}}_{r_2}}=\int _{\Sigma _{r_2}^c} Q[W](n,X,Y,Z)\mathrm {d}\mu _{{\overline{g}}_{r_2}}\nonumber \\ \end{aligned}$$

where *n* is the unit normal to $\Sigma _r$; note that in the boundary integrals arising in Stokes theorem, the normal is always outward pointing, in particular it will have the opposite sign on the past boundary $\Sigma _{r_1}$ . For the null boundaries we recall first from (1.204-6) in [8] that

4.14a $$\begin{aligned} \epsilon= & {} \mathrm {d}\mu _{g}=2\Omega ^2\sqrt{\det g\!\!\!/}\,\mathrm {d}u\wedge \mathrm {d}v\wedge \mathrm {d}\vartheta ^1\wedge \mathrm {d}\vartheta ^2 \end{aligned}$$

4.14b $$\begin{aligned} \mathrm {d}\mu _{g\!\!\!/}= & {} \sqrt{\det g\!\!\!/}\mathrm {d}\vartheta ^1\wedge \mathrm {d}\vartheta ^2 \end{aligned}$$

and then calculate, using that $(u,\vartheta ^1,\vartheta ^2)$ are coordinates on ${\underline{C}}$,

4.15 $$\begin{aligned} \begin{aligned} \int _{{\underline{C}}_{{\underline{v}}}^c} {}^*P&= -\int {}^*P(\partial _u,\partial _{\vartheta ^1},\partial _{\vartheta ^2})\mathrm {d}u\mathrm {d}\vartheta ^1\mathrm {d}\vartheta ^2 \\&=-\int P^\mu \epsilon _{\mu u \vartheta ^1 \vartheta ^2} \mathrm {d}u\mathrm {d}\vartheta ^1\mathrm {d}\vartheta ^2=\int P^v \epsilon _{uv\vartheta ^1\vartheta ^2}\mathrm {d}u\mathrm {d}\vartheta ^1\mathrm {d}\vartheta ^2\\&= \int 2\Omega ^2 P^v \sqrt{\det g\!\!\!/}\mathrm {d}u\mathrm {d}\vartheta ^1\mathrm {d}\vartheta ^2=-\int g(P,\partial _u)\sqrt{\det g\!\!\!/}\mathrm {d}u\mathrm {d}\vartheta ^1\mathrm {d}\vartheta ^2\\&= \int Q[W](\partial _u,X,Y,Z) \mathrm {d}u\mathrm {d}\mu _{g\!\!\!/}=\int \Omega Q[W](\hat{{\underline{L}}},X,Y,Z) \mathrm {d}u\mathrm {d}\mu _{g\!\!\!/} \end{aligned}\qquad \end{aligned}$$

where we used that

4.16 $$\begin{aligned} P^\sharp= & {} P^u\partial _u+P^v\partial _v+P^A\partial _{\vartheta ^A} \end{aligned}$$

4.17 $$\begin{aligned} g(P,\partial _u)= & {} -2\Omega ^2 P^v\,. \end{aligned}$$

On the null hypersurface $C_u$ we have to be more careful because

4.18 $$\begin{aligned} g(P,\partial _v)=-2\Omega ^2 P^u+P^vg\!\!\!/_{AB}b^Ab^B-g\!\!\!/_{AB}P^A b^B \end{aligned}$$

However,

4.19 $$\begin{aligned} g(P,b^A\partial _{\vartheta ^A})=-P^v g\!\!\!/_{AB}b^A b^B+g\!\!\!/_{AB}P^Bb^A \end{aligned}$$

and so

4.20 $$\begin{aligned} g(P,\partial _v+b^A\partial _{\vartheta ^A})=-2\Omega ^2 P^u \end{aligned}$$

Therefore, similarly

4.21 $$\begin{aligned} \begin{aligned} \int _{C_u} {}^*P&= \int {}^*P_{v\vartheta ^1\vartheta ^2}\mathrm {d}v\mathrm {d}\vartheta ^2\vartheta ^2=\int 2\Omega ^2 P^u \sqrt{\det g\!\!\!/}\mathrm {d}v\mathrm {d}\vartheta ^1\vartheta ^2\\&=-\int g(P,\partial _v+b^A\partial _{\vartheta ^A})\mathrm {d}v\mathrm {d}\mu _{g\!\!\!/}=\int \Omega Q[W](\hat{{L}},X,Y,Z)\mathrm {d}v\mathrm {d}\mu _{g\!\!\!/} \end{aligned} \end{aligned}$$

To summarize we have proven the following:

#### Proposition 4.1

Let $({\mathcal {M}},g)$ be a spacetime, *g* be globally expressed in double null gauge on a domain ${\mathcal {R}}\subset {\mathcal {M}}$, such that the level sets of the area radius *r* are *spacelike* on ${\mathcal {R}}$. Moreover let ${\mathcal {D}}$ be a domain of the form (4.11); see Fig. 7. Then for any Weyl field *W* satisfying the Bianchi equations

4.22 $$\begin{aligned} \nabla ^\alpha W_{\alpha \beta \gamma \delta }=J_{\beta \gamma \delta } \end{aligned}$$

we have

4.23 $$\begin{aligned}&\int \mathrm {d}v\int _{S_{{\underline{u}},v}}\mathrm {d}\mu _{g\!\!\!/}\, \Omega Q[W](\hat{{L}},X,Y,Z)+\int _{\Sigma _{r_2}^c}\, Q[W](n,X,Y,Z)\mathrm {d}\mu _{{\overline{g}}_{r_2}}\nonumber \\&\quad +\,\int _{{\mathcal {D}}} \bigl ({{\,\mathrm{div}\,}}Q(W))(X,Y,Z) \mathrm {d}\mu _{g} +\int \mathrm {d}u\int _{S_{u,{\underline{v}}}}\,\mathrm {d}\mu _{g\!\!\!/} \Omega Q[W](\hat{{\underline{L}}},X,Y,Z) \nonumber \\&\quad +\,\int _{{\mathcal {D}}} K^{(X,Y,Z)}[W] \mathrm {d}\mu _{g}=\int _{\Sigma _{r_1}^c} Q[W](n,X,Y,Z)\mathrm {d}\mu _{{\overline{g}}_{r_1}} \end{aligned}$$

where *Q*[*W*] denotes the Bel-Robinson tensor of *W*, and

4.24 $$\begin{aligned} K^{(X,Y,Z)}[W]=\frac{1}{2}Q[W]_{\alpha \beta \gamma \delta }\Bigl ({}^{(X)}{\hat{\pi }}^{\alpha \beta } Y^\gamma Z^\delta +{}^{(Y)}{\hat{\pi }}^{\alpha \beta } X^\gamma Z^\delta +{}^{(Z)}{\hat{\pi }}^{\alpha \beta } X^\gamma Y^\delta \Bigr )\,.\nonumber \\ \end{aligned}$$

#### Proof

By (4.9),

4.25 $$\begin{aligned} \int _{\mathcal {D}} \nabla ^\mu P_\mu \mathrm {d}\mu _{g}=\int _{\partial {\mathcal {D}}}{}^*P=\int _{\Sigma _{r_2}^c}{}^*P +\int _{C_{{\underline{u}}}^c}{}^*P+\int _{{\underline{C}}_{{\underline{v}}}^c}{}^*P-\int _{\Sigma _{r_1}^c}{}^*P \end{aligned}$$

and we can insert the expressions (4.13) and (4.21) for the energy flux from above.

If $J=0$, or *W* is the *conformal* Weyl field, then by (4.5)

4.26 $$\begin{aligned} -\nabla ^\alpha P[W]_\alpha ^{(X,Y,Z)}=K^{(X,Y,Z)}[W]\, \end{aligned}$$

More generally, if $J\ne 0$, then the divergence contains the additional term

4.27 $$\begin{aligned} -\nabla ^\alpha P[W]_\alpha ^{(X,Y,Z)}=({{\,\mathrm{div}\,}}Q(W))(X,Y,Z)+K^{(X,Y,Z)}[W]\,. \end{aligned}$$

$$\square $$

### Global redshift vectorfield

We define

4.28 $$\begin{aligned} M=\frac{1}{2}\frac{1}{\Omega }\Bigl (\hat{{\underline{L}}}+\hat{{L}}\Bigr )\,. \end{aligned}$$

Note *M* is time-like future-directed, and the associated energy flux (4.13) is positive. Its crucial property however is that also the associated divergence (4.9) has a sign and bounds the energy flux, which lends it the name of a “redshift vectorfield”.

#### Remark 4.2

The choice (4.28) is motivated by the form of the “global redshift vectorfield” used in our treatment of linear waves on Schwarzschild de Sitter cosmologies in [38]. Therein we introduced

4.29 $$\begin{aligned} M=\frac{1}{r}\frac{\partial }{\partial r} \end{aligned}$$

relative to coordinates $(t,r,\vartheta ^1,\vartheta ^2)$ such that in the cosmological region the metric takes the form (1.24) with

4.30 $$\begin{aligned} \phi =\frac{1}{\sqrt{\frac{\Lambda r^2}{3}+\frac{2m}{r}-1}}\,, \end{aligned}$$

and

4.31 $$\begin{aligned} {\overline{g}}_{r}=\Bigl (\frac{\Lambda r^2}{3}+\frac{2m}{r}-1\Bigr )\mathrm {d}t^2+r^2{\mathop {\gamma }\limits ^{\circ }}_{AB}\mathrm {d}\vartheta ^A\mathrm {d}\vartheta ^B\,. \end{aligned}$$

Alternatively, using the gradient vectorfield *V* of *r* introduced in (2.48), this vectorfield can be expressed as

4.32 $$\begin{aligned} M=\frac{1}{r}\phi ^2 V\,, \end{aligned}$$

and in the coordinates introduced in Section 3.2,

4.33 $$\begin{aligned} V=\frac{1}{2}\Bigl (\frac{\partial }{\partial {u_*}}+\frac{\partial }{\partial {v_*}}\Bigr ) \end{aligned}$$

which implies

4.34 $$\begin{aligned} M=\frac{\phi ^2}{2r}\Bigl (\frac{\partial }{\partial {u_*}}+\frac{\partial }{\partial {v_*}}\Bigr ) =\frac{1}{2}\frac{1}{r}\frac{1}{\frac{\Lambda r^2}{3}+\frac{2m}{r}-1}\Bigl (\frac{\partial }{\partial {u_*}}+\frac{\partial }{\partial {v_*}}\Bigr )\,. \end{aligned}$$

In fact, as discussed in Section 4.1 of [38] it is equivalent to use the vectorfield

4.35 $$\begin{aligned} M^\prime =\frac{\partial }{\partial r} =\frac{1}{2}\frac{1}{\Omega ^2}\Bigl (\frac{\partial }{\partial {u_*}}+\frac{\partial }{\partial {v_*}}\Bigr )\,, \end{aligned}$$

which takes a remarkably simple form, and coincides precisely with (4.28).

#### Fluxes

We will derive an energy identity associated to the multiplier vectorfield *M* on a domain foliated by level sets of the area radius *r*. Let us first look at the energy flux of a current constructed from *M* through a surface $\Sigma _r$. Recall here Lemma 2.1 concerning the normal to $\Sigma _r$.

##### Lemma 4.3

Let *M* denote the vectorfield (4.28) and $P^M$ the current

4.36 $$\begin{aligned} P^M[W]=P[W]^{(M,M,M)}\,. \end{aligned}$$

Then

4.37 $$\begin{aligned} \int _{\Sigma _r} {}^*P^M= & {} \int _{\Sigma _r}\frac{1}{(2\Omega )^3}\Bigl \{q|{\underline{\alpha }}[W]|^2+2(3q+q^{-1})|{\underline{\beta }}[W] |^2\nonumber \\&\quad +\,6(q+q^{-1})\bigl (\rho [W]^2+\sigma [W]^2\bigr )+2(q+3q^{-1})|\beta [W]|^2\nonumber \\&\quad +\,q^{-1}|\alpha [W] |^2\Bigr \}\mathrm {d}\mu _{{\overline{g}}_{r}} \end{aligned}$$

and

4.38 $$\begin{aligned} \int _{C_u}{}^*P^M=\int \mathrm {d}v\int _{S_{u,v}}\mathrm {d}\mu _{g\!\!\!/}\frac{1}{4}\frac{1}{\Omega ^2}\Bigl [2|{\underline{\beta }}|^2+6\rho ^2+6\sigma ^2+6|\beta |^2+|\alpha |^2\Bigr ] \end{aligned}$$

##### Proof

This follows immediately from Lemma 12.2 in [8]. $\square$

#### Deformation Tensor

Next we calculate the components of the trace-free part of the deformation tensor of *M*, which enter the expression for $K^{(M,M,M)}$ in (4.24).

##### Lemma 4.4

The null components of the trace-free part ${\hat{\pi }}$ of the deformation tensor of *M* are given by

4.39a $$\begin{aligned} {\hat{\pi }}^{\hat{{\underline{L}}}\hat{{\underline{L}}}}= & {} \frac{{\hat{\omega }}}{\Omega }\quad {\hat{\pi }}^{\hat{{\underline{L}}}\hat{{L}}}=\frac{1}{8}\frac{1}{\Omega }\Bigl ({{\,\mathrm{tr}\,}}{\underline{\chi }}+{{\,\mathrm{tr}\,}}\chi \Bigr ) \quad {\hat{\pi }}^{\hat{{L}}\hat{{L}}}=\frac{{\hat{{\underline{\omega }}}}}{\Omega } \end{aligned}$$

4.39b $$\begin{aligned} {\hat{\pi }}^{\hat{{\underline{L}}}A}= & {} -\frac{1}{\Omega }{\underline{\eta }}^{\sharp A}\qquad {\hat{\pi }}^{\hat{{L}}A}=-\frac{1}{\Omega }\eta ^{\sharp A} \end{aligned}$$

4.39c $$\begin{aligned} {\hat{\pi }}^{AB}= & {} \frac{1}{\Omega }\Bigl ({\hat{{\underline{\chi }}}}^{\sharp \sharp AB}+{\hat{\chi }}^{\sharp \sharp AB}+\frac{1}{4}\bigl ({{\,\mathrm{tr}\,}}\chi +{{\,\mathrm{tr}\,}}{\underline{\chi }}\bigr )(g^{-1})^{AB}\Bigr ) \end{aligned}$$

##### Proof

In view of the frame relations (1.18) and (2.46) we have

4.40a $$\begin{aligned} \nabla _{\hat{{\underline{L}}}}M= & {} \frac{1}{\Omega }\Bigl (\eta ^\sharp -{\hat{{\underline{\omega }}}}\hat{{L}}\Bigr ) \end{aligned}$$

4.40b $$\begin{aligned} \nabla _{\hat{{L}}}M= & {} \frac{1}{\Omega }\Bigl ({\underline{\eta }}^\sharp -{\hat{\omega }}\hat{{\underline{L}}}\Bigr ) \end{aligned}$$

4.40c $$\begin{aligned} \nabla _AM= & {} \frac{1}{2}\frac{1}{\Omega }\Bigl ({\underline{\chi }}_A^\sharp -{\underline{\eta }}_A\hat{{\underline{L}}}+\chi _A^\sharp -\eta _A\hat{{L}}\Bigr ) \end{aligned}$$

and thus

4.41a $$\begin{aligned} \pi _{\hat{{\underline{L}}}\hat{{\underline{L}}}}= & {} 2g(\nabla _{\hat{{\underline{L}}}}M,\hat{{\underline{L}}})=\frac{4}{\Omega }{\hat{{\underline{\omega }}}}\end{aligned}$$

4.41b $$\begin{aligned} \pi _{\hat{{L}}\hat{{L}}}= & {} 2 g(\nabla _{\hat{{L}}}M,\hat{{L}})=\frac{4}{\Omega }{\hat{\omega }}\end{aligned}$$

4.41c $$\begin{aligned} \pi _{\hat{{\underline{L}}}\hat{{L}}}= & {} 0 \end{aligned}$$

4.41d $$\begin{aligned} \pi _{\hat{{\underline{L}}}e_A}= & {} g(\nabla _{\hat{{\underline{L}}}}M,e_A)+g(\hat{{\underline{L}}},\nabla _AM)=\frac{2}{\Omega }\eta _A \end{aligned}$$

4.41e $$\begin{aligned} \pi _{\hat{{L}}e_A}= & {} \frac{2}{\Omega }{\underline{\eta }}_A \end{aligned}$$

4.41f $$\begin{aligned} \pi _{e_Ae_B}= & {} g(\nabla _AM,e_A)+g(e_A,\nabla _BM)=\frac{1}{\Omega }\Bigl ({\underline{\chi }}_{AB}+\chi _{AB}\Bigr ) \end{aligned}$$

and

4.42 $$\begin{aligned} {{\,\mathrm{tr}\,}}\pi =\frac{1}{\Omega }\Bigl ({{\,\mathrm{tr}\,}}{\underline{\chi }}+{{\,\mathrm{tr}\,}}\chi \Bigr ) \end{aligned}$$

Therefore

4.43a $$\begin{aligned} {\hat{\pi }}_{\hat{{\underline{L}}}\hat{{\underline{L}}}}= & {} \frac{4}{\Omega }{\hat{{\underline{\omega }}}}\qquad {\hat{\pi }}_{\hat{{L}}\hat{{L}}}=\frac{4}{\Omega }{\hat{\omega }}\end{aligned}$$

4.43b $$\begin{aligned} {\hat{\pi }}_{\hat{{\underline{L}}}\hat{{L}}}= & {} \frac{1}{2}\frac{1}{\Omega }\Bigl ({{\,\mathrm{tr}\,}}{\underline{\chi }}+{{\,\mathrm{tr}\,}}\chi \Bigr ) \end{aligned}$$

4.43c $$\begin{aligned} {\hat{\pi }}_{\hat{{\underline{L}}}e_A}= & {} \frac{2}{\Omega }\eta _A\qquad {\hat{\pi }}_{\hat{{L}}e_A}=\frac{2}{\Omega }{\underline{\eta }}_A \end{aligned}$$

4.43d $$\begin{aligned} {\hat{\pi }}_{e_Ae_B}= & {} \frac{1}{\Omega }\Bigl ({\hat{{\underline{\chi }}}}_{AB}+{\hat{\chi }}_{AB}+\frac{1}{4}\bigl ({{\,\mathrm{tr}\,}}\chi +{{\,\mathrm{tr}\,}}{\underline{\chi }}\bigr )g_{AB}\Bigr ) \end{aligned}$$

or alternatively

4.44a $$\begin{aligned} {\hat{\pi }}^{\hat{{\underline{L}}}\hat{{\underline{L}}}}= & {} \frac{1}{4}{\hat{\pi }}_{\hat{{L}}\hat{{L}}}=\frac{{\hat{\omega }}}{\Omega }\qquad {\hat{\pi }}^{\hat{{L}}\hat{{L}}}=\frac{{\hat{{\underline{\omega }}}}}{\Omega } \end{aligned}$$

4.44b $$\begin{aligned} {\hat{\pi }}^{\hat{{\underline{L}}}\hat{{L}}}= & {} \frac{1}{8}\frac{1}{\Omega }\Bigl ({{\,\mathrm{tr}\,}}{\underline{\chi }}+{{\,\mathrm{tr}\,}}\chi \Bigr ) \end{aligned}$$

4.44c $$\begin{aligned} {\hat{\pi }}^{\hat{{\underline{L}}}A}= & {} -\frac{1}{\Omega }{\underline{\eta }}^{\sharp A}\qquad {\hat{\pi }}^{\hat{{L}}A}=-\frac{1}{\Omega }\eta ^{\sharp A} \end{aligned}$$

4.44d $$\begin{aligned} {\hat{\pi }}^{AB}= & {} \frac{1}{\Omega }\Bigl ({\hat{{\underline{\chi }}}}^{\sharp \sharp AB}+{\hat{\chi }}^{\sharp \sharp AB}+\frac{1}{4}\bigl ({{\,\mathrm{tr}\,}}\chi +{{\,\mathrm{tr}\,}}{\underline{\chi }}\bigr )(g\!\!\!/^{-1})^{AB}\Bigr )\,. \end{aligned}$$

$$\square $$

Now we can calculate

4.45 $$\begin{aligned} \begin{aligned} K^M&:=K^{(M,M,M)}[W]=\frac{3}{2}Q[W]_{\alpha \beta \gamma \delta }{}^{(M)}{\hat{\pi }}^{\alpha \beta } M^\gamma M^\delta \\&=\frac{3}{8}\frac{1}{\Omega ^2}Q[W]_{\alpha \beta \gamma \delta }{}^{(M)}{\hat{\pi }}^{\alpha \beta } \hat{{\underline{L}}}^\gamma \hat{{\underline{L}}}^\delta +\frac{3}{4}\frac{1}{\Omega ^2}Q[W]_{\alpha \beta \gamma \delta }{}^{(M)}{\hat{\pi }}^{\alpha \beta } \hat{{\underline{L}}}^\gamma \hat{{L}}^\delta \\&\qquad +\frac{3}{8}\frac{1}{\Omega ^2}Q[W]_{\alpha \beta \gamma \delta }{}^{(M)}{\hat{\pi }}^{\alpha \beta } \hat{{L}}^\gamma \hat{{L}}^\delta \end{aligned} \end{aligned}$$

which involves the null components of *Q*[*W*]. These are quadratic expressions in the Weyl curvature, which are given by Lemma 12.2 in [8]. In particular, we have

4.46a $$\begin{aligned} Q[W](\hat{{\underline{L}}},\hat{{\underline{L}}},\hat{{\underline{L}}},\hat{{\underline{L}}})= & {} 2|{\underline{\alpha }}[W]|^2\qquad \quad Q[W](\hat{{L}},\hat{{L}},\hat{{L}},\hat{{L}})=2|\alpha [W]|^2\qquad \quad \end{aligned}$$

4.46b $$\begin{aligned} Q[W](\hat{{L}},\hat{{\underline{L}}},\hat{{\underline{L}}},\hat{{\underline{L}}})= & {} 4|{\underline{\beta }}[W]|^2\qquad Q[W](\hat{{\underline{L}}},\hat{{L}},\hat{{L}},\hat{{L}})=4|\beta [W]|^2\qquad \quad \end{aligned}$$

4.46c $$\begin{aligned} Q(W)(\hat{{\underline{L}}},\hat{{\underline{L}}},\hat{{L}},\hat{{L}})= & {} 4\bigl (\rho [W]^2+\sigma [W]^2\bigr ) \end{aligned}$$

##### Lemma 4.5

With *M* defined by (4.28), we have

4.47 $$\begin{aligned} K^M[W]=K_+[W]+K_-[W] \end{aligned}$$

where

4.48 $$\begin{aligned} \frac{\Omega ^3}{3}K_+[W]= & {} \frac{1}{4}{\hat{\omega }}|{\underline{\alpha }}[W]|^2+{\hat{\omega }}|{\underline{\beta }}[W]|^2\nonumber \\&\quad +\,\frac{1}{2}({\hat{{\underline{\omega }}}}+{\hat{\omega }})(\rho [W]^2+\sigma [W]^2)+{\hat{{\underline{\omega }}}}|\beta [W]|^2\nonumber \\&\quad +\,\frac{1}{4}{\hat{{\underline{\omega }}}}|\alpha [W]|^2\nonumber \\&\quad +\,\frac{1}{2}\bigl ({{\,\mathrm{tr}\,}}\chi +{{\,\mathrm{tr}\,}}{\underline{\chi }}\bigr )\Bigl (\frac{1}{2}|{\underline{\beta }}[W]|^2+\rho [W]^2+\sigma [W]^2+\frac{1}{2}|\beta [W]|^2\Bigr )\nonumber \\ \end{aligned}$$

and

4.49 $$\begin{aligned} \frac{\Omega ^3}{3}K_-[W]= & {} {\underline{\alpha }}[W]({\underline{\eta }}^\sharp ,{\underline{\beta }}^\sharp [W])-\alpha [W](\eta ^\sharp ,\beta ^\sharp [W])\nonumber \\&\quad +\,\rho [W]\bigl ((2{\underline{\eta }}^\sharp +\eta ^\sharp )\cdot {\underline{\beta }}[W]-(2\eta +{\underline{\eta }}^\sharp )\cdot \beta [W]\bigr )\nonumber \\&\quad +\,\sigma [W]\bigl ((2{\underline{\eta }}^\sharp +\eta ^\sharp )\cdot {}^*{\underline{\beta }}[W]+(2\eta ^\sharp +{\underline{\eta }}^\sharp )\cdot {}^*\beta [W]\bigr )\nonumber \\&\quad -\,({\hat{{\underline{\chi }}}}+{\hat{\chi }})({\underline{\beta }}[W]^\sharp ,\beta [W]^\sharp )+\frac{1}{4}\rho [W]({\hat{{\underline{\chi }}}}+{\hat{\chi }},{\underline{\alpha }}[W]+\alpha [W])\nonumber \\&\quad +\,\frac{1}{4}\sigma [W]({\hat{{\underline{\chi }}}}+{\hat{\chi }},{}^*{\underline{\alpha }}[W]-{}^*\alpha [W]) \end{aligned}$$

##### Proof

Using the expressions for the null components of the Bel-Robinson tensor listed in Lemma 12.2 in [8] we note first that

4.50a $$\begin{aligned}&{\underline{\eta }}^{\sharp A}Q_{3A33}=-4{\underline{\alpha }}({\underline{\eta }}^\sharp ,{\underline{\beta }}^\sharp ) \end{aligned}$$

4.50b $$\begin{aligned}&\eta ^{\sharp A} Q_{4A33}=-4\rho \eta ^\sharp \cdot {\underline{\beta }}-4\sigma \eta ^\sharp \cdot {}^*{\underline{\beta }}\end{aligned}$$

4.50c $$\begin{aligned}&\bigl ({\hat{{\underline{\chi }}}}^{\sharp \sharp AB}+{\hat{\chi }}^{\sharp \sharp AB}\bigr ) Q_{AB33}=2\rho ({\hat{{\underline{\chi }}}}+{\hat{\chi }},{\underline{\alpha }})+2\sigma ({\hat{{\underline{\chi }}}}+{\hat{\chi }},{}^*{\underline{\alpha }}) \end{aligned}$$

4.50d $$\begin{aligned}&(g^{-1})^{AB} Q_{AB33}=4|{\underline{\beta }}|^2 \end{aligned}$$

and hence

4.51 $$\begin{aligned}&\Omega \,Q_{\alpha \beta \gamma \delta }\,{\hat{\pi }}^{\alpha \beta }\hat{{\underline{L}}}^\gamma \hat{{\underline{L}}}^\delta = 2{\hat{\omega }}|{\underline{\alpha }}|^2+4{\hat{{\underline{\omega }}}}(\rho ^2+\sigma ^2)+2({{\,\mathrm{tr}\,}}{\underline{\chi }}+{{\,\mathrm{tr}\,}}\chi )|{\underline{\beta }}|^2\nonumber \\&\quad +\,8{\underline{\alpha }}({\underline{\eta }}^\sharp ,{\underline{\beta }}^\sharp )+8\rho \eta ^\sharp \cdot {\underline{\beta }}+8\sigma \eta ^\sharp \cdot {}^*{\underline{\beta }}+2\rho ({\hat{{\underline{\chi }}}}+{\hat{\chi }},{\underline{\alpha }})+2\sigma ({\hat{{\underline{\chi }}}}+{\hat{\chi }},{}^*{\underline{\alpha }})\,.\nonumber \\ \end{aligned}$$

Similarly

4.52 $$\begin{aligned}&\Omega \,Q_{\alpha \beta \gamma \delta }\,{\hat{\pi }}^{\alpha \beta }\hat{{L}}^\gamma \hat{{L}}^\delta = 2{\hat{{\underline{\omega }}}}|\alpha |^2+4{\hat{\omega }}(\rho ^2+\sigma ^2) +2({{\,\mathrm{tr}\,}}{\underline{\chi }}+{{\,\mathrm{tr}\,}}\chi )|\beta |^2\nonumber \\&\quad -\,8\alpha (\eta ^\sharp ,\beta ^\sharp )-8\rho {\underline{\eta }}^\sharp \cdot \beta +8\sigma {\underline{\eta }}^\sharp \cdot {}^*\beta +2\rho ({\hat{{\underline{\chi }}}}+{\hat{\chi }},\alpha )-2\sigma ({\hat{{\underline{\chi }}}}+{\hat{\chi }},{}^*\alpha )\,.\nonumber \\ \end{aligned}$$

Moreover, by Lemma 12.2 in [8] we have

4.53a $$\begin{aligned}&{\underline{\eta }}^{\sharp A}Q_{3A34}=-4\rho {\underline{\eta }}^{\sharp }\cdot {\underline{\beta }}-4\sigma {\underline{\eta }}^\sharp \cdot {}^*{\underline{\beta }}\end{aligned}$$

4.53b $$\begin{aligned}&\eta ^{\sharp A} Q_{4A34}=4\rho \eta ^\sharp \cdot \beta -4\sigma \eta ^{\sharp A}\cdot {}^*\beta \end{aligned}$$

4.53c $$\begin{aligned}&\Bigl ({\hat{{\underline{\chi }}}}^{\sharp \sharp AB}+{\hat{\chi }}^{\sharp \sharp AB}\Bigr )Q_{AB34}=-2\Bigl ({\hat{{\underline{\chi }}}}+{\hat{\chi }},{\underline{\beta }}[W]{\hat{\otimes }}\beta [W]\Bigr ) \end{aligned}$$

4.53d $$\begin{aligned}&(g^{-1})^{AB}Q_{AB34}=4\Bigl (\rho ^2+\sigma ^2\Bigr ) \end{aligned}$$

where ${\underline{\beta }}{\hat{\otimes }}\beta$ denotes the symmetric trace-free 2-covariant tensorfield:

4.54 $$\begin{aligned} ({\underline{\beta }}{\hat{\otimes }}\beta )_{AB}={\underline{\beta }}_A\beta _B+{\underline{\beta }}_B\beta _A-({\underline{\beta }},\beta )g\!\!\!/_{AB}\,. \end{aligned}$$

In particular we can write on the right hand side of (4.53c)

4.55 $$\begin{aligned} \Bigl ({\hat{{\underline{\chi }}}}^{\sharp \sharp AB}+{\hat{\chi }}^{\sharp \sharp AB}\Bigr )\bigl ({\underline{\beta }}[W]{\hat{\otimes }}\beta [W]\bigr )_{AB}=2{\hat{{\underline{\chi }}}}({\underline{\beta }}[W]^\sharp ,\beta [W]^\sharp )+2{\hat{\chi }}({\underline{\beta }}[W]^\sharp ,\beta [W]^\sharp )\,.\nonumber \\ \end{aligned}$$

Therefore,

4.56 $$\begin{aligned}&\Omega Q_{\alpha \beta \gamma \delta }{\hat{\pi }}^{\alpha \beta }\hat{{\underline{L}}}^\gamma \hat{{L}}^\delta = 4{\hat{\omega }}|{\underline{\beta }}|^2+4{\hat{{\underline{\omega }}}}|\beta |^2+2\bigl ({{\,\mathrm{tr}\,}}\chi +{{\,\mathrm{tr}\,}}{\underline{\chi }}\bigr )\Bigl (\rho ^2+\sigma ^2\Bigr )\nonumber \\&\quad +\,8\rho [W]{\underline{\eta }}^{\sharp }\cdot {\underline{\beta }}+8\sigma {\underline{\eta }}^\sharp \cdot {}^*{\underline{\beta }}-8\rho \eta ^\sharp \cdot \beta +8\sigma \eta ^{\sharp A}\cdot {}^*\beta -4({\hat{{\underline{\chi }}}}+{\hat{\chi }})({\underline{\beta }}^\sharp ,\beta ^\sharp )\nonumber \\ \end{aligned}$$

Summing up these contributions according to (4.45) yields the statement of the Lemma. $\square$

##### Remark 4.6

We already see that $K_+[W]$ is manifestly positive if ${{\,\mathrm{tr}\,}}\chi >0$, ${{\,\mathrm{tr}\,}}{\underline{\chi }}>0$, and ${\hat{\omega }}>0$, ${\hat{{\underline{\omega }}}}>0$, as it will be the case under our assumptions. Indeed the assumption (${\varvec{BA:I}}.ii$) ensures that $2{\hat{\omega }}$ is close to ${{\,\mathrm{tr}\,}}\chi$, and $2{\hat{{\underline{\omega }}}}$ is close to ${{\,\mathrm{tr}\,}}{\underline{\chi }}$, the latter of which are positive by (${\varvec{BA:I}}.i$). In fact, the difference asymptotically tends to zero because $\Omega$ is comparable to *r* by (${\varvec{BA:III}}.i$).

#### Lorentz Transformations

We will see in Section 4.3 that the simple choice (4.28) for *M* suffices to obtain the desired energy estimate for the Weyl curvature *W*. However, it turns out in Section 5 that a more refined choice is necessary to obtain an estimates for higher order energies.

The required adjustment amounts to co-aligning the vectorfield with the normal to $\Sigma _r$. This can be achieved by formally keeping exactly the same definition of *M*, *but* changing the null frame that is used in (4.28). The fact that we can keep this simple definition in terms of another null frame will be computationally very advantageous.

A simple Lorentz transformation is given by:

4.57 $$\begin{aligned} \hat{{\underline{L}}}\mapsto a \hat{{\underline{L}}}\qquad \hat{{L}}\mapsto a^{-1}\hat{{L}}\end{aligned}$$

for some function $a>0$.

##### Remark 4.7

Let us give a heuristic discussion for the choice of *a* which aligns *M* with *n*. Regarding the normal, we have by Lemma 2.1, and taking $r\rightarrow \infty$,

4.58 $$\begin{aligned} \phi n=\frac{1}{r}\frac{\Omega }{\overline{\Omega {{\,\mathrm{tr}\,}}{\underline{\chi }}}}\hat{{\underline{L}}}+\frac{1}{r}\frac{\Omega }{\overline{\Omega {{\,\mathrm{tr}\,}}\chi }}\rightarrow \frac{1}{r}\Bigl (\frac{1}{{{\,\mathrm{tr}\,}}{\underline{\chi }}}\hat{{\underline{L}}}+\frac{1}{{{\,\mathrm{tr}\,}}\chi }\hat{{L}}\Bigr ) \end{aligned}$$

where we have neglected asymptotic deviations from spherical averages. Since by the Gauss equation (2.43),

4.59 $$\begin{aligned} \frac{1}{4}{{\,\mathrm{tr}\,}}\chi {{\,\mathrm{tr}\,}}{\underline{\chi }}\rightarrow \frac{\Lambda }{3}\end{aligned}$$

we can expect that for some function $a_\chi$, as $r\rightarrow \infty$,

4.60 $$\begin{aligned} {{\,\mathrm{tr}\,}}\chi \rightarrow 2\sqrt{\frac{\Lambda }{3}}a_\chi \qquad {{\,\mathrm{tr}\,}}{\underline{\chi }}\rightarrow 2\sqrt{\frac{\Lambda }{3}}a_\chi ^{-1} \end{aligned}$$

Now we see clearly that the function $a_\chi$ appearing in the asymptotics of ${{\,\mathrm{tr}\,}}\chi$, and ${{\,\mathrm{tr}\,}}{\underline{\chi }}$, is the required rescaling of the null vectors in (4.57). More precisely, with $a=a_\chi$, we can expect that *M*, formally given by (4.28) relative to a frame resulting from the Lorentz transformation (4.57), satifies asymptotically

4.61 $$\begin{aligned} M\rightarrow \phi n\,. \end{aligned}$$

For any function $a>0$, let us denote by

4.62 $$\begin{aligned} e_3=a\hat{{\underline{L}}}\qquad e_4=a^{-1}\hat{{L}}\end{aligned}$$

and $e_A:A=1,2$ an arbitrary frame on the spheres. Note that both $(\hat{{\underline{L}}},\hat{{L}};e_A)$ and $(e_3,e_4;e_A)$ are null frames. Here the frame $(\hat{{\underline{L}}},\hat{{L}};e_A)$ is the null frame derived from the double null coordiantes, and we continue to denote by ${\hat{{\underline{\omega }}}},{\hat{\omega }},\eta ,$ etc. the associated structure coefficients. Now for the frame $(e_3,e_4;e_A)$ we find the following connection coefficients:

4.63a $$\begin{aligned} \nabla _3e_3= & {} \bigl (a{\hat{{\underline{\omega }}}}+\hat{{\underline{L}}}a\Bigr )e_3\qquad \nabla _4 e_4=\bigl (a^{-1}{\hat{\omega }}+\hat{{L}}a^{-1}\Bigr )e_4 \end{aligned}$$

4.63b $$\begin{aligned} \nabla _3 e_4= & {} 2\eta ^\sharp -\bigl (a{\hat{{\underline{\omega }}}}-a^2\hat{{\underline{L}}}a^{-1}\bigr )e_4\qquad \nabla _4 e_3=2{\underline{\eta }}^\sharp -\bigl (a^{-1}{\hat{\omega }}-a^{-2}\hat{{L}}a\bigr )e_3\qquad \quad \end{aligned}$$

4.63c $$\begin{aligned} \nabla _A e_3= & {} a{\underline{\chi }}_A^{\sharp B}e_B+\bigl (\zeta _A+\mathrm {d}\!\!\!/_A\log a\bigr )e_3\nonumber \\ \nabla _A e_4= & {} a^{-1}\chi _A^{\sharp B}e_B-\bigl (\zeta _A+\mathrm {d}\!\!\!/_A\log a\bigr )e_4 \end{aligned}$$

4.63d $$\begin{aligned} \nabla _3 e_A= & {} \nabla \!\!\!\!/_3e_A +\eta _Ae_3\qquad \nabla _4e_A=\nabla \!\!\!\!/_4 e_A+{\underline{\eta }}_A e_4 \end{aligned}$$

4.63e $$\begin{aligned} \nabla _A e_B= & {} \nabla \!\!\!\!/_A e_B+\frac{1}{2}a^{-1}\chi _{AB}e_3+\frac{1}{2}a{\underline{\chi }}_{AB}e_4 \end{aligned}$$

Here we use the notation:

4.64 $$\begin{aligned} \nabla \!\!\!\!/_3e_A=\Pi \nabla _{\hat{{\underline{L}}}}e_A\qquad \nabla \!\!\!\!/_4e_A=\Pi \nabla _{\hat{{L}}}e_A \end{aligned}$$

where

4.65 $$\begin{aligned} \Pi X=X+\frac{1}{2}g(X,\hat{{\underline{L}}})\hat{{L}}+\frac{1}{2}g(X,\hat{{L}})\hat{{\underline{L}}}\end{aligned}$$

is the projection to subspace of the tangent space spanned by $e_A:A=1,2$, namely the tangent space of the spheres.

Also note:

4.66 $$\begin{aligned} e_3\log \Omega =a{\hat{{\underline{\omega }}}}\qquad e_4\log \Omega =a^{-1}{\hat{\omega }}\end{aligned}$$

In other words, the Lorentz transformation (4.57) induces the following transformations of the structure coefficients:

4.67a $$\begin{aligned}&{\underline{\chi }}\mapsto a{\underline{\chi }}\qquad \chi \mapsto a^{-1}\chi \end{aligned}$$

4.67b $$\begin{aligned}&\zeta \mapsto \zeta +\mathrm {d}\!\!\!/\log a\qquad \eta \mapsto \eta +\mathrm {d}\!\!\!/\log a\qquad {\underline{\eta }}\rightarrow {\underline{\eta }}-\mathrm {d}\!\!\!/\log a \end{aligned}$$

4.67c $$\begin{aligned}&{\hat{{\underline{\omega }}}}\mapsto a{\hat{{\underline{\omega }}}}+\frac{1}{\Omega }{\underline{D}}a\qquad {\hat{\omega }}\mapsto a^{-1}{\hat{\omega }}+\frac{1}{\Omega }D a^{-1} \end{aligned}$$

Recall here the notation ${\underline{D}}a={\underline{L}}a$, and $D a^{-1}=L a^{-1}$ introduced in Section 2.4.1.

##### Lemma 4.8

For any function $a=a(u,v,\vartheta ^1,\vartheta ^2)>0$, let $M_a$ denote the vectorfield

4.68 $$\begin{aligned} M_a=\frac{1}{2}\frac{1}{\Omega }\bigl (a\hat{{\underline{L}}}+a^{-1}\hat{{L}}\bigr )=\frac{1}{2}\frac{1}{\Omega }\bigl (e_3+e_4\bigr ) \end{aligned}$$

Then with respect to the null frame $(e_3,e_4;e_A)$ the null components of the deformation tensor of $M_a$ are:

4.69a $$\begin{aligned} {}^{(M_a)}{\hat{\pi }}_{33}= & {} \frac{4}{\Omega }a{\hat{{\underline{\omega }}}}+\frac{2}{\Omega }\hat{{\underline{L}}}a\qquad {}^{(M_a)}{\hat{\pi }}_{44}=\frac{4}{\Omega }a^{-1}{\hat{\omega }}+\frac{2}{\Omega }\hat{{L}}a^{-1} \end{aligned}$$

4.69b $$\begin{aligned} {}^{(M_a)}{\hat{\pi }}_{34}= & {} \frac{1}{2}\frac{1}{\Omega }\bigl (a{{\,\mathrm{tr}\,}}{\underline{\chi }}+a^{-1}{{\,\mathrm{tr}\,}}\chi \bigr )-\frac{1}{2}\frac{1}{\Omega }\bigl (\hat{{\underline{L}}}a+\hat{{L}}a^{-1}\bigr ) \end{aligned}$$

4.69c $$\begin{aligned} {}^{(M_a)}{\hat{\pi }}_{3A}= & {} \frac{1}{\Omega }\bigl (2\eta _A+\mathrm {d}\!\!\!/_A\log a\bigr )\qquad {}^{(M_a)}{\hat{\pi }}_{4A}=\frac{1}{\Omega }\bigl (2{\underline{\eta }}_A-\mathrm {d}\!\!\!/_A\log a\bigr ) \end{aligned}$$

4.69d $$\begin{aligned} {}^{(M_a)}{\hat{\pi }}_{AB}= & {} \frac{1}{\Omega }\Bigl (a{\hat{{\underline{\chi }}}}_{AB}+a^{-1}{\hat{\chi }}_{AB}+\frac{1}{4}\bigl (a{{\,\mathrm{tr}\,}}{\underline{\chi }}+a^{-1}{{\,\mathrm{tr}\,}}\chi -\hat{{\underline{L}}}a-\hat{{L}}a^{-1}\bigr )g\!\!\!/_{AB}\Bigr )\nonumber \\ \end{aligned}$$

where ${\underline{\chi }},\chi ,\eta ,{\underline{\eta }},\zeta ,{\hat{{\underline{\omega }}}},{\hat{\omega }}$ are the structure coefficients associated to the null frame $(\hat{{\underline{L}}},\hat{{L}};e_A)$.

##### Proof

Analogously to Lemma 4.4 we first compute

4.70a $$\begin{aligned} \nabla _3 M_a= & {} \frac{1}{\Omega }\bigl (\eta ^\sharp -a{\hat{{\underline{\omega }}}}e_4\bigr )+\frac{1}{2}\frac{1}{\Omega }(\hat{{\underline{L}}}a)\bigl (e_3-e_4\bigr ) \end{aligned}$$

4.70b $$\begin{aligned} \nabla _4M_a= & {} \frac{1}{\Omega }\bigl ({\underline{\eta }}^\sharp -a^{-1}{\hat{\omega }}e_3\bigr )+\frac{1}{2}\frac{1}{\Omega }(\hat{{L}}a^{-1})(e_4-e_3) \end{aligned}$$

4.70c $$\begin{aligned} \nabla _AM_a= & {} \frac{1}{2}\frac{1}{\Omega }\bigl (a{\underline{\chi }}^{\sharp B}_A+a^{-1}\chi ^{\sharp B}_A\bigr )e_B-\frac{1}{2}\frac{1}{\Omega }\bigl ({\underline{\eta }}_A-\mathrm {d}\!\!\!/_A\log a\bigr )e_3\nonumber \\&\quad -\,\frac{1}{2}\frac{1}{\Omega }\bigl (\eta _A+\mathrm {d}\!\!\!/_A\log a\bigr )e_4 \end{aligned}$$

using the frame relations (4.63) and (4.66), and then infer

4.71a $$\begin{aligned} \pi _{33}= & {} \frac{4}{\Omega }a{\hat{{\underline{\omega }}}}+\frac{2}{\Omega }\hat{{\underline{L}}}a\qquad \pi _{44}=\frac{4}{\Omega }a^{-1}{\hat{\omega }}+\frac{2}{\Omega }\hat{{L}}a^{-1} \end{aligned}$$

4.71b $$\begin{aligned} \pi _{34}= & {} -\frac{1}{\Omega }\hat{{\underline{L}}}a-\frac{1}{\Omega }\hat{{L}}a^{-1} \end{aligned}$$

4.71c $$\begin{aligned} \pi _{3A}= & {} \frac{1}{\Omega }\bigl (2\eta _A+\mathrm {d}\!\!\!/_A\log a\bigr )\qquad \pi _{4A}=\frac{1}{\Omega }\bigl (2{\underline{\eta }}_A-\mathrm {d}\!\!\!/_A\log a\bigr ) \end{aligned}$$

4.71d $$\begin{aligned} \pi _{AB}= & {} \frac{1}{\Omega }\bigl (a{\underline{\chi }}_{AB}+a^{-1}\chi _{AB}\bigr ) \end{aligned}$$

and

4.72 $$\begin{aligned} {{\,\mathrm{tr}\,}}\pi =\frac{1}{\Omega }\bigl (a{{\,\mathrm{tr}\,}}{\underline{\chi }}+a^{-1}{{\,\mathrm{tr}\,}}\chi +\hat{{\underline{L}}}a+\hat{{L}}a^{-1}\bigr ) \end{aligned}$$

which gives the formulas of the Lemma for the trace free part of $\pi$. $\square$

We continue to denote by $({\underline{\alpha }}[W],\alpha [W],{\underline{\beta }}[W],\beta [W],\rho [W],\sigma [W])$ the null components of *W* with respect to the null frame $(\hat{{\underline{L}}},\hat{{L}};e_A)$. To avoid confusion, we will explicitly denote the null components of *W* with respect to the null frame $(e_3,e_4;e_A)$ by $({\underline{\alpha }}_a[W],\alpha _a[W],{\underline{\beta }}_a[W],\beta _a[W],\rho _a[W],\sigma _a[W])$, and note that

4.73a $$\begin{aligned} {\underline{\alpha }}_a[W]= & {} a^2{\underline{\alpha }}[W]\qquad \alpha _a[W]=a^{-2}\alpha [W] \end{aligned}$$

4.73b $$\begin{aligned} {\underline{\beta }}_a[W]= & {} a{\underline{\beta }}[W]\qquad \beta _a[W]=a^{-1}\beta [W] \end{aligned}$$

4.73c $$\begin{aligned} \rho _a[W]= & {} \rho [W]\qquad \sigma _a[W]=\sigma [W] \end{aligned}$$

Note that if we take $a:=q$, where *q* is the quotient appearing in (2.52), then the normal takes the simple form

4.74 $$\begin{aligned} n=\frac{1}{2}\bigl (e_3+e_4\bigr ); \end{aligned}$$

this, of course, is the purpose of introducing the frame $(e_3,e_4;e_A)$ in the first place.

Moreover, it follows immediately from (4.13) with (4.68) and (4.74) that

4.75 $$\begin{aligned} \int _{\Sigma _r}{}^*P^{M_q}[W]= & {} \int _{\Sigma _r}Q[W](n,M_q,M_q,M_q)\mathrm {d}\mu _{{\overline{g}}_{r}}\nonumber \\= & {} \int _{\Sigma _r}\frac{1}{(2\Omega )^3}\Bigl [|{\underline{\alpha }}_q[W]|^2+8|{\underline{\beta }}_q[W]|^2\nonumber \\&\quad +\,12\rho [W]^2+12\sigma [W]^2+8|\beta _q[W]|^2\nonumber \\&\quad +\,|\alpha _q[W]|^2\Bigr ]\mathrm {d}\mu _{{\overline{g}}_{r}} \end{aligned}$$

and from (4.21) with (4.68) that

4.76 $$\begin{aligned} \int _{C_u}{}^*P^{M_q}[W]= & {} \int \mathrm {d}v\int _{S_{u,v}}\mathrm {d}\mu _{g\!\!\!/}\Omega Q[W](\hat{{L}},M_q,M_q,M_q)=\nonumber \\= & {} \int \mathrm {d}v\int _{S_{u,v}}\mathrm {d}\mu _{g\!\!\!/}\frac{q}{(2\Omega )^2}\Bigl [2|{\underline{\beta }}_q[W]|^2+6\rho [W]^2\nonumber \\&\quad +\,6\sigma [W]^2+6|\beta _q[W]|^2+|\alpha _q[W]|^2\Bigr ]\,. \end{aligned}$$

These fluxes should be compared with Lemma 4.3 where the corresponding fluxes associated to *M* were stated.

Let us prove the analogue of Lemma 4.5:

##### Lemma 4.9

Let $M_a$ be defined as in (4.68). Then

4.77 $$\begin{aligned} K^{M_a}[W]=K_+^a[W]+K_-^a[W] \end{aligned}$$

where

4.78 $$\begin{aligned} \frac{(2\Omega )^3}{3}K_+^a[W]= & {} \bigl (2a^{-1}{\hat{\omega }}+\hat{{L}}a^{-1}\bigr )a^4|{\underline{\alpha }}|^2+2(a{{\,\mathrm{tr}\,}}{\underline{\chi }}+a^{-1}{{\,\mathrm{tr}\,}}\chi \nonumber \\&+\,4a^{-1}{\hat{\omega }}-\hat{{\underline{L}}}a+\hat{{L}}a^{-1})a^2|{\underline{\beta }}|^2\nonumber \\&+\,\bigl (4a {\hat{{\underline{\omega }}}}+4a{{\,\mathrm{tr}\,}}{\underline{\chi }}-2\hat{{\underline{L}}}a+4a^{-1}{\hat{\omega }}+4a^{-1}{{\,\mathrm{tr}\,}}\chi -2\hat{{L}}a^{-1}\bigr )\nonumber \\&\times (\rho ^2+\sigma ^2) +\,2\bigl (a{{\,\mathrm{tr}\,}}{\underline{\chi }}+a^{-1}{{\,\mathrm{tr}\,}}\chi +4a{\hat{{\underline{\omega }}}}+\hat{{\underline{L}}}a-\hat{{L}}a^{-1}\bigr )a^{-2}|\beta |^2\nonumber \\&+\bigl (2a{\hat{{\underline{\omega }}}}+\hat{{\underline{L}}}a\bigr )a^{-4}|\alpha |^2 \end{aligned}$$

and

4.79 $$\begin{aligned} \frac{\Omega ^3}{3}K_-^a[W]= & {} a^3{\underline{\alpha }}(2{\underline{\eta }}^\sharp -\nabla \!\!\!\!/\log a,{\underline{\beta }}^\sharp )+a(\rho {\underline{\beta }}+\sigma {}^*{\underline{\beta }},2\eta +\nabla \!\!\!\!/\log a)\nonumber \\&\quad +\frac{1}{4}a^2(a{\hat{{\underline{\chi }}}}+a^{-1}{\hat{\chi }},\rho {\underline{\alpha }}+\sigma {}^*{\underline{\alpha }}) \nonumber \\&\quad +\,2 a(\rho {\underline{\beta }}+\sigma {}^*{\underline{\beta }},2{\underline{\eta }}-\mathrm {d}\!\!\!/\log a)-2a^{-1}(\rho \beta -\sigma {}^*\beta ,2\eta +\mathrm {d}\!\!\!/\log a)\nonumber \\&\quad -(a{\hat{{\underline{\chi }}}}+a^{-1}{\hat{\chi }})({\underline{\beta }}^\sharp ,\beta ^\sharp )\nonumber \\&\quad -\,a^{-3}\alpha (2\eta ^\sharp +\nabla \!\!\!\!/\log a,\beta ^\sharp )-a^{-1}(\rho \beta -\sigma {}^*\beta ,2{\underline{\eta }}-\nabla \!\!\!\!/\log a) \nonumber \\&\quad +\,\frac{1}{4}a^{-2}(a{\hat{{\underline{\chi }}}}+a^{-1}{\hat{\chi }},\rho \alpha -\sigma {}^*\alpha ) \end{aligned}$$

##### Remark 4.10

Note that the formula for $K_+^a$ reduces to (4.48) when $a=1$. Moreover, the formulas in Lemma 4.9 are obtained from those in Lemma 4.5 with the replacements

4.80a $$\begin{aligned}&{\hat{{\underline{\omega }}}}\mapsto a{\hat{{\underline{\omega }}}}+\frac{1}{2}\hat{{\underline{L}}}a\qquad {\hat{\omega }}\mapsto a^{-1}{\hat{\omega }}+\frac{1}{2}\hat{{L}}a^{-1} \end{aligned}$$

4.80b $$\begin{aligned}&{{\,\mathrm{tr}\,}}{\underline{\chi }}+{{\,\mathrm{tr}\,}}\chi \mapsto a{{\,\mathrm{tr}\,}}{\underline{\chi }}+a^{-1}{{\,\mathrm{tr}\,}}\chi -\hat{{\underline{L}}}a-\hat{{L}}a^{-1} \end{aligned}$$

4.80c $$\begin{aligned}&{\underline{\eta }}\mapsto {\underline{\eta }}-\mathrm {d}\!\!\!/\log a\qquad \eta \mapsto \eta +\mathrm {d}\!\!\!/\log a \end{aligned}$$

4.80d $$\begin{aligned}&{\hat{{\underline{\chi }}}}\mapsto a{\hat{{\underline{\chi }}}}\qquad {\hat{\chi }}\mapsto a^{-1}{\hat{\chi }}\end{aligned}$$

##### Proof

As in (4.45) we have

4.81 $$\begin{aligned} \begin{aligned} K^{M_a}&=\frac{3}{8}\frac{1}{\Omega ^2}Q[W]_{\alpha \beta \gamma \delta }{}^{(M)}{\hat{\pi }}^{\alpha \beta } e_3^\gamma e_3^\delta +\frac{3}{4}\frac{1}{\Omega ^2}Q[W]_{\alpha \beta \gamma \delta }{}^{(M)}{\hat{\pi }}^{\alpha \beta } e_3^\gamma e_4^\delta \\&\qquad +\frac{3}{8}\frac{1}{\Omega ^2}Q[W]_{\alpha \beta \gamma \delta }{}^{(M)}{\hat{\pi }}^{\alpha \beta } e_4^\gamma e_4^\delta \end{aligned} \end{aligned}$$

The statement of the Lemma then follows from the following contributions:

4.82 $$\begin{aligned} \Omega \,Q_{\alpha \beta \gamma \delta }\,{\hat{\pi }}^{\alpha \beta }e_3^\gamma e_3^\delta= & {} \bigl (2a^{-1}{\hat{\omega }}+\hat{{L}}a^{-1}\bigr )a^4|{\underline{\alpha }}|^2+2(a{{\,\mathrm{tr}\,}}{\underline{\chi }}+a^{-1}{{\,\mathrm{tr}\,}}\chi -\hat{{\underline{L}}}a-\hat{{L}}a^{-1})\nonumber \\&\quad a^2|{\underline{\beta }}|^2 +\bigl (4a {\hat{{\underline{\omega }}}}+2\hat{{\underline{L}}}a\bigr )(\rho ^2+\sigma ^2)\nonumber \\&\quad +\,8a^3{\underline{\alpha }}(2{\underline{\eta }}^\sharp -\nabla \!\!\!\!/\log a,{\underline{\beta }}^\sharp )+8a(\rho {\underline{\beta }}+\sigma {}^*{\underline{\beta }},2\eta +\nabla \!\!\!\!/\log a)\nonumber \\&\quad +2a^2(a{\hat{{\underline{\chi }}}}+a^{-1}{\hat{\chi }},\rho {\underline{\alpha }}+\sigma {}^*{\underline{\alpha }}) \end{aligned}$$

4.83 $$\begin{aligned} \Omega Q_{\alpha \beta \gamma \delta }{\hat{\pi }}^{\alpha \beta }e_3^\gamma e_4^\delta= & {} \bigl (4a^{-1}{\hat{\omega }}+2\hat{{L}}a^{-1}\bigr )a^2|{\underline{\beta }}|^2\nonumber \\&\quad +\,2\bigl (a{{\,\mathrm{tr}\,}}{\underline{\chi }}+a^{-1}{{\,\mathrm{tr}\,}}\chi -\hat{{\underline{L}}}a-\hat{{L}}a^{-1}\bigr )\bigl (\rho ^2+\sigma ^2\bigr )\nonumber \\&\quad +\,\bigl (4a{\hat{{\underline{\omega }}}}+2\hat{{\underline{L}}}a\bigr )a^{-2}|\beta |^2 +8 a(\rho {\underline{\beta }}+\sigma {}^*{\underline{\beta }},2{\underline{\eta }}-\mathrm {d}\!\!\!/\log a)\nonumber \\&\quad -\,8a^{-1}(\rho \beta -\sigma {}^*\beta ,2\eta +\mathrm {d}\!\!\!/\log a)\nonumber \\&\quad -4(a{\hat{{\underline{\chi }}}}+a^{-1}{\hat{\chi }})({\underline{\beta }}^\sharp ,\beta ^\sharp ) \end{aligned}$$

4.84 $$\begin{aligned} \Omega \,Q_{\alpha \beta \gamma \delta }\,\pi ^{\alpha \beta }\hat{{L}}^\gamma \hat{{L}}^\delta= & {} \bigl (2a{\hat{{\underline{\omega }}}}+\hat{{\underline{L}}}a\bigr )a^{-4}|\alpha |^2\nonumber \\&\quad +2\bigl (a{{\,\mathrm{tr}\,}}{\underline{\chi }}+a^{-1}{{\,\mathrm{tr}\,}}\chi -\hat{{\underline{L}}}a-\hat{{L}}a^{-1}\bigr )a^{-2}|\beta |^2 \nonumber \\&\quad +\,\bigl (4a^{-1}{\hat{\omega }}+2\hat{{L}}a^{-1}\bigr )(\rho ^2\!+\!\sigma ^2) -8a^{-3}\alpha (2\eta ^\sharp \!+\!\nabla \!\!\!\!/\log a,\beta ^\sharp )\nonumber \\&\quad -\,8a^{-1}(\rho \beta -\sigma {}^*\beta ,2{\underline{\eta }}-\nabla \!\!\!\!/\log a) \nonumber \\&\quad +\,2a^{-2}(a{\hat{{\underline{\chi }}}}+a^{-1}{\hat{\chi }},\rho \alpha -\sigma {}^*\alpha ) \end{aligned}$$

$$\square $$

### Global redshift estimate

In this Section we will show that the energy on $\Sigma _r$ associated to the current $P^M[W]$ decays uniformly in *r*. The decay mechanism lies in the expansion of the spacetime — as manifested in our assumptions, in particular ${{\,\mathrm{tr}\,}}\chi >0$, and ${{\,\mathrm{tr}\,}}{\underline{\chi }}>0$ — and results in the positivity of the $K^M$: In Section 4.2 we have proven that $K_+^M$ is positive, and in this section we will show that $K_-^M$ is an error which can be absorbed in $K_+^M$.

In order to exploit the positivity of $$K_+[W]$$ in the bulk term — in comparison to the flux terms associated to $$ P^M[W]$$ — we need a version of the coarea formula: We foliate the spacetime domain $${\mathcal {D}}$$ by the level sets of $$r(u,v)=c$$, and first note that we have already calculated the normal separation of the leaves in Lemma 2.1:

4.85 $$\begin{aligned} \phi =\frac{1}{\sqrt{-g(V,V)}}=\frac{2}{r}\frac{\Omega }{\sqrt{\overline{\Omega {{\,\mathrm{tr}\,}}\chi }\,\overline{\Omega {{\,\mathrm{tr}\,}}{\underline{\chi }}}}} \end{aligned}$$

Therefore we have, for any function *f*,

4.86 $$\begin{aligned} \int _{{\mathcal {D}}}f\mathrm {d}\mu _{g}=\int \mathrm {d}r\int _{\Sigma _r}\phi f\mathrm {d}\mu _{{\overline{g}}_{r}}=\int \mathrm {d}r\int _{\Sigma _r}\frac{2}{r}\frac{\Omega f}{\sqrt{\overline{\Omega {{\,\mathrm{tr}\,}}\chi }\,\overline{\Omega {{\,\mathrm{tr}\,}}{\underline{\chi }}}}}\mathrm {d}\mu _{{\overline{g}}_{r}} \end{aligned}$$

Will first demonstrate the positivity of $K^M=K^M_++K^M_-$ under assumptions that are explicitly stated in the following Lemma 4.11 and Lemma 4.12. These assumptions are slightly more general than the assumptions (**BA:I**) which we discuss below to prove the positivity of $K^{M_q}$ in Lemma 4.14.

#### Lemma 4.11

Let the null structure coefficients satisfy

4.87a $$\begin{aligned}&{{\,\mathrm{tr}\,}}\chi>0\qquad {{\,\mathrm{tr}\,}}{\underline{\chi }}>0 \end{aligned}$$

4.87b $$\begin{aligned}&|2\omega -\Omega {{\,\mathrm{tr}\,}}\chi |\le \epsilon \, \Omega {{\,\mathrm{tr}\,}}\chi \qquad |2{\underline{\omega }}- \Omega {{\,\mathrm{tr}\,}}{\underline{\chi }}|\le \epsilon \, \Omega {{\,\mathrm{tr}\,}}{\underline{\chi }}\end{aligned}$$

4.87c $$\begin{aligned}&|\Omega {{\,\mathrm{tr}\,}}\chi -\overline{\Omega {{\,\mathrm{tr}\,}}\chi }|\le \epsilon \,\overline{\Omega {{\,\mathrm{tr}\,}}\chi } \qquad |\Omega {{\,\mathrm{tr}\,}}{\underline{\chi }}-\overline{\Omega {{\,\mathrm{tr}\,}}{\underline{\chi }}}|\le \epsilon \,\overline{\Omega {{\,\mathrm{tr}\,}}{\underline{\chi }}} \end{aligned}$$

for some $\epsilon >0$. Then

4.88 $$\begin{aligned} \int _{{\mathcal {D}}} K_+[W] \mathrm {d}\mu _{g}\ge 6\int \frac{\mathrm {d}r}{r}\int _{\Sigma _r}\,(1-\epsilon )^2\,{}^*P^M[W]\,. \end{aligned}$$

#### Proof

Consider the expression (4.48) for $K_+$ in Lemma 4.5. We compare the coefficients to each curvature term with the corresponding coefficients in the expression (4.37) for the curvature flux ${}^*P^M$ in Lemma 4.3. We begin with $\alpha$, ${\underline{\alpha }}$, $\beta$, ${\underline{\beta }}$:

$$\begin{aligned}&2\biggl [\frac{3}{4}{\hat{\omega }}|{\underline{\alpha }}[W]|^2+3{\hat{\omega }}|{\underline{\beta }}[W]|^2+\frac{3}{4}\bigl ({{\,\mathrm{tr}\,}}\chi +{{\,\mathrm{tr}\,}}{\underline{\chi }}\bigr )\Bigr (|{\underline{\beta }}[W]|^2+|\beta [W]|^2\Bigr ) \\&\quad +\,3{\hat{{\underline{\omega }}}}|\beta [W]|^2+\frac{3}{4}{\hat{{\underline{\omega }}}}|\alpha [W]|^2\biggr ]\\&\quad \ge \frac{6(1-\epsilon )}{8}\biggl [{{\,\mathrm{tr}\,}}\chi |{\underline{\alpha }}[W]|^2+2\Bigl (3{{\,\mathrm{tr}\,}}\chi +{{\,\mathrm{tr}\,}}{\underline{\chi }}\Bigr )|{\underline{\beta }}[W]|^2\\&\quad +\,2\Bigl ({{\,\mathrm{tr}\,}}\chi +3{{\,\mathrm{tr}\,}}{\underline{\chi }}\Bigr )|\beta [W]|^2+{{\,\mathrm{tr}\,}}{\underline{\chi }}|\alpha [W]|^2\biggr ] \end{aligned}$$

and continue with $\rho$, $\sigma$:

$$\begin{aligned}&2\frac{3}{2}\Bigl [{\hat{{\underline{\omega }}}}+{\hat{\omega }}+{{\,\mathrm{tr}\,}}\chi +{{\,\mathrm{tr}\,}}{\underline{\chi }}\Bigr ]\bigl (\rho [W]^2+\sigma [W]^2\bigr ) \\&\quad \ge \frac{6(1-\epsilon )}{8}6\Bigl [{{\,\mathrm{tr}\,}}\chi +{{\,\mathrm{tr}\,}}{\underline{\chi }}\Bigr ]\bigl (\rho [W]^2+\sigma [W]^2\bigr ) \end{aligned}$$

We add up these two inequalties, and in view of the formula (4.85) for the lapse function, it then follows immediately from Lemma 4.5 and Lemma 4.3 that

$$\begin{aligned} \phi K_+\mathrm {d}\mu _{{\overline{g}}_{r}}\ge \frac{6(1-\epsilon )^2}{r}\,{}^*P^M \end{aligned}$$

and therefore by the co-area formula the statement of the Lemma follows. $\square$

It remains to estimate the error terms occuring in Lemma 4.5.

#### Lemma 4.12

Let the structure coefficients satisfy

4.89a $$\begin{aligned}&4 |{\underline{\eta }}|_{g\!\!\!/} \le \epsilon {\hat{\omega }}\quad 4 |\eta |_{g\!\!\!/} \le \epsilon {\hat{{\underline{\omega }}}}\end{aligned}$$

4.89b $$\begin{aligned}&4 |{\underline{\eta }}|_{g\!\!\!/} \le \epsilon ({{\,\mathrm{tr}\,}}\chi +{{\,\mathrm{tr}\,}}{\underline{\chi }}) \qquad 4 |\eta |_{g\!\!\!/} \le \epsilon ({{\,\mathrm{tr}\,}}\chi +{{\,\mathrm{tr}\,}}{\underline{\chi }}) \end{aligned}$$

4.89c $$\begin{aligned}&4 |{\hat{{\underline{\chi }}}}|_{g\!\!\!/} \le \epsilon {\hat{\omega }}\quad 4 |{\hat{\chi }}|_{g\!\!\!/} \le \epsilon {\hat{\omega }}\end{aligned}$$

4.89d $$\begin{aligned}&4 |{\hat{{\underline{\chi }}}}|_{g\!\!\!/} \le \epsilon {\hat{{\underline{\omega }}}}\quad 4 |{\hat{\chi }}|_{g\!\!\!/} \le \epsilon {\hat{{\underline{\omega }}}}\end{aligned}$$

for some $\epsilon >0$. Then

4.90 $$\begin{aligned} |K_-|\le \epsilon K_+\,. \end{aligned}$$

#### Proof

Immediate from the expressions (4.48) and (4.49) obtained in Lemma 4.5. $\square$

We will state the redshift estimate for $M_q$ under the stronger assumptions (${\varvec{BA:I}}.i$), (${\varvec{BA:I}}.ii$), (${\varvec{BA:I}}.iii$), (${\varvec{BA:I}}.iv$), introduced in Section 1.2, which correspond to the choice $\epsilon = C_0\Omega ^{-1}$ in Lemma 4.11, 4.12: 
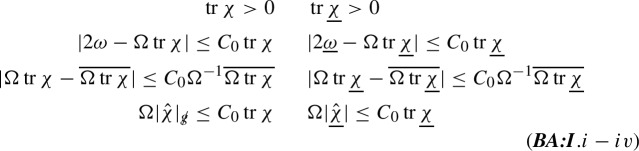
 and the additional assumptions 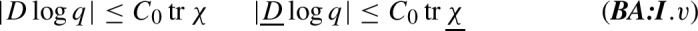

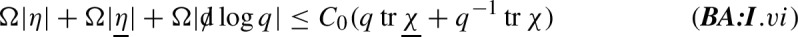


#### Remark 4.13

With these assumptions — together with (${\varvec{BA:III}}.i$) recalled below — the error as quantified in Lemma 4.14 is of one order *r* smaller than the error arising from the assumptions in Lemma 4.11 and Lemma 4.12. This is ultimately the reason the decay rate of the Weyl curvature is $r^{-3}$ as opposed to $r^{-3+\epsilon }$.

Let us denote for simplicity by

4.91 $$\begin{aligned} P^q[W]:= P^{(M_q,M_q,M_q)}\,,\qquad K^q[W]:=K^{(M_q,M_q,M_q)}[W]\,. \end{aligned}$$

#### Lemma 4.14

Let the structure coefficients satisfy (**BA:I**) for some $C_0>0$. Then

4.92 $$\begin{aligned} \phi K_+^q[W]\mathrm {d}\mu _{{\overline{g}}_{r}}\ge \frac{6}{r} \bigl (1-2C_0\Omega ^{-1}\bigr )^2 \,{}^*P^q[W] \end{aligned}$$

Moreover, for some constant $C>0$,

4.93 $$\begin{aligned} \phi |K_-^q[W] |\le C C_0 \Omega ^{-1} \phi K_+^q[W]\,. \end{aligned}$$

#### Proof

Note first that under the assumptions on the structure coefficients

$$\begin{aligned}&\Omega |\hat{{\underline{L}}}q|\le C_0 q{{\,\mathrm{tr}\,}}{\underline{\chi }}\qquad \Omega |\hat{{L}}q^{-1}|\le C_0 q^{-1}{{\,\mathrm{tr}\,}}\chi \\&\quad 2 q^{-1}{\hat{\omega }}+\hat{{L}}q^{-1}\ge (1-2C_0\Omega ^{-1}) q^{-1}{{\,\mathrm{tr}\,}}\chi \\&\quad q{{\,\mathrm{tr}\,}}{\underline{\chi }}+q^{-1}{{\,\mathrm{tr}\,}}\chi +4q^{-1}{\hat{\omega }}-\hat{{\underline{L}}}q+\hat{{L}}q^{-1}\\&\quad \ge (1-C_0\Omega ^{-1})q{{\,\mathrm{tr}\,}}{\underline{\chi }}+3 (1-C_0\Omega ^{-1}) q^{-1}{{\,\mathrm{tr}\,}}\chi \\&\quad 4q {\hat{{\underline{\omega }}}}+4q{{\,\mathrm{tr}\,}}{\underline{\chi }}-2\hat{{\underline{L}}}q+4q^{-1}{\hat{\omega }}+4q^{-1}{{\,\mathrm{tr}\,}}\chi -2\hat{{L}}q^{-1}\\&\quad \ge (6-4 C_0\Omega ^{-1})q{{\,\mathrm{tr}\,}}{\underline{\chi }}+(6-4 C_0\Omega ^{-1})q^{-1}{{\,\mathrm{tr}\,}}\chi \\&\quad q{{\,\mathrm{tr}\,}}{\underline{\chi }}+q^{-1}{{\,\mathrm{tr}\,}}\chi +4q{\hat{{\underline{\omega }}}}+\hat{{\underline{L}}}q-\hat{{L}}q^{-1} \\&\quad \ge 3(1-C_0\Omega ^{-1}) q{{\,\mathrm{tr}\,}}{\underline{\chi }}+(1-C_0\Omega ^{-1})q^{-1}{{\,\mathrm{tr}\,}}\chi \\&\quad 2q{\hat{{\underline{\omega }}}}+\hat{{\underline{L}}}q \ge (1-2 C_0\Omega ^{-1})q{{\,\mathrm{tr}\,}}{\underline{\chi }}\end{aligned}$$

Hence by Lemma 4.9:

$$\begin{aligned}&\frac{(2\Omega )^3}{3}K_+^q[W]\ge (1-2C_0\Omega ^{-1}) q^{-1}{{\,\mathrm{tr}\,}}\chi q^4|{\underline{\alpha }}|^2\\&\quad +\,2(1-C_0\Omega ^{-1})\bigl (q{{\,\mathrm{tr}\,}}{\underline{\chi }}+3 q^{-1}{{\,\mathrm{tr}\,}}\chi \bigr )q^2|{\underline{\beta }}|^2\\&\quad +\,(6-4C_0\Omega ^{-1})\bigl (q{{\,\mathrm{tr}\,}}{\underline{\chi }}+q^{-1}{{\,\mathrm{tr}\,}}\chi \bigr )(\rho ^2+\sigma ^2)\\&\quad +\,2(1-C_0\Omega ^{-1})\bigl (3 q{{\,\mathrm{tr}\,}}{\underline{\chi }}+q^{-1}{{\,\mathrm{tr}\,}}\chi \bigr )q^{-2}|\beta |^2 +(1-2C_0\Omega ^{-1})q{{\,\mathrm{tr}\,}}{\underline{\chi }}q^{-4}|\alpha |^2 \end{aligned}$$

Secondly note that by the definition of *q*,

$$\begin{aligned} \phi \, q^{-1}{{\,\mathrm{tr}\,}}\chi= & {} \frac{2}{r}\frac{\Omega {{\,\mathrm{tr}\,}}\chi }{\overline{\Omega {{\,\mathrm{tr}\,}}\chi }}\ge \frac{2}{r}(1-C_0\Omega ^{-1})\\ \phi \, q{{\,\mathrm{tr}\,}}{\underline{\chi }}= & {} \frac{2}{r}\frac{\Omega {{\,\mathrm{tr}\,}}{\underline{\chi }}}{\overline{\Omega {{\,\mathrm{tr}\,}}{\underline{\chi }}}}\ge \frac{2}{r}(1-C_0\Omega ^{-1}) \end{aligned}$$

Therefore

$$\begin{aligned}&\frac{1}{3} \phi K_+^q[W]\ge \frac{2}{r}\frac{(1-2C_0\Omega ^{-1})^2}{(2\Omega )^3}\biggl [ q^4|{\underline{\alpha }}|^2\\&\quad +\, 8q^2|{\underline{\beta }}|^2+12(\rho ^2+\sigma ^2)+8q^{-2}|\beta |^2 + q^{-4}|\alpha |^2\biggr ] \end{aligned}$$

and thus in comparison to (4.75),

$$\begin{aligned} \phi K_+^q \mathrm {d}\mu _{{\overline{g}}_{r}} \ge (1-2C_0\Omega ^{-1})^2\,\frac{6}{r}\,{}^*P^{M_q} \end{aligned}$$

The final bound (4.93) follows by inspection of the formulas given in Lemma 4.9. $\square$

Now by (${\varvec{BA:III}}.i$) we assume

4.94 $$\begin{aligned} \frac{C_0}{r}\ge \Omega ^{-1}\ge \frac{1}{C_0 r}\,; \end{aligned}$$

this uniform bound of $\Omega$ on $\Sigma _r$ is necessary for the sharp decay rate in the following Proposition. We will state the main conclusion for the energy current associated to $M_q$, but this result of course also holds for the energy associated to *M* and the same proof applies.

#### Proposition 4.15

Assume (**BA:I**) and (${\varvec{BA:III}}.i$) for some $C_0>0$. Then there exists a constant $C(r_0)$ such that for any solution *W* to (1.27) with initial data such that

4.95 $$\begin{aligned} D^q[W]=\int _{\Sigma _{r_0}}{}^*P^q[W]<\infty \end{aligned}$$

we have

4.96 $$\begin{aligned} r^{6}\int _{\Sigma _{r}}{}^*P^q[W]\le C( r_0 ) D^q[W]\,,\qquad (r\ge r_0)\,. \end{aligned}$$

#### Proof

Apply Proposition 4.1 to the energy current $P^q[W]$ to obtain the inequality

$$\begin{aligned} \int _{\Sigma _{r_2}^c}{}^*P^q[W] + \int _{{\mathcal {D}}_{(r_1,r_2)}} K^q[W]\mathrm {d}\mu _{g}\le \int _{\Sigma _{r_1}^c}{}^*P^q[W] \end{aligned}$$

for any $r_2>r_1>r_0$. Here we used that the flux terms through the null hypersurfaces $C_{{\underline{u}}}^c$ and ${\underline{C}}_{{\underline{v}}}^c$ as given by (4.21) are nonnegative due to the positivity properties of *Q*(*W*) (see Section 1.4 and Proposition 12.5 in [8]) which allows us to drop the first and the fourth term on the left hand side of (4.23):

4.97 $$\begin{aligned} \int _{C_u} {}^*P^q =\int \Omega Q[W](\hat{{L}},M_q,M_q,M_q)\mathrm {d}v\mathrm {d}\mu _{g\!\!\!/}\ge 0\qquad \int _{{\underline{C}}_{{\underline{v}}}^c} {}^*P^q\ge 0 \end{aligned}$$

Moreover the third term on the left hand side of (4.23) vanishes by (1.29). Let us choose $r_0$ sufficiently large, so that

$$\begin{aligned} CC_0\Omega ^{-1}< 1\qquad (r\ge r_0) \end{aligned}$$

Then by the co-area formula (4.86), and by Lemma 4.14,

$$\begin{aligned}&\int _{{\mathcal {D}}_{(r_1,r_2)}} K^q[W]\mathrm {d}\mu _{g} = \int _{r_1}^{r_2}\mathrm {d}r \int _{\Sigma _r^c}\phi K^q[W] \mathrm {d}\mu _{{\overline{g}}_{r}}\\&\quad \ge \int _{r_1}^{r_2}\mathrm {d}r \int _{\Sigma _r^c}(1-C C_0\Omega ^{-1})\phi K_+^q[W] \mathrm {d}\mu _{{\overline{g}}_{r}}\\&\quad \ge 6 \int _{r_1}^{r_2} \frac{\mathrm {d}r}{r}\bigl (1-\frac{CC_0^2}{r}\bigr )^3\int _{\Sigma _r^c} {}^*P^q[W] \end{aligned}$$

which implies the inequality

$$\begin{aligned}&\int _{\Sigma _{r_2}^c}{}^*P^q[W] + 6 \int _{r_1}^{r_2}\mathrm {d}r\frac{1}{r}\int _{\Sigma _r^c} {}^*P^q[W]\\&\quad \le \int _{\Sigma _{r_1}^c}{}^*P^q[W]+ C \int _{r_1}^{r_2}\mathrm {d}r\frac{1}{r^2}\int _{\Sigma _r^c} {}^*P^q[W]\,. \end{aligned}$$

A Gronwall-type argument then implies the statement of the Proposition, see Section 5.4.

Note that in view (4.95), and again using (4.97) we can pass from the estimate on ${\mathcal {D}}^{({\underline{u}},{\underline{v}})}_{(r_1,r_2)}$ defined in (4.11) to the unbounded domain $\cup _{r_1\le r\le r_2}\Sigma _r$ by taking the limits ${\underline{u}}\rightarrow \infty$, and ${\underline{v}}\rightarrow \infty$. $\square$

#### Remark 4.16

An equivalent statement can also be derived for a weighted null flux.

## First order redshift

The aim of this Section is derive an energy estimate for $\nabla W$, similar to the redshift estimate for *W* in Section 4.3. This is achieved by commuting the Bianchi equations (1.27) with a vectorfield *X*, which yields an inhomogeneous equation of the form (5.6) for the modified Lie derivative $\tilde{{\mathcal {L}}}_{X}W$. The strategy here is to choose *X* to be future-directed time-like, in fact colinear with the normal to $\Sigma _r$, and to derive a redshift estimate for solutions $\tilde{{\mathcal {L}}}_{X}W$ to (5.6), which can then be used to control all derivatives ${\overline{\nabla }}W$ *tangential* to $\Sigma _r$. This last step relies on an elliptic estimate in the context of the electro-magnetic decomposition of *W* with respect to $\Sigma _r$, which we will discuss separately in Section 6.

A natural choice of the commutator would be

5.1 $$\begin{aligned} M_q=\frac{1}{2\Omega }\bigl (e_3+e_4) \end{aligned}$$

where

5.2 $$\begin{aligned} e_3=q\hat{{\underline{L}}}\qquad e_4=q^{-1}\hat{{L}}\,; \end{aligned}$$

namely the “aligned” redshift vectorfield of Section 4.2. The task is then to exhibit a positivity property of

5.3 $$\begin{aligned} \bigl ({{\,\mathrm{div}\,}}Q(\tilde{{\mathcal {L}}}_{M_q}W)\bigr )(M_q,M_q,M_q)\,, \end{aligned}$$

which appears as an additional term in the energy identity of Proposition 4.1, for solutions to the inhomogeneous Bianchi equations. While (5.3) *does* have a sign in the highest order terms $\nabla W$, the lower order terms at the level of *W* still form an obstruction to the required decay *rate* of the energy associated to $\tilde{{\mathcal {L}}}_{M_q}W$ on $\Sigma _r$. We choose instead as commutator vectorfield:^40^

5.4 $$\begin{aligned} N=\Omega ^2 M_q \end{aligned}$$

### Remark 5.1

In this section *n* will denote one of the components of the deformation tensor of *N* to be defined in (5.11c), and *not* the unit norm to $\Sigma _r$.

### Remark 5.2

Note that *N* is orthogonal to the spheres $S_{u,v}$. So while the scalars *q* and $\Omega$ determine its direction and magnitude in the plane $\langle \hat{{\underline{L}}},\hat{{L}}\rangle \subset \mathrm {T}{\mathcal {M}}$ spanend by $(\hat{{\underline{L}}},\hat{{L}})$, the plane itself always satisfies $\langle \hat{{\underline{L}}},\hat{{L}}\rangle ^\perp \subset \mathrm {T}S_{u,v}$. Its orientation thus depends on the spheres $S_{u,v}$ whose embedding in ${\mathcal {M}}$ is characterised by the assumptions $({\textbf {BA}})$.

Now by the classical Proposition 12.1 in [8] the Weyl field $\tilde{{\mathcal {L}}}_{N}W$,

5.5 $$\begin{aligned}&\tilde{{\mathcal {L}}}_{N}W_{\alpha \beta \gamma \delta }={\mathcal {L}}_NW_{\alpha \beta \gamma \delta }-\frac{1}{8}{{\,\mathrm{tr}\,}}{}^{(N)}\pi \,W_{\alpha \beta \gamma \delta }\nonumber \\&\quad -\,\frac{1}{2}\Bigl [{}^{(N)}{\hat{\pi }}_{\alpha }^{\mu }W_{\mu \beta \gamma \delta }+{}^{(N)}{\hat{\pi }}_{\beta }^{\mu }W_{\alpha \mu \gamma \delta }+{}^{(N)}{\hat{\pi }}_{\gamma }^{\mu }W_{\alpha \beta \mu \delta }+{}^{(N)}{\hat{\pi }}_{\delta }^{\mu }W_{\alpha \beta \gamma \mu }\Bigr ]\qquad \quad \end{aligned}$$

satisfies the equation

5.6 $$\begin{aligned} \nabla ^\alpha \bigl (\tilde{{\mathcal {L}}}_{N}W\bigr )_{\alpha \beta \gamma \delta }={}^{(N)}J(W)_{\beta \gamma \delta } \end{aligned}$$

where the Weyl current ${}^{(N)}J(W)$ is detailed below in (5.8). Therefore, according to Lemma 12.3 in [8] we have the important formula

5.7 $$\begin{aligned}&8\Omega ^3 \bigl ({{\,\mathrm{div}\,}}Q(\tilde{{\mathcal {L}}}_{N}W)\bigr )(M_q,M_q,M_q) = 4({\tilde{{\underline{\alpha }}}}_q,\tilde{{\underline{\Theta }}}_q)+8({\tilde{{\underline{\beta }}}}_q,\tilde{{\underline{\Xi }}}_q)\nonumber \\&\quad +\,3\Bigl (8{\tilde{\rho }}_q\bigl (\tilde{{\underline{\Lambda }}}_q+{\tilde{\Lambda }}_q\bigr )-8{\tilde{\sigma }}_q\bigl (\tilde{{\underline{K}}}_q-{\tilde{K}}_q\bigr )-8({\tilde{{\underline{\beta }}}}_q,\tilde{{\underline{I}}}_q)+8({\tilde{\beta }}_q,{\tilde{I}}_q)\Bigr )\nonumber \\&\quad +\,4({\tilde{\alpha }}_q,{\tilde{\Theta }}_q)-8({\tilde{\beta }}_q,{\tilde{\Xi }}_q) \end{aligned}$$

where ${\tilde{{\underline{\alpha }}}}_q,{\tilde{{\underline{\beta }}}}_q$,${\tilde{\rho }}_q,{\tilde{\sigma }}_q$,${\tilde{\beta }}_q,{\tilde{\alpha }}_q$ refer to the null components of $\tilde{{\mathcal {L}}}_{N}W$, and $\tilde{{\underline{\Xi }}}_q,{\tilde{\Xi }}_q$, $\tilde{{\underline{\Lambda }}}_q,{\tilde{\Lambda }}_q$, $\tilde{{\underline{K}}}_q,{\tilde{K}}_q$, $\tilde{{\underline{\Theta }}}_q,{\tilde{\Theta }}_q$ refer to the null components of ${}^{(N)}J(W)$.

### Remark 5.3

Throughout this Section we use a null decomposition with respect to the null frame $(e_3,e_4;e_A)$. However the null structure coefficents are still associated to the frame coming from the foliation $(\hat{{\underline{L}}},\hat{{L}};e_A)$. To avoid confusion we append a subscript *q* to any null components decomposed relative to $(e_3,e_4;e_A)$. In particular in reference to (5.7) we have

$$\begin{aligned}&({\tilde{{\underline{\alpha }}}}_q)_{AB}:=({\underline{\alpha }}_q[\tilde{{\mathcal {L}}}_{N}W])_{AB}:=\tilde{{\mathcal {L}}}_{N}W(e_A,e_3,e_B,e_3)\\&({\tilde{\alpha }}_q)_{AB}:=\tilde{{\mathcal {L}}}_{N}W(e_A,e_4,e_B,e_4)\\&({\tilde{{\underline{\beta }}}}_q)_A:=({\underline{\beta }}_q[\tilde{{\mathcal {L}}}_{N}W])_{A}:=\frac{1}{2}\tilde{{\mathcal {L}}}_{N}W(e_A,e_3,e_3,e_4)\\&({\tilde{\beta }}_q)_A:=\frac{1}{2}\tilde{{\mathcal {L}}}_{N}W(e_A,e_4,e_3,e_4)\\&{\tilde{\rho }}_q:=\frac{1}{4}(\tilde{{\mathcal {L}}}_{N}W)(e_3,e_4,e_3,e_4)\\&{\tilde{\sigma }}_q:=\frac{1}{4}\epsilon \!\!/^{AB}(\tilde{{\mathcal {L}}}_{N}W)(e_A,e_B,e_3,e_4) \end{aligned}$$

where in fact ${\tilde{\rho }}_q={\tilde{\rho }}$ and ${\tilde{\sigma }}_q={\tilde{\sigma }}$ by (4.73c), and

$$\begin{aligned}&(\tilde{{\underline{\Xi }}}_q)_A:=({\underline{\Xi }}_q[{}^{(N)} J(W)])_A:=\frac{1}{2}{}^{(N)} J(W)_{33A}\\&\quad ({\tilde{\Xi }}_q)_A:=\frac{1}{2}{}^{(N)} J(W)_{44A}\\&\tilde{{\underline{\Lambda }}}_q:={\underline{\Lambda }}_q[{}^{(N)}J(W)]:=\frac{1}{4}{}^{(N)}J(W)_{343}\\&\quad {\tilde{\Lambda }}_q:=\frac{1}{4}{}^{(N)}J(W)_{434}\\&\tilde{{\underline{K}}}_q:={\underline{K}}_q[{}^{(N)}J(W)]:=\frac{1}{4}\epsilon \!\!/^{AB}{}^{(N)}J(W)_{3AB}\\&\quad {\tilde{K}}_q:=\frac{1}{4}\epsilon \!\!/^{AB}{}^{(N)}J(W)_{4AB}\\&(\tilde{{\underline{\Theta }}}_q)_{AB}={\underline{\Theta }}({}^{(N)}J(W))_{AB}\\&\quad ({\tilde{\Theta }}_q)_{AB}=\Theta ({}^{(N)}J(W))_{AB}\,, \end{aligned}$$

cf. Chapter 12.2 in [8].

According to Proposition 12.1 in [8] we have that the Weyl current ${}^{(N)}J(W)$ in (5.6) is given by

5.8 $$\begin{aligned} {}^{(N)}J(W)={}^{(N)}J^1(W)+{}^{(N)}J^2(W)+{}^{(N)}J^3(W) \end{aligned}$$

where, cf. (14.5) in [8],

5.9a $$\begin{aligned} {}^{(N)}J^1(W)_{\beta \gamma \delta }= & {} \frac{1}{2}{}^{(N)}{\hat{\pi }}^{\mu \nu }\nabla _\nu W_{\mu \beta \gamma \delta } \end{aligned}$$

5.9b $$\begin{aligned} {}^{(N)}J^2(W)_{\beta \gamma \delta }= & {} \frac{1}{2}p_\mu W^{\mu }_{\beta \gamma \delta } \end{aligned}$$

5.9c $$\begin{aligned} {}^{(N)}J^3(W)_{\beta \gamma \delta }= & {} \frac{1}{2}\Bigl (d_{\mu \beta \nu }W^{\mu \nu }_{\gamma \delta }+d_{\mu \gamma \nu }W^{\mu \nu }_{\beta \delta }+d_{\mu \delta \nu }W^{\mu \nu }_{\beta \gamma }\Bigr ) \end{aligned}$$

and^41^

5.10a $$\begin{aligned}&p_\mu := {}^{(N)}p_\mu := \nabla ^\alpha {}^{(N)}\,{\hat{\pi }}_{\alpha \mu } \end{aligned}$$

5.10b $$\begin{aligned}&d_{\alpha \beta \gamma }:={}^{(N)}d_{\alpha \beta \gamma }:=\nabla _\beta {}^{(N)}{\hat{\pi }}_{\gamma \alpha }-\nabla _\gamma {}^{(N)}{\hat{\pi }}_{\beta \alpha }+\frac{1}{3}\Bigl (p_\beta g_{\alpha \gamma }-p_\gamma g_{\alpha \beta }\Bigr )\qquad \quad \end{aligned}$$

### Remark 5.4

Note that only the part $J^1(W)$ contains terms $\nabla W$, and thus only the null decomposition of $J^1$ may contain ${\tilde{{\underline{\alpha }}}},{\tilde{\alpha }}$,${\tilde{{\underline{\beta }}}},{\tilde{\beta }}$,${\tilde{\rho }},{\tilde{\sigma }}$. We sometimes refer to these as “principal terms”. In the first place it suffices then to look at the components of $J^1$ to show the presence of positive quadratic terms in $\nabla W$ in (5.7). Its null components are calculated in Lemma 14.1 in [8]; in fact, the formulas in Lemma 14.1 in [8] are only true in the special case that two components of the deformation tensor $n={\underline{n}}=0$ (defined below) vanish, and we will discuss the general case in Section 5.2.

### Remark 5.5

The discussion of the “lower order” terms — involving *W* — then requires the inclusion of the parts $J^2$, and $J^3$. The null decompositions of $J^2$ and $J^3$ are detailed in Chapter 14.3-4 in [8], cf. Lemma 14.2 in [8]. We emphasize that these parts cannot be treated separately, because cancellations appear accross the expressions for $J^1$, $J^2$, and $J^3$. The relevant terms are collected under the label *X* — in fact, $X:=X^1+X^{2+3}$ where $X^1$ is defined in (5.28) and refers to the relevant terms appearing in $J^1$, and $X^{2+3}$ is defined in (5.50) and refers to the relevant terms coming from the currents $J^2$ and $J^3$ — and the cancellations are exhibited in Section 5.3.3. We also refer to Remark 5.13 below — where we discuss specific terms contributing to these cancellations — for further explanation of the choice of (5.12).

We adopt the notation of Chapter 8.1 in [8] for the components of the deformation tensor, i.e.

5.11a $$\begin{aligned}&i:={}^{(N)}i:={}^{(N)}{\hat{\pi }}\!\!\!/ \end{aligned}$$

5.11b $$\begin{aligned}&{\underline{m}}:={}^{(N)}{\underline{m}}_q:={}^{(N)}{\hat{\pi }}\!\!\!/_3\qquad m:={}^{(N)}m_q:={}^{(N)}{\hat{\pi }}\!\!\!/_4 \end{aligned}$$

5.11c $$\begin{aligned}&{\underline{n}}:={}^{(N)}{\underline{n}}_q:={}^{(N)}{\hat{\pi }}_{33}\qquad n:={}^{(N)}n_q:={}^{(N)}{\hat{\pi }}_{44} \end{aligned}$$

5.11d $$\begin{aligned}&j:={}^{(N)}{\hat{\pi }}_{34} \end{aligned}$$

and calculate here in particular the values for the commutator vectorfield *N*; recall also our results from Lemma. 4.8.

### Lemma 5.6

The components of the deformation tensor of

5.12 $$\begin{aligned} N=\frac{\Omega }{2}\bigl (e_3+e_4) \end{aligned}$$

are

5.13a $$\begin{aligned} i= & {} \Omega \bigl (q{\hat{{\underline{\chi }}}}+q^{-1}{\hat{\chi }}\bigr )+\frac{\Omega }{4}\bigl (q{{\,\mathrm{tr}\,}}{\underline{\chi }}+q^{-1}{{\,\mathrm{tr}\,}}\chi -2q{\hat{{\underline{\omega }}}}-2q^{-1}{\hat{\omega }}-\hat{{\underline{L}}}q-\hat{{L}}q^{-1} \bigr )g\!\!\!/\nonumber \\ \end{aligned}$$

5.13b $$\begin{aligned} {\underline{m}}= & {} \Omega \bigl (2\zeta +\mathrm {d}\!\!\!/\log q\bigr )\qquad m=-\Omega \bigl (2\zeta +\mathrm {d}\!\!\!/\log q\bigr ) \end{aligned}$$

5.13c $$\begin{aligned} {\underline{n}}= & {} 2\Omega \hat{{\underline{L}}}q\qquad n=2\Omega \hat{{L}}q^{-1} \end{aligned}$$

5.13d $$\begin{aligned} j= & {} \frac{\Omega }{2}\Bigl (q{{\,\mathrm{tr}\,}}{\underline{\chi }}+q^{-1}{{\,\mathrm{tr}\,}}\chi -2q{\hat{{\underline{\omega }}}}-2q^{-1}{\hat{\omega }}-\hat{{\underline{L}}}q-\hat{{L}}q^{-1}\Bigr ) \end{aligned}$$

### Proof

Recall that we have already calculated the deformation tensor of $M_q$ in Lemma. 4.8. Moreover,

$$\begin{aligned} {}^{(N)}\pi _{34}= & {} -2\bigl (e_3+e_4\bigr )\Omega +\Omega ^2\,{}^{(M_q)}\pi _{34}\\ {}^{(N)}\pi _{33}= & {} -4e_3\Omega +\Omega ^2\,{}^{(M_q)}\pi _{33}\qquad {}^{(N)}\pi _{44}=-4e_4\Omega +\Omega ^2\,{}^{(M_q)}\pi _{44}\\ {}^{(N)}\pi _{3A}= & {} -2e_A\Omega +\Omega ^2\,{}^{(M_q)}\pi _{3A}\qquad {}^{(N)}\pi _{4A}=-2e_A\Omega +\Omega ^2\,{}^{(M_q)}\pi _{4A}\\ {}^{(N)}\pi _{AB}= & {} \Omega ^2\,{}^{(M_q)}\pi _{AB} \end{aligned}$$

and

$$\begin{aligned} {{\,\mathrm{tr}\,}}{}^{(N)}\pi =2\bigl (e_3+e_4\bigr )\Omega +\Omega ^2{{\,\mathrm{tr}\,}}{}^{(M_q)}\pi \end{aligned}$$

Now recall from (2.40a) that

$$\begin{aligned} e_3\Omega= & {} q{\underline{D}}\log \Omega =q{\underline{\omega }}\qquad e_4\Omega =q^{-1}\omega \\ {{\,\mathrm{tr}\,}}{}^{(M_q)}\pi= & {} \frac{1}{\Omega }\bigl (q{{\,\mathrm{tr}\,}}{\underline{\chi }}+q^{-1}{{\,\mathrm{tr}\,}}\chi +\hat{{\underline{L}}}q+\hat{{L}}q^{-1}\bigr ) \end{aligned}$$

to infer that

$$\begin{aligned} {{\,\mathrm{tr}\,}}{}^{(N)}\pi = \Omega \Bigl ( 2q{\hat{{\underline{\omega }}}}+2q^{-1}{\hat{\omega }}+q{{\,\mathrm{tr}\,}}{\underline{\chi }}+q^{-1}{{\,\mathrm{tr}\,}}\chi +\hat{{\underline{L}}}q+\hat{{L}}q^{-1}\Bigr ) \end{aligned}$$

Moreover, using the results of Lemma 4.8,

$$\begin{aligned} {}^{(N)}{\hat{\pi }}_{34}= & {} -\bigl (q{\underline{\omega }}+q^{-1}\omega \bigr )+\Omega ^2{}^{(M_q)}{\hat{\pi }}_{34}\\&\quad =\frac{\Omega }{2}\Bigl (q{{\,\mathrm{tr}\,}}{\underline{\chi }}+q^{-1}{{\,\mathrm{tr}\,}}\chi -2q{\hat{{\underline{\omega }}}}-2q^{-1}{\hat{\omega }}-\hat{{\underline{L}}}q-\hat{{\underline{L}}}q^{-1}\Bigr )\\ {}^{(N)}{\hat{\pi }}_{33}= & {} 2\Omega \hat{{\underline{L}}}q\qquad {}^{(N)}{\hat{\pi }}_{44}=2\Omega \hat{{L}}q^{-1}\\ {}^{(N)}{\hat{\pi }}_{3A}= & {} \Omega \bigl (2\eta _A+\mathrm {d}\!\!\!/_A\log q-2\mathrm {d}\!\!\!/_A\log \Omega \bigr )=\Omega \bigl (2\zeta _A+\mathrm {d}\!\!\!/_A\log q\bigr )\\ {}^{(N)}{\hat{\pi }}_{4A}= & {} \Omega \bigl (2{\underline{\eta }}_A-\mathrm {d}\!\!\!/_A\log q-2\mathrm {d}\!\!\!/_A\log \Omega \bigr )=-\Omega \bigl (2\zeta _A+\mathrm {d}\!\!\!/_A\log q\bigr )\\ {}^{(N)}{\hat{\pi }}_{AB}= & {} \Omega \bigl (q{\hat{{\underline{\chi }}}}+q^{-1}{\hat{\chi }}\bigr )+\frac{\Omega }{4}\bigl (q{{\,\mathrm{tr}\,}}{\underline{\chi }}+q^{-1}{{\,\mathrm{tr}\,}}\chi -2q{\hat{{\underline{\omega }}}}\\&\qquad -\,2q^{-1}{\hat{\omega }}-\hat{{\underline{L}}}q-\hat{{L}}q^{-1}\bigr ){g\!\!\!/}_{AB} \end{aligned}$$

$$\square $$

Given that in this Section we work mainly with the null decomposition with respect to $(e_3,e_4;e_A)$ we will state here for convenience the form of the Bianchi equations relative to this frame.

### Proposition 5.7

The Bianchi equations decomposed in the frame $(e_3,e_4;e_A)$ read as in Proposition 12.4 in [8] with the following replacements:

5.15a $$\begin{aligned}&D\rightarrow q^{-1}D\qquad {\underline{D}}\rightarrow q{\underline{D}}\end{aligned}$$

5.15b $$\begin{aligned}&\alpha \rightarrow \alpha _q\quad \beta \rightarrow \beta _q\quad {\underline{\beta }}\rightarrow {\underline{\beta }}_q\quad {\underline{\alpha }}\rightarrow {\underline{\alpha }}_q \end{aligned}$$

5.15c $$\begin{aligned}&\chi \rightarrow q^{-1}\chi \qquad {\underline{\chi }}\rightarrow q{\underline{\chi }}\qquad \zeta \rightarrow \zeta +\mathrm {d}\!\!\!/\log q \end{aligned}$$

5.15d $$\begin{aligned}&{\hat{{\underline{\omega }}}}\rightarrow q{\hat{{\underline{\omega }}}}+\Omega ^{-1}{\underline{D}}q\qquad {\hat{\omega }}\rightarrow q^{-1}{\hat{\omega }}+\Omega ^{-1}D q^{-1} \end{aligned}$$

### Proof

First derive the analogous formulas to Proposition 1.1 in [8]. Since these are derived using Leibniz rule and the frame relations (1.175) in [8], it is clear that if instead the relations (4.63) are used, they are formally obtained with replacements

$$\begin{aligned}&\chi \rightarrow q^{-1}\chi \qquad {\underline{\chi }}\rightarrow q{\underline{\chi }}\qquad \zeta \rightarrow \zeta +\mathrm {d}\!\!\!/\log q\\&\quad {\hat{{\underline{\omega }}}}\rightarrow q{\hat{{\underline{\omega }}}}+\Omega ^{-1}{\underline{D}}q\qquad {\hat{\omega }}\rightarrow q^{-1}{\hat{\omega }}+\Omega ^{-1}D q^{-1} \end{aligned}$$

and

$$\begin{aligned} \alpha \rightarrow \alpha _q\quad \beta \rightarrow \beta _q\quad {\underline{\beta }}\rightarrow {\underline{\beta }}_q\quad {\underline{\alpha }}\rightarrow {\underline{\alpha }}_q \end{aligned}$$

because the null decomposition now refers to the frame $(e_3=q\hat{{\underline{L}}}, e_4=q^{-1}\hat{{L}};e_A)$. Therefore (1.190) in [8] holds for the null decomposition of *W* with respect to this frame, and with the above replacements. It remains to check that, by (1.191,1.193) in [8] for any 1-form $\xi$, and any 2-form $\theta$,

$$\begin{aligned} q^{-1}D\xi= & {} q^{-1}\Omega \bigl (\nabla \!\!\!\!/_{\hat{{L}}}\xi +\chi ^\sharp \cdot \xi \bigr )=\Omega \bigl (\nabla \!\!\!\!/_4\xi +q^{-1}\chi ^\sharp \cdot \xi \bigr )\\ q^{-1}D\theta= & {} q^{-1}\Omega \bigl (\nabla \!\!\!\!/_{\hat{{L}}}\theta +\chi \times \theta +\theta \times \chi \bigr )=\Omega \bigl (\nabla \!\!\!\!/_4\theta +q^{-1}\chi \times \theta +q^{-1}\theta \times \chi \bigr ) \end{aligned}$$

(similarly for the conjugate equations) and thus the above replacements of the structure coefficients are consistent with the replacements

$$\begin{aligned} D\rightarrow q^{-1}D\qquad {\underline{D}}\rightarrow q{\underline{D}}\,. \end{aligned}$$

$$\square $$

### Commutations

In this section we compute the null decomposition of the Weyl field $\tilde{{\mathcal {L}}}_{N}W$. These computations are carried out using Leibniz rule for the Lie derivative

5.16 $$\begin{aligned}&({\mathcal {L}}_NW)(e_\alpha ,e_\beta ,e_\gamma ,e_\delta )=N(W(e_\alpha ,e_\beta ,e_\gamma ,e_\delta ))\nonumber \\&\quad -\,W([N,e_\alpha ],e_\beta ,e_\gamma ,e_\delta )-W(e_\alpha ,[N,e_\beta ],e_\gamma ,e_\delta )\nonumber \\&\quad -\,W(,e_\alpha ,e_\beta ,[N,e_\gamma ],e_\delta )-W(,e_\alpha ,e_\beta ,e_\gamma ,[N,e_\delta ]) \end{aligned}$$

which is also used to define ${\mathcal {L}}\!\!\!/_N{\underline{\alpha }}$, ${\mathcal {L}}\!\!\!/_N\alpha$, ${\mathcal {L}}\!\!\!/_N{\underline{\beta }}$, ${\mathcal {L}}\!\!\!/_N\beta$, for example:

5.17 $$\begin{aligned} ({\mathcal {L}}\!\!\!/_N\alpha )_{AB}=N(\alpha _{AB})-\alpha (\Pi [N,e_A],e_B)-\alpha (e_B,\Pi [N,e_B]) \end{aligned}$$

where the projection $\Pi$ to the spheres is defined in (4.65).

#### Lemma 5.8

The null components of $\tilde{{\mathcal {L}}}_{N}W$ are given by

5.18a $$\begin{aligned} \alpha _q[\tilde{{\mathcal {L}}}_{N}W]= & {} {\hat{{\mathcal {L}}\!\!\!/}}_{N} \alpha _q+\frac{\Omega }{8}\bigl (6q{\hat{{\underline{\omega }}}}+6q^{-1}{\hat{\omega }}-q{{\,\mathrm{tr}\,}}{\underline{\chi }}\nonumber \\&\quad -\,q^{-1}{{\,\mathrm{tr}\,}}\chi +7\hat{{\underline{L}}}q-\hat{{L}}q^{-1}\bigr )\alpha _q\nonumber \\&\quad -\,\Omega \bigl (2\zeta -\mathrm {d}\!\!\!/\log q\bigr ){\hat{\otimes }}\beta _q \end{aligned}$$

5.18b $$\begin{aligned} {\underline{\alpha }}_q[\tilde{{\mathcal {L}}}_{N}W]= & {} {\hat{{\mathcal {L}}\!\!\!/}}_{N} {\underline{\alpha }}_q+\frac{\Omega }{8}\bigl (6q^{-1}{\hat{\omega }}+6q{\hat{{\underline{\omega }}}}-q{{\,\mathrm{tr}\,}}{\underline{\chi }}\nonumber \\&\quad -\,q^{-1}{{\,\mathrm{tr}\,}}\chi +7\hat{{L}}q^{-1}-\hat{{\underline{L}}}q\bigr ){\underline{\alpha }}_q\nonumber \\&\quad -\,\Omega \bigl (2\zeta +\mathrm {d}\!\!\!/\log q\bigr ){\hat{\otimes }}{\underline{\beta }}_q \end{aligned}$$

5.18c $$\begin{aligned} \beta _q[\tilde{{\mathcal {L}}}_{N}W]= & {} {\mathcal {L}}\!\!\!/_{N}\beta _q-\frac{1}{2}{\hat{i}}^\sharp \cdot \beta _q+\frac{\Omega }{8} \bigl (6q{\hat{{\underline{\omega }}}}+6q^{-1}{\hat{\omega }}\nonumber \\&\quad +\,q{{\,\mathrm{tr}\,}}{\underline{\chi }}+q{{\,\mathrm{tr}\,}}\chi +\hat{{\underline{L}}}q+\hat{{L}}q\bigr )\beta _q\nonumber \\&\quad -\,\frac{3}{4}\Omega \bigl (2\zeta +\mathrm {d}\!\!\!/\log q\bigr )\rho -\frac{3}{4}\Omega \bigl (2{}^*\zeta +{}^*\mathrm {d}\!\!\!/\log q\bigr )\sigma \nonumber \\&\quad +\,\frac{1}{4}\Omega \alpha ^\sharp _q\cdot \bigl (2\zeta +\mathrm {d}\!\!\!/\log q\bigr ) \end{aligned}$$

5.18d $$\begin{aligned} {\underline{\beta }}_q[\tilde{{\mathcal {L}}}_{N}W]= & {} {\mathcal {L}}\!\!\!/_{N}{\underline{\beta }}_q-\frac{1}{2}{\hat{i}}^\sharp \cdot {\underline{\beta }}_q+\frac{\Omega }{8}\bigl (6q{\hat{{\underline{\omega }}}}+6q^{-1}{\hat{\omega }}\nonumber \\&\quad +\,q{{\,\mathrm{tr}\,}}{\underline{\chi }}+q{{\,\mathrm{tr}\,}}\chi +\hat{{\underline{L}}}q+\hat{{L}}q\bigr ){\underline{\beta }}_q\nonumber \\&\quad -\,\frac{3}{4}\Omega \bigl (2\zeta +\mathrm {d}\!\!\!/\log q\bigr )\rho +\frac{3}{4}\Omega \bigl (2{}^*\zeta +{}^*\mathrm {d}\!\!\!/\log q\bigr )\sigma \nonumber \\&\quad +\,\frac{1}{4}\Omega {\underline{\alpha }}^\sharp _q\cdot \bigl (2\zeta + \mathrm {d}\!\!\!/\log q\bigr ) \end{aligned}$$

5.18e $$\begin{aligned} \rho [\tilde{{\mathcal {L}}}_{N}W]= & {} N\rho +\frac{3}{8}\Omega \bigl (2q{\hat{{\underline{\omega }}}}+2q^{-1}{\hat{\omega }}+q{{\,\mathrm{tr}\,}}{\underline{\chi }}\nonumber \\&\quad +\,q^{-1}{{\,\mathrm{tr}\,}}\chi +\hat{{\underline{L}}}q+\hat{{L}}q^{-1}\bigr )\rho \nonumber \\&\quad +\,\frac{\Omega }{2}(2\zeta +\mathrm {d}\!\!\!/\log q,\beta _q+{\underline{\beta }}_q)\nonumber \\ \sigma [\tilde{{\mathcal {L}}}_{N}W]= & {} N\sigma +\frac{3}{8}\Omega \Bigl (2q{\hat{{\underline{\omega }}}}+2q^{-1}{\hat{\omega }}\nonumber \\&\quad +\,q{{\,\mathrm{tr}\,}}{\underline{\chi }}+q^{-1}{{\,\mathrm{tr}\,}}\chi +\hat{{\underline{L}}}q+\hat{{L}}q^{-1}\Bigr )\sigma \nonumber \\&\quad +\,\frac{\Omega }{2}\bigl (2{}^*\zeta +{}^*\mathrm {d}\!\!\!/\log q,\beta _q-{\underline{\beta }}_q\bigr ) \end{aligned}$$

#### Lemma 5.9

The commutation relations of the vectorfield *N* as given by (5.12) with the frame are

5.19a $$\begin{aligned} {[}N,e_A]= & {} \frac{\Omega }{2}\Pi [e_3+e_4,e_A]-\frac{\Omega }{2}\mathrm {d}\!\!\!/_A\log q \bigl (e_3-e_4\bigr ) \end{aligned}$$

5.19b $$\begin{aligned} {[}N,e_3]= & {} -\frac{1}{2}\bigl ({\underline{m}}-m\bigr )^{\sharp C}e_C+\Omega \mathrm {d}\!\!\!/\log q^{\sharp C}e_C+\frac{1}{4}{\underline{n}}e_4\nonumber \\&\quad -\,\frac{\Omega }{2}\bigl (q^{-1}{\hat{\omega }}+q{\hat{{\underline{\omega }}}}+\hat{{L}}q^{-1}\bigr )e_3 \end{aligned}$$

5.19c $$\begin{aligned} {[}N,e_4]= & {} \frac{1}{2}\bigl ({\underline{m}}-m\bigr )^{\sharp C}e_C-\Omega \mathrm {d}\!\!\!/\log q^{\sharp C}e_C \nonumber \\&\quad -\,\frac{\Omega }{2}\bigl (q{\hat{{\underline{\omega }}}}+\hat{{\underline{L}}}q+q^{-1}{\hat{\omega }}\bigr )e_4+\frac{1}{4}n e_3 \end{aligned}$$

#### Proof

It follows from the frame relations (4.63) that

$$\begin{aligned} {[}e_3,e_4]= & {} \nabla _3e_4-\nabla _4e_3=2\eta ^\sharp -2{\underline{\eta }}^\sharp -\bigl (q{\hat{{\underline{\omega }}}}+\hat{{\underline{L}}}q\bigr )e_4+\bigl (q^{-1}{\hat{\omega }}+\hat{{L}}q^{-1}\bigr )e_3\\ {[}e_3,e_A]= & {} \nabla \!\!\!\!/_3e_A-q{\underline{\chi }}_A^\sharp +\bigl (\mathrm {d}\!\!\!/_A\log \Omega -\mathrm {d}\!\!\!/_A\log q\bigr )e_3\\ {[}e_4,e_A]= & {} \nabla \!\!\!\!/_4e_A-q^{-1}\chi _A^\sharp +\bigl (\mathrm {d}\!\!\!/_A\log \Omega +\mathrm {d}\!\!\!/_A\log q\bigr )e_4 \end{aligned}$$

and thus

$$\begin{aligned} {[}N,e_3]= & {} -\Omega \eta ^\sharp +\Omega {\underline{\eta }}^\sharp +\frac{\Omega }{2}\hat{{\underline{L}}}q\, e_4-\frac{\Omega }{2}\bigl (q^{-1}{\hat{\omega }}+q{\hat{{\underline{\omega }}}}+\hat{{L}}q^{-1}\bigr )e_3\\ {[}N,e_4]= & {} \Omega \eta ^\sharp -\Omega {\underline{\eta }}^\sharp -\frac{\Omega }{2}\bigl (q{\hat{{\underline{\omega }}}}+\hat{{\underline{L}}}q+q^{-1}{\hat{\omega }}\bigr )e_4+\frac{\Omega }{2}\hat{{L}}q^{-1}\,e_3\\ {[}N,e_A]= & {} \frac{\Omega }{2}\bigl (\nabla \!\!\!\!/_3e_A+\nabla \!\!\!\!/_4 e_A-q{\underline{\chi }}_A^\sharp -q^{-1}\chi _A^\sharp \bigr )-\frac{\Omega }{2}\mathrm {d}\!\!\!/_A\log q\,(e_3-e_4) \end{aligned}$$

The stated formulas then follow with (5.13). $\square$

#### Proof of Lemma 5.8

Let us first compute ${\mathcal {L}}_N W(\cdot ,e_4,\cdot ,e_4)$, where the slots denoted by a $\cdot$ are to be evaluated on the frame $e_A:A=1,2$ on $S_{u,v}$. Using (5.16) we have

$$\begin{aligned}&({\mathcal {L}}_NW)(e_A,e_4,e_B,e_4)=N(W(e_A,e_4,e_B,e_4))\\&\quad -\,W([N,e_A],e_4,e_B,e_4)-W(e_A,[N,e_4],e_B,e_4)\\&\quad -\,W(,e_A,e_4,[N,e_B],e_4)-W(,e_A,e_4,e_B,[N,e_4]) \end{aligned}$$

In view of the commutation formula for $[N,e_B]$ from Lemma 5.9 we obtain

$$\begin{aligned}&N(W(e_A,e_4,e_B,e_4))-W([N,e_A],e_4,e_B,e_4)-W(,e_A,e_4,[N,e_B],e_4)\\&\quad =N((\alpha _q)_{AB})-W(\frac{\Omega }{2}\Pi [e_3+e_4,e_A],e_4,e_B,e_4)\\&\qquad -\,W(e_A,e_4,\frac{\Omega }{2}\Pi [e_3+e_4,e_B],e_4)\\&\qquad +\,\frac{\Omega }{2}\mathrm {d}\!\!\!/_A\log q \,W(e_3-e_4,e_4,e_B,e_4)+\frac{\Omega }{2}\mathrm {d}\!\!\!/_B\log q \,W(e_A,e_4,e_3-e_4,e_4)\\&\quad =({\mathcal {L}}\!\!\!/_{N}\alpha _q)_{AB}+\Omega \mathrm {d}\!\!\!/_A\log q (\beta _q)_B+\Omega \mathrm {d}\!\!\!/_B\log q(\beta _q)_A \end{aligned}$$

where in the last equality we have used the definition (5.17) and that $(\Omega /2)\Pi [e_3+e_4,e_A]=\Pi [N,e_A]$. Moreover using the commutation formula for $[N,e_4]$ from Lemma 5.9 we obtain

$$\begin{aligned}&-W(e_A,[N,e_4],e_B,e_4)-W(e_A,e_4,e_B,[N,e_4])\\&\quad =-\bigl (\frac{1}{2}({\underline{m}}-m)^\sharp -\Omega \mathrm {d}\!\!\!/\log q^{\sharp }\bigr )^C \bigl ( W(e_A,e_C,e_B,e_4)+ W(e_A,e_4,e_B,e_C)\bigr )\\&\qquad +\,\Omega \bigl (q{\hat{{\underline{\omega }}}}+\hat{{\underline{L}}}q+q^{-1}{\hat{\omega }}\bigr )W(e_A,e_4,e_B,e_4)\\&\qquad -\frac{1}{4} n W(e_A,e_3,e_B,e_4)-\frac{1}{4} n W(e_A,e_4,e_B,e_3)\\&\quad =-2\Omega \zeta ^{\sharp C}\bigl (-2g\!\!\!/_{AB}(\beta _q)_C+g\!\!\!/_{BC}\beta _A+g\!\!\!/_{AC}(\beta _q)_B\bigr ) \\&\qquad +\,\Omega \bigl (q{\hat{{\underline{\omega }}}}+\hat{{\underline{L}}}q+q^{-1}{\hat{\omega }}\bigr )(\alpha _q)_{AB}+\frac{1}{2}n \rho g\!\!\!/_{AB} \end{aligned}$$

where we have used the formula for ${\underline{m}}-m$ from Lemma 5.6 as well as the formulas (2.17) for the null components of a Weyl field. In view of the formula (1.182) in [8] for the ${\hat{\otimes }}$ product of two $S_{u,v}$ 1-forms, which we apply here in form

$$\begin{aligned} \Omega \mathrm {d}\!\!\!/_A\log q\,\beta _B+\Omega \mathrm {d}\!\!\!/_B\log q\,\beta _A=\Bigl (\mathrm {d}\!\!\!/\log q{\hat{\otimes }}\beta )_{AB}+\bigl (\Omega \mathrm {d}\!\!\!/\log q,\beta )g\!\!\!/_{AB} \end{aligned}$$

we then obtain the following formula for $({\mathcal {L}}_NW)_{A4B4}$:

$$\begin{aligned} ({\mathcal {L}}_{N}W)(\cdot ,e_4,\cdot ,e_4)= & {} {\mathcal {L}}\!\!\!/_{N}\alpha _q+\Omega \bigl (q{\hat{{\underline{\omega }}}}+q^{-1}{\hat{\omega }}+\hat{{\underline{L}}}q\bigr )\alpha _q+\frac{1}{2}n\rho g\!\!\!/\\&\quad -\,\Omega \bigl (\eta -{\underline{\eta }}-\mathrm {d}\!\!\!/\log q\bigr ){\hat{\otimes }}\beta _q+\Omega \bigl (\eta -{\underline{\eta }}-ds\log q,\beta \bigr )g\!\!\!/\end{aligned}$$

Similarly we compute:

$$\begin{aligned}&({\mathcal {L}}_{N}W)(\cdot ,e_3,\cdot ,e_3)={\mathcal {L}}\!\!\!/_{N}{\underline{\alpha }}_q+\Omega \bigl (q^{-1}{\hat{\omega }}+q{\hat{{\underline{\omega }}}}+\hat{{L}}q^{-1}\bigr ){\underline{\alpha }}_q+\frac{1}{2}{\underline{n}}\rho g\!\!\!/\\&\quad -\,\Omega \bigl (\eta -{\underline{\eta }}+\mathrm {d}\!\!\!/\log q\bigr ){\hat{\otimes }}{\underline{\beta }}+\Omega (\eta -{\underline{\eta }}+\mathrm {d}\!\!\!/\log q,{\underline{\beta }})g\!\!\!/\\&({\mathcal {L}}_{N}W)(\cdot ,e_4,e_3,e_4)=2 {\mathcal {L}}\!\!\!/_{N}\beta _q+\Omega \bigl (3q{\hat{{\underline{\omega }}}}+3q^{-1}{\hat{\omega }}+\hat{{\underline{L}}}q+\hat{{L}}q^{-1}\Bigr )\beta _q\\&\quad -\,\Omega \bigl (2\mathrm {d}\!\!\!/\log q+\eta -{\underline{\eta }}) \rho -3\Omega \,{}^*(\eta -{\underline{\eta }}+\mathrm {d}\!\!\!/\log q)\sigma \\&\quad -\,\frac{n}{2}{\underline{\beta }}_q+\Omega \alpha ^\sharp \cdot (\eta -{\underline{\eta }}+\mathrm {d}\!\!\!/\log q)\\&({\mathcal {L}}_{N}W)(\cdot ,e_3,e_3,e_4)=2 {\mathcal {L}}\!\!\!/_{N}{\underline{\beta }}_q+\Omega \bigl (3q{\hat{{\underline{\omega }}}}+3q^{-1}{\hat{\omega }}+\hat{{\underline{L}}}q+\hat{{L}}q^{-1}\Bigr ){\underline{\beta }}_q\\&\quad -\,\Omega \bigl (\mathrm {d}\!\!\!/\log q+\eta -{\underline{\eta }}) \rho +3\Omega \,{}^*(\eta -{\underline{\eta }}+\mathrm {d}\!\!\!/\log q)\sigma -\frac{{\underline{n}}}{2}\beta _q\\&\quad +\,\Omega {\underline{\alpha }}^\sharp \cdot (\eta -{\underline{\eta }}+\mathrm {d}\!\!\!/\log q)\\&({\mathcal {L}}_{N}W)(e_3,e_4,e_3,e_4)=4{\mathcal {L}}_{N}\rho +4\Omega \bigl (2q{\hat{{\underline{\omega }}}}+2q^{-1}{\hat{\omega }}+\hat{{\underline{L}}}q+\hat{{L}}q^{-1}\bigr )\rho \\&\quad +\,4\Omega (\eta -{\underline{\eta }}+\mathrm {d}\!\!\!/\log q)^\sharp \cdot \beta _q+4\Omega (\eta -{\underline{\eta }}+\mathrm {d}\!\!\!/\log q)^\sharp \cdot {\underline{\beta }}_q\\&\epsilon \!\!/^{AB}{\mathcal {L}}_{N}W_{AB34}=4N \sigma +2\Omega \Bigl ( 2q{\hat{{\underline{\omega }}}}+2q^{-1}{\hat{\omega }}+q{{\,\mathrm{tr}\,}}{\underline{\chi }}+q^{-1}{{\,\mathrm{tr}\,}}\chi +\hat{{\underline{L}}}q+\hat{{L}}q^{-1}\Bigr )\sigma \\&\quad +\,2\Omega \bigl ( \mathrm {d}\!\!\!/\log q +\eta -{\underline{\eta }},{}^*{\underline{\beta }}_q\bigr )-2\Omega \bigl ( \mathrm {d}\!\!\!/\log q +\eta -{\underline{\eta }},{}^*\beta _q\bigr ) \end{aligned}$$

In the last formula we also used (12.46) in [8].

We adopt the notation of (12.48) in [8] for the terms appearing in the second line of the formula (5.5) for the modified Lie derivative, namely we write

$$\begin{aligned} (\tilde{{\mathcal {L}}}_{N}W)_{\alpha \beta \gamma \delta }=({\mathcal {L}}_{N}W)_{\alpha \beta \gamma \delta }-\frac{1}{2}{}^{(N)}[W]_{\alpha \beta \gamma \delta }-\frac{1}{8}{{\,\mathrm{tr}\,}}{}^{(N)}\pi \,W_{\alpha \beta \gamma \delta } \end{aligned}$$

where

$$\begin{aligned} {}^{(N)}[W]_{\alpha \beta \gamma \delta }={}^{(N)}{\hat{\pi }}_{\alpha }^{\mu }W_{\mu \beta \gamma \delta }+{}^{(N)}{\hat{\pi }}_{\beta }^{\mu }W_{\alpha \mu \gamma \delta }+{}^{(N)}{\hat{\pi }}_{\gamma }^{\mu }W_{\alpha \beta \mu \delta }+{}^{(N)}{\hat{\pi }}_{\delta }^{\mu }W_{\alpha \beta \gamma \mu }\,. \end{aligned}$$

We then proceed similarly to (12.49) in [8] and find that:

$$\begin{aligned} {}^{(N)}[W]_{A4B4}&=({\hat{i}},\alpha _q)g\!\!\!/_{AB}-2 m^C(\beta _q)_Cg\!\!\!/_{AB}+n\rho g\!\!\!/_{AB}\\ {}^{(N)}[W]_{A3B3}&=({\hat{i}},{\underline{\alpha }}_q)g\!\!\!/_{AB}+2{\underline{m}}^C({\underline{\beta }}_q)_Cg\!\!\!/_{AB}+{\underline{n}}\rho g\!\!\!/_{AB}\\ {}^{(N)}[W]_{A434}&=-m_A\rho +3{}^*m_A\sigma +2{\hat{i}}_A^{\sharp B}(\beta _q)_B-\\&\quad 2j(\beta _q)_A-n({\underline{\beta }}_q)_A+(\alpha _q)_A^{\sharp B}{\underline{m}}_B\\ {}^{(N)}[W]_{A334}&={\underline{m}}_A\rho +3{}^*{\underline{m}}_A\sigma +2{\hat{i}}_A^{\sharp B}({\underline{\beta }}_q)_B\\&\quad -2j{\underline{\beta }}_A-{\underline{n}}(\beta _q)_A-({\underline{\alpha }}_q)_A^{\sharp B}\cdot m_B\\ {}^{(N)}[W]_{3434}&=-8j\rho +4{\underline{m}}^\sharp \cdot \beta _q-4m^\sharp \cdot {\underline{\beta }}_q\\ \epsilon \!\!/^{AB}{}^{(N)}[W]_{AB34}&=0 \end{aligned}$$

The formulas given in the statement of the Lemma then follow. $\square$

### Weyl Currents

The components of the first order Weyl current

5.21 $$\begin{aligned} {}^{(X)}J^1(W)_{\beta \gamma \delta }=\frac{1}{2}{}^{(X)}{\hat{\pi }}^{\mu \nu }\nabla _\nu W_{\mu \beta \gamma \delta } \end{aligned}$$

have been calculated for a general commutation vectorfield *X*, and are presented *in the special case* $n={\underline{n}}=0$, and *relative to the frame* $(\hat{{\underline{L}}},\hat{{L}};e_A)$ in Lemma 14.1 in [8]. Here and in the following Lemma *i*, ${\underline{m}}$, *m*, ${\underline{n}}$, *n*, and *j* refer to the null decomposition of the deformation tensor ${}^{(X)}{\hat{\pi }}$ of a general commutation vectorfield *X* as given in (5.11) in the case $X=N$. Note that ${{\,\mathrm{tr}\,}}i=j$ holds generally because ${{\,\mathrm{tr}\,}}{\hat{\pi }}=0$.

In the following Lemma we list the formulas for the components of ${}^{(X)}J^1(W)$ in the general case, $n\ne {\underline{n}}\ne 0$, decomposed relative to the frame $(e_3,e_4;e_A)$. These formulas can be inferred from the expressions in Lemma 14.1 in [8] using the replacements (4.67).

#### Lemma 5.10

The null components of the Weyl current $J^1(W)$ relative to the frame $(e_3,e_4;e_A)$ are given by

5.22a $$\begin{aligned} \begin{aligned} 4\Xi ^1_A&= -\frac{1}{2}j\Bigl \{ q^{-1}\Omega ^{-1}( D\beta _q)_A+({{\,\mathrm{div}\,}}\!\!\!\!\!/\,\alpha _q)_A -q^{-1}\chi _A^B(\beta _q)_B\\&\qquad \qquad \quad -q^{-1}{{\,\mathrm{tr}\,}}\chi (\beta _q)_A-\bigl (q^{-1}{\hat{\omega }}+\frac{1}{\Omega }D q^{-1}\bigr )(\beta _q)_A\\&\qquad \qquad \quad +\alpha _A^B\bigl (2\zeta _B+2\mathrm {d}\!\!\!/_B\log q-{\underline{\eta }}_B\bigr )\Bigr \}\\&\quad +\frac{1}{2}m^B\Bigl \{2\nabla \!\!\!\!/_B(\beta _q)_A+q\Omega ^{-1}({\hat{{\underline{D}}}}\alpha _q)_{AB}-q{\underline{\chi }}_B^C(\alpha _q)_{AC}-q{{\,\mathrm{tr}\,}}{\underline{\chi }}(\alpha _q)_{AB}\\&\qquad \qquad \qquad +2\bigl (q{\hat{{\underline{\omega }}}}+\frac{1}{\Omega }{\underline{D}}q\bigr )(\alpha _q)_{AB}\\&\qquad \qquad \qquad +2\bigl ((\zeta +\mathrm {d}\!\!\!/\log q-2\eta ){\hat{\otimes }}\beta _q\bigr )_{AB}-3q^{-1}\bigl (\chi _{AB}\rho +{}^*\chi _{AB}\sigma \bigr )\Bigr \}\\&\quad +\frac{1}{2}{\underline{m}}^B\Bigl \{q^{-1}\Omega ^{-1}({\hat{D}}\alpha _q)_{AB}-q^{-1}{{\,\mathrm{tr}\,}}\chi (\alpha _q)_{AB}-2\bigl (q^{-1}{\hat{\omega }}+\frac{1}{\Omega }D q^{-1}\bigr )(\alpha _q)_{AB}\Bigr \}\\&\quad -{\hat{i}}^{BC}\Bigl \{\nabla \!\!\!\!/_C(\alpha _q)_{AB}-q^{-1}(\chi {\hat{\otimes }}\beta _q)_{CAB}+2\bigl (\zeta +\mathrm {d}\!\!\!/\log q\bigr )_C(\alpha _q)_{AB}\Bigr \}\\&\quad +\frac{1}{2}n\Bigl \{-q\Omega ^{-1}({\underline{D}}\beta _q)_A+q{\underline{\chi }}_A^B(\beta _q)_B-\bigl (q{\hat{{\underline{\omega }}}}+\frac{1}{\Omega }{\underline{D}}q\bigr )(\beta _q)_A+3\eta _A\rho +3{}^*\eta _A\sigma \Bigr \} \end{aligned} \end{aligned}$$

Moreover

5.22b $$\begin{aligned} 4{\underline{\Xi }}^1= & {} *+\frac{1}{2}{\underline{n}}\Bigl \{q^{-1}\Omega ^{-1}D{\underline{\beta }}_q-q^{-1}\chi ^\sharp \cdot {\underline{\beta }}_q \nonumber \\&\quad +\,\bigl (q^{-1}{\hat{\omega }}+\frac{1}{\Omega }D q^{-1}\bigr ){\underline{\beta }}_q+3{\underline{\eta }}\rho -3{}^*{\underline{\eta }}\sigma \Bigr \} \end{aligned}$$

5.22c $$\begin{aligned} 4{\underline{\Lambda }}^1= & {} *+\frac{1}{2}{\underline{n}}\Bigl \{q^{-1}\Omega ^{-1}D\rho -2{\underline{\eta }}^\sharp \cdot \beta _q\Bigr \} \end{aligned}$$

5.22d $$\begin{aligned} 4\Lambda ^1= & {} *+\frac{1}{2}n\Bigl \{q\Omega ^{-1}{\underline{D}}\rho +2\eta ^\sharp \cdot {\underline{\beta }}_q\Bigr \} \end{aligned}$$

5.22e $$\begin{aligned} 4{\underline{K}}^1= & {} *+\frac{1}{2}{\underline{n}}\Bigl \{-q^{-1}\Omega ^{-1}D\sigma -2{\underline{\eta }}\wedge \beta _q\Bigr \} \end{aligned}$$

5.22f $$\begin{aligned} 4K^1= & {} *+\frac{1}{2}n\Bigl \{q\Omega ^{-1}{\underline{D}}\sigma +2\eta \wedge {\underline{\beta }}_q\Bigr \} \end{aligned}$$

5.22g $$\begin{aligned} 4{\underline{I}}^1= & {} *+\frac{1}{2}n\Bigl \{-q\Omega ^{-1}{\underline{D}}{\underline{\beta }}_q+q{\underline{\chi }}^\sharp \cdot {\underline{\beta }}_q\nonumber \\&\quad +\,\bigl (q{\hat{{\underline{\omega }}}}+\frac{1}{\Omega }{\underline{D}}q\bigr ){\underline{\beta }}_q-\eta ^\sharp \cdot {\underline{\alpha }}_q\Bigr \} \end{aligned}$$

5.22h $$\begin{aligned} 4I^1= & {} *+\frac{1}{2}{\underline{n}}\Bigl \{q^{-1}\Omega ^{-1}D\beta _q-q^{-1}\chi ^\sharp \cdot \beta _q\nonumber \\&\quad -\,\bigl (q^{-1}{\hat{\omega }}+\frac{1}{\Omega }D q^{-1}\bigr )\beta _q-{\underline{\eta }}^\sharp \cdot \alpha _q\Bigr \}\ \end{aligned}$$

5.22i $$\begin{aligned} 4{\underline{\Theta }}^1= & {} *+\frac{1}{2}n\Bigl \{q\Omega ^{-1}{\hat{{\underline{D}}}}{\underline{\alpha }}_q-q{{\,\mathrm{tr}\,}}{\underline{\chi }}{\underline{\alpha }}_q\nonumber \\&\quad -\,2\bigl ( q{\hat{{\underline{\omega }}}}+\frac{1}{\Omega }{\underline{D}}q\bigr ){\underline{\alpha }}_q\Bigr \} \end{aligned}$$

5.22j $$\begin{aligned} 4\Theta ^1= & {} *+\frac{1}{2}{\underline{n}}\Bigl \{q^{-1}\Omega ^{-1}{\hat{D}}\alpha _q-q^{-1}{{\,\mathrm{tr}\,}}\chi \alpha _q\nonumber \\&\quad -\,2\bigl (q^{-1}{\hat{\omega }}+\frac{1}{\Omega }D q^{-1}\bigr )\alpha _q\Bigr \} \end{aligned}$$

where $*$ denotes the corresponding terms listed in Lemma 14.1 in [8] (pages 446-451) with the formal replacements of (5.15).

#### Proof

Consider for example

$$\begin{aligned} {\underline{\Lambda }}^1=\frac{1}{4} J_{343}^1(W)=\frac{1}{2}\frac{1}{4}{\hat{\pi }}^{\mu \nu }\nabla _\nu W_{\mu 343} \end{aligned}$$

Let us write out the terms which have *j* as a common factor. This can arise either from $(\mu \nu )=(43)$, or $(\mu \nu )=(AB)$, because ${{\,\mathrm{tr}\,}}i=j$. Indeed

$$\begin{aligned} \frac{1}{2}{\hat{\pi }}^{43}\nabla _3 W_{4 343}=\frac{1}{2}\frac{1}{4}j\Bigl [ e_3\bigl ( 4\rho \bigr )-2W(\nabla _3e_4,e_3,e_4,e_3)-2W(e_4,\nabla _3e_3,e_4,e_3) \Bigr ] \end{aligned}$$

Now inserting the frame relations (4.63) (with $a=q$) we see that these differ only in the coefficients from those used in (1.175) in [8], and can be obtained from the latter using the replacements (4.67) with ($a=q$). For definiteness,

$$\begin{aligned} -2W(\nabla _3e_4,e_3,e_4,e_3)= & {} 8\eta ^\sharp \cdot {\underline{\beta }}_q+8\bigl (q{\hat{{\underline{\omega }}}}-q^2\hat{{\underline{L}}}q^{-1}\bigr )\rho \\ -2W(e_4,\nabla _3e_3,e_4,e_3)= & {} -8\bigl (q{\hat{{\underline{\omega }}}}+\hat{{\underline{L}}}q\bigr )\rho \end{aligned}$$

and thus

$$\begin{aligned} \frac{1}{2}{\hat{\pi }}^{43}\nabla _3 W_{4 343}=\frac{1}{2}j\Bigl [ q \Omega ^{-1} {\underline{D}}\rho +2\eta ^\sharp \cdot {\underline{\beta }}_q\Bigr ] \end{aligned}$$

Furthermore we have the following contribution:

$$\begin{aligned} \frac{1}{2}{\hat{\pi }}^{AB}\nabla _B W_{A 343}=\frac{1}{2}{\hat{i}}^{AB}\nabla _BW_{A343}+\frac{1}{4} j g^{AB}\nabla _B W_{A343} \end{aligned}$$

while

$$\begin{aligned} g^{AB}\nabla _B W_{A343}= & {} -g^{AB}e_B\bigl ( 2{\underline{\beta }}_A\bigr )-g^{AB}W(\nabla _Be_A,e_3,e_4,e_3)\\&\quad -\,g^{AB}W(e_A,\nabla _Be_3,e_4,e_3)-\,g^{AB}W(e_A,e_3,\nabla _Be_4,e_3)\\&\quad -\,g^{AB}W(e_A,e_3,e_4,\nabla _B e_3) \end{aligned}$$

and again inserting the frame relations (4.63) (with $a=q$) gives the same result as inserting (1.175) in [8] followed by the replacements (4.67). For definiteness,

$$\begin{aligned} -g^{AB}W(\nabla _B e_A,e_3,e_4,e_3)= & {} +2g\!\!\!/^{AB}\nabla \!\!\!\!/_B e_A^C({\underline{\beta }}_q)_C-2 q{{\,\mathrm{tr}\,}}{\underline{\chi }}\rho \\ -g^{AB}W(e_A,\nabla _Be_3,e_4,e_3)= & {} 2qg\!\!\!/^{AB}{\underline{\chi }}_B^{\sharp C}\sigma \epsilon \!\!/_{AC}\\&\quad +\,2g\!\!\!/^{AB}\bigl (\zeta _B+\mathrm {d}\!\!\!/_B\log q\bigr )({\underline{\beta }}_q)_A\\ -g^{AB}W(e_A,e_3,\nabla _Be_4,e_3)= & {} -q^{-1}g\!\!\!/^{AB}\chi _B^{\sharp C}({\underline{\alpha }}_q)_{AC}\\&\quad -\,2g\!\!\!/^{AB}\bigl (\zeta _B+\mathrm {d}\!\!\!/_B\log q\bigr )({\underline{\beta }}_q)_A\\ -g^{AB}W(e_A,e_3,e_4,\nabla _B e_3)= & {} qg\!\!\!/^{AB}{\underline{\chi }}_B^{\sharp C}\bigl (-\rho g\!\!\!/_{AC}+\sigma \epsilon \!\!/_{AC}\bigr )\\&\quad +\,2g\!\!\!/^{AB}\bigl (\zeta _B+\mathrm {d}\!\!\!/_B\log q\bigr )({\underline{\beta }}_q)_A \end{aligned}$$

and thus

$$\begin{aligned} g^{AB}\nabla _B W_{A343}= & {} -2g\!\!\!/^{AB}\nabla \!\!\!\!/_B({\underline{\beta }}_q)_A-3 q{{\,\mathrm{tr}\,}}{\underline{\chi }}\rho -q^{-1}({\hat{\chi }},{\underline{\alpha }}_q)\\&\quad +\,3q{{\,\mathrm{tr}\,}}{}^*{\underline{\chi }}\sigma +2\bigl (\zeta +\mathrm {d}\!\!\!/\log q,{\underline{\beta }}_q\bigr ) \end{aligned}$$

In conclusion, we have calculated that the terms in ${\underline{\Lambda }}$ *which come with a factor j* are given by

$$\begin{aligned} 4 {\underline{\Lambda }}^1\doteq \frac{1}{2}j\Bigl [ q \Omega ^{-1} {\underline{D}}\rho \!-\!\frac{3}{2} q{{\,\mathrm{tr}\,}}{\underline{\chi }}\rho +\bigl (\zeta +2\eta +\mathrm {d}\!\!\!/\log q,{\underline{\beta }}_q\bigr ) \!-\!{{\,\mathrm{div}\,}}\!\!\!\!\!/\,{\underline{\beta }}_q\!-\!\frac{1}{2}q^{-1}({\hat{\chi }},{\underline{\alpha }}_q)\Bigr ] \end{aligned}$$

This coincides precisely with the formula given in Lemma 14.1 in [8] (pages 448-449) modulo the replacements indicated in the statement of this Lemma.

Similarly for all other components. $\square$

### Positive Current

Finally in this section we will analyse in all detail the terms appearing in (5.7) for the commutation vectorfield *N* defined in (5.4). As explained in Section 1.2, and as it is clear from (5.9), a quantitative bound on ${{\,\mathrm{div}\,}}Q[\tilde{{\mathcal {L}}}_{N}W]$ can only be obtained under additional assumptions on $\nabla {}^{(N)}\pi$.

In addition to (${{\varvec{BA:I}}.i-vi}$) we assume 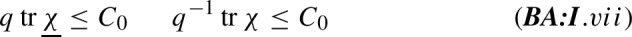

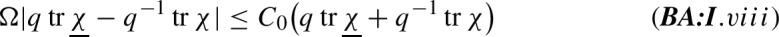
 and to deal with several “borderline” terms we strengthen (${{\varvec{BA:I}}.iv}$) and (${{\varvec{BA:I}}.vi}$) to 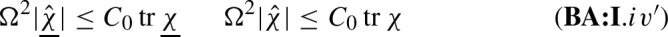

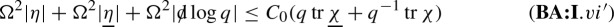
 Furthermore we assume 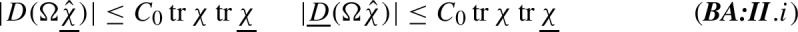

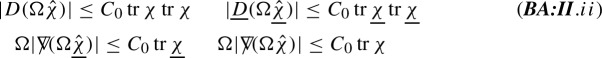


and ($${{\varvec{BA:II}}.iii-viii}$$) below.

The main conclusion is that under these assumptions the divergence (5.7) is *positive* up to a sufficiently fast decaying error:

#### Proposition 5.11

Assume (**BA:I**) — including (${{\textbf {BA:I}}.iv}^\prime$) and (${{\textbf {BA:I}}.vi}^\prime$) — and (**BA:II**) hold for some $C_0>0$. Then there is a constant $C>0$, such that for all solutions *W* to (1.27),

5.23 $$\begin{aligned}&\phi \bigl ({{\,\mathrm{div}\,}}Q(\tilde{{\mathcal {L}}}_{N}W)\bigr )(M_q,M_q,M_q) \ge \nonumber \\&\quad \ge -\frac{C}{r}\frac{C_0}{\Omega }\Bigl [Q[\tilde{{\mathcal {L}}}_{N}W](n,M_q,M_q,M_q)+Q[W](n,M_q,M_q,M_q)\Bigr ] -\frac{C}{r}\frac{C_0}{\Omega }\Omega ^2{P\!\!\!\!/\,}^q\nonumber \\ \end{aligned}$$

where

5.24 $$\begin{aligned} {P\!\!\!\!/\,}^q:=\frac{1}{(2\Omega )^3}\Bigl [|\nabla \!\!\!\!/{\underline{\alpha }}_q |^2 + |\nabla \!\!\!\!/{\underline{\beta }}_q |^2 + |\nabla \!\!\!\!/\rho |^2 + |\nabla \!\!\!\!/\sigma |^2 + |\nabla \!\!\!\!/\beta _q|^2 +|\nabla \!\!\!\!/\alpha _q|^2\Bigr ]\nonumber \\ \end{aligned}$$

We will give the proof of this Proposition in Sections 5.3.1-5.3.3.

#### $J^1$

##### Lemma 5.12

Assume that (**BA:I**.i-iii,v,vii,viii) hold, i.e. for some $C_0>0$,

$$\begin{aligned}&{{\,\mathrm{tr}\,}}\chi>0\qquad {{\,\mathrm{tr}\,}}{\underline{\chi }}>0 \\&\quad |2\omega -\Omega {{\,\mathrm{tr}\,}}\chi |\le C_0 {{\,\mathrm{tr}\,}}\chi \qquad |2{\underline{\omega }}-\Omega {{\,\mathrm{tr}\,}}{\underline{\chi }}|\le C_0 {{\,\mathrm{tr}\,}}{\underline{\chi }}\\&\quad |\Omega {{\,\mathrm{tr}\,}}\chi -\overline{\Omega {{\,\mathrm{tr}\,}}\chi }|\le C_0\Omega ^{-1}\,\overline{\Omega {{\,\mathrm{tr}\,}}\chi }\qquad |\Omega {{\,\mathrm{tr}\,}}{\underline{\chi }}-\overline{\Omega {{\,\mathrm{tr}\,}}{\underline{\chi }}}|\le C_0\Omega ^{-1}\,\overline{\Omega {{\,\mathrm{tr}\,}}{\underline{\chi }}} \\&\quad |D \log q|\le C_0 {{\,\mathrm{tr}\,}}\chi \qquad |{\underline{D}}\log q |\le C_0 {{\,\mathrm{tr}\,}}{\underline{\chi }}\\&\quad q{{\,\mathrm{tr}\,}}{\underline{\chi }}\le C_0\qquad q^{-1}{{\,\mathrm{tr}\,}}\chi \le C_0\\&\quad \Omega |q{{\,\mathrm{tr}\,}}{\underline{\chi }}-q^{-1}{{\,\mathrm{tr}\,}}\chi |\le C_0 \bigl ( q{{\,\mathrm{tr}\,}}{\underline{\chi }}+q^{-1}{{\,\mathrm{tr}\,}}\chi \bigr ) \end{aligned}$$

and moreover that (**BA:I**.iv,vi) hold,^42^ i.e. for some $C_0>0$,

$$\begin{aligned}&\Omega |{\hat{{\underline{\chi }}}}|\le C_0 {{\,\mathrm{tr}\,}}{\underline{\chi }}\qquad \Omega |{\hat{\chi }}|\le C_0 {{\,\mathrm{tr}\,}}\chi \\&\quad \Omega |\eta |+ \Omega |{\underline{\eta }}|+ \Omega |\mathrm {d}\!\!\!/\log q|\le C_0 \bigl ( q{{\,\mathrm{tr}\,}}{\underline{\chi }}+q^{-1}{{\,\mathrm{tr}\,}}\chi \bigr )\,. \end{aligned}$$

Then,

5.27 $$\begin{aligned}&\phi \Bigl [ \bigl ({{\,\mathrm{div}\,}}Q(\tilde{{\mathcal {L}}}_{N}W)\bigr )(M_q,M_q,M_q) \Bigr ]^1\ge \frac{\phi }{(2\Omega )^3}\bigl [X^1+Y^1\bigr ]\nonumber \\&\quad -\,\frac{C}{r}\frac{1}{\Omega }Q[\tilde{{\mathcal {L}}}_{N}W](n,M_q,M_q,M_q) -\frac{C}{r}\frac{1}{\Omega }Q[W](n,M_q,M_q,M_q) \nonumber \\&\quad -\frac{C}{r}\frac{1}{\Omega }\Bigl [\Omega ^2{P\!\!\!\!/\,}^q\Bigr ] \end{aligned}$$

where

5.28a $$\begin{aligned}&X^1:=X_\Lambda ^1+X_K^1+X_{{\underline{\Xi }}}^1+X_{\Xi }^1+X_{{\underline{\Theta }}}^1+X_{\Theta }^1 \end{aligned}$$

5.28b $$\begin{aligned}&Y^1:=X_\Lambda ^1+Y_K^1+Y_{{\underline{\Xi }}}^1+Y_{\Xi }^1+Y_{{\underline{\Theta }}}^1+Y_{\Theta }^1 \end{aligned}$$

and $X_\Lambda ^1$, $X_K^1$, $X_{{\underline{\Xi }}}^1$, $X_{\Xi }^1$, $X_{{\underline{\Theta }}}^1$, $X_{\Theta }^1$, are given by (5.31e), (5.41b), (5.42b), (5.43b), (5.44b), (5.45b), respectively, while $Y_\Lambda ^1$, $Y_K^1$, $Y_{{\underline{\Xi }}}^1$, $Y_{\Xi }^1$, $Y_{{\underline{\Theta }}}^1$, $Y_{\Theta }^1$, are given by (5.31f), (5.41d), (5.42d), (5.43d), (5.44d), (5.45d), respectively.

##### Proof

We use Lemma 5.10, and Lemma 14.1 in [8] as discussed above, to write out the null components of the current ${}^{(N)}J^1(W)$.

We begin with

$$\begin{aligned} \tilde{{\underline{\Lambda }}}_q^1 := \frac{1}{4}{}^{(N)}J^1[W](e_3,e_4,e_3)\qquad {\tilde{\Lambda }}_q^1 := \frac{1}{4}{}^{(N)}J^1[W](e_4,e_3,e_4) \end{aligned}$$

In the first place we are only interested in terms whose factors are either *j*, or *n*, ${\underline{n}}$, and ignore all other terms with factors *m*, ${\underline{m}}$, and ${\hat{i}}$; thus in the following formula we set

5.29a $$\begin{aligned} m=0\,,\qquad {\underline{m}}=0\,,\qquad {\hat{i}}=0\,. \end{aligned}$$

By Lemma 5.10,

5.29b $$\begin{aligned}&8{\tilde{\Lambda }}^1_q+8\tilde{{\underline{\Lambda }}}^1_q=j\Bigl \{q^{-1}\Omega ^{-1}D\rho +{{\,\mathrm{div}\,}}\!\!\!\!\!/\,\beta _q-\frac{3}{2}q^{-1}{{\,\mathrm{tr}\,}}\chi \rho \nonumber \\&\quad +\,(\zeta +\mathrm {d}\!\!\!/\log q-2{\underline{\eta }},\beta _q)-\frac{1}{2}q({\hat{{\underline{\chi }}}},\alpha _q)\nonumber \\&\quad +\,q\Omega ^{-1}{\underline{D}}\rho -{{\,\mathrm{div}\,}}\!\!\!\!\!/\,{\underline{\beta }}_q-\frac{3}{2}q{{\,\mathrm{tr}\,}}{\underline{\chi }}\rho +(\zeta +\mathrm {d}\!\!\!/\log q+2\eta ,{\underline{\beta }}_q)-\frac{1}{2}q^{-1}({\hat{\chi }},{\underline{\alpha }}_q)\Bigr \}\nonumber \\&\quad +\,{\underline{n}}\Bigl \{q^{-1}\Omega ^{-1}D\rho -2{\underline{\eta }}^\sharp \cdot \beta _q\Bigr \} +n\Bigl \{q\Omega ^{-1}{\underline{D}}\rho +2\eta ^\sharp \cdot {\underline{\beta }}_q\Bigr \}\nonumber \\&\quad =j\Bigl \{\frac{2}{\Omega }\bigl (q^{-1}D\rho +q{\underline{D}}\rho \bigr )-4({\underline{\eta }},\beta _q)+4(\eta ,{\underline{\beta }}_q)\Bigr \} \nonumber \\&\quad +\,{\underline{n}}\Bigl \{q^{-1}\Omega ^{-1}D\rho -2{\underline{\eta }}^\sharp \cdot \beta _q\Bigr \} +n\Bigl \{q\Omega ^{-1}{\underline{D}}\rho +2\eta ^\sharp \cdot {\underline{\beta }}_q\Bigr \} \end{aligned}$$

where we have used the Bianchi equations in the form of Prop. 5.7. We symmetrize the terms with coefficients in ${\underline{n}}$, and *n*, and use commutation Lemma 5.8 to obtain:

5.29c $$\begin{aligned}&8{\tilde{\Lambda }}^1_q+8\tilde{{\underline{\Lambda }}}^1_q=\Bigl (j+\frac{{\underline{n}}+n}{4}\Bigr )\Bigl \{\frac{2}{\Omega }\bigl (q^{-1}D\rho +q{\underline{D}}\rho \bigr )-4({\underline{\eta }},\beta _q)+4(\eta ,{\underline{\beta }}_q)\Bigr \} \nonumber \\&\quad -\frac{{\underline{n}}-n}{2}\Bigl \{q^{-1}\Omega ^{-1}D\rho -2{\underline{\eta }}^\sharp \cdot \beta _q-q\Omega ^{-1}{\underline{D}}\rho -2\eta ^\sharp \cdot {\underline{\beta }}_q\Bigr \}\nonumber \\&\quad =\Bigl (j+\frac{n+{\underline{n}}}{4}\Bigr )\Bigl \{\frac{4}{\Omega }{\tilde{\rho }}-\mathbf {\frac{3}{2}}\bigl (2q{\hat{{\underline{\omega }}}}+2q^{-1}{\hat{\omega }}+q{{\,\mathrm{tr}\,}}{\underline{\chi }}+q^{-1}{{\,\mathrm{tr}\,}}\chi +\hat{{\underline{L}}}q+\hat{{L}}q^{-1}\bigr )\rho \nonumber \\&\quad -\,2(\eta +{\underline{\eta }}+\mathrm {d}\!\!\!/\log q,\beta _q)+2(\eta +{\underline{\eta }}-\mathrm {d}\!\!\!/\log q,{\underline{\beta }}_q)\Bigr \}\nonumber \\&\quad -\,\frac{{\underline{n}}-n}{2}\Bigl \{-\frac{3}{2}\bigl (q^{-1}{{\,\mathrm{tr}\,}}\chi -q{{\,\mathrm{tr}\,}}{\underline{\chi }}\bigr )\rho +{{\,\mathrm{div}\,}}\!\!\!\!\!/\,\beta _q+{{\,\mathrm{div}\,}}\!\!\!\!\!/\,{\underline{\beta }}_q\nonumber \\&\quad +\,(\zeta +\mathrm {d}\!\!\!/\log q,\beta )-(\zeta +\mathrm {d}\!\!\!/\log q,{\underline{\beta }})-\frac{1}{2}q({\hat{{\underline{\chi }}}},\alpha _q)+\frac{1}{2}q^{-1}({\hat{\chi }},{\underline{\alpha }}_q)\Bigr \} \end{aligned}$$

In this symmetrization, we have on one hand gained that the sum yields an additional *positive* term, while on the other hand by the Bianchi equations of Prop. 5.7 the difference leaves us with terms only involving *angular* derivatives:

5.29d $$\begin{aligned}&q^{-1}\Omega ^{-1}D\rho -2{\underline{\eta }}^\sharp \cdot \beta _q-q\Omega ^{-1}{\underline{D}}\rho -2\eta ^\sharp \cdot {\underline{\beta }}_q=\nonumber \\&\quad =-\frac{3}{2}\bigl (q^{-1}{{\,\mathrm{tr}\,}}\chi -q{{\,\mathrm{tr}\,}}{\underline{\chi }}\bigr )\rho +{{\,\mathrm{div}\,}}\!\!\!\!\!/\,\beta _q+{{\,\mathrm{div}\,}}\!\!\!\!\!/\,{\underline{\beta }}_q\nonumber \\&\qquad +\,(\zeta +\mathrm {d}\!\!\!/\log q,\beta )-(\zeta +\mathrm {d}\!\!\!/\log q,{\underline{\beta }})-\frac{1}{2}q({\hat{{\underline{\chi }}}},\alpha _q)+\frac{1}{2}q^{-1}({\hat{\chi }},{\underline{\alpha }}_q)\qquad \qquad \end{aligned}$$

##### Remark 5.13

With $M_q$ as commutator the prefactor that appears in bold in (5.29c) would be different, and this term would fail to cancel with similar terms appearing in ${\tilde{\Lambda }}^2+\tilde{{\underline{\Lambda }}}^2$ discussed below. The cancellations that *do* occur with *N* — defined by (5.4) and expressed as in (5.12) — as a commutator are discussed in Section 5.3.3.

In the second instance we consider all terms with factors *m*, ${\underline{m}}$, and ${\hat{i}}$, and set

5.30a $$\begin{aligned} j=0\,,\qquad n=0\,,\qquad {\underline{n}}=0\,, \end{aligned}$$

in the following formula:

5.30b $$\begin{aligned} 8{\tilde{\Lambda }}^1= & {} -\Bigl (m,\Omega ^{-1}q{\underline{D}}\beta _q-q{\underline{\chi }}^\sharp \cdot \beta +\bigl (q{\hat{{\underline{\omega }}}}+\Omega ^{-1}{\underline{D}}q\bigr )\beta _q-3\eta \rho -3{}^*\eta \sigma \Bigr )\nonumber \\&\quad -\,2\Bigl (m,\mathrm {d}\!\!\!/\rho +q^{-1}\chi ^\sharp \cdot {\underline{\beta }}_q-q{\underline{\chi }}^\sharp \cdot \beta _q\Bigr )\nonumber \\&\quad -\,2\Bigl ({\underline{m}},\Omega ^{-1}q^{-1}D\beta _q-q^{-1}\chi ^\sharp \cdot \beta _q-\bigl (q^{-1}{\hat{\omega }}+\Omega ^{-1}D q^{-1}\bigr )\beta _q-{\underline{\eta }}^\sharp \cdot \alpha _q\Bigr )\nonumber \\&\quad +\,\Bigl ({\hat{i}},\nabla \!\!\!\!/{\hat{\otimes }}\beta _q+\bigl (\zeta +\mathrm {d}\!\!\!/\log q\bigr ){\hat{\otimes }}\beta _q-3\bigl (q^{-1}{\hat{\chi }}\rho +q^{-1}{}^*{\hat{\chi }}\sigma \bigr )-\frac{1}{2}q{{\,\mathrm{tr}\,}}{\underline{\chi }}\alpha _q\Bigr )\nonumber \\= & {} -\Bigl (m,-2q{{\,\mathrm{tr}\,}}{\underline{\chi }}\beta _q+q^{-1}{{\,\mathrm{tr}\,}}\chi {\underline{\beta }}_q+3\mathrm {d}\!\!\!/\rho -{}^*\mathrm {d}\!\!\!/\sigma +4q^{-1}{\hat{\chi }}^\sharp \cdot {\underline{\beta }}_q-2q{\hat{{\underline{\chi }}}}^\sharp \cdot \beta _q\Bigr )\nonumber \\&\quad -\,2\Bigl ({\underline{m}},-2q^{-1}{{\,\mathrm{tr}\,}}\chi \beta _q+{{\,\mathrm{div}\,}}\!\!\!\!\!/\,\alpha _q+2\bigl (\zeta +\mathrm {d}\!\!\!/\log q\bigr )^\sharp \cdot \alpha _q\Bigr )\nonumber \\&\quad +\,\Bigl ({\hat{i}},\nabla \!\!\!\!/{\hat{\otimes }}\beta _q+\bigl (\zeta +\mathrm {d}\!\!\!/\log q\bigr ){\hat{\otimes }}\beta _q-3\bigl (q^{-1}{\hat{\chi }}\rho +q^{-1}{}^*{\hat{\chi }}\sigma \bigr )-\frac{1}{2}q{{\,\mathrm{tr}\,}}{\underline{\chi }}\alpha _q\Bigr )\nonumber \\ \end{aligned}$$

5.30c $$\begin{aligned} 8\tilde{{\underline{\Lambda }}}^1= & {} -\Bigl ({\underline{m}},-\Omega ^{-1}q^{-1}D{\underline{\beta }}_q+q^{-1}\chi ^\sharp \cdot {\underline{\beta }}_q\nonumber \\&\quad -\,\bigl (q^{-1}{\hat{\omega }}+\Omega ^{-1}D q^{-1}\bigr ){\underline{\beta }}_q-3{\underline{\eta }}\rho +3{}^*{\underline{\eta }}\sigma \Bigr )\nonumber \\&\quad -\,2\Bigl ({\underline{m}},\mathrm {d}\!\!\!/\rho -q{\underline{\chi }}^\sharp \cdot \beta _q+q^{-1}\chi ^\sharp \cdot {\underline{\beta }}_q\Bigr )\nonumber \\&\quad -\,2\Bigl (m,-\Omega ^{-1}q{\underline{D}}{\underline{\beta }}_q+q{\underline{\chi }}^\sharp \cdot \beta _q+\bigl (q{\underline{\omega }}+\Omega ^{-1}{\underline{D}}q\bigr ){\underline{\beta }}_q-\eta ^\sharp \cdot {\underline{\alpha }}_q\Bigr )\nonumber \\&\quad +\,\Bigl ({\hat{i}},-\nabla \!\!\!\!/{\hat{\otimes }}{\underline{\beta }}+\bigl (\zeta +\mathrm {d}\!\!\!/\log q\bigr ){\underline{\beta }}_q-3\bigl (q{\hat{{\underline{\chi }}}}\rho -q{}^*{\hat{{\underline{\chi }}}}\sigma \bigr )-\frac{1}{2}q^{-1}{{\,\mathrm{tr}\,}}\chi {\underline{\alpha }}_q\Bigr )\nonumber \\= & {} -\Bigl ({\underline{m}},2q^{-1}{{\,\mathrm{tr}\,}}\chi {\underline{\beta }}_q-q{{\,\mathrm{tr}\,}}{\underline{\chi }}\beta _q+3\mathrm {d}\!\!\!/\rho -{}^*\mathrm {d}\!\!\!/\sigma -4{\hat{{\underline{\chi }}}}^\sharp \cdot \beta _q+2q^{-1}\chi ^\sharp \cdot {\underline{\beta }}_q\Bigr )\nonumber \\&\quad -\,2\Bigl (m,2q{{\,\mathrm{tr}\,}}{\underline{\chi }}{\underline{\beta }}_q+{{\,\mathrm{div}\,}}\!\!\!\!\!/\,{\underline{\alpha }}_q-2\bigl (\zeta +\mathrm {d}\!\!\!/\log q\bigr )^\sharp \cdot {\underline{\alpha }}_q\Bigr )\nonumber \\&\quad +\,\Bigl ({\hat{i}},-\nabla \!\!\!\!/{\hat{\otimes }}{\underline{\beta }}+\bigl (\zeta +\mathrm {d}\!\!\!/\log q\bigr ){\underline{\beta }}_q-3\bigl (q{\hat{{\underline{\chi }}}}\rho -q{}^*{\hat{{\underline{\chi }}}}\sigma \bigr )-\frac{1}{2}q^{-1}{{\,\mathrm{tr}\,}}\chi {\underline{\alpha }}_q\Bigr )\nonumber \\ \end{aligned}$$

where we used the Bianchi equations in the second step to eliminate ${\underline{D}}\beta _q$, $D\beta _q$, $D{\underline{\beta }}_q$, and ${\underline{D}}{\underline{\beta }}_q$ in terms of derivatives tangential to the spheres.

Let us employ here already that by Lemma 5.6,

5.30d $$\begin{aligned} {\underline{m}}=-m \end{aligned}$$

to conclude

5.30e $$\begin{aligned} 8{\tilde{\Lambda }}^1_q+8\tilde{{\underline{\Lambda }}}^1_q= & {} \Bigl (q{{\,\mathrm{tr}\,}}{\underline{\chi }}-4q^{-1}{{\,\mathrm{tr}\,}}\chi \Bigr )\bigl (m, \beta _q\bigr )+\Bigl (-q^{-1}{{\,\mathrm{tr}\,}}\chi +4q{{\,\mathrm{tr}\,}}{\underline{\chi }}\Bigr )\bigl ({\underline{m}},{\underline{\beta }}_q\bigr )\nonumber \\&\quad -\,\Bigl (m,4q^{-1}{\hat{\chi }}^\sharp \cdot {\underline{\beta }}_q-2q{\hat{{\underline{\chi }}}}^\sharp \cdot \beta _q+4{\hat{{\underline{\chi }}}}^\sharp \cdot \beta _q-2q^{-1}\chi ^\sharp \cdot {\underline{\beta }}_q\Bigr )\nonumber \\&\quad -\,2\Bigl ({\underline{m}},{{\,\mathrm{div}\,}}\!\!\!\!\!/\,\alpha _q+2\bigl (\zeta +\mathrm {d}\!\!\!/\log q\bigr )^\sharp \cdot \alpha _q\Bigr )\nonumber \\&\quad -\,2\Bigl (m,{{\,\mathrm{div}\,}}\!\!\!\!\!/\,{\underline{\alpha }}_q-2\bigl (\zeta +\mathrm {d}\!\!\!/\log q\bigr )^\sharp \cdot {\underline{\alpha }}_q\Bigr )\nonumber \\&\quad +\,\Bigl ({\hat{i}},\nabla \!\!\!\!/{\hat{\otimes }}\beta _q+\bigl (\zeta +\mathrm {d}\!\!\!/\log q\bigr ){\hat{\otimes }}\beta _q\nonumber \\&\quad -\,3\bigl (q^{-1}{\hat{\chi }}\rho +q^{-1}{}^*{\hat{\chi }}\sigma \bigr )-\frac{1}{2}q{{\,\mathrm{tr}\,}}{\underline{\chi }}\alpha _q\Bigr )\nonumber \\&\quad -\,\Bigl ({\hat{i}},\nabla \!\!\!\!/{\hat{\otimes }}{\underline{\beta }}-\bigl (\zeta +\mathrm {d}\!\!\!/\log q\bigr ){\underline{\beta }}_q\nonumber \\&\quad +\,3\bigl (q{\hat{{\underline{\chi }}}}\rho -q{}^*{\hat{{\underline{\chi }}}}\sigma \bigr )+\frac{1}{2}q^{-1}{{\,\mathrm{tr}\,}}\chi {\underline{\alpha }}_q\Bigr ) \end{aligned}$$

In particular we have the following contributions to the divergence (5.7):

5.31a $$\begin{aligned} \boxed {3\cdot 8{\tilde{\rho }}\bigl (\tilde{{\underline{\Lambda }}}^1+{\tilde{\Lambda }}^1\bigr ) = 6\frac{2}{\Omega }\Bigl ( j + \frac{1}{4}({\underline{n}}+n)\Bigr ) {\tilde{\rho }}^2+X_\Lambda ^1+Y_\Lambda ^1+Q_\Lambda ^1+R_\Lambda ^1}\qquad \quad \end{aligned}$$

Note here that

5.31b $$\begin{aligned}&|j |\le \frac{1}{2} q |\Omega {{\,\mathrm{tr}\,}}{\underline{\chi }}-2{\underline{\omega }}|+\frac{1}{2} q^{-1}|\Omega {{\,\mathrm{tr}\,}}\chi -2\omega |\nonumber \\&\quad +\,\frac{1}{2}|{\underline{D}}q|+\frac{1}{2}|D q^{-1}|\le C\bigl (q{{\,\mathrm{tr}\,}}{\underline{\chi }}+q^{-1}{{\,\mathrm{tr}\,}}\chi \bigr ) \end{aligned}$$

5.31c $$\begin{aligned}&|\frac{{\underline{n}}+n}{4} |= \frac{1}{2}|{\underline{D}}q |+ \frac{1}{2} |D q^{-1}|\le \frac{C}{2}\bigl (q{{\,\mathrm{tr}\,}}{\underline{\chi }}+q^{-1}{{\,\mathrm{tr}\,}}\chi \bigr ) \end{aligned}$$

Further to the notation used in (5.31a), we have a quadratic error term

5.31d $$\begin{aligned}&Q_\Lambda ^1:=3{\tilde{\rho }}\Bigl (j+\frac{n+{\underline{n}}}{4}\Bigr )\Bigl \{-2(\eta +{\underline{\eta }}+\mathrm {d}\!\!\!/\log q,\beta _q)+2(\eta +{\underline{\eta }}-\mathrm {d}\!\!\!/\log q,{\underline{\beta }}_q)\Bigr \}\nonumber \\&\quad -\,3{\tilde{\rho }}\frac{{\underline{n}}-n}{4}\Bigl \{-\frac{3}{2}\bigl (q^{-1}{{\,\mathrm{tr}\,}}\chi -q{{\,\mathrm{tr}\,}}{\underline{\chi }}\bigr )\rho +{{\,\mathrm{div}\,}}\!\!\!\!\!/\,\beta _q+{{\,\mathrm{div}\,}}\!\!\!\!\!/\,{\underline{\beta }}_q \nonumber \\&\quad +\,(\zeta +\mathrm {d}\!\!\!/\log q,\beta )-(\zeta +\mathrm {d}\!\!\!/\log q,{\underline{\beta }})-\frac{1}{2}q({\hat{{\underline{\chi }}}},\alpha _q)+\frac{1}{2}q^{-1}({\hat{\chi }},{\underline{\alpha }}_q)\Bigr \} \end{aligned}$$

and a term which we do not estimate but rather keep in its precise form:

5.31e $$\begin{aligned} X_\Lambda ^1:=-\frac{9}{2}\Bigl (j+\frac{n+{\underline{n}}}{4}\Bigr )\bigl (2q{\hat{{\underline{\omega }}}}+2q^{-1}{\hat{\omega }}+q{{\,\mathrm{tr}\,}}{\underline{\chi }}+q^{-1}{{\,\mathrm{tr}\,}}\chi +\hat{{\underline{L}}}q+\hat{{L}}q^{-1}\bigr )\rho {\tilde{\rho }}\nonumber \\ \end{aligned}$$

Finally we collect the following “borderline error terms” in

5.31f $$\begin{aligned}&Y_\Lambda ^1:= -\frac{3}{2}{\tilde{\rho }}q{{\,\mathrm{tr}\,}}{\underline{\chi }}\bigl ({\hat{i}},\alpha _q\bigr )- \frac{3}{2}{\tilde{\rho }}q^{-1}{{\,\mathrm{tr}\,}}\chi \bigl ({\hat{i}},{\underline{\alpha }}_q\bigr ) \nonumber \\&\quad -\,9 q^{-1}{{\,\mathrm{tr}\,}}\chi {\tilde{\rho }}\bigl (m, \beta _q\bigr )+9q{{\,\mathrm{tr}\,}}{\underline{\chi }}{\tilde{\rho }}\bigl ({\underline{m}},{\underline{\beta }}_q\bigr ) \end{aligned}$$

and call

5.31g $$\begin{aligned} R_{\Lambda }^1:= \Bigl (\text {quadratic error term from case } j={\underline{n}}=n=0\Bigr ) - Y_\Lambda ^1 \end{aligned}$$

In fact we can already estimate the quadratic error terms by:

5.31h $$\begin{aligned}&|Q_\Lambda ^1 |\le 12C \bigl (q{{\,\mathrm{tr}\,}}{\underline{\chi }}+q^{-1}{{\,\mathrm{tr}\,}}\chi \bigr ) {\tilde{\rho }}\bigl ( |\mathrm {d}\!\!\!/\log q |+ |\eta |+|{\underline{\eta }}|\bigr )\bigl ( |\beta _q |+ |{\underline{\beta }}_q |\bigr )\nonumber \\&\quad +\, \frac{3C}{2}{\tilde{\rho }}\bigl (q{{\,\mathrm{tr}\,}}{\underline{\chi }}+q^{-1}{{\,\mathrm{tr}\,}}\chi \bigr )\Bigl \{\frac{3}{2}\bigl |q^{-1}{{\,\mathrm{tr}\,}}\chi -q{{\,\mathrm{tr}\,}}{\underline{\chi }}\bigr |\rho + |{{\,\mathrm{div}\,}}\!\!\!\!\!/\,\beta _q|+|{{\,\mathrm{div}\,}}\!\!\!\!\!/\,{\underline{\beta }}_q|\nonumber \\&\quad +\,|\zeta +\mathrm {d}\!\!\!/\log q||\beta _q|+|\zeta +\mathrm {d}\!\!\!/\log q||{\underline{\beta }}_q|+\frac{1}{2}|q{\hat{{\underline{\chi }}}}||\alpha _q|+\frac{1}{2}|q^{-1}{\hat{\chi }}||{\underline{\alpha }}_q|\Bigr \} \end{aligned}$$

5.31i $$\begin{aligned}&|R_\Lambda ^1 |\le 3 |q{{\,\mathrm{tr}\,}}{\underline{\chi }}-q^{-1}{{\,\mathrm{tr}\,}}\chi ||m||{\tilde{\rho }}|| \beta _q|+3|-q^{-1}{{\,\mathrm{tr}\,}}\chi +q{{\,\mathrm{tr}\,}}{\underline{\chi }}||{\underline{m}}||{\tilde{\rho }}||{\underline{\beta }}_q|\nonumber \\&\quad +\,\frac{3|{\tilde{\rho }}|}{\Omega }|m|\Bigl |4q^{-1}\Omega {\hat{\chi }}^\sharp \cdot {\underline{\beta }}_q-2q\Omega {\hat{{\underline{\chi }}}}^\sharp \cdot \beta _q+4q\Omega {\hat{{\underline{\chi }}}}^\sharp \cdot \beta _q-2q^{-1}\Omega \chi ^\sharp \cdot {\underline{\beta }}_q\Bigr |\nonumber \\&\quad +\,6|{\tilde{\rho }}||{\underline{m}}|\Bigl |{{\,\mathrm{div}\,}}\!\!\!\!\!/\,\alpha _q+2\bigl (\zeta +\mathrm {d}\!\!\!/\log q\bigr )^\sharp \cdot \alpha _q\Bigr |\!+\!6|{\tilde{\rho }}||m|\Bigl |{{\,\mathrm{div}\,}}\!\!\!\!\!/\,{\underline{\alpha }}_q-2\bigl (\zeta +\mathrm {d}\!\!\!/\log q\bigr )^\sharp \cdot {\underline{\alpha }}_q\Bigr |\nonumber \\&\quad +\,3|{\tilde{\rho }}||{\hat{i}}|\Bigl |\nabla \!\!\!\!/{\hat{\otimes }}\beta _q+\bigl (\zeta +\mathrm {d}\!\!\!/\log q\bigr ){\hat{\otimes }}\beta _q-3\bigl (q^{-1}{\hat{\chi }}\rho +q^{-1}{}^*{\hat{\chi }}\sigma \bigr )\Bigr |\nonumber \\&\quad +\,3|{\tilde{\rho }}||{\hat{i}}|\Bigl |\nabla \!\!\!\!/{\hat{\otimes }}{\underline{\beta }}-\bigl (\zeta +\mathrm {d}\!\!\!/\log q\bigr ){\underline{\beta }}_q+3\bigl (q{\hat{{\underline{\chi }}}}\rho -q{}^*{\hat{{\underline{\chi }}}}\sigma \bigr )\Bigr | \end{aligned}$$

Note here also that

5.31j $$\begin{aligned} |{\hat{i}}|= & {} \Omega \bigl (q|{\hat{{\underline{\chi }}}}|+q^{-1}|{\hat{\chi }}|\bigr )\le C_0\bigl (q{{\,\mathrm{tr}\,}}{\underline{\chi }}+q^{-1}{{\,\mathrm{tr}\,}}\chi \bigr ) \end{aligned}$$

5.31k $$\begin{aligned} |{\underline{m}}|= & {} |m|=\Omega \bigl (2|\zeta |+|\mathrm {d}\!\!\!/\log q|\bigr ) \le C_0\bigl (q{{\,\mathrm{tr}\,}}{\underline{\chi }}+q^{-1}{{\,\mathrm{tr}\,}}\chi \bigr ) \end{aligned}$$

Let us now turn to the remaining components:

$$\begin{aligned}&\tilde{{\underline{K}}}^1_q := \frac{1}{4}\epsilon \!\!/^{AB}{}^{(N)}J^1[W](e_3,e_A,e_B)\qquad {\tilde{K}}^1_q := \frac{1}{4}\epsilon ^{AB}{}^{(N)}J^1[W](e_4,e_A,e_B)\\&(\tilde{{\underline{I}}}_q^1)_A := \frac{1}{2}{}^{(N)}J^1[W](e_4,e_3,e_A)\qquad ({\tilde{I}}_q^1)_A := \frac{1}{2}{}^{(N)}J^1[W](e_3,e_4,e_A)\\&(\tilde{{\underline{\Xi }}}^1_q)_A := \frac{1}{2}{}^{(N)}J^1[W](e_3,e_3,e_A)\qquad ({\tilde{\Xi }}^1_q)_A := \frac{1}{2}{}^{(N)}J^1[W](e_4,e_4,e_A) \end{aligned}$$

and

$$\begin{aligned}&(\tilde{{\underline{\Theta }}}_q^1)_{AB} := \frac{1}{2}\bigl ( (J\!\!\!\!/_3)_{AB}+(J\!\!\!\!/_3)_{BA}-g\!\!\!/_{AB}{{\,\mathrm{tr}\,}}(J\!\!\!\!/_3)_{AB}\bigr )\\&({\tilde{\Theta }}_q^1)_{AB} := \frac{1}{2}\bigl ( (J\!\!\!\!/_4)_{AB}+(J\!\!\!\!/_4)_{BA}-g\!\!\!/_{AB}{{\,\mathrm{tr}\,}}(J\!\!\!\!/_4)_{AB}\bigr ) \end{aligned}$$

where

$$\begin{aligned} (J\!\!\!\!/_3)_{AB} := {}^{(N)}J^1[W](e_A,e_3,e_B)\qquad (J\!\!\!\!/_4)_{AB} := {}^{(N)}J^1[W](e_A,e_4,e_B)\,. \end{aligned}$$

In the first instance we set

5.32 $$\begin{aligned}&m=0\,,\qquad {\underline{m}}=0\,,\qquad {\hat{i}}=0\,. \end{aligned}$$

5.33 $$\begin{aligned}&8{\tilde{K}}^1_q-8\tilde{{\underline{K}}}^1_q=j\Bigl \{q^{-1}\Omega ^{-1}D\sigma -{{\,\mathrm{curl}\,}}\!\!\!\!\!/\,\beta _q-\frac{3}{2}q^{-1}{{\,\mathrm{tr}\,}}\chi \sigma \nonumber \\&\quad -\,(\zeta +\mathrm {d}\!\!\!/\log q-2{\underline{\eta }})\wedge \beta _q+\frac{1}{2}q{\hat{{\underline{\chi }}}}\wedge \alpha _q\nonumber \\&\quad +\,q\Omega ^{-1}{\underline{D}}\sigma -{{\,\mathrm{curl}\,}}\!\!\!\!\!/\,{\underline{\beta }}_q-\frac{3}{2}q{{\,\mathrm{tr}\,}}{\underline{\chi }}\sigma +(\zeta +\mathrm {d}\!\!\!/\log q+2\eta )\wedge {\underline{\beta }}_q-\frac{1}{2}q^{-1}{\hat{\chi }}\wedge {\underline{\alpha }}_q\Bigr \}\nonumber \\&\quad +\,n\Bigl \{q\Omega ^{-1}{\underline{D}}\sigma +2\eta \wedge {\underline{\beta }}_q\Bigr \}-{\underline{n}}\Bigl \{-q^{-1}\Omega ^{-1}D\sigma -2{\underline{\eta }}\wedge \beta _q\Bigr \}\nonumber \\&\quad =\Bigl (j+\frac{n+{\underline{n}}}{4}\Bigr )\Bigl \{\frac{2}{\Omega }\bigl (q{\underline{D}}\sigma +q^{-1}D\sigma )+4{\underline{\eta }}\wedge \beta _q+4\eta \wedge {\underline{\beta }}_q\Bigr \} \nonumber \\&\quad +\,\frac{n-{\underline{n}}}{2}\Bigl \{q\Omega ^{-1}{\underline{D}}\sigma +2\eta \wedge {\underline{\beta }}_q-q^{-1}\Omega ^{-1}D\sigma -2{\underline{\eta }}\wedge \beta _q\Bigr \}\nonumber \\&\quad =\Bigl (j+\frac{n+{\underline{n}}}{4}\Bigr )\Bigl \{\frac{4}{\Omega }{\tilde{\sigma }}-\frac{3}{2}\Bigl (2q{\hat{{\underline{\omega }}}}+2q^{-1}{\hat{\omega }}+q{{\,\mathrm{tr}\,}}{\underline{\chi }}+q^{-1}{{\,\mathrm{tr}\,}}\chi +\hat{{\underline{L}}}q+\hat{{L}}q^{-1}\Bigr )\sigma \nonumber \\&\quad +\,2(3{\underline{\eta }}-\eta -\mathrm {d}\!\!\!/\log q)\wedge \beta _q+2(3\eta -{\underline{\eta }}+\mathrm {d}\!\!\!/\log q)\wedge {\underline{\beta }}_q\Bigr \}\nonumber \\&\quad +\,\frac{n-{\underline{n}}}{2}\Bigl \{-\frac{3}{2}\bigl (q{{\,\mathrm{tr}\,}}{\underline{\chi }}-q^{-1}{{\,\mathrm{tr}\,}}\chi \bigr )\sigma -{{\,\mathrm{curl}\,}}\!\!\!\!\!/\,{\underline{\beta }}+{{\,\mathrm{curl}\,}}\!\!\!\!\!/\,\beta \nonumber \\&\quad +\,\bigl (\zeta +\mathrm {d}\!\!\!/\log q\bigr )\wedge ({\underline{\beta }}_q+\beta _q)-\frac{1}{2}q^{-1}{\hat{\chi }}\wedge {\underline{\alpha }}_q-\frac{1}{2}q{\hat{{\underline{\chi }}}}\wedge \alpha _q\Bigr \} \end{aligned}$$

5.34 $$\begin{aligned}&8\tilde{{\underline{\Xi }}}^1_q-8\tilde{{\underline{I}}}^1_q=j\Bigl \{q\Omega ^{-1}{\underline{D}}{\underline{\beta }}_q-{{\,\mathrm{div}\,}}\!\!\!\!\!/\,{\underline{\alpha }}_q-q{\underline{\chi }}^\sharp \cdot {\underline{\beta }}_q-q{{\,\mathrm{tr}\,}}{\underline{\chi }}{\underline{\beta }}_q\nonumber \\&\quad -\,\bigl (q{\hat{{\underline{\omega }}}}+\Omega ^{-1}{\underline{D}}q\bigr ){\underline{\beta }}_q+{\underline{\alpha }}_q^\sharp \cdot (2\zeta +2\mathrm {d}\!\!\!/\log q+\eta )\nonumber \\&\quad +q^{-1}\Omega ^{-1}D{\underline{\beta }}_q-\mathrm {d}\!\!\!/\rho +{}^*\mathrm {d}\!\!\!/\sigma -q^{-1}{{\,\mathrm{tr}\,}}\chi {\underline{\beta }}_q+\bigl (q^{-1}{\hat{\omega }}+\Omega ^{-1}D q^{-1}\bigr ){\underline{\beta }}_q\nonumber \\&\quad +\,3{\underline{\eta }}\rho -3{}^*{\underline{\eta }}\sigma -q^{-1}\chi ^\sharp \cdot {\underline{\beta }}_q+2q{\hat{{\underline{\chi }}}}\cdot \beta _q\Bigr \}\nonumber \\&\quad +{\underline{n}}\Bigl \{q^{-1}\Omega ^{-1}D{\underline{\beta }}_q-q^{-1}\chi ^\sharp \cdot {\underline{\beta }}_q+\bigl (q^{-1}{\hat{\omega }}+D q^{-1}\bigr ){\underline{\beta }}_q+3{\underline{\eta }}\rho -3{}^*{\underline{\eta }}\sigma \Bigr \}\nonumber \\&\quad -\,n\Bigl \{-q\Omega ^{-1}{\underline{D}}{\underline{\beta }}_q+q{\underline{\chi }}^\sharp \cdot {\underline{\beta }}_q+\bigl (q{\hat{{\underline{\omega }}}}+\Omega ^{-1}{\underline{D}}q\bigr ){\underline{\beta }}_q-\eta ^\sharp \cdot {\underline{\alpha }}_q\Bigr \}\nonumber \\&\quad =\Bigl (j+\frac{n+{\underline{n}}}{4}\Bigr )\Bigl \{\frac{2}{\Omega }q{\underline{D}}{\underline{\beta }}-2q{\underline{\chi }}^\sharp \cdot {\underline{\beta }}_q-2\bigl (q{\hat{{\underline{\omega }}}}+\Omega ^{-1}{\underline{D}}q\bigr ){\underline{\beta }}_q+2\eta ^\sharp \cdot {\underline{\alpha }}_q\nonumber \\&\quad +\frac{2}{\Omega }q^{-1}D{\underline{\beta }}+2\bigl (q^{-1}{\hat{\omega }}+\Omega ^{-1}Dq^{-1}\bigr ){\underline{\beta }}_q+6{\underline{\eta }}\rho -6{}^*{\underline{\eta }}\sigma \nonumber \\&\quad -\,2q^{-1}\chi ^\sharp \cdot {\underline{\beta }}_q\Bigr \}+jq{{\,\mathrm{tr}\,}}{\underline{\chi }}{\underline{\beta }}_q\nonumber \\&\quad +\,\frac{{\underline{n}}-n}{2}\Bigl \{q^{-1}\Omega ^{-1}D{\underline{\beta }}_q-q^{-1}\chi ^\sharp \cdot {\underline{\beta }}_q+\bigl (q^{-1}{\hat{\omega }}+D q^{-1}\bigr ){\underline{\beta }}_q+3{\underline{\eta }}\rho -3{}^*{\underline{\eta }}\sigma \nonumber \\&\quad -\,q\Omega ^{-1}{\underline{D}}{\underline{\beta }}_q+q{\underline{\chi }}^\sharp \cdot {\underline{\beta }}_q+\bigl (q{\hat{{\underline{\omega }}}}+\Omega ^{-1}{\underline{D}}q\bigr ){\underline{\beta }}_q-\eta ^\sharp \cdot {\underline{\alpha }}_q\Bigr \}\nonumber \\&\quad =\Bigl (j+\frac{n+{\underline{n}}}{4}\Bigr )\Bigl \{\frac{4}{\Omega }{\tilde{{\underline{\beta }}}}_q-\frac{1}{2}\bigl (10 q{\hat{{\underline{\omega }}}}+2 q^{-1}{\hat{\omega }}\nonumber \\&\quad +\,3q{{\,\mathrm{tr}\,}}{\underline{\chi }}+3q^{-1}{{\,\mathrm{tr}\,}}\chi +5 \hat{{\underline{L}}}q-3\hat{{L}}q\bigr ){\underline{\beta }}_q\nonumber \\&\quad +\,3\bigl (\eta +{\underline{\eta }}+\mathrm {d}\!\!\!/\log q\bigr )\rho -3{}^*\bigl (\eta +{\underline{\eta }}+\mathrm {d}\!\!\!/\log q\bigr )\sigma \nonumber \\&\quad +\,{\underline{\alpha }}^\sharp _q\cdot \bigl (\eta +{\underline{\eta }}- \mathrm {d}\!\!\!/\log q\bigr )\Bigr \}+j\,q{{\,\mathrm{tr}\,}}{\underline{\chi }}{\underline{\beta }}_q\nonumber \\&\quad +\,\frac{{\underline{n}}-n}{2}\Bigl \{\bigl (-q^{-1}{{\,\mathrm{tr}\,}}\chi +2q{{\,\mathrm{tr}\,}}{\underline{\chi }}\bigr ){\underline{\beta }}_q-\mathrm {d}\!\!\!/\rho +{}^*\mathrm {d}\!\!\!/\sigma +{{\,\mathrm{div}\,}}\!\!\!\!\!/\,{\underline{\alpha }}_q+2q {\hat{{\underline{\chi }}}}^\sharp \cdot \beta \nonumber \\&\quad -\,2\bigl (\zeta +\mathrm {d}\!\!\!/\log q\bigr )^\sharp \cdot {\underline{\alpha }}_q\Bigr \} \end{aligned}$$

5.35 $$\begin{aligned}&-16\tilde{{\underline{I}}}^1_q=j\Bigl \{\frac{2}{\Omega }q^{-1}D{\underline{\beta }}-2\mathrm {d}\!\!\!/\rho +2{}^*\mathrm {d}\!\!\!/\sigma \nonumber \\&\quad -\,2q^{-1}{{\,\mathrm{tr}\,}}\chi {\underline{\beta }}+2\bigl (q^{-1}{\hat{\omega }}+\Omega ^{-1}D q^{-1}\bigr ){\underline{\beta }}_q\nonumber \\&\quad +\,6{\underline{\eta }}\rho -6{}^*{\underline{\eta }}\sigma -2q^{-1}\chi \cdot {\underline{\beta }}_q+4q{\hat{{\underline{\chi }}}}\cdot \beta _q\Bigr \} -2n\Bigl \{-\Omega ^{-1}q{\underline{D}}{\underline{\beta }}\nonumber \\&\quad +\,q{\underline{\chi }}^\sharp \cdot {\underline{\beta }}_q+\bigl (q{\hat{{\underline{\omega }}}}+\Omega ^{-1}{\underline{D}}q\bigr ){\underline{\beta }}_q-\eta ^\sharp \cdot {\underline{\alpha }}_q\Bigr \}\nonumber \\&\quad =4j\Bigl \{\frac{1}{\Omega }q^{-1}D{\underline{\beta }}+\bigl (q^{-1}{\hat{\omega }}+D q^{-1}\bigr ){\underline{\beta }}_q+3{\underline{\eta }}\rho \nonumber \\&\quad -\,3{}^*{\underline{\eta }}\sigma -q^{-1}\chi ^\sharp \cdot {\underline{\beta }}_q\Bigr \} +2n\Bigl \{\Omega ^{-1}q{\underline{D}}{\underline{\beta }}\nonumber \\&\quad -\,q{\underline{\chi }}^\sharp \cdot {\underline{\beta }}-\bigl (q{\hat{{\underline{\omega }}}}+\Omega ^{-1}{\underline{D}}q\bigr ){\underline{\beta }}_q+\eta ^\sharp \cdot {\underline{\alpha }}_q\Bigr \}\nonumber \\&\quad =\bigl (2j+n\bigr )\Bigl \{\frac{2}{\Omega }{\tilde{{\underline{\beta }}}}_q-\frac{1}{4}\bigl (10 q{\hat{{\underline{\omega }}}}+2 q^{-1}{\hat{\omega }}\nonumber \\&\quad +\,3q{{\,\mathrm{tr}\,}}{\underline{\chi }}+3q^{-1}{{\,\mathrm{tr}\,}}\chi +5\hat{{\underline{L}}}q-3\hat{{L}}q^{-1}\bigr ){\underline{\beta }}_q\nonumber \\&\quad +\,\frac{3}{2}\bigl (\eta +{\underline{\eta }}+\mathrm {d}\!\!\!/\log q\bigr )\rho -\frac{3}{2}{}^*\bigl (\eta +{\underline{\eta }}+\mathrm {d}\!\!\!/\log q\bigr )\sigma \nonumber \\&\quad +\,\frac{1}{2}{\underline{\alpha }}^\sharp _q\cdot \bigl (\eta +{\underline{\eta }}- \mathrm {d}\!\!\!/\log q\bigr )\Bigr \}\nonumber \\&\quad +\,\bigl (2j-n\bigr )\Bigl \{\bigl (2q{{\,\mathrm{tr}\,}}{\underline{\chi }}-q^{-1}{{\,\mathrm{tr}\,}}\chi \bigr ){\underline{\beta }}_q-\mathrm {d}\!\!\!/\rho +{}^*\mathrm {d}\!\!\!/\sigma +{{\,\mathrm{div}\,}}\!\!\!\!\!/\,{\underline{\alpha }}_q\nonumber \\&\quad +\,2q {\hat{{\underline{\chi }}}}^\sharp \cdot \beta -2\bigl (\zeta +\mathrm {d}\!\!\!/\log q\bigr )^\sharp \cdot {\underline{\alpha }}_q\Bigr \} \end{aligned}$$

5.36 $$\begin{aligned}&8{\tilde{I}}^1_q-8{\tilde{\Xi }}^1_q=j\Bigl \{\Omega ^{-1}q{\underline{D}}\beta +\mathrm {d}\!\!\!/\rho +{}^*\mathrm {d}\!\!\!/\sigma -q{{\,\mathrm{tr}\,}}{\underline{\chi }}\beta _q+\bigl (q{\hat{{\underline{\omega }}}}+\Omega ^{-1}{\underline{D}}q\bigr )\beta _q\nonumber \\&\quad -3\eta \rho -3{}^*\eta \sigma -q{\underline{\chi }}^\sharp \cdot \beta _q+2q^{-1}\chi ^\sharp \cdot {\underline{\beta }}_q\nonumber \\&\quad +\Omega ^{-1}q^{-1}D\beta _q+{{\,\mathrm{div}\,}}\!\!\!\!\!/\,\alpha -q^{-1}\chi ^\sharp \cdot \beta _q-q^{-1}{{\,\mathrm{tr}\,}}\chi \beta _q\nonumber \\&\quad -\bigl (q^{-1}{\hat{\omega }}+D q^{-1}\bigr )\beta _q+(2\zeta +2\mathrm {d}\!\!\!/\log q-{\underline{\eta }})^\sharp \cdot \alpha _q\Bigr \}\nonumber \\&\quad +{\underline{n}}\Bigl \{\Omega ^{-1}q^{-1}D\beta _q-q^{-1}\chi ^\sharp \cdot \beta _q-\bigl (q^{-1}{\hat{\omega }}+\Omega ^{-1}D q^{-1}\bigr )\beta _q-{\underline{\eta }}^\sharp \cdot \alpha _q\Bigr \}\nonumber \\&\quad -\,n\Bigl \{-\Omega ^{-1}q{\underline{D}}\beta _q+q{\underline{\chi }}^\sharp \cdot \beta _q-\bigl (q{\hat{{\underline{\omega }}}}+\Omega ^{-1}{\underline{D}}q\bigr )\beta _q+3\eta \rho +3{}^*\eta \sigma \Bigr \}\nonumber \\&\quad =\Bigl (j+\frac{{\underline{n}}+n}{4}\Bigr )\Bigl \{\frac{2}{\Omega }q{\underline{D}}\beta +2\bigl (q{\hat{{\underline{\omega }}}}+\Omega ^{-1}{\underline{D}}q\bigr )\beta _q-6\eta \rho -6{}^*\eta \sigma -2q{\underline{\chi }}^\sharp \cdot \beta _q\nonumber \\&\quad +\,\frac{2}{\Omega }q^{-1}D\beta _q-2q^{-1}\chi ^\sharp \cdot \beta _q-2\bigl (q^{-1}{\hat{\omega }}+\Omega ^{-1}Dq^{-1}\bigr )\beta _q\nonumber \\&\quad -2{\underline{\eta }}^\sharp \cdot \alpha _q\Bigr \}+jq^{-1}{{\,\mathrm{tr}\,}}\chi \beta _q\nonumber \\&\quad +\frac{{\underline{n}}-n}{2}\Bigl \{\Omega ^{-1}q^{-1}D\beta _q-q^{-1}\chi ^\sharp \cdot \beta _q-\bigl (q^{-1}{\hat{\omega }}+\Omega ^{-1}D q^{-1}\bigr )\beta _q-{\underline{\eta }}^\sharp \cdot \alpha _q\nonumber \\&\quad -\,\Omega ^{-1}q{\underline{D}}\beta _q+q{\underline{\chi }}^\sharp \cdot \beta _q-\bigl (q{\hat{{\underline{\omega }}}}+\Omega ^{-1}{\underline{D}}q\bigr )\beta _q+3\eta \rho +3{}^*\eta \sigma \Bigr \}\nonumber \\&\quad =\Bigl (j+\frac{{\underline{n}}+n}{4}\Bigr )\Bigl \{\frac{4}{\Omega }{\tilde{\beta }}_q-\frac{1}{2}\bigl (2q{\hat{{\underline{\omega }}}}+10q^{-1}{\hat{\omega }}\nonumber \\&\quad +3q{{\,\mathrm{tr}\,}}{\underline{\chi }}+3q{{\,\mathrm{tr}\,}}\chi -\hat{{\underline{L}}}q+5\hat{{L}}q\bigr )\beta _q\nonumber \\&\quad -\,3\bigl (\eta +{\underline{\eta }}-\mathrm {d}\!\!\!/\log q\bigr )\rho -3{}^*\bigl (\eta +{\underline{\eta }}-\mathrm {d}\!\!\!/\log q\bigr )\sigma \nonumber \\&\quad -\alpha ^\sharp _q\cdot \bigl (\eta +{\underline{\eta }}+\mathrm {d}\!\!\!/\log q\bigr )\Bigr \}+jq^{-1}{{\,\mathrm{tr}\,}}\chi \beta _q\nonumber \\&\quad +\frac{{\underline{n}}-n}{2}\Bigl \{\bigl (q{{\,\mathrm{tr}\,}}{\underline{\chi }}-2 q^{-1}{{\,\mathrm{tr}\,}}\chi \bigr )\beta _q+{{\,\mathrm{div}\,}}\!\!\!\!\!/\,\alpha _q-\mathrm {d}\!\!\!/\rho -{}^*\mathrm {d}\!\!\!/\sigma \nonumber \\&\quad +\,2\bigl (\zeta +\mathrm {d}\!\!\!/\log q\bigr )^\sharp \cdot {\underline{\alpha }}_q-2q^{-1}{\hat{\chi }}^\sharp \cdot {\underline{\beta }}_q\Bigr \} \end{aligned}$$

5.37 $$\begin{aligned}&16{\tilde{I}}^1_q=j\Bigl \{2\Omega ^{-1}q{\underline{D}}\beta +2\mathrm {d}\!\!\!/\rho +2{}^*\mathrm {d}\!\!\!/\sigma -2q{{\,\mathrm{tr}\,}}{\underline{\chi }}\beta _q+2{\hat{{\underline{\omega }}}}\beta _q-6\eta \rho -6{}^*\eta \sigma \nonumber \\&\quad -\,2q{\underline{\chi }}^\sharp \cdot \beta _q+4q^{-1}{\hat{\chi }}^\sharp \cdot {\underline{\beta }}_q\Bigr \}\nonumber \\&\quad +2{\underline{n}}\Bigl \{\Omega ^{-1}q^{-1}D\beta -q^{-1}\chi ^\sharp \cdot \beta _q-\bigl (q^{-1}{\hat{\omega }}+\Omega ^{-1} D q^{-1}\bigr )\beta _q-{\underline{\eta }}^\sharp \cdot \alpha _q\Bigr \}\nonumber \\&\quad =4j\Bigl \{\frac{1}{\Omega }q{\underline{D}}\beta +\bigl (q{\hat{{\underline{\omega }}}}+\Omega ^{-1}{\underline{D}}q\bigr )\beta _q-q{\underline{\chi }}^\sharp \cdot \beta _q-3\eta \rho -3{}^*\eta \sigma \Bigr \}\nonumber \\&\quad +2{\underline{n}}\Bigl \{\Omega ^{-1}q^{-1}D\beta -q^{-1}\chi ^\sharp \cdot \beta _q-\bigl (q^{-1}{\hat{\omega }}+\Omega ^{-1} D q^{-1}\bigr )\beta _q-{\underline{\eta }}^\sharp \cdot \alpha _q\Bigr \}\nonumber \\&\quad =\Bigl (\frac{2j+{\underline{n}}}{2}\Bigr )\Bigl \{\frac{4}{\Omega }{\tilde{\beta }}_q-\frac{1}{2}\bigl (2q{\hat{{\underline{\omega }}}}+10q^{-1}{\hat{\omega }}+3q{{\,\mathrm{tr}\,}}{\underline{\chi }}+3q{{\,\mathrm{tr}\,}}\chi -\hat{{\underline{L}}}q+5\hat{{L}}q\bigr )\beta _q\nonumber \\&\quad -\,3\bigl (\eta +{\underline{\eta }}-\mathrm {d}\!\!\!/\log q\bigr )\rho -3{}^*\bigl (\eta +{\underline{\eta }}-\mathrm {d}\!\!\!/\log q\bigr )\sigma -\alpha ^\sharp _q\cdot \bigl (\eta +{\underline{\eta }}+\mathrm {d}\!\!\!/\log q\bigr )\Bigr \}\nonumber \\&\quad +\bigl (2j-{\underline{n}}\bigr )\Bigl \{\bigl (q{{\,\mathrm{tr}\,}}{\underline{\chi }}-2 q^{-1}{{\,\mathrm{tr}\,}}\chi \bigr )\beta _q+{{\,\mathrm{div}\,}}\!\!\!\!\!/\,\alpha _q-\mathrm {d}\!\!\!/\rho -{}^*\mathrm {d}\!\!\!/\sigma \nonumber \\&\quad +\,2\bigl (\zeta +\mathrm {d}\!\!\!/\log q\bigr )^\sharp \cdot {\underline{\alpha }}_q-2q^{-1}{\hat{\chi }}^\sharp \cdot {\underline{\beta }}_q\Bigr \} \end{aligned}$$

5.38 $$\begin{aligned}&4\tilde{{\underline{\Theta }}}^1_q=\frac{1}{2}j\Bigl \{\Omega ^{-1}q^{-1}{\hat{D}}{\underline{\alpha }}_q-\nabla \!\!\!\!/{\hat{\otimes }}{\underline{\beta }}_q-\frac{3}{2}q^{-1}{{\,\mathrm{tr}\,}}\chi {\underline{\alpha }}+2\bigl (q^{-1}{\hat{\omega }}+\Omega ^{-1}D q^{-1}\bigr ){\underline{\alpha }}_q\nonumber \\&\quad +\,(\zeta +\mathrm {d}\!\!\!/\log q+4{\underline{\eta }}){\hat{\otimes }}{\underline{\beta }}_q-3q{\hat{{\underline{\chi }}}}\rho +3q{}^*{\hat{{\underline{\chi }}}}\sigma \Bigr \}\nonumber \\&\quad +\,\frac{1}{2}n\Bigl \{\Omega ^{-1}q{\hat{{\underline{D}}}}{\underline{\alpha }}_q-q{{\,\mathrm{tr}\,}}{\underline{\chi }}{\underline{\alpha }}_q-2\bigl (q{\hat{{\underline{\omega }}}}+\Omega ^{-1}D q^{-1}\bigr ){\underline{\alpha }}_q\Bigr \}\nonumber \\&\quad =j\Bigl \{\frac{1}{\Omega }q^{-1}{\hat{D}}{\underline{\alpha }}-q^{-1}{{\,\mathrm{tr}\,}}\chi {\underline{\alpha }}_q+2\bigl (q^{-1}{\hat{\omega }}+\Omega ^{-1}D q^{-1}\bigr ){\underline{\alpha }}_q+4{\underline{\eta }}{\hat{\otimes }}{\underline{\beta }}\Bigr \}\nonumber \\&\quad +\,\frac{1}{2}n\Bigl \{\Omega ^{-1}q{\hat{{\underline{D}}}}{\underline{\alpha }}_q-q{{\,\mathrm{tr}\,}}{\underline{\chi }}{\underline{\alpha }}_q-2\bigl (q{\hat{{\underline{\omega }}}}+\Omega ^{-1}{\underline{D}}q\bigr ){\underline{\alpha }}_q\Bigr \}\nonumber \\&\quad =\frac{2j+n}{4}\Bigl \{\frac{2}{\Omega }{\tilde{{\underline{\alpha }}}}_q-\frac{1}{4}\bigl (-2q^{-1}{\hat{\omega }}+14q{\hat{{\underline{\omega }}}}+3q{{\,\mathrm{tr}\,}}{\underline{\chi }}\nonumber \\&\quad +\,3q^{-1}{{\,\mathrm{tr}\,}}\chi -\hat{{L}}q^{-1}+7\hat{{\underline{L}}}q\bigr ){\underline{\alpha }}_q +2\bigl (\eta +{\underline{\eta }}+\mathrm {d}\!\!\!/\log q\bigr ){\hat{\otimes }}{\underline{\beta }}_q\Bigr \}\nonumber \\&\quad +\frac{2j-n}{4}\Bigl \{-\frac{2}{\Omega }{\tilde{{\underline{\alpha }}}}_q+\frac{1}{4}\bigl (-2q^{-1}{\hat{\omega }}\nonumber \\&\quad +\,14q{\hat{{\underline{\omega }}}}+3q{{\,\mathrm{tr}\,}}{\underline{\chi }}+3q^{-1}{{\,\mathrm{tr}\,}}\chi -\hat{{L}}q^{-1}+7\hat{{\underline{L}}}q\bigr ){\underline{\alpha }}_q\nonumber \\&\quad -\,q^{-1}{{\,\mathrm{tr}\,}}\chi {\underline{\alpha }}_q-2\bigl (\eta +3{\underline{\eta }}\bigr ){\hat{\otimes }}{\underline{\beta }}_q-2\nabla \!\!\!\!/{\hat{\otimes }}{\underline{\beta }}_q-6q{\hat{{\underline{\chi }}}}\rho +6{}^*{\hat{{\underline{\chi }}}}\sigma \Bigr \} \end{aligned}$$

5.39 $$\begin{aligned}&4{\tilde{\Theta }}^1_q=\frac{1}{2}j\Bigl \{\Omega ^{-1}q{\hat{{\underline{D}}}}\alpha _q+\nabla \!\!\!\!/{\hat{\otimes }}\beta _q-\frac{3}{2}q{{\,\mathrm{tr}\,}}{\underline{\chi }}\alpha _q+2\bigl (q{\hat{{\underline{\omega }}}}+\Omega ^{-1}{\underline{D}}q\bigr )\alpha _q\nonumber \\&\quad +\,(\zeta +\mathrm {d}\!\!\!/\log q-4\eta ){\hat{\otimes }}\beta _q-3q^{-1}{\hat{\chi }}\rho -3q^{-1}{}^*{\hat{\chi }}\sigma \Bigr \}\nonumber \\&\quad +\,\frac{1}{2}{\underline{n}}\Bigl \{\Omega ^{-1}q^{-1}{\hat{D}}\alpha -q^{-1}{{\,\mathrm{tr}\,}}\chi \alpha _q-2\bigl (q^{-1}{\hat{\omega }}+\Omega ^{-1}D q^{-1}\bigr )\alpha _q\Bigr \}\nonumber \\&\quad =j\Bigl \{\frac{1}{\Omega }q{\hat{{\underline{D}}}}\alpha -q{{\,\mathrm{tr}\,}}{\underline{\chi }}\alpha _q+2\bigl (q{\hat{{\underline{\omega }}}}+\Omega ^{-1}{\underline{D}}q\bigr )\alpha _q-4\eta {\hat{\otimes }}\beta _q\Bigr \}\nonumber \\&\quad +\,\frac{1}{2}{\underline{n}}\Bigl \{\Omega ^{-1}q^{-1}{\hat{D}}\alpha _q-q^{-1}{{\,\mathrm{tr}\,}}\chi \alpha _q-2\bigl (q^{-1}{\hat{\omega }}+\Omega ^{-1}D q^{-1}\bigr )\alpha _q\Bigr \}\nonumber \\&\quad =\frac{2j+{\underline{n}}}{4}\Bigl \{\frac{2}{\Omega }{\tilde{\alpha }}_q-\frac{1}{4}\bigl (-2q{\hat{{\underline{\omega }}}}+14q^{-1}{\hat{\omega }}+3q{{\,\mathrm{tr}\,}}{\underline{\chi }}\nonumber \\&\quad +\,3q^{-1}{{\,\mathrm{tr}\,}}\chi -\hat{{\underline{L}}}q+7\hat{{L}}q^{-1}\bigr )\alpha _q\nonumber \\&\quad -\,2\bigl (\eta +{\underline{\eta }}-\mathrm {d}\!\!\!/\log q\bigr ){\hat{\otimes }}\beta _q\Bigr \}\nonumber \\&\quad +\frac{2j-{\underline{n}}}{4}\Bigl \{-\frac{2}{\Omega }{\tilde{\alpha }}_q+\frac{1}{4}\bigl (-2q{\hat{{\underline{\omega }}}}+14q^{-1}{\hat{\omega }}+3q{{\,\mathrm{tr}\,}}{\underline{\chi }}\nonumber \\&\quad +\,3q^{-1}{{\,\mathrm{tr}\,}}\chi -\hat{{\underline{L}}}q+7\hat{{L}}q^{-1}\bigr )\alpha _q\nonumber \\&\quad +\,2\bigl (\eta +{\underline{\eta }}-\mathrm {d}\!\!\!/\log q\bigr ){\hat{\otimes }}\beta _q-q{{\,\mathrm{tr}\,}}{\underline{\chi }}\alpha _q\nonumber \\&\quad +\,2\nabla \!\!\!\!/{\hat{\otimes }}\beta _q+2(\zeta +\mathrm {d}\!\!\!/\log q){\hat{\otimes }}\beta -6{\hat{\chi }}\rho -6{}^*{\hat{\chi }}\sigma \Bigr \} \end{aligned}$$

In the second instance we set

$$\begin{aligned} j=0\,,\qquad n=0\,,\qquad {\underline{n}}=0\,, \end{aligned}$$

but we shall not list these expressions here and instead collect all terms with factors *m*, ${\underline{m}}$, and ${\hat{i}}$, directly under the label $R^1$, and $Y^1$ below. In view of Lemma 5.10 the algebraic expressions for the null components of $J^1$ involving factors in *m*, ${\underline{m}}$, and ${\hat{i}}$ can be read off verbatim from Lemma 14.1 in [8]. We employ immediately Prop. 5.7 to eliminate derivatives of *W*, *W* in null directions, *DW*, ${\underline{D}}W$ in favor of angular derivatives $\nabla \!\!\!\!/W$. In the extreme cases where derivatives appear that cannot be directly eliminated using the Bianchi equations — such as ${\hat{{\underline{D}}}}{\underline{\alpha }}$ in $\tilde{{\underline{\Xi }}}^1$ — we also use Lemma 5.8 to rewrite these in terms of $\tilde{{\mathcal {L}}}_{N}W$:

5.40 $$\begin{aligned}&\frac{q}{\Omega }{\hat{{\underline{D}}}}{\underline{\alpha }}-q{{\,\mathrm{tr}\,}}{\underline{\chi }}{\underline{\alpha }}_q-2({\hat{{\underline{\omega }}}}+\hat{{\underline{L}}}q){\underline{\alpha }}_q=\nonumber \\&\quad =\frac{2}{\Omega }{\tilde{{\underline{\alpha }}}}-\frac{1}{4}\bigl (6q^{-1}{\hat{\omega }}+6q{\hat{{\underline{\omega }}}}-q{{\,\mathrm{tr}\,}}{\underline{\chi }}-q^{-1}{{\,\mathrm{tr}\,}}\chi +7\hat{{L}}q^{-1}-\hat{{\underline{L}}}q\bigr ){\underline{\alpha }}_q\nonumber \\&\quad -\,q^{-1}\Omega ^{-1}{\hat{D}}{\underline{\alpha }}-q{{\,\mathrm{tr}\,}}{\underline{\chi }}{\underline{\alpha }}_q-2({\hat{{\underline{\omega }}}}+\hat{{\underline{L}}}q){\underline{\alpha }}_q+2\bigl (2\zeta +\mathrm {d}\!\!\!/\log q\bigr ){\hat{\otimes }}{\underline{\beta }}_q\nonumber \\&\quad = \frac{2}{\Omega }{\tilde{{\underline{\alpha }}}}-\frac{1}{4}\bigl (6q^{-1}{\hat{\omega }}+6q{\hat{{\underline{\omega }}}}+3q{{\,\mathrm{tr}\,}}{\underline{\chi }}+q^{-1}{{\,\mathrm{tr}\,}}\chi +7\hat{{L}}q^{-1}-\hat{{\underline{L}}}q\bigr ){\underline{\alpha }}_q\nonumber \\&\quad +\,\nabla \!\!\!\!/{\hat{\otimes }}{\underline{\beta }}+\bigl (4{\underline{\eta }}+3\zeta +\mathrm {d}\!\!\!/\log q){\hat{\otimes }}{\underline{\beta }}_q+3q{\hat{{\underline{\chi }}}}\rho -3q{}^*{\hat{{\underline{\chi }}}}\sigma \end{aligned}$$

In conclusion:

5.41a $$\begin{aligned} \boxed {3(-8{\tilde{\sigma }})\bigl (\tilde{{\underline{K}}}^1-{\tilde{K}}^1\bigr )=6 \frac{2}{\Omega }\Bigl (j+\frac{n+{\underline{n}}}{4}\Bigr ) {\tilde{\sigma }}^2+X_K^1+Q_K^1+Y_K^1+R_K^1}\qquad \quad \end{aligned}$$

5.41b $$\begin{aligned}&X_K^1:=-\frac{9}{2}\Bigl (j+\frac{n+{\underline{n}}}{4}\Bigr )\nonumber \\&\quad \Bigl (2q{\hat{{\underline{\omega }}}}+2q^{-1}{\hat{\omega }}+q{{\,\mathrm{tr}\,}}{\underline{\chi }}+q^{-1}{{\,\mathrm{tr}\,}}\chi +\hat{{\underline{L}}}q+\hat{{L}}q^{-1}\Bigr ){\tilde{\sigma }}\sigma \end{aligned}$$

5.41c $$\begin{aligned}&Q_K^1:=3{\tilde{\sigma }}\Bigl (j+\frac{n+{\underline{n}}}{4}\Bigr )\Bigl \{2(3{\underline{\eta }}-\eta -\mathrm {d}\!\!\!/\log q)\wedge \beta _q+2(3\eta -{\underline{\eta }}+\mathrm {d}\!\!\!/\log q)\wedge {\underline{\beta }}_q\Bigr \}\nonumber \\&\quad +3{\tilde{\sigma }}\frac{n-{\underline{n}}}{2}\Bigl \{-\frac{3}{2}\bigl (q{{\,\mathrm{tr}\,}}{\underline{\chi }}-q^{-1}{{\,\mathrm{tr}\,}}\chi \bigr )\sigma -{{\,\mathrm{curl}\,}}\!\!\!\!\!/\,{\underline{\beta }}+{{\,\mathrm{curl}\,}}\!\!\!\!\!/\,\beta \nonumber \\&\quad +\,\bigl (\zeta +\mathrm {d}\!\!\!/\log q\bigr )\wedge ({\underline{\beta }}_q+\beta _q)-\frac{1}{2}q^{-1}{\hat{\chi }}\wedge {\underline{\alpha }}_q-\frac{1}{2}q{\hat{{\underline{\chi }}}}\wedge \alpha _q\Bigr \} \end{aligned}$$

5.41d $$\begin{aligned}&Y_K^1 := \frac{3}{2}q{{\,\mathrm{tr}\,}}{\underline{\chi }}{\tilde{\sigma }}{\hat{i}}\wedge \alpha -\frac{3}{2}q^{-1}{{\,\mathrm{tr}\,}}\chi {\tilde{\sigma }}{\hat{i}}\wedge {\underline{\alpha }}_q-9q{{\,\mathrm{tr}\,}}{\underline{\chi }}{\tilde{\sigma }}m\wedge {\underline{\beta }}_q\nonumber \\&\quad +\,9q^{-1}{{\,\mathrm{tr}\,}}\chi {\tilde{\sigma }}m\wedge \beta _q \end{aligned}$$

5.41e $$\begin{aligned}&R_K^1 := 3{\tilde{\sigma }}m\wedge \Bigl \{\bigl (q^{-1}{{\,\mathrm{tr}\,}}\chi -q{{\,\mathrm{tr}\,}}{\underline{\chi }}\bigr ){\underline{\beta }}_q+\bigl (q^{-1}{{\,\mathrm{tr}\,}}\chi -q{{\,\mathrm{tr}\,}}{\underline{\chi }}\bigr ) \beta _q\nonumber \\&\quad +\,2\mathrm {d}\!\!\!/\rho -2q{\hat{{\underline{\chi }}}}^\sharp \cdot \beta _q+2q^{-1}{\hat{\chi }}^\sharp \cdot {\underline{\beta }}_q\Bigr \}\nonumber \\&\quad -2{{\,\mathrm{div}\,}}\!\!\!\!\!/\,\alpha -2{{\,\mathrm{div}\,}}\!\!\!\!\!/\,{\underline{\alpha }}-2(\eta -2\zeta -2\mathrm {d}\!\!\!/\log q)^\sharp \cdot \alpha _q-2({\underline{\eta }}+2\zeta +2\mathrm {d}\!\!\!/\log q)^\sharp \cdot \alpha _q\Bigr \}\nonumber \\&\quad -6{\tilde{\sigma }}\Bigl ({\underline{m}},{\underline{\eta }}^\sharp \cdot {}^*\alpha _q+\eta ^\sharp \cdot {}^*{\underline{\alpha }}_q\Bigr )\nonumber \\&\quad -3{\tilde{\sigma }}\Bigl ({\hat{i}},\nabla \!\!\!\!/{\hat{\otimes }}{}^*\beta +\nabla \!\!\!\!/{\hat{\otimes }}{}^*{\underline{\beta }}+(\zeta +\mathrm {d}\!\!\!/\log q){\hat{\otimes }}({}^*\beta _q-{}^*{\underline{\beta }}_q)\nonumber \\&\quad +\,3(q{}^*{\hat{{\underline{\chi }}}}-q^{-1}{}^*{\hat{\chi }})\rho +3(q{\hat{{\underline{\chi }}}}+q^{-1}{\hat{\chi }})\sigma \Bigr ) \end{aligned}$$

5.42a $$\begin{aligned} \boxed { 8({\tilde{{\underline{\beta }}}}_q,\tilde{{\underline{\Xi }}}^1-\tilde{{\underline{I}}}^1) -16({\tilde{{\underline{\beta }}}}_q,\tilde{{\underline{I}}}^1) = 2 \frac{2}{\Omega } \Bigl (j+\frac{n+{\underline{n}}}{4}+\frac{2j+n}{2}\Bigr ) |{\tilde{{\underline{\beta }}}}_q |^2 + X_{{\underline{\Xi }}}^1+ Q_{{\underline{\Xi }}}^1 +Y_{{\underline{\Xi }}}^1+R_{{\underline{\Xi }}}^1}\nonumber \\ \end{aligned}$$

5.42b $$\begin{aligned}&X_{{\underline{\Xi }}}^1:=-\frac{1}{2}\Bigl (j+\frac{n+{\underline{n}}}{4}+\frac{2j+n}{2}\Bigr )\bigl (10 q{\hat{{\underline{\omega }}}}+2 q^{-1}{\hat{\omega }}+3q{{\,\mathrm{tr}\,}}{\underline{\chi }}+3q^{-1}{{\,\mathrm{tr}\,}}\chi +5 \hat{{\underline{L}}}q\nonumber \\&\quad -\,3\hat{{L}}q\bigr )({\tilde{{\underline{\beta }}}}_q,{\underline{\beta }}_q) +j\,q{{\,\mathrm{tr}\,}}{\underline{\chi }}({\tilde{{\underline{\beta }}}}_q,{\underline{\beta }}_q)+\Bigl (\frac{{\underline{n}}-n}{2}+2j-n\Bigr )\nonumber \\&\quad \bigl (2q{{\,\mathrm{tr}\,}}{\underline{\chi }}-q^{-1}{{\,\mathrm{tr}\,}}\chi \bigr )({\tilde{{\underline{\beta }}}}_q,{\underline{\beta }}_q) \end{aligned}$$

5.42c $$\begin{aligned}&Q_{{\underline{\Xi }}}^1:=\Bigl (j+\frac{n+{\underline{n}}}{4}+\frac{2j+n}{2}\Bigr ) \Bigl ({\tilde{{\underline{\beta }}}}_q,3\bigl (\eta +{\underline{\eta }}+\mathrm {d}\!\!\!/\log q\bigr )\rho -3{}^*\nonumber \\&\quad \bigl (\eta +{\underline{\eta }}+\mathrm {d}\!\!\!/\log q\bigr )\sigma +{\underline{\alpha }}^\sharp _q\cdot \bigl (\eta +{\underline{\eta }}- \mathrm {d}\!\!\!/\log q\bigr )\Bigr ) +\Bigl (\frac{{\underline{n}}-n}{2}+2j-n\Bigr )\nonumber \\&\quad \Bigl ({\tilde{{\underline{\beta }}}}_q,-\mathrm {d}\!\!\!/\rho +{}^*\mathrm {d}\!\!\!/\sigma +{{\,\mathrm{div}\,}}\!\!\!\!\!/\,{\underline{\alpha }}_q+2q {\hat{{\underline{\chi }}}}^\sharp \cdot \beta -2\bigl (\zeta +\mathrm {d}\!\!\!/\log q\bigr )^\sharp \cdot {\underline{\alpha }}_q\Bigr ) \end{aligned}$$

5.42d $$\begin{aligned}&Y_{{\underline{\Xi }}}^1 := 6q{{\,\mathrm{tr}\,}}{\underline{\chi }}\,{\tilde{{\underline{\beta }}}}^A{\hat{i}}_A^{B}\beta _B +\frac{1}{2}q^{-1}{{\,\mathrm{tr}\,}}\chi {\underline{\alpha }}_{AB}{\tilde{{\underline{\beta }}}}^A{\underline{m}}^B +3q{{\,\mathrm{tr}\,}}{\underline{\chi }}({\tilde{{\underline{\beta }}}},{\underline{m}}) \rho \nonumber \\&\quad -3q{{\,\mathrm{tr}\,}}{\underline{\chi }}({\tilde{{\underline{\beta }}}},{}^*{\underline{m}})\sigma -\frac{1}{4}\bigl (6q^{-1}{\hat{\omega }}+6q{\hat{{\underline{\omega }}}}+3q{{\,\mathrm{tr}\,}}{\underline{\chi }}+q^{-1}{{\,\mathrm{tr}\,}}\chi \nonumber \\&\quad +7\hat{{L}}q^{-1}-\hat{{\underline{L}}}q\bigr ){\underline{\alpha }}_{AB}{\tilde{{\underline{\beta }}}}^A m^B \end{aligned}$$

5.42e $$\begin{aligned}&R_{{\underline{\Xi }}}^1 := \frac{2}{\Omega }{\tilde{{\underline{\alpha }}}}_{AB}{\tilde{{\underline{\beta }}}}^A m^B+{\tilde{{\underline{\beta }}}}^A{\underline{m}}^B\Bigl \{4\nabla \!\!\!\!/_B{\underline{\beta }}_A-q^{-1}{\hat{\chi }}_B^{C}{\underline{\alpha }}_{AC}\nonumber \\&\quad +\,2((\zeta +\mathrm {d}\!\!\!/\log q+2{\underline{\eta }}){\hat{\otimes }}{\underline{\beta }})_{AB}\nonumber \\&\quad -\,2(\nabla \!\!\!\!/{\hat{\otimes }}{\underline{\beta }})_{AB}-((4{\underline{\eta }}-\zeta -\mathrm {d}\!\!\!/\log q){\hat{\otimes }}\beta )_{AB}-\bigl ((4{\underline{\eta }}+3\zeta +\mathrm {d}\!\!\!/\log q){\hat{\otimes }}{\underline{\beta }}\bigr )_{AB}\Bigr \}\nonumber \\&\quad -\,2{\tilde{{\underline{\beta }}}}^{A}{\hat{i}}^{BC}\Bigl \{\nabla \!\!\!\!/_C{\underline{\alpha }}_{AB}+(q{\hat{{\underline{\chi }}}}-3q^{-1}{\hat{\chi }})_{CA}{\underline{\beta }}_B+(q{\hat{{\underline{\chi }}}}+3q^{-1}{\hat{\chi }})_{CB}{\underline{\beta }}_A\nonumber \\&\quad -\,(q{\hat{{\underline{\chi }}}}-3q^{-1}{\hat{\chi }})_C^{D}{\underline{\beta }}_Dg\!\!\!/_{AB}\nonumber \\&\quad -\,2(\zeta +\mathrm {d}\!\!\!/\log q)_C{\underline{\alpha }}_{AB} -3q{\hat{{\underline{\chi }}}}_{CA}\beta _B+3q{\hat{{\underline{\chi }}}}_{CB}\beta _A+3g\!\!\!/_{AB}q{\hat{{\underline{\chi }}}}_C^{D}\beta _D\Bigr \}\nonumber \\&\quad +\,3({\tilde{{\underline{\beta }}}},{\underline{m}})\Bigl \{\frac{3}{2}(q{{\,\mathrm{tr}\,}}{\underline{\chi }}-q^{-1}{{\,\mathrm{tr}\,}}\chi )\rho +{{\,\mathrm{div}\,}}\!\!\!\!\!/\,\beta +{{\,\mathrm{div}\,}}\!\!\!\!\!/\,{\underline{\beta }}\nonumber \\&\quad +\,(2{\underline{\eta }}+\zeta +\mathrm {d}\!\!\!/\log q,\beta )-(2\eta -\zeta -\mathrm {d}\!\!\!/\log q,\beta )-\frac{1}{2}(q{\hat{{\underline{\chi }}}},\alpha )+\frac{1}{2}(q^{-1}{\hat{\chi }},{\underline{\alpha }})\Bigr \}\nonumber \\&\quad -\,3({\tilde{{\underline{\beta }}}},{}^*{\underline{m}})\Bigl \{\frac{3}{2}(q{{\,\mathrm{tr}\,}}{\underline{\chi }}-q^{-1}{{\,\mathrm{tr}\,}}\chi )\sigma -{{\,\mathrm{curl}\,}}\!\!\!\!\!/\,\beta +{{\,\mathrm{curl}\,}}\!\!\!\!\!/\,{\underline{\beta }}\nonumber \\&\quad -\,(2{\underline{\eta }}+\zeta +\mathrm {d}\!\!\!/\log q)\wedge \beta +(2\eta -\zeta -\mathrm {d}\!\!\!/\log q)\wedge {\underline{\beta }}+\frac{1}{2}q{\hat{{\underline{\chi }}}}\wedge \alpha +\frac{1}{2}q^{-1}{\hat{\chi }}\wedge {\underline{\alpha }}\Bigr \}\nonumber \\&\quad -\,6{\tilde{{\underline{\beta }}}}^A{\underline{m}}^B\Bigl \{{\underline{\eta }}_A\beta _B-{\underline{\eta }}_B\beta _A+g\!\!\!/_{AB}({\underline{\eta }},\beta )+\eta _A{\underline{\beta }}_B-\eta _B{\underline{\beta }}_A+g\!\!\!/_{AB}(\eta ,{\underline{\beta }})\Bigr \}\nonumber \\&\quad +\,3{\tilde{{\underline{\beta }}}}^Am^B\Bigl \{2(\zeta +\mathrm {d}\!\!\!/\log q)_B{\underline{\beta }}_A-q^{-1}{\hat{\chi }}_B^{C}{\underline{\alpha }}_{AC}\Bigr \}\nonumber \\&\quad -\,6{\tilde{{\underline{\beta }}}}^A{\hat{i}}_A^B\mathrm {d}\!\!\!/_B\rho +6{\tilde{{\underline{\beta }}}}^A{\hat{i}}_A^{B}\mathrm {d}\!\!\!/_B\sigma \end{aligned}$$

5.43a $$\begin{aligned} \boxed { 8({\tilde{\beta }}_q,{\tilde{I}}^1-{\tilde{\Xi }}^1) + 16({\tilde{\beta }}_q,{\tilde{I}}^1) = 2 \frac{2}{\Omega } \Bigl (j+\frac{{\underline{n}}+n}{4}+\frac{2j+{\underline{n}}}{2}\Bigr )|{\tilde{\beta }}_q |^2 + X_\Xi ^1 +Q_{\Xi }^1+Y_{\Xi }^1+R_{\Xi }^1}\nonumber \\ \end{aligned}$$

5.43b $$\begin{aligned}&X_\Xi ^1:=-\Bigl (j+\frac{{\underline{n}}+n}{4}+\frac{2j+{\underline{n}}}{2}\Bigr )\frac{1}{2}\bigl (2q{\hat{{\underline{\omega }}}}+10q^{-1}{\hat{\omega }}+3q{{\,\mathrm{tr}\,}}{\underline{\chi }}\nonumber \\&\qquad \quad \ +\,3q{{\,\mathrm{tr}\,}}\chi -\hat{{\underline{L}}}q+5\hat{{L}}q\bigr )({\tilde{\beta }}_q,\beta _q)+jq^{-1}{{\,\mathrm{tr}\,}}\chi ({\tilde{\beta }}_q,\beta _q)\nonumber \\&\qquad \quad \ +\Bigl (\frac{{\underline{n}}-n}{2}+2j-{\underline{n}}\Bigr )\bigl (q{{\,\mathrm{tr}\,}}{\underline{\chi }}-2 q^{-1}{{\,\mathrm{tr}\,}}\chi \bigr )({\tilde{\beta }}_q,\beta _q) \end{aligned}$$

5.43c $$\begin{aligned}&Q_{\Xi }^1:=\Bigl (j+\frac{{\underline{n}}+n}{4}+\frac{2j+{\underline{n}}}{2}\Bigr )\Bigl ({\tilde{\beta }}_q,-3\bigl (\eta +{\underline{\eta }}-\mathrm {d}\!\!\!/\log q\bigr )\rho \nonumber \\&\quad -\,3{}^*\bigl (\eta +{\underline{\eta }}-\mathrm {d}\!\!\!/\log q\bigr )\sigma -\alpha ^\sharp _q\cdot \bigl (\eta +{\underline{\eta }}+\mathrm {d}\!\!\!/\log q\bigr )\Bigr )\nonumber \\&\quad +\Bigl (\frac{{\underline{n}}-n}{2}+2j-{\underline{n}}\Bigr )\Bigl ({\tilde{\beta }}_q,{{\,\mathrm{div}\,}}\!\!\!\!\!/\,\alpha _q-\mathrm {d}\!\!\!/\rho -{}^*\mathrm {d}\!\!\!/\sigma \nonumber \\&\quad +\,2\bigl (\zeta +\mathrm {d}\!\!\!/\log q\bigr )^\sharp \cdot {\underline{\alpha }}_q-2q^{-1}{\hat{\chi }}^\sharp \cdot {\underline{\beta }}_q\Bigr ) \end{aligned}$$

5.43d $$\begin{aligned}&Y_{\Xi }^1 := 6q^{-1}{{\,\mathrm{tr}\,}}\chi {\tilde{\beta }}^A{\hat{i}}_A^{B}{\underline{\beta }}_B +\frac{1}{2}q{{\,\mathrm{tr}\,}}{\underline{\chi }}\alpha _{AB}{\tilde{\beta }}^Am^B\nonumber \\&\quad +\,3q^{-1}{{\,\mathrm{tr}\,}}\chi ({\tilde{\beta }},m) \rho -3q^{-1}{{\,\mathrm{tr}\,}}\chi ({\tilde{\beta }},{}^*m)\sigma \nonumber \\&\quad -\frac{1}{4}\bigl (6q{\hat{{\underline{\omega }}}}+6q^{-1}{\hat{\omega }}+3q^{-1}{{\,\mathrm{tr}\,}}\chi +q{{\,\mathrm{tr}\,}}{\underline{\chi }}+7\hat{{\underline{L}}}q-\hat{{L}}q^{-1}\bigr )\alpha _{AB}{\tilde{\beta }}^A {\underline{m}}^B \end{aligned}$$

5.43e $$\begin{aligned}&R_{\Xi }^1 :=\frac{2}{\Omega }{\tilde{\alpha }}_{AB}{\tilde{\beta }}^A {\underline{m}}^B+ {\tilde{\beta }}^Am^B\Bigl \{4\nabla \!\!\!\!/_B\beta _A-q{\hat{{\underline{\chi }}}}_B^{C}\alpha _{AC}\nonumber \\&\quad -\,2((-\zeta -\mathrm {d}\!\!\!/\log q+2\eta ){\hat{\otimes }}\beta )_{AB}\nonumber \\&\quad -2(\nabla \!\!\!\!/{\hat{\otimes }}\beta )_{AB}+((4\eta -\zeta -\mathrm {d}\!\!\!/\log q){\hat{\otimes }}{\underline{\beta }})_{AB}\nonumber \\&\quad +\,\bigl ((4\eta +3\zeta +\mathrm {d}\!\!\!/\log q){\hat{\otimes }}\beta \bigr )_{AB}\Bigr \}\nonumber \\&\quad -2{\tilde{\beta }}^{A}{\hat{i}}^{BC}\Bigl \{\nabla \!\!\!\!/_C\alpha _{AB}+(q^{-1}{\hat{\chi }}-3q{\hat{{\underline{\chi }}}})_{CA}\beta _B+(q^{-1}{\hat{\chi }}+3q{\hat{{\underline{\chi }}}})_{CB}\beta _A\nonumber \\&\quad -\,(q^{-1}{\hat{\chi }}-3q{\hat{{\underline{\chi }}}})_C^{D}\beta _Dg\!\!\!/_{AB}\nonumber \\&\quad +\,2(\zeta +\mathrm {d}\!\!\!/\log q)_C\alpha _{AB} -3q^{-1}{\hat{\chi }}_{CA}{\underline{\beta }}_B+3q^{-1}{\hat{\chi }}_{CB}{\underline{\beta }}_A+3g\!\!\!/_{AB}q^{-1}{\hat{\chi }}_C^{D}{\underline{\beta }}_D\Bigr \}\nonumber \\&\quad +3({\tilde{\beta }},m)\Bigl \{-\frac{3}{2}(q{{\,\mathrm{tr}\,}}{\underline{\chi }}-q^{-1}{{\,\mathrm{tr}\,}}\chi )\rho +{{\,\mathrm{div}\,}}\!\!\!\!\!/\,{\underline{\beta }}+{{\,\mathrm{div}\,}}\!\!\!\!\!/\,\beta \nonumber \\&\quad -\,(2{\underline{\eta }}-\zeta -\mathrm {d}\!\!\!/\log q,{\underline{\beta }})+(2{\underline{\eta }}+\zeta +\mathrm {d}\!\!\!/\log q,\beta )-\frac{1}{2}(q^{-1}{\hat{\chi }},{\underline{\alpha }})+\frac{1}{2}(q{\hat{{\underline{\chi }}}},\alpha )\Bigr \}\nonumber \\&\quad -3({\tilde{\beta }},{}^*m)\Bigl \{-\frac{3}{2}(q{{\,\mathrm{tr}\,}}{\underline{\chi }}-q^{-1}{{\,\mathrm{tr}\,}}\chi )\sigma -{{\,\mathrm{curl}\,}}\!\!\!\!\!/\,{\underline{\beta }}+{{\,\mathrm{curl}\,}}\!\!\!\!\!/\,\beta \nonumber \\&\quad +\,(2\eta -\zeta -\mathrm {d}\!\!\!/\log q)\wedge {\underline{\beta }}-(2{\underline{\eta }}+\zeta +\mathrm {d}\!\!\!/\log q)\wedge \beta +\frac{1}{2}q^{-1}{\hat{\chi }}\wedge {\underline{\alpha }}+\frac{1}{2}q{\hat{{\underline{\chi }}}}\wedge \alpha \Bigr \}\nonumber \\&\quad -6{\tilde{\beta }}^Am^B\Bigl \{\eta _A{\underline{\beta }}_B-\eta _B{\underline{\beta }}_A+g\!\!\!/_{AB}(\eta ,{\underline{\beta }})+{\underline{\eta }}_A\beta _B-{\underline{\eta }}_B\beta _A+g\!\!\!/_{AB}({\underline{\eta }},\beta )\Bigr \}\nonumber \\&\quad +3{\tilde{\beta }}^A{\underline{m}}^B\Bigl \{-2(\zeta +\mathrm {d}\!\!\!/\log q)_B\beta _A-q{\hat{{\underline{\chi }}}}_B^{C}\alpha _{AC}\Bigr \} +6{\tilde{\beta }}^A{\hat{i}}_A^B\mathrm {d}\!\!\!/_B\rho +6{\tilde{\beta }}^A{\hat{i}}_A^{B}\mathrm {d}\!\!\!/_B\sigma \nonumber \\ \end{aligned}$$

5.44a $$\begin{aligned} \boxed { 4({\tilde{{\underline{\alpha }}}}_q,\tilde{{\underline{\Theta }}}^1) = \frac{n}{\Omega } |{\tilde{{\underline{\alpha }}}}_q |^2 +X_{{\underline{\Theta }}}^1 + Q_{{\underline{\Theta }}}^1+Y_{{\underline{\Theta }}}^1+R_{{\underline{\Theta }}}^1 } \end{aligned}$$

5.44b $$\begin{aligned}&X_{{\underline{\Theta }}}^1 := -\frac{1}{4} \frac{n}{2} \bigl (-2q^{-1}{\hat{\omega }}+14q{\hat{{\underline{\omega }}}}+3q{{\,\mathrm{tr}\,}}{\underline{\chi }}+3q^{-1}{{\,\mathrm{tr}\,}}\chi -\hat{{L}}q^{-1}+7\hat{{\underline{L}}}q\bigr )({\tilde{{\underline{\alpha }}}}_q,{\underline{\alpha }}_q)\nonumber \\&\quad -\frac{2j-n}{4} q^{-1}{{\,\mathrm{tr}\,}}\chi ({\tilde{{\underline{\alpha }}}}_q,{\underline{\alpha }}_q) \end{aligned}$$

5.44c $$\begin{aligned}&Q_{{\underline{\Theta }}}^1 := n\Bigl ({\tilde{{\underline{\alpha }}}}_q,\bigl (\eta +{\underline{\eta }}+\mathrm {d}\!\!\!/\log q\bigr ){\hat{\otimes }}{\underline{\beta }}_q\Bigr ) -\frac{2j-n}{2}\Bigl ({\tilde{{\underline{\alpha }}}}_q,\bigl (\zeta +\mathrm {d}\!\!\!/\log q\bigr )\nonumber \\&\quad {\hat{\otimes }}{\underline{\beta }}_q+\nabla \!\!\!\!/{\hat{\otimes }}{\underline{\beta }}_q +3q{\hat{{\underline{\chi }}}}\rho -3q{}^*{\hat{{\underline{\chi }}}}\sigma \Bigr ) \end{aligned}$$

5.44d $$\begin{aligned}&Y_{{\underline{\Theta }}}^1 := -\frac{3}{2}q{{\,\mathrm{tr}\,}}{\underline{\chi }}\bigl ({\tilde{{\underline{\alpha }}}},{\hat{i}}\bigr )\rho +\frac{3}{2}q{{\,\mathrm{tr}\,}}{\underline{\chi }}\bigl ({\tilde{{\underline{\alpha }}}},{}^*{\hat{i}}\bigr )\sigma -\frac{1}{4}q{{\,\mathrm{tr}\,}}{\underline{\chi }}\Bigl ({\tilde{{\underline{\alpha }}}},m{\hat{\otimes }}{\underline{\beta }}_q\Bigr ) \end{aligned}$$

5.44e $$\begin{aligned}&R_{{\underline{\Theta }}}^1 := -\frac{1}{2}\biggl ({\tilde{{\underline{\alpha }}}},{\underline{m}}{\hat{\otimes }}\Bigl (\bigl (q^{-1}{{\,\mathrm{tr}\,}}\chi -q{{\,\mathrm{tr}\,}}{\underline{\chi }}\bigr ){\underline{\beta }}_q+\mathrm {d}\!\!\!/\rho -{}^*\mathrm {d}\!\!\!/\sigma -2q^{-1}{\hat{\chi }}^\sharp \cdot {\underline{\beta }}\Bigr )\biggr )\nonumber \\&\quad -\frac{1}{2}\biggl ({\tilde{{\underline{\alpha }}}},m{\hat{\otimes }}\Bigl ({{\,\mathrm{div}\,}}\!\!\!\!\!/\,{\underline{\alpha }}_q+({\underline{\eta }}-2\zeta -2\mathrm {d}\!\!\!/\log q)^\sharp \cdot {\underline{\alpha }}_q\Bigr )\biggr )\nonumber \\&\quad -\frac{1}{2}\biggl ({\tilde{{\underline{\alpha }}}},{\underline{\eta }}{\hat{\otimes }}\Bigl (m^\sharp \cdot {\underline{\alpha }}_q\Bigr )\biggr )+\frac{1}{2}\bigl (m,\eta +2\mathrm {d}\!\!\!/\log q)\bigl ({\tilde{{\underline{\alpha }}}},{\underline{\alpha }}_q\bigr )\nonumber \\&\quad -\frac{1}{2}{\tilde{{\underline{\alpha }}}}^{AB}m^C\Bigl \{\nabla \!\!\!\!/_C{\underline{\alpha }}_{AB}+(q{\hat{{\underline{\chi }}}}{\hat{\otimes }}{\underline{\beta }}_q)_{CAB}\Bigr \} -2{\tilde{{\underline{\alpha }}}}^{AB}{\hat{i}}_{A}^{D}\nabla \!\!\!\!/_D{\underline{\beta }}_B \nonumber \\&\quad +{\tilde{{\underline{\alpha }}}}^{AB}{\hat{i}}^{CD}q^{-1}{\hat{\chi }}_{CA}{\underline{\alpha }}_{DB} -\bigl ({\hat{i}},q^{-1}{\hat{\chi }}\bigr )\bigl ({\tilde{{\underline{\alpha }}}},{\underline{\alpha }}_q)+\frac{1}{2}\Bigl ({\tilde{{\underline{\alpha }}}},\bigl ({\hat{i}}^\sharp \cdot (\zeta +\mathrm {d}\!\!\!/\log q)\bigr ){\hat{\otimes }}{\underline{\beta }}_q\Bigr )\nonumber \\ \end{aligned}$$

5.45a $$\begin{aligned} \boxed { 4({\tilde{\alpha }}_q,{\tilde{\Theta }}^1) = \frac{{\underline{n}}}{\Omega }|{\tilde{\alpha }}_q |^2 +X_\Theta ^1 + Q_\Theta ^1 +Y_{\Theta }^1+R_{\Theta }^1} \end{aligned}$$

5.45b $$\begin{aligned}&X_\Theta ^1 := -\frac{1}{4}\frac{{\underline{n}}}{2}\bigl (-2q{\hat{{\underline{\omega }}}}+14q^{-1}{\hat{\omega }}+3q{{\,\mathrm{tr}\,}}{\underline{\chi }}+3q^{-1}{{\,\mathrm{tr}\,}}\chi -\hat{{\underline{L}}}q+7\hat{{L}}q^{-1}\bigr )({\tilde{\alpha }}_q,\alpha _q)\nonumber \\&\quad -\frac{2j-{\underline{n}}}{4} q{{\,\mathrm{tr}\,}}{\underline{\chi }}({\tilde{\alpha }}_q,\alpha _q) \end{aligned}$$

5.45c $$\begin{aligned}&Q_\Theta ^1 := -{\underline{n}}\Bigl ({\tilde{\alpha }}_q,\bigl (\eta +{\underline{\eta }}-\mathrm {d}\!\!\!/\log q\bigr ){\hat{\otimes }}\beta _q\Bigr )\nonumber \\&\quad +\frac{2j-{\underline{n}}}{2}\Bigl ({\tilde{\alpha }}_q,\nabla \!\!\!\!/{\hat{\otimes }}\beta _q+(\zeta +\mathrm {d}\!\!\!/\log q){\hat{\otimes }}\beta -3q^{-1}{\hat{\chi }}\rho -3q^{-1}{}^*{\hat{\chi }}\sigma \Bigr ) \end{aligned}$$

5.45d $$\begin{aligned}&Y_{\Theta }^1 := -\frac{3}{2}q^{-1}{{\,\mathrm{tr}\,}}\chi \bigl ({\tilde{\alpha }},{\hat{i}}\bigr )\rho +\frac{3}{2}q^{-1}{{\,\mathrm{tr}\,}}\chi \bigl ({\tilde{\alpha }},{}^*{\hat{i}}\bigr )\sigma +\frac{1}{4}q^{-1}{{\,\mathrm{tr}\,}}\chi \Bigl ({\tilde{\alpha }},{\underline{m}}{\hat{\otimes }}\beta _q\Bigr ) \end{aligned}$$

5.45e $$\begin{aligned}&R_{\Theta }^1 := -\frac{1}{2}\biggl ({\tilde{\alpha }},m{\hat{\otimes }}\Bigl (\bigl (q^{-1}{{\,\mathrm{tr}\,}}\chi -q{{\,\mathrm{tr}\,}}{\underline{\chi }}\bigr )\beta _q+\mathrm {d}\!\!\!/\rho +{}^*\mathrm {d}\!\!\!/\sigma +2q^{-1}{\hat{\chi }}^\sharp \cdot {\underline{\beta }}\Bigr )\biggr )\nonumber \\&\quad -\frac{1}{2}\biggl ({\tilde{\alpha }},{\underline{m}}{\hat{\otimes }}\Bigl (-\frac{3}{2}q^{-1}{{\,\mathrm{tr}\,}}\chi \beta _q+{{\,\mathrm{div}\,}}\!\!\!\!\!/\,\alpha _q+({\underline{\eta }}+2\zeta +2\mathrm {d}\!\!\!/\log q)^\sharp \cdot \alpha _q\Bigr )\biggr )\nonumber \\&\quad -\frac{1}{2}\biggl ({\tilde{\alpha }},\eta {\hat{\otimes }}\Bigl ({\underline{m}}^\sharp \cdot \alpha _q\Bigr )\biggr )+\frac{1}{2}\bigl ({\underline{m}},{\underline{\eta }}-2\mathrm {d}\!\!\!/\log q)\bigl ({\tilde{\alpha }},\alpha _q\bigr )\nonumber \\&\quad -\frac{1}{2}{\tilde{\alpha }}^{AB}{\underline{m}}^C\Bigl \{\nabla \!\!\!\!/_C\alpha _{AB}+(q^{-1}{\hat{\chi }}{\hat{\otimes }}\beta _q)_{CAB}\Bigr \} +2{\tilde{\alpha }}^{AB}{\hat{i}}_{A}^{D}\nabla \!\!\!\!/_D\beta _B \nonumber \\&\quad +{\tilde{\alpha }}^{AB}{\hat{i}}^{CD}q{\hat{{\underline{\chi }}}}_{CA}\alpha _{DB} -\bigl ({\hat{i}},q{\hat{{\underline{\chi }}}}\bigr )\bigl ({\tilde{\alpha }},\alpha _q)+\frac{1}{2}\Bigl ({\tilde{\alpha }},\bigl ({\hat{i}}^\sharp \cdot (\zeta +\mathrm {d}\!\!\!/\log q)\bigr ){\hat{\otimes }}\beta _q\Bigr )\nonumber \\ \end{aligned}$$

The full divergence, ${{\,\mathrm{div}\,}}Q[\tilde{{\mathcal {L}}}_{N}W](M_q,M_q,M_q)$, is given — according to (5.7) — by the sum of (5.31a), (5.41a), (5.42a), (5.43a), (5.44a), and (5.45a). After multiplying by $\phi$, the sum of the “principal terms” — namely the first terms in each of the formulas — is bounded by

$$\begin{aligned}&\frac{\phi }{(2\Omega )^3}\biggl [\frac{2}{\Omega }\Bigl ( j + \frac{1}{4}({\underline{n}}+n)\Bigr ) \bigl (2|{\tilde{{\underline{\beta }}}}_q |^2+6{\tilde{\rho }}^2 + 6{\tilde{\sigma }}^2 +2|{\tilde{\beta }}_q |^2\bigr )\\&\quad +\frac{n}{\Omega } |{\tilde{{\underline{\alpha }}}}_q |^2+2 \frac{2}{\Omega } (2j+n) |{\tilde{{\underline{\beta }}}}_q |^2+2 \frac{2}{\Omega } (2j+{\underline{n}})|{\tilde{\beta }}_q |^2+\frac{{\underline{n}}}{\Omega }|{\tilde{\alpha }}_q |^2\biggr ]\le \\&\quad \le \frac{C}{\Omega }\frac{2}{r}\frac{1}{(2\Omega )^3}\Bigl [|{\tilde{{\underline{\alpha }}}}|^2+|{\tilde{{\underline{\beta }}}}|^2+{\tilde{\rho }}^2+{\tilde{\sigma }}^2+|{\tilde{\beta }}|^2+|{\tilde{\alpha }}|^2\Bigr ] \\&\quad \le \frac{1}{\Omega }\frac{C}{r} Q[\tilde{{\mathcal {L}}}_{N}W](n,M_q,M_q,M_q) \end{aligned}$$

where on the right hand side *n* denotes the normal vector (4.74), and we used — the lower bound was already used in the prooof of Lemma 4.14 — that

5.46a $$\begin{aligned} \phi \, q^{-1}{{\,\mathrm{tr}\,}}\chi= & {} \frac{2}{r}\frac{\Omega {{\,\mathrm{tr}\,}}\chi }{\overline{\Omega {{\,\mathrm{tr}\,}}\chi }}\le \frac{2}{r}(1+C_0\Omega ^{-1}) \end{aligned}$$

5.46b $$\begin{aligned} \phi \, q{{\,\mathrm{tr}\,}}{\underline{\chi }}= & {} \frac{2}{r}\frac{\Omega {{\,\mathrm{tr}\,}}{\underline{\chi }}}{\overline{\Omega {{\,\mathrm{tr}\,}}{\underline{\chi }}}}\le \frac{2}{r}(1+C_0\Omega ^{-1}) \end{aligned}$$

Moreover, it then follows from (5.31h), (5.41c), (5.42c), (5.43c), (5.44c), and (5.45c) that

5.47 $$\begin{aligned}&\frac{\phi }{(2\Omega )^3}\Bigl [ |Q_\Lambda ^1|+ |Q_\Lambda ^1|+|Q_{{\underline{\Xi }}}^1|^2+|Q_{\Xi }^1|^2+|Q_{{\underline{\Theta }}}|^2+|Q_{\Theta }|^2\Bigr ]\nonumber \\&\quad \le \frac{C}{\Omega r} \Bigl (Q[\tilde{{\mathcal {L}}}_{N}W](n,M_q,M_q,M_q)+ Q[W](n,M_q,M_q,M_q)\Bigr )\nonumber \\&\quad +\frac{C}{\Omega r}\frac{1}{(2\Omega )^3}\Bigl [\Omega ^2|\nabla \!\!\!\!/{\underline{\alpha }}_q|^2+\Omega ^2|\nabla \!\!\!\!/{\underline{\beta }}_q|^2+\Omega ^2|\nabla \!\!\!\!/\rho |^2\nonumber \\&\quad +\,\Omega ^2|\nabla \!\!\!\!/\sigma |^2+\Omega ^2|\nabla \!\!\!\!/\beta _q|^2+\Omega ^2|\alpha _q|^2\Bigr ] \end{aligned}$$

Similarly for the terms $R_\Lambda ^1$, $R_K^1$, $R_{{\underline{\Xi }}}^1$, $R_{\Xi }^1$, $R_{{\underline{\Theta }}}^1$, $R_{\Theta }^1$. $\square$

#### $J^2$, and $J^3$

Here we analyse the currents $J^2$, and $J^3$. They contain “lower order” terms at the level of *W*, but also factors at the level of $\nabla \pi$. We are interested in their precise structure to show cancellations with “lower order” terms from $J^1$, and control the remainder with assumptions on $\nabla \Gamma$.

Recall the notation

$$\begin{aligned} p_\beta =\nabla ^\alpha \,{}^{(N)}{\hat{\pi }}_{\alpha \beta }\qquad p_3=p_\beta e_3^\beta \qquad p_4=p_\beta e_4^\beta \,, \end{aligned}$$

and also that we denote by *d* the 3-form

$$\begin{aligned} d_{\alpha \beta \gamma }=\nabla _\beta \, {}^{(N)}{\hat{\pi }}_{\gamma \alpha }-\nabla _\gamma \,{}^{(N)}{\hat{\pi }}_{\beta \alpha }+\frac{1}{3}\Bigl (p_\beta g_{\alpha \gamma }-p_\gamma g_{\alpha \beta }\Bigr ) \end{aligned}$$

which has the algebraic properties of a Weyl current, and can be decomposed into the $S_{u,v}$-1-forms ${\underline{\Xi }}(d)$, $\Xi (d)$, the functions ${\underline{\Lambda }}(d)$, $\Lambda (d)$, ${\underline{K}}(d)$, *K*(*d*), and the $S_{u,v}$ 2-forms ${\underline{\Theta }}(d)$, $\Theta (d)$; cf. (12.51-61) in [8].

In order to control the derivatives of the deformation tensor of *N* we introduce the following assumptions: 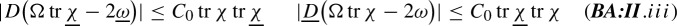

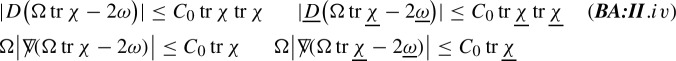

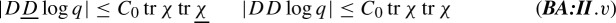

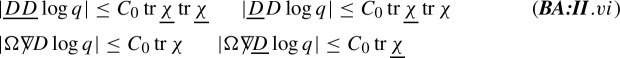

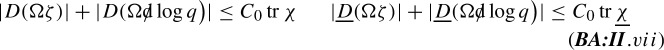

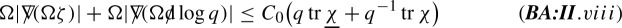


##### Remark 5.14

In the context of the *bootstrap argument* the assumptions (***BA:II***) should be formulated in the $\mathrm {L}^4(S_{u,v})$ norm, but we state them here for simplicity as $\mathrm {L}^\infty$ assumptions. While the scaling and weights are identical to the appropriate assumptions in $\mathrm {L}^4$, we will proceed here for convenience with estimating all quantities pointwise, as opposed to also considering integrations on $S_{u,v}$.

##### Lemma 5.15

Assume that (**BA:I**.i-viii) hold for some $C_0>0$. Then,

5.49 $$\begin{aligned}&\phi \Bigl [ \bigl ({{\,\mathrm{div}\,}}Q(\tilde{{\mathcal {L}}}_{N}W)\bigr )(M_q,M_q,M_q) \Bigr ]^{2}+ \phi \Bigl [ \bigl ({{\,\mathrm{div}\,}}Q(\tilde{{\mathcal {L}}}_{N}W)\bigr )(M_q,M_q,M_q) \Bigr ]^{2}\mathrm {d}\mu _{{\overline{g}}_{r}}\ge \nonumber \\&\quad \ge \frac{\phi }{(2\Omega )^3}\bigl [X^{2+3}+Y^{2+3}\bigr ]\nonumber \\&\quad -\frac{C}{r}\frac{1}{\Omega }Q[\tilde{{\mathcal {L}}}_{N}W](n,M_q,M_q,M_q) \!-\!\frac{C}{r}\frac{1}{\Omega }Q[W](n,M_q,M_q,M_q) \!-\!\frac{C}{r}\frac{1}{\Omega }\Bigl [\Omega ^2{P\!\!\!\!/\,}^q\Bigr ]\nonumber \\ \end{aligned}$$

where

5.50a $$\begin{aligned}&X^{2+3}:=X_\Lambda ^{2+3}+X_K^{2+3}+X_{{\underline{\Xi }}}^{2+3}+X_{\Xi }^{2+3}+X_{{\underline{\Theta }}}^{2+3}+X_{\Theta }^{2+3} \end{aligned}$$

5.50b $$\begin{aligned}&Y^{2+3}:=X_\Lambda ^{2+3}+Y_K^{2+3}+Y_{{\underline{\Xi }}}^{2+3}+Y_{\Xi }^{2+3}+Y_{{\underline{\Theta }}}^{2+3}+Y_{\Theta }^{2+3} \end{aligned}$$

and $X_\Lambda ^{2+3}$, $X_K^{2+3}$, $X_{{\underline{\Theta }}}^{2+3}$, $X_{\Theta }^{2+3}$, are given by (5.54b), (5.55b), (5.57b), (5.56b), respectively, while $Y_\Lambda ^{2+3}$, $Y_K^{2+3}$, $Y_{{\underline{\Theta }}}^{2+3}$, $Y_{\Theta }^{2+3}$, are given by (5.54c), (5.55c), (5.57c), (5.56c), respectively.

##### Proof

For the null components of ${}^{(N)}J^2[W]$, we directly refer to (14.98) in [8]:

5.51a $$\begin{aligned} 8{\tilde{\Lambda }}^2+8\tilde{{\underline{\Lambda }}}^2= & {} -2(p_3+p_4)\rho +2(p\!\!\!/\,,\beta _q-{\underline{\beta }}_q) \end{aligned}$$

5.51b $$\begin{aligned} 8{\tilde{K}}^2-8\tilde{{\underline{K}}}^2= & {} -2(p_4+p_3)\sigma -2p\!\!\!/\,\wedge (\beta _q+{\underline{\beta }}_q) \end{aligned}$$

5.51c $$\begin{aligned} 4{\tilde{\Theta }}^2= & {} -p_3\alpha _q+p\!\!\!/\,{\hat{\otimes }}\beta _q \end{aligned}$$

5.51d $$\begin{aligned} 4\tilde{{\underline{\Theta }}}^2= & {} -p_4{\underline{\alpha }}_q-p\!\!\!/\,{\hat{\otimes }}{\underline{\beta }}_q \end{aligned}$$

5.51e $$\begin{aligned} 8(\tilde{{\underline{\Xi }}}^2-3\tilde{{\underline{I}}}^2)= & {} 2p_3{\underline{\beta }}_q-2p\!\!\!/\,^\sharp \cdot {\underline{\alpha }}_q-6p_4{\underline{\beta }}_q-6p\!\!\!/\,\rho +{}^*p\!\!\!/\,\sigma \end{aligned}$$

5.51f $$\begin{aligned} 8(-{\tilde{\Xi }}^2+3{\tilde{I}}^2)= & {} 2p_4\beta _q+2p\!\!\!/\,^\sharp \cdot \alpha _q-6p_3\beta _q+6p\!\!\!/\,\rho +6{}^*p\!\!\!/\,\sigma \end{aligned}$$

We do not write out the expressions for *p* at this point, due to cancellations with contributions from $J^3$.

For the null components of ${}^{(N)}J^3[W]$ we have Lemma 14.2 in [8]:

5.52a $$\begin{aligned}&8{\tilde{\Lambda }}^3+8\tilde{{\underline{\Lambda }}}^3=\bigl ({\underline{\Theta }}(d),\alpha _q\bigr )+\bigl (\Theta (d),{\underline{\alpha }}_q\bigr )-3\bigl (2\Lambda (d)+2{\underline{\Lambda }}(d)\bigr )\rho \nonumber \\&\quad +\,3\bigl (2K(d)-2{\underline{K}}(d)\bigr )\sigma +2\bigl (\Xi (d),{\underline{\beta }}_q\bigr )-2\bigl ({\underline{\Xi }}(d),\beta _q\bigr ) \end{aligned}$$

5.52b $$\begin{aligned}&8{\tilde{K}}^3-8\tilde{{\underline{K}}}^3=-{\underline{\Theta }}(d)\wedge \alpha _q+\Theta (d)\wedge {\underline{\alpha }}_q-3\bigl (2\Lambda (d)+2{\underline{\Lambda }}(d)\bigr )\sigma \nonumber \\&\quad -\,3\bigl (2K(d)-2{\underline{K}}(d)\bigr )\rho +2\Xi (d)\wedge {\underline{\beta }}_q+2{\underline{\Xi }}(d)\wedge \beta _q \end{aligned}$$

5.52c $$\begin{aligned}&4{\tilde{\Theta }}^3 = 3 {\underline{\Lambda }}(d) \alpha _q - 3{\underline{K}}(d){}^*\alpha _q-6 I(d){\hat{\otimes }}\beta +3\Theta (d)\rho +3{}^*\Theta (d)\sigma \end{aligned}$$

5.52d $$\begin{aligned}&4\tilde{{\underline{\Theta }}}^3 = 3 \Lambda (d) {\underline{\alpha }}_q - 3 K(d){}^*{\underline{\alpha }}_q + 6 {\underline{I}}(d){\hat{\otimes }}{\underline{\beta }}+ 3{\underline{\Theta }}(d)\rho -3{}^*{\underline{\Theta }}(d)\sigma \end{aligned}$$

5.52e $$\begin{aligned}&8\bigl (\tilde{{\underline{\Xi }}}^3-3\tilde{{\underline{I}}}^3\bigr )=6(\Xi (d)-I(d))^\sharp \cdot {\underline{\alpha }}_q-12{\underline{\Lambda }}(d){\underline{\beta }}_q-12{\underline{K}}(d){}^*{\underline{\beta }}_q\nonumber \\&\quad +\,6({\underline{\Xi }}(d)+3{\underline{I}}(d))\rho -6{}^*({\underline{\Xi }}(d)+3{\underline{I}}(d))\sigma -12{\underline{\Theta }}(d)\cdot \beta _q^\sharp \end{aligned}$$

5.52f $$\begin{aligned}&8\bigl (-{\tilde{\Xi }}^3+3I^3\bigr )=+6({\underline{I}}(d)-{\underline{\Xi }}(d))^\sharp \cdot \alpha _q-12\Lambda (d)\beta _q-12K(d){}^*\beta _q\nonumber \\&\quad -6(\Xi (d)+3I(d))\rho -3{}^*(\Xi (d)+3I(d))\sigma -12\Theta (d)\cdot {\underline{\beta }}_q^\sharp \end{aligned}$$

Now most importantly,

$$\begin{aligned}&2\Lambda (d)=\frac{1}{2}d_{434}=\frac{1}{2}\nabla _3{}^{(N)}{\hat{\pi }}_{44}-\frac{1}{2}\nabla _4{}^{(N)}{\hat{\pi }}_{34}+\frac{1}{3}p_4\\&\quad =-\nabla _4{}^{(N)}{\hat{\pi }}_{34}+g^{AC}\nabla _C{}^{(N)}{\hat{\pi }}_{A4}-\frac{2}{3}p_4\\ 2{\underline{\Lambda }}(d)= & {} \frac{1}{2}d_{343}=-\nabla _3{}^{(N)}{\hat{\pi }}_{34}+g^{AC}\nabla _C{}^{(N)}{\hat{\pi }}_{A3}-\frac{2}{3}p_3 \end{aligned}$$

where we used that

$$\begin{aligned} p_4= & {} \nabla ^\alpha {}^{(N)}{\hat{\pi }}_{\alpha 4}=-\frac{1}{2}\nabla _4{}^{(N)}{\hat{\pi }}_{34}-\frac{1}{2}\nabla _3{}^{(N)}{\hat{\pi }}_{44}+\nabla ^A{}^{(N)}{\hat{\pi }}_{A4}\\ p_3= & {} -\frac{1}{2}\nabla _4{}^{(N)}{\hat{\pi }}_{33}-\frac{1}{2}\nabla _3{}^{(N)}{\hat{\pi }}_{43}+\nabla ^A{}^{(N)}{\hat{\pi }}_{A3} \end{aligned}$$

Moreover,

$$\begin{aligned} 2\Xi _A(d)= & {} \Omega ^{-1}q^{-1}Dm+q^{-1}\chi ^\sharp \cdot m-\bigl (q^{-1}{\hat{\omega }}+\hat{{L}}q^{-1}\bigr )m-\nabla \!\!\!\!/_A n-\bigl (\eta +\mathrm {d}\!\!\!/\log q\bigr )n\\ 2{\underline{\Xi }}_A(d)= & {} \Omega ^{-1}q{\underline{D}}{\underline{m}}+q{\underline{\chi }}^\sharp \cdot {\underline{m}}-\bigl (q{\hat{{\underline{\omega }}}}+\hat{{\underline{L}}}q\bigr ){\underline{m}}-\nabla \!\!\!\!/_A{\underline{n}}+\bigl (\eta +\mathrm {d}\!\!\!/\log q\bigr ){\underline{n}}\\ 2\Theta (d)= & {} 2q^{-1}\Omega ^{-1}{\hat{D}}{\hat{i}}-q^{-1}{{\,\mathrm{tr}\,}}\chi {\hat{i}}-\nabla \!\!\!\!/{\hat{\otimes }}m+2q^{-1}{\hat{\chi }}j+q{\hat{{\underline{\chi }}}}n\\&\quad -\,\bigl (2{\underline{\eta }}+\zeta +\mathrm {d}\!\!\!/\log q\bigr ){\hat{\otimes }}m\\ 2{\underline{\Theta }}(d)= & {} 2q\Omega ^{-1}{\hat{{\underline{D}}}}{\hat{i}}-{{\,\mathrm{tr}\,}}{\underline{\chi }}{\hat{i}}-\nabla \!\!\!\!/{\hat{\otimes }}{\underline{m}}+2q{\hat{{\underline{\chi }}}}j+q^{-1}{\hat{\chi }}{\underline{n}}-\bigl (2{\underline{\eta }}-\zeta -\mathrm {d}\!\!\!/\log q\bigr ){\hat{\otimes }}{\underline{m}}\\ 2K(d)= & {} {{\,\mathrm{curl}\,}}\!\!\!\!\!/\,m+ q^{-1}{\hat{\chi }}\wedge {\hat{i}}+\bigl (\zeta +\mathrm {d}\!\!\!/\log q\bigr )\wedge m\\ 2{\underline{K}}(d)= & {} {{\,\mathrm{curl}\,}}\!\!\!\!\!/\,{\underline{m}}+ q {\hat{{\underline{\chi }}}}\wedge {\hat{i}}-\bigl (\zeta +\mathrm {d}\!\!\!/\log q\bigr )\wedge {\underline{m}}\\ 2I(d)= & {} q^{-1}\Omega ^{-1}D{\underline{m}}-\bigl (q^{-1}{\hat{\omega }}+\hat{{L}}q^{-1}\bigr ){\underline{m}}-\mathrm {d}\!\!\!/j-2{\underline{\eta }}j-2{\underline{\eta }}^\sharp \cdot {\hat{i}}+{\underline{\chi }}^\sharp \cdot m+\frac{2}{3}p\!\!\!/\,\\ 2{\underline{I}}(d)= & {} q\Omega ^{-1}{\underline{D}}m-\bigl (q{\hat{{\underline{\omega }}}}+\hat{{\underline{L}}}q\bigr )m-\mathrm {d}\!\!\!/j-2\eta j-2\eta ^\sharp \cdot {\hat{i}}+\chi ^\sharp \cdot {\underline{m}}+\frac{2}{3}p\!\!\!/\,\end{aligned}$$

Note also,

$$\begin{aligned} p\!\!\!/\,= & {} -\frac{1}{2}\bigl (q^{-1}\Omega ^{-1}D{\underline{m}}+q\Omega ^{-1}{\underline{D}}m\bigr )+{{\,\mathrm{div}\,}}\!\!\!\!\!/\,{\hat{i}}+\frac{1}{2}\mathrm {d}\!\!\!/j +\bigl ({\underline{\eta }}+\eta \bigr )^\sharp \cdot {\hat{i}}+\bigl (\eta +{\underline{\eta }}\bigr )j\\&\quad -\,\frac{1}{2}\bigl (q^{-1}{\hat{\omega }}+\hat{{L}}q^{-1}+q^{-1}{{\,\mathrm{tr}\,}}\chi \bigr ){\underline{m}}-\frac{1}{2}\bigl (q{\hat{{\underline{\omega }}}}+\hat{{\underline{L}}}q+q{{\,\mathrm{tr}\,}}{\underline{\chi }}\bigr )m \end{aligned}$$

Since

$$\begin{aligned}&\nabla _4{}^{(N)}{\hat{\pi }}_{34}=q^{-1}\hat{{L}}j\qquad \nabla _3{}^{(N)}{\hat{\pi }}_{34}=q\hat{{\underline{L}}}j\\&\quad g^{AC}\nabla _C{}^{(N)}{\hat{\pi }}_{A4}={{\,\mathrm{div}\,}}\!\!\!\!\!/\,m-q^{-1}{{\,\mathrm{tr}\,}}\chi j-\frac{1}{2} q{{\,\mathrm{tr}\,}}{\underline{\chi }}n\\&\quad g^{AC}\nabla _C{}^{(N)}{\hat{\pi }}_{A3}={{\,\mathrm{div}\,}}\!\!\!\!\!/\,{\underline{m}}-q{{\,\mathrm{tr}\,}}{\underline{\chi }}j-\frac{1}{2} q^{-1}{{\,\mathrm{tr}\,}}\chi {\underline{n}}\end{aligned}$$

we obtain

5.53 $$\begin{aligned}&8{\tilde{\Lambda }}^3+8\tilde{{\underline{\Lambda }}}^3=\Bigl [3\Omega ^{-1}q^{-1}Dj+3\Omega ^{-1}q{\underline{D}}j-3{{\,\mathrm{div}\,}}\!\!\!\!\!/\,(m+{\underline{m}})\nonumber \\&\quad +3\bigl (q{{\,\mathrm{tr}\,}}{\underline{\chi }}+q^{-1}{{\,\mathrm{tr}\,}}\chi \bigr )j+\frac{3}{2}q{{\,\mathrm{tr}\,}}{\underline{\chi }}\,n+\frac{3}{2}q^{-1}{{\,\mathrm{tr}\,}}\chi {\underline{n}}\Bigr ]\rho +2(p_3+p_4)\rho \nonumber \\&\quad +\Bigl (\Omega ^{-1}q^{-1}Dm+q^{-1}\chi ^\sharp \cdot m-\bigl (q^{-1}{\hat{\omega }}+\hat{{L}}q^{-1}\bigr )m-\nabla \!\!\!\!/_A n-\bigl (\eta +\mathrm {d}\!\!\!/\log q\bigr )n,{\underline{\beta }}_q\Bigr )\nonumber \\&\quad -\Bigl (\Omega ^{-1}q{\underline{D}}{\underline{m}}+q{\underline{\chi }}^\sharp \cdot {\underline{m}}-\bigl (q{\hat{{\underline{\omega }}}}+\hat{{\underline{L}}}q\bigr ){\underline{m}}-\nabla \!\!\!\!/_A{\underline{n}}+\bigl (\eta +\mathrm {d}\!\!\!/\log q\bigr ){\underline{n}},\beta _q\Bigr )\nonumber \\&\quad +\frac{1}{2}\bigl (2q\Omega ^{-1}{\hat{{\underline{D}}}}{\hat{i}}-{{\,\mathrm{tr}\,}}{\underline{\chi }}{\hat{i}}-\nabla \!\!\!\!/{\hat{\otimes }}{\underline{m}}+2q{\hat{{\underline{\chi }}}}j+q^{-1}{\hat{\chi }}{\underline{n}}-\bigl (2{\underline{\eta }}-\zeta -\mathrm {d}\!\!\!/\log q\bigr ){\hat{\otimes }}{\underline{m}},\alpha _q\bigr )\nonumber \\&\quad +\frac{1}{2}\bigl (2q^{-1}\Omega ^{-1}{\hat{D}}{\hat{i}}-q^{-1}{{\,\mathrm{tr}\,}}\chi {\hat{i}}-\nabla \!\!\!\!/{\hat{\otimes }}m+2q^{-1}{\hat{\chi }}j+q{\hat{{\underline{\chi }}}}n\nonumber \\&\quad -\,\bigl (2{\underline{\eta }}+\zeta +\mathrm {d}\!\!\!/\log q\bigr ){\hat{\otimes }}m,{\underline{\alpha }}_q\bigr )\nonumber \\&\quad +3\Bigl ({{\,\mathrm{curl}\,}}\!\!\!\!\!/\,m+ q^{-1}{\hat{\chi }}\wedge {\hat{i}}+\bigl (\zeta +\mathrm {d}\!\!\!/\log q\bigr )\wedge m-{{\,\mathrm{curl}\,}}\!\!\!\!\!/\,{\underline{m}}\nonumber \\&\quad -\, q {\hat{{\underline{\chi }}}}\wedge {\hat{i}}+\bigl (\zeta +\mathrm {d}\!\!\!/\log q\bigr )\wedge {\underline{m}}\Bigr )\sigma \end{aligned}$$

Note now that by adding (5.51a) and (5.53) the term $2(p_3+p_4)\rho$ cancels. Therefore — let us only write the $\rho$ and $\sigma$ terms in the following formula — we have:

$$\begin{aligned}&8\bigl ({\tilde{\Lambda }}^2+{\tilde{\Lambda }}^3\bigr )+8\bigl (\tilde{{\underline{\Lambda }}}^2+\tilde{{\underline{\Lambda }}}^3\bigr )\doteq 3\Bigl (j+\frac{n+{\underline{n}}}{4}\Bigr )\bigl (q{{\,\mathrm{tr}\,}}{\underline{\chi }}+q^{-1}{{\,\mathrm{tr}\,}}\chi \Bigr )\rho \\&\quad +\,\Bigl [3\Omega ^{-1}q^{-1}D j+3\Omega ^{-1}q{\underline{D}}j+3\frac{n-{\underline{n}}}{4}\bigl (q{{\,\mathrm{tr}\,}}{\underline{\chi }}-q^{-1}{{\,\mathrm{tr}\,}}\chi \bigr )\Bigr ]\rho \end{aligned}$$

For the following recall also that

$$\begin{aligned} {\underline{m}}+m=0\,. \end{aligned}$$

In conclusion:

5.54a $$\begin{aligned} \boxed {3\cdot 8{\tilde{\rho }}\Bigl (\tilde{{\underline{\Lambda }}}^2+\tilde{{\underline{\Lambda }}}^3+{\tilde{\Lambda }}^2+{\tilde{\Lambda }}^3\Bigr )=X_\Lambda ^{2+3}+Y_\Lambda ^{2+3}+Q_\Lambda ^{2}+Q_{\Lambda }^{2,4}+Q_\Lambda ^{3}+Q_{\Lambda }^{3,4}}\nonumber \\ \end{aligned}$$

where

5.54b $$\begin{aligned} X_{\Lambda }^{2+3}:= & {} 9\Bigl (j+\frac{n+{\underline{n}}}{4}\Bigr )\bigl (q{{\,\mathrm{tr}\,}}{\underline{\chi }}+q^{-1}{{\,\mathrm{tr}\,}}\chi \Bigr )\rho {\tilde{\rho }}\end{aligned}$$

5.54c $$\begin{aligned} Y_\Lambda ^{2+3}:= & {} 3{\tilde{\rho }}\bigl (q\Omega ^{-1}{\hat{{\underline{D}}}}{\hat{i}},\alpha _q\bigr )-\frac{3}{2}{\tilde{\rho }}q{{\,\mathrm{tr}\,}}{\underline{\chi }}\bigl ({\hat{i}},\alpha _q\bigr )+3{\tilde{\rho }}\bigl (q^{-1}\Omega ^{-1}{\hat{D}}{\hat{i}},{\underline{\alpha }}_q\bigr )\nonumber \\&-\frac{3}{2}{\tilde{\rho }}q^{-1}{{\,\mathrm{tr}\,}}\chi \bigl ({\hat{i}},{\underline{\alpha }}_q\bigr ) \end{aligned}$$

and

$$\begin{aligned} Q_\Lambda ^2= & {} 3{\tilde{\rho }}\Bigl (2\bigl ({\underline{\eta }}+\eta \bigr )^\sharp \cdot {\hat{i}}+2\bigl (\eta +{\underline{\eta }}\bigr )j,\beta _q-{\underline{\beta }}_q\Bigr )\\&\quad +3{\tilde{\rho }}\Bigl (q^{-1}{\hat{\omega }}+\hat{{L}}q^{-1}+q^{-1}{{\,\mathrm{tr}\,}}\chi -q{\hat{{\underline{\omega }}}}-\hat{{\underline{L}}}q-q{{\,\mathrm{tr}\,}}{\underline{\chi }}\Bigr )\bigl (m,\beta _q-{\underline{\beta }}_q\bigr )\\ Q_{\Lambda }^{2,4}= & {} -\frac{3{\tilde{\rho }}}{\Omega }\Bigl (q^{-1}D{\underline{m}}+q{\underline{D}}m-2\Omega {{\,\mathrm{div}\,}}\!\!\!\!\!/\,{\hat{i}}-\Omega \mathrm {d}\!\!\!/j,\beta _q-{\underline{\beta }}_q\Bigr )\\ Q_{\Lambda }^{3}= & {} 9{\tilde{\rho }}\frac{n-{\underline{n}}}{4}\bigl (q{{\,\mathrm{tr}\,}}{\underline{\chi }}-q^{-1}{{\,\mathrm{tr}\,}}\chi \bigr )\rho \\&\quad +\,\frac{3{\tilde{\rho }}}{2}\Bigl (q^{-1}{{\,\mathrm{tr}\,}}\chi -2q^{-1}{\hat{\omega }}-2\hat{{L}}q^{-1}\Bigr )\bigl (m,{\underline{\beta }}_q\bigr ) \\&\quad -\,\frac{3{\tilde{\rho }}}{2}\Bigl (q{{\,\mathrm{tr}\,}}{\underline{\chi }}-2q{\hat{{\underline{\omega }}}}-2\hat{{\underline{L}}}q\Bigr )\bigl ({\underline{m}},\beta _q\bigr )\\&\quad +\,\frac{9{\tilde{\rho }}}{2\Omega }\Bigl [D\log q\bigl (\Omega {{\,\mathrm{tr}\,}}{\underline{\chi }}-2{\underline{\omega }}\bigr )-q^{-2}D\log q\bigl (\Omega {{\,\mathrm{tr}\,}}\chi -2\omega \bigr )\\&\quad -\,D\log q{\underline{D}}\log q-q^{-2}D\log q D\log q\Bigr ]\rho \\&\quad +\,\frac{9{\tilde{\rho }}}{2\Omega }\Bigl [q^2{\underline{D}}\log q\bigl (\Omega {{\,\mathrm{tr}\,}}{\underline{\chi }}-2{\underline{\omega }}\bigr )- {\underline{D}}\log q\bigl (\Omega {{\,\mathrm{tr}\,}}\chi -2\omega \bigr )\\&\quad -\,q^2 {\underline{D}}\log q{\underline{D}}\log q-{\underline{D}}\log qD\log q\Bigr ]\rho \\&\quad +\,\frac{3{\tilde{\rho }}}{\Omega }\Bigl (q^{-1}\Omega {\hat{\chi }}^\sharp \cdot m-2 q^{-1}\Omega \nabla \!\!\!\!/\log q D\log q-\Omega \bigl (\eta +\mathrm {d}\!\!\!/\log q\bigr )n,{\underline{\beta }}_q\Bigr )\\&\quad -\,\frac{3{\tilde{\rho }}}{\Omega }\Bigl (q\Omega {\hat{{\underline{\chi }}}}^\sharp \cdot {\underline{m}}-2 q\Omega \nabla \!\!\!\!/\log q {\underline{D}}\log q+\Omega \bigl (\eta +\mathrm {d}\!\!\!/\log q\bigr ){\underline{n}},\beta _q\Bigr )\\&\quad +\,\frac{3}{2}{\tilde{\rho }}\bigl (2q{\hat{{\underline{\chi }}}}j+q^{-1}{\hat{\chi }}{\underline{n}}-\bigl (2{\underline{\eta }}-\zeta -\mathrm {d}\!\!\!/\log q\bigr ){\hat{\otimes }}{\underline{m}},\alpha _q\bigr )\\&\quad +\,\frac{3}{2}{\tilde{\rho }}\bigl (2q^{-1}{\hat{\chi }}j+q{\hat{{\underline{\chi }}}}n-\bigl (2{\underline{\eta }}+\zeta +\mathrm {d}\!\!\!/\log q\bigr ){\hat{\otimes }}m,{\underline{\alpha }}_q\bigr )\\&\quad +\,9{\tilde{\rho }}\Bigl ( q^{-1}{\hat{\chi }}\wedge {\hat{i}}+\bigl (\zeta +\mathrm {d}\!\!\!/\log q\bigr )\wedge m- q {\hat{{\underline{\chi }}}}\wedge {\hat{i}}+\bigl (\zeta +\mathrm {d}\!\!\!/\log q\bigr )\wedge {\underline{m}}\Bigr )\sigma \\ Q_{\Lambda }^{3,4}= & {} \frac{9{\tilde{\rho }}}{2\Omega } \Bigl [ D\bigl (\Omega {{\,\mathrm{tr}\,}}{\underline{\chi }}-2{\underline{\omega }}\bigr )+q^{-2}D\bigl (\Omega {{\,\mathrm{tr}\,}}\chi -2\omega \bigr )- D{\underline{D}}\log q+q^{-2} DD\log q\\&\quad +\,q^2{\underline{D}}\bigl (\Omega {{\,\mathrm{tr}\,}}{\underline{\chi }}-2{\underline{\omega }}\bigr )+{\underline{D}}\bigl (\Omega {{\,\mathrm{tr}\,}}\chi -2\omega \bigr )-q^2{\underline{D}}{\underline{D}}\log q+{\underline{D}}D\log q\Bigr ]\rho \\&\quad +\,\frac{3{\tilde{\rho }}}{\Omega }\Bigl (q^{-1}Dm+2 q^{-1}\Omega \nabla \!\!\!\!/D\log q,{\underline{\beta }}_q\Bigr )-\frac{3{\tilde{\rho }}}{\Omega }\Bigl (q{\underline{D}}{\underline{m}}-2 q \Omega \nabla \!\!\!\!/{\underline{D}}\log q,\beta _q\Bigr )\\&\quad +\,9{\tilde{\rho }}\Bigl ({{\,\mathrm{curl}\,}}\!\!\!\!\!/\,m-{{\,\mathrm{curl}\,}}\!\!\!\!\!/\,{\underline{m}}\Bigr )\sigma -\frac{3}{2}{\tilde{\rho }}\bigl (\nabla \!\!\!\!/{\hat{\otimes }}{\underline{m}},\alpha _q\bigr )-\frac{3}{2}{\tilde{\rho }}\bigl (\nabla \!\!\!\!/{\hat{\otimes }}m,{\underline{\alpha }}_q\bigr ) \end{aligned}$$

Here we used that

$$\begin{aligned}&j=\frac{1}{2}q\bigl (\Omega {{\,\mathrm{tr}\,}}{\underline{\chi }}-2{\underline{\omega }}\bigr )+\frac{1}{2}q^{-1}\bigl (\Omega {{\,\mathrm{tr}\,}}\chi -2\omega \bigr )-\frac{1}{2}q{\underline{D}}\log q+\frac{1}{2}q^{-1}D\log q\\&q^{-1}Dj=\frac{1}{2}D\log q\bigl (\Omega {{\,\mathrm{tr}\,}}{\underline{\chi }}-2{\underline{\omega }}\bigr )-\frac{1}{2}q^{-2}D\log q\bigl (\Omega {{\,\mathrm{tr}\,}}\chi -2\omega \bigr )\\&\quad -\,\frac{1}{2}D\log q{\underline{D}}\log q-\frac{1}{2}q^{-2}D\log q D\log q \\&\quad +\frac{1}{2} D\bigl (\Omega {{\,\mathrm{tr}\,}}{\underline{\chi }}-2{\underline{\omega }}\bigr )+\frac{1}{2}q^{-2}D\bigl (\Omega {{\,\mathrm{tr}\,}}\chi -2\omega \bigr )-\frac{1}{2} D{\underline{D}}\log q+\frac{1}{2}q^{-2} DD\log q \end{aligned}$$

and similarly for $q{\underline{D}}j$. Also,

$$\begin{aligned} n= & {} -2 q^{-1}D\log q\\&\nabla \!\!\!\!/n=2 q^{-1}\nabla \!\!\!\!/\log q D\log q-2 q^{-1}\nabla \!\!\!\!/D\log q \end{aligned}$$

and similarly for $\nabla \!\!\!\!/{\underline{n}}$.

Analogously we conclude:

5.55a $$\begin{aligned} \boxed {3\cdot 8{\tilde{\sigma }}\Bigl ({\tilde{K}}^2+{\tilde{K}}^3-\tilde{{\underline{K}}}^2-\tilde{{\underline{K}}}^3\Bigr )=X_K^{2+3}+Y_K^{2+3}+Q_K^{2}+Q_K^{2,4}+Q_K^{3}+Q_K^{3,4}}\nonumber \\ \end{aligned}$$

where

5.55b $$\begin{aligned}&X_K^{2+3}:=9\Bigl (j+\frac{n+{\underline{n}}}{4}\Bigr )\bigl (q{{\,\mathrm{tr}\,}}{\underline{\chi }}+q^{-1}{{\,\mathrm{tr}\,}}\chi \Bigr )\sigma {\tilde{\sigma }}\end{aligned}$$

5.55c $$\begin{aligned}&Y_K^{2+3} := -3{\tilde{\sigma }}q\Omega ^{-1}{\hat{{\underline{D}}}}{\hat{i}}\wedge \alpha +\frac{3}{2}{\tilde{\sigma }}q{{\,\mathrm{tr}\,}}{\underline{\chi }}{\hat{i}}\wedge \alpha +3\sigma q^{-1}\Omega ^{-1}{\hat{D}}{\hat{i}}\wedge {\underline{\alpha }}\nonumber \\&\quad -\,\frac{3}{2}{\tilde{\sigma }}q^{-1}{{\,\mathrm{tr}\,}}\chi {\hat{i}}\wedge {\underline{\alpha }}\end{aligned}$$

The terms $Q_K^{2}$, $Q_K^{2,4}$, $Q_K^{3}$, $Q_K^{3,4}$, are of a similar structure as $Q_\Lambda ^{2}$, $Q_\Lambda ^{2,4}$, $Q_\Lambda ^{3}$, $Q_\Lambda ^{3,4}$, respectively.

With regard of the ${\underline{\Theta }}$, and $\Theta$ components, we compute:

$$\begin{aligned} -p_3+3{\underline{\Lambda }}(d)= & {} \nabla _4{}^{(N)}{\hat{\pi }}_{33}-\frac{1}{2}\nabla _3{}^{(N)}{\hat{\pi }}_{34}-\frac{1}{2}\nabla ^A{}^{(N)}{\hat{\pi }}_{A3}\\ -p_4+3\Lambda (d)= & {} \nabla _3{}^{(N)}{\hat{\pi }}_{44}-\frac{1}{2}\nabla _4{}^{(N)}{\hat{\pi }}_{34}-\frac{1}{2}\nabla ^A{}^{(N)}{\hat{\pi }}_{A4}\\ \nabla _4{}^{(N)}{\hat{\pi }}_{33}= & {} q^{-1}\Omega ^{-1} D {\underline{n}}-4{\underline{\eta }}^\sharp \cdot {\underline{m}}+2\bigl (q^{-1}{\hat{\omega }}+\hat{{L}}q^{-1}\bigr ){\underline{n}}\\ \nabla _3{}^{(N)}{\hat{\pi }}_{44}= & {} q\Omega ^{-1}{\underline{D}}n-4\eta ^\sharp \cdot m+2\bigl (q{\hat{{\underline{\omega }}}}+\hat{{\underline{L}}}q\bigr )n \end{aligned}$$

and

$$\begin{aligned} p\!\!\!/\,-6I(d)= & {} -p\!\!\!/\,-3q^{-1}\Omega ^{-1}D{\underline{m}}+3\bigl (q^{-1}{\hat{\omega }}+\hat{{L}}q^{-1}\bigr ){\underline{m}}+3\mathrm {d}\!\!\!/j \\&\quad +\,6{\underline{\eta }}j+6{\underline{\eta }}^\sharp \cdot {\hat{i}}-3q{\underline{\chi }}^\sharp \cdot m\\&\quad =\frac{1}{2}\bigl (-5q^{-1}\Omega ^{-1}D{\underline{m}}+q\Omega ^{-1}{\underline{D}}m\bigr )-{{\,\mathrm{div}\,}}\!\!\!\!\!/\,{\hat{i}}+\frac{5}{2}\mathrm {d}\!\!\!/j \\&\quad -\bigl (-5{\underline{\eta }}+\eta \bigr )^\sharp \cdot {\hat{i}}-\bigl (\eta -5{\underline{\eta }}\bigr )j\\&\quad +\,\frac{1}{2}\bigl (7q^{-1}{\hat{\omega }}+7\hat{{L}}q^{-1}+q^{-1}{{\,\mathrm{tr}\,}}\chi \bigr ){\underline{m}}\\&\quad +\,\frac{1}{2}\bigl (q{\hat{{\underline{\omega }}}}+\hat{{\underline{L}}}q-2q{{\,\mathrm{tr}\,}}{\underline{\chi }}\bigr )m-3{\hat{{\underline{\chi }}}}^\sharp \cdot m\\ -p\!\!\!/\,+6{\underline{I}}(d)= & {} p\!\!\!/\,+3q\Omega ^{-1}{\underline{D}}m-3\bigl (q{\hat{{\underline{\omega }}}}+\hat{{\underline{L}}}q\bigr )m-3\mathrm {d}\!\!\!/j-6\eta j \\&\quad -\,6\eta ^\sharp \cdot {\hat{i}}+3q^{-1}\chi ^\sharp \cdot {\underline{m}}\\&\quad = -\frac{1}{2}\bigl (q^{-1}\Omega ^{-1}D{\underline{m}}-5q\Omega ^{-1}{\underline{D}}m\bigr )+{{\,\mathrm{div}\,}}\!\!\!\!\!/\,{\hat{i}} \\&\quad -\,\frac{5}{2}\mathrm {d}\!\!\!/j +\bigl ({\underline{\eta }}-5\eta \bigr )^\sharp \cdot {\hat{i}}+\bigl (-5\eta +{\underline{\eta }}\bigr )j\\&\quad -\,\frac{1}{2}\bigl (q^{-1}{\hat{\omega }}+\hat{{L}}q^{-1}-4q^{-1}{{\,\mathrm{tr}\,}}\chi \bigr ){\underline{m}}\\&\quad -\,\frac{1}{2}\bigl (7q{\hat{{\underline{\omega }}}}+7\hat{{\underline{L}}}q+q{{\,\mathrm{tr}\,}}{\underline{\chi }}\bigr )m+3{\hat{\chi }}^\sharp \cdot {\underline{m}}\end{aligned}$$

Also,

$$\begin{aligned} \nabla _4{}^{(N)}{\hat{\pi }}_{34}= & {} q^{-1}\hat{{L}}j\qquad \nabla _3{}^{(N)}{\hat{\pi }}_{34}=q\hat{{\underline{L}}}j\\ g^{AC}\nabla _C{}^{(N)}{\hat{\pi }}_{A4}= & {} {{\,\mathrm{div}\,}}\!\!\!\!\!/\,m-q^{-1}{{\,\mathrm{tr}\,}}\chi j-\frac{1}{2} q{{\,\mathrm{tr}\,}}{\underline{\chi }}n\\ g^{AC}\nabla _C{}^{(N)}{\hat{\pi }}_{A3}= & {} {{\,\mathrm{div}\,}}\!\!\!\!\!/\,{\underline{m}}-q{{\,\mathrm{tr}\,}}{\underline{\chi }}j-\frac{1}{2} q^{-1}{{\,\mathrm{tr}\,}}\chi {\underline{n}}\end{aligned}$$

Therefore

5.56a $$\begin{aligned} \boxed { 4({\tilde{\alpha }}_q,{\tilde{\Theta }}^2+{\tilde{\Theta }}^3) = X_\Theta ^{2+3} +Y_\Theta ^{2+3}+ Q_\Theta ^{2+3} } \end{aligned}$$

where

5.56b $$\begin{aligned} X_\Theta ^{2+3}:=\frac{1}{2}q{{\,\mathrm{tr}\,}}{\underline{\chi }}j ({\tilde{\alpha }}_q,\alpha _q)+\frac{1}{4}\Bigl (q^{-1}{{\,\mathrm{tr}\,}}\chi +8 q^{-1}{\hat{\omega }}+8\hat{{L}}q^{-1}\Bigr ){\underline{n}}({\tilde{\alpha }}_q,\alpha _q)\qquad \quad \end{aligned}$$

5.56c $$\begin{aligned}&Y_\Theta ^{2+3} := -\frac{3}{2}q^{-1}{{\,\mathrm{tr}\,}}\chi \Bigl (\rho {\tilde{\alpha }}_q-\sigma {}^*{\tilde{\alpha }},{\hat{i}}\Bigr )+3\Bigl (\rho {\tilde{\alpha }}_q-\sigma {}^*{\tilde{\alpha }}_q,q^{-1}\Omega ^{-1}{\hat{D}}{\hat{i}}\Bigr )\nonumber \\&\quad +\frac{1}{2}\bigl (7q^{-1}{\hat{\omega }}+7\hat{{L}}q^{-1}+q^{-1}{{\,\mathrm{tr}\,}}\chi \bigr )({\tilde{\alpha }},{\underline{m}}{\hat{\otimes }}\beta )+\frac{1}{2}\bigl (q{\hat{{\underline{\omega }}}}+\hat{{\underline{L}}}q-2q{{\,\mathrm{tr}\,}}{\underline{\chi }}\bigr ) ({\tilde{\alpha }},m{\hat{\otimes }}\beta )\nonumber \\ \end{aligned}$$

and

5.56d $$\begin{aligned}&Q_\Theta ^{2+3} := \Bigl [q^{-1}\Omega ^{-1} D {\underline{n}}-4{\underline{\eta }}^\sharp \cdot {\underline{m}}-\frac{1}{2}q\Omega ^{-1}{\underline{D}}j-\frac{1}{2}{{\,\mathrm{div}\,}}\!\!\!\!\!/\,{\underline{m}}\Bigr ]({\tilde{\alpha }}_q,\alpha _q)\nonumber \\&\quad -\frac{3}{2}\Bigl [{{\,\mathrm{curl}\,}}\!\!\!\!\!/\,{\underline{m}}+ q {\hat{{\underline{\chi }}}}\wedge {\hat{i}}-\bigl (\zeta +\mathrm {d}\!\!\!/\log q\bigr )\wedge {\underline{m}}\Bigr ]({\tilde{\alpha }}_q,{}^*\alpha _q) +\Bigl ({\tilde{\alpha }}_q,\bigl (p\!\!\!/\,-6I(d)\bigr )'{\hat{\otimes }}\beta _q\Bigr )\nonumber \\&\quad +\frac{3}{2}\Bigl (\rho {\tilde{\alpha }}_q-\sigma {}^*{\tilde{\alpha }}_q,-\nabla \!\!\!\!/{\hat{\otimes }}m-\bigl (2{\underline{\eta }}+\zeta +\mathrm {d}\!\!\!/\log q\bigr ){\hat{\otimes }}m +2q^{-1}{\hat{\chi }}j+q{\hat{{\underline{\chi }}}}n\Bigr )\nonumber \\ \end{aligned}$$

(Here $(p\!\!\!/\,-6I)'$ denotes the above expression for $p\!\!\!/\,-6I$ without the border line terms included in $Y_\Theta ^{2+3}$ above.)

Similarly,

5.57a $$\begin{aligned}&\boxed { 4({\tilde{{\underline{\alpha }}}}_q,\tilde{{\underline{\Theta }}}^2+\tilde{{\underline{\Theta }}}^3) = X_{{\underline{\Theta }}}^{2+3} +Y_{{\underline{\Theta }}}^{2+3} + Q_{{\underline{\Theta }}}^{2+3} } \end{aligned}$$

5.57b $$\begin{aligned}&X_{{\underline{\Theta }}}^{2+3} := \frac{1}{2} q^{-1}{{\,\mathrm{tr}\,}}\chi j({\tilde{{\underline{\alpha }}}}_q,{\underline{\alpha }}_q)+\frac{1}{4}\Bigl ( q{{\,\mathrm{tr}\,}}{\underline{\chi }}+8q{\hat{{\underline{\omega }}}}+8\hat{{\underline{L}}}q\Bigr ) n({\tilde{{\underline{\alpha }}}}_q,{\underline{\alpha }}_q)\qquad \quad \end{aligned}$$

5.57c $$\begin{aligned}&Y_{{\underline{\Theta }}}^{2+3} := -\frac{3}{2}{{\,\mathrm{tr}\,}}{\underline{\chi }}\Bigl (\rho {\tilde{{\underline{\alpha }}}}+\sigma {}^*{\tilde{{\underline{\alpha }}}},{\hat{i}}\Bigr )+3\Bigl (\rho {\tilde{{\underline{\alpha }}}}+\sigma {}^*{\tilde{{\underline{\alpha }}}},q\Omega ^{-1}{\hat{{\underline{D}}}}{\hat{i}}\Bigr )\nonumber \\&\quad -\,\frac{1}{2}\bigl (q^{-1}{\hat{\omega }}+\hat{{L}}q^{-1}-4q^{-1}{{\,\mathrm{tr}\,}}\chi \bigr )\Bigl ({\tilde{{\underline{\alpha }}}},{\underline{m}}{\hat{\otimes }}{\underline{\beta }}\Bigr )\nonumber \\&\quad -\frac{1}{2}\bigl (7q{\hat{{\underline{\omega }}}}+7\hat{{\underline{L}}}q+q{{\,\mathrm{tr}\,}}{\underline{\chi }}\bigr )\Bigl ({\tilde{{\underline{\alpha }}}},m{\hat{\otimes }}{\underline{\beta }}\Bigr ) \end{aligned}$$

5.57d $$\begin{aligned}&Q_{{\underline{\Theta }}}^{2+3} := \Bigl [q\Omega ^{-1}{\underline{D}}n-4\eta ^\sharp \cdot m-\frac{1}{2}q^{-1}\hat{{L}}j -\frac{1}{2} {{\,\mathrm{div}\,}}\!\!\!\!\!/\,m \Bigr ]({\tilde{{\underline{\alpha }}}}_q,{\underline{\alpha }}_q)\nonumber \\&\quad -\frac{3}{2}\Bigl [{{\,\mathrm{curl}\,}}\!\!\!\!\!/\,m+ q^{-1}{\hat{\chi }}\wedge {\hat{i}}+\bigl (\zeta +\mathrm {d}\!\!\!/\log q\bigr )\wedge m\Bigr ]({\tilde{{\underline{\alpha }}}}_q,{}^*{\underline{\alpha }}_q)+\Bigl ({\tilde{{\underline{\alpha }}}},\bigl (6{\underline{I}}(d)-p\!\!\!/\,\bigr )'{\hat{\otimes }}{\underline{\beta }}\Bigr )\nonumber \\&\quad +\frac{3}{2}\Bigl (\rho {\tilde{{\underline{\alpha }}}}+\sigma {}^*{\tilde{{\underline{\alpha }}}},-\nabla \!\!\!\!/{\hat{\otimes }}{\underline{m}}+2q{\hat{{\underline{\chi }}}}j+q^{-1}{\hat{\chi }}{\underline{n}}-\bigl (2{\underline{\eta }}-\zeta -\mathrm {d}\!\!\!/\log q\bigr ){\hat{\otimes }}{\underline{m}}\Bigr ) \end{aligned}$$

$$\square $$

#### Proof of Prop. 5.11

The statement of Proposition 5.11 follows from Lemma 5.12 and Lemma 5.15. Indeed by adding (5.27) and (5.49) we obtain

5.58 $$\begin{aligned}&\phi \bigl ({{\,\mathrm{div}\,}}Q(\tilde{{\mathcal {L}}}_{N}W)\bigr )(M_q,M_q,M_q) \ge \frac{\phi }{(2\Omega )^3}\bigl [X+Y\bigr ]\nonumber \\&\quad -\frac{C}{r}\frac{1}{\Omega }Q[\tilde{{\mathcal {L}}}_{N}W](n,M_q,M_q,M_q) -\frac{C}{r}\frac{1}{\Omega }Q[W](n,M_q,M_q,M_q)\nonumber \\&\quad -\frac{C}{r}\frac{1}{\Omega }\Bigl [\Omega ^2{P\!\!\!\!/\,}^q\Bigr ] \end{aligned}$$

and it remains to control

5.59 $$\begin{aligned} X:=X^1+X^{2+3}\,,\qquad Y:=Y^1+Y^{2+3}\,, \end{aligned}$$

where $X^1$, and $Y^1$ are defined in Lemma 5.12, see (5.28), and where $X^{2+3}$, $Y^{2+3}$ are defined in Lemma 5.15, see (5.50). *We will show that the terms in X essentially cancel, while the terms in Y are controlled by our assumptions.*

Consider first

5.60a $$\begin{aligned} X_\Lambda :=X_\Lambda ^1+X_{\Lambda }^{2+3}\qquad X_K:=X_K^1+X_K^{2+3} \end{aligned}$$

We have by (5.31e) and (5.54b) as well as (5.41b) and (5.55b) that

5.60b $$\begin{aligned} \begin{aligned} X_\Lambda&=\frac{9}{2}\Bigl (j+\frac{n+{\underline{n}}}{4}\Bigr )\Bigl [q{{\,\mathrm{tr}\,}}{\underline{\chi }}+q^{-1}{{\,\mathrm{tr}\,}}\chi -2q{\hat{{\underline{\omega }}}}-2q^{-1}{\hat{\omega }}-\hat{{\underline{L}}}q-\hat{{L}}q^{-1}\bigr ]\rho {\tilde{\rho }}\\&=\frac{9}{\Omega }\Bigl (j+\frac{n+{\underline{n}}}{4}\Bigr )j\rho {\tilde{\rho }}\\ X_K&=\frac{9}{\Omega }\Bigl (j+\frac{n+{\underline{n}}}{4}\Bigr )j{\tilde{\sigma }}\sigma \end{aligned} \end{aligned}$$

Thus

5.60c $$\begin{aligned} \phi |X_\Lambda |\le \frac{9}{\Omega }\frac{3C}{2}\phi \bigl (q{{\,\mathrm{tr}\,}}{\underline{\chi }}+q^{-1}{{\,\mathrm{tr}\,}}\chi \bigr )|j|{\tilde{\rho }}\rho \le \frac{27}{\Omega }\frac{(2C)^3}{r}{\tilde{\rho }}\rho \end{aligned}$$

Moreover for

5.61a $$\begin{aligned} X_\Theta :=X_\Theta ^1+X_\Theta ^{2+3}\qquad X_{{\underline{\Theta }}}=X_{{\underline{\Theta }}}^1+X_{{\underline{\Theta }}}^{2+3} \end{aligned}$$

we have by (5.44b) and (5.57b) as well as (5.45b) and (5.56b) that

5.61b $$\begin{aligned} \begin{aligned} X_\Theta&=\frac{{\underline{n}}}{8}\bigl (2q{\hat{{\underline{\omega }}}}+2q^{-1}{\hat{\omega }}-q{{\,\mathrm{tr}\,}}{\underline{\chi }}-q^{-1}{{\,\mathrm{tr}\,}}\chi +\hat{{\underline{L}}}q+9\hat{{L}}q^{-1}\bigr )({\tilde{\alpha }}_q,\alpha _q)\\&=-\frac{{\underline{n}}j}{4\Omega }({\tilde{\alpha }}_q,\alpha _q)+{\underline{n}}\hat{{L}}q^{-1}({\tilde{\alpha }}_q,\alpha _q)\\ X_{{\underline{\Theta }}}&= \frac{n}{8} \bigl (2q^{-1}{\hat{\omega }}+2q{\hat{{\underline{\omega }}}}-q{{\,\mathrm{tr}\,}}{\underline{\chi }}-q^{-1}{{\,\mathrm{tr}\,}}\chi +\hat{{L}}q^{-1}+9\hat{{\underline{L}}}q\bigr )({\tilde{{\underline{\alpha }}}}_q,{\underline{\alpha }}_q)\\&= -\frac{n j}{4\Omega } ({\tilde{{\underline{\alpha }}}}_q,{\underline{\alpha }}_q) +n \hat{{\underline{L}}}q ({\tilde{{\underline{\alpha }}}}_q,{\underline{\alpha }}_q) \end{aligned} \end{aligned}$$

and thus

5.61c $$\begin{aligned} \phi |X_{\Theta } |\le \frac{1}{\Omega }\frac{C}{r}|{\tilde{\alpha }}_q ||\alpha _q |\qquad \phi |X_{{\underline{\Theta }}} |\le \frac{1}{\Omega }\frac{C}{r}|{\tilde{{\underline{\alpha }}}}_q ||{\underline{\alpha }}_q |\end{aligned}$$

Similarly for $X_{{\underline{\Xi }}}$ and $X_{\Xi }$.

Let us now turn to the “borderline error terms” *Y*.^43^ We consider first

5.62a $$\begin{aligned} Y_\Lambda :=Y_\Lambda ^1+Y_\Lambda ^{2+3} \end{aligned}$$

which in view of (5.31f) and (5.54c) is given by

5.62b $$\begin{aligned} Y_\Lambda= & {} -3{\tilde{\rho }}q{{\,\mathrm{tr}\,}}{\underline{\chi }}\bigl ({\hat{i}},\alpha _q\bigr )- 3{\tilde{\rho }}q^{-1}{{\,\mathrm{tr}\,}}\chi \bigl ({\hat{i}},{\underline{\alpha }}_q\bigr ) -9 q^{-1}{{\,\mathrm{tr}\,}}\chi {\tilde{\rho }}\bigl (m, \beta _q\bigr )\nonumber \\&\quad +9q{{\,\mathrm{tr}\,}}{\underline{\chi }}{\tilde{\rho }}\bigl ({\underline{m}},{\underline{\beta }}_q\bigr ) +3{\tilde{\rho }}\bigl (q\Omega ^{-1}{\hat{{\underline{D}}}}{\hat{i}},\alpha _q\bigr )+3{\tilde{\rho }}\bigl (q^{-1}\Omega ^{-1}{\hat{D}}{\hat{i}},{\underline{\alpha }}_q\bigr )\qquad \quad \end{aligned}$$

By (${{\textbf {BA:I}}.iv}^\prime$), (${{\textbf {BA:I}}.vi}^\prime$) and (${\varvec{BA:II}}.i$) we estimate

$$\begin{aligned}&\phi |{\tilde{\rho }}q{{\,\mathrm{tr}\,}}{\underline{\chi }}\bigl ({\hat{i}},\alpha _q\bigr )| \le |{\tilde{\rho }}|\frac{4C_0}{r\Omega }\bigl (q{{\,\mathrm{tr}\,}}{\underline{\chi }}+q^{-1}{{\,\mathrm{tr}\,}}\chi \bigr )|\alpha _q| \le \frac{C_0}{\Omega }\frac{C}{r}\bigl [|{\tilde{\rho }}|^2+|\alpha _q|^2\bigr ]\\&\quad \phi |q{{\,\mathrm{tr}\,}}{\underline{\chi }}{\tilde{\rho }}\bigl ({\underline{m}},{\underline{\beta }}_q\bigr )|\le \frac{4}{r}{\tilde{\rho }}\bigl (\Omega |\eta |+\Omega |{\underline{\eta }}|+\Omega |\mathrm {d}\!\!\!/\log q|\bigr )|{\underline{\beta }}|\le \frac{C_0}{\Omega }\frac{C}{r}\bigl [{\tilde{\rho }}^2+|{\underline{\beta }}|^2\bigr ]\\&\quad \phi |{\tilde{\rho }}\bigl (q\Omega ^{-1}{\hat{{\underline{D}}}}{\hat{i}},\alpha _q\bigr )|\le \frac{\phi }{\Omega }|{\tilde{\rho }}| q|{\hat{{\underline{D}}}}{\hat{i}}||\alpha _q|\le \frac{|{\tilde{\rho }}|}{\Omega }\phi \Bigl ( q{{\,\mathrm{tr}\,}}{\underline{\chi }}q{{\,\mathrm{tr}\,}}{\underline{\chi }}\\&\quad +\,q{{\,\mathrm{tr}\,}}{\underline{\chi }}q^{-1}{{\,\mathrm{tr}\,}}\chi \bigr )|\alpha _q|\le \frac{C}{\Omega r}|{\tilde{\rho }}||\alpha _q| \end{aligned}$$

and thus

5.62c $$\begin{aligned} \frac{\phi }{(2\Omega )^3} |Y_\Lambda | \ge -\frac{C_0}{\Omega }\frac{C}{r}\bigl (Q[W](n,M_q,M_q,M_q)+Q[\tilde{{\mathcal {L}}}_{N}W](n,M_q,M_q,M_q)\bigr )\nonumber \\ \end{aligned}$$

Next consider

5.63a $$\begin{aligned} Y_\Theta :=Y_\Theta ^1+Y_\Theta ^{2+3}\qquad Y_{{\underline{\Theta }}}:=Y_{{\underline{\Theta }}}^1+Y_{{\underline{\Theta }}}^{2+3}\,. \end{aligned}$$

By (5.45d) and (5.56c) as well as (5.44d) and (5.57c) we have

5.63b $$\begin{aligned} Y_{\Theta }= & {} -3q^{-1}{{\,\mathrm{tr}\,}}\chi \bigl ({\tilde{\alpha }},{\hat{i}}\bigr )\rho +3q^{-1}{{\,\mathrm{tr}\,}}\chi \bigl ({\tilde{\alpha }},{}^*{\hat{i}}\bigr )\sigma \nonumber \\&\quad +\,\frac{3}{4}\bigl (q^{-1}{{\,\mathrm{tr}\,}}\chi +q{{\,\mathrm{tr}\,}}{\underline{\chi }}\bigr )\Bigl ({\tilde{\alpha }},{\underline{m}}{\hat{\otimes }}\beta _q\Bigr )\nonumber \\&\quad +3\Bigl (\rho {\tilde{\alpha }}_q-\sigma {}^*{\tilde{\alpha }}_q,q^{-1}\Omega ^{-1}{\hat{D}}{\hat{i}}\Bigr )+\frac{1}{4}\bigl (q{{\,\mathrm{tr}\,}}{\underline{\chi }}-2q{\hat{{\underline{\omega }}}}-2\hat{{\underline{L}}}q\bigr )\Bigl ({\tilde{\alpha }},{\underline{m}}{\hat{\otimes }}\beta _q\Bigr )\nonumber \\&\quad +\frac{7}{2}\bigl (q^{-1}{\hat{\omega }}+\hat{{L}}q^{-1}\bigr )({\tilde{\alpha }},{\underline{m}}{\hat{\otimes }}\beta ) \end{aligned}$$

5.63c $$\begin{aligned} Y_{{\underline{\Theta }}}= & {} -3q{{\,\mathrm{tr}\,}}{\underline{\chi }}\bigl ({\tilde{{\underline{\alpha }}}},{\hat{i}}\bigr )\rho +3 q{{\,\mathrm{tr}\,}}{\underline{\chi }}\bigl ({\tilde{{\underline{\alpha }}}},{}^*{\hat{i}}\bigr )\sigma -\frac{3}{4}\bigl (q{{\,\mathrm{tr}\,}}{\underline{\chi }}+q^{-1}{{\,\mathrm{tr}\,}}\chi \bigr )\Bigl ({\tilde{{\underline{\alpha }}}},m{\hat{\otimes }}{\underline{\beta }}_q\Bigr )\nonumber \\&\quad +3\Bigl (\rho {\tilde{{\underline{\alpha }}}}+\sigma {}^*{\tilde{{\underline{\alpha }}}},q\Omega ^{-1}{\hat{{\underline{D}}}}{\hat{i}}\Bigr )-\frac{1}{4}\bigl (2q^{-1}{\hat{\omega }}+2\hat{{L}}q^{-1}-q^{-1}{{\,\mathrm{tr}\,}}\chi \bigr )\Bigl ({\tilde{{\underline{\alpha }}}},{\underline{m}}{\hat{\otimes }}{\underline{\beta }}\Bigr )\nonumber \\&\quad -\frac{7}{2}\bigl (q{\hat{{\underline{\omega }}}}+\hat{{\underline{L}}}q\bigr )\Bigl ({\tilde{{\underline{\alpha }}}},m{\hat{\otimes }}{\underline{\beta }}\Bigr ) \end{aligned}$$

and thus

5.63d $$\begin{aligned}&\phi |Y_\Theta | \le \frac{C}{r} \frac{C_0}{\Omega }|{\tilde{\alpha }}|\bigl [|\rho |+|\sigma |+|\beta _q|\bigr ] \end{aligned}$$

5.63e $$\begin{aligned}&\phi |Y_{{\underline{\Theta }}} | \le \frac{C}{r}\frac{C_0}{\Omega }|{\tilde{{\underline{\alpha }}}}|\bigl [|\rho |+|\sigma |+|{\underline{\beta }}_q|\bigr ] \end{aligned}$$

Similarly for $Y_{{\underline{\Xi }}}$ and $Y_{\Xi }$. $\square$

### Integral inequality

We discuss briefly the implications of the integral inequality obtained with the help of Prop. 5.11. The remaining error terms — namely the terms involving angular derivatives in the second line of (5.47) — are the subject of Section 6, where it will be shown that after integration on $\Sigma _r$ it is controlled by the *same* error terms already introduced in (5.23), and thus does not alter the following discussion.

#### Gronwall argument

##### Lemma 5.16

Suppose $f:{\mathbb {R}}^+\rightarrow {\mathbb {R}}^+$ is a positive function that satisfies an integral inequality of the form

5.64 $$\begin{aligned} f(r_2)+\int _{r_1}^{r_2}\Bigl [\frac{\kappa (r)}{r}f(r) -\frac{C}{r}h(r)\Bigr ]\mathrm {d}r\le f(r_1) \qquad \text {: for all } r_2>r_1\ge r_0\qquad \end{aligned}$$

where $C>0$ is a constant, $h:{\mathbb {R}}^+\rightarrow {\mathbb {R}}$ an arbitrary function, and $\kappa :{\mathbb {R}}^+\rightarrow {\mathbb {R}}^+$ is a positive function with the property that for some positive constants $0<\kappa _0<\kappa _1<\infty$:

5.65 $$\begin{aligned} \kappa _0\le \kappa (r)\le \kappa _1 \end{aligned}$$

Assume moreover that for some $K_1<\infty$, and $H_1<\infty$:

5.66 $$\begin{aligned} K(r):=\int _{r_0}^r\frac{\kappa _1-\kappa (r)}{r}\mathrm {d}r \le K_1\,, \qquad H(r_1,r_0):=\int _{r_0}^{r_1}|h(r)|r^{\kappa _1-1}\mathrm {d}r \le H_1 \end{aligned}$$

Then for some constant *C*, depending on $\kappa _1/\kappa _0$, $K_1$, $H_1$ and $f(r_0)$, we have that for all $r\ge 2r_0$:

5.67 $$\begin{aligned} f(r)\le \frac{C}{r^{\kappa _1}}\,. \end{aligned}$$

##### Proof

With

5.68 $$\begin{aligned} F(r_2,r_1):= \int _{r_1}^{r_2}\Bigl [\frac{\kappa (r)}{r}f(r) -\frac{C}{r}h(r)\Bigr ]\mathrm {d}r \end{aligned}$$

we obtain from the integral inequality that

$$\begin{aligned}&\frac{\mathrm {d}}{\mathrm {d}r}\Bigl [F(r_2,r)r^{\kappa _1}\Bigr ]_{r=r_1}=\frac{\kappa (r_1)}{r_1}\Bigl [-f(r_1)+\frac{\kappa _1}{\kappa (r_1)}F(r_2,r_1)+\frac{C}{\kappa (r_1)}h(r_1)\Bigr ]r_1^{\kappa _1}\\&\quad \le -\kappa (r_1) f(r_2) r_1^{\kappa _1-1}+\frac{\kappa _1-\kappa (r_1)}{r_1}F(r_2,r_1)r_1^{\kappa _1}+C h(r_1)r_1^{\kappa _1-1} \end{aligned}$$

or

$$\begin{aligned} \frac{\mathrm {d}}{\mathrm {d}r}\Bigl [F(r_2,r)r^{\kappa _1}e^{-K(r)}\Bigr ]_{r=r_1} \le \Bigl [-\kappa _0 f(r_2) r_1^{\kappa _1-1}+C h(r_1)r_1^{\kappa _1-1}\Bigr ]e^{-K(r_1)} \end{aligned}$$

where *K*(*r*) is defined by (5.66) and we assume $K\le K_1$ for some constant $0<K_1<\infty$.

Then we may integrate in $r_1$ on $[r_0,r_2]$

$$\begin{aligned} -F(r_2,r_0)r_0^{\kappa _1} e^{-K(r_0)}\le -f(r_2)\frac{\kappa _0e^{-K_1}}{\kappa _1}\bigl (r_2^{\kappa _1}-r_0^{\kappa _1}\bigr )+C\int _{r_0}^{r_2}|h(r)|r^{\kappa _1-1}\mathrm {d}r\,. \end{aligned}$$

This implies the following upper bound on *f*:

$$\begin{aligned} \bigl (r^{\kappa _1}-r_0^{\kappa _1}\bigr )f(r)\le \frac{\kappa _1 r_0^{\kappa _1} e^{K_1}}{\kappa _0}f(r_0)+\frac{C\kappa _1 e^{K_1}}{\kappa _0}H(r,r_0) \end{aligned}$$

where *H* is defined in (5.66) and since $H\le H_1<\infty$ is finite, the estimate for *f* follows. $\square$

##### Remark 5.17

(Application in Proof of Proposition 4.15) In Section 4.3 we have seen (5.64) to be true with

5.69 $$\begin{aligned} f(r):=\int _{\Sigma _r}{}^*P^q[W]\qquad \kappa (r) = 6-\frac{C}{r}\le \kappa _1=6 \qquad h=0 \end{aligned}$$

which implies the statement of Proposition 4.15,

5.70 $$\begin{aligned} r^{6}f(r)\le C r_0^{6} f(r_0)\,, \end{aligned}$$

because

5.71 $$\begin{aligned} K(r)\le \int _{r_0}^\infty \frac{C}{r^2}\mathrm {d}r<\infty \,. \end{aligned}$$

##### Remark 5.18

(Application in Proof of Prop. 5.19) In Section 5.3 we show that (5.64) also holds for

5.72a $$\begin{aligned}&f(r):=\int _{\Sigma _r}{}^*P^q[\tilde{{\mathcal {L}}}_{N}W] \end{aligned}$$

5.72b $$\begin{aligned}&\kappa (r) = 6\Bigl (1-\frac{C_0^2}{r}\Bigr )^3-\frac{CC_0^2}{r}\le \kappa _1=6 \qquad h(r)=\frac{C_0}{r}\int _{\Sigma _r}{}^*P^q[W]\qquad \qquad \end{aligned}$$

Note that with these choices

5.73 $$\begin{aligned} K(r)=\int _{r_0}^r\frac{\kappa _1-\kappa (r)}{r}\mathrm {d}r\le \int _{r_0}^{\infty }\frac{C}{r}\frac{C_0^2}{r}\mathrm {d}r<\infty \end{aligned}$$

and using (5.70),

5.74 $$\begin{aligned} H(r,r_0)\le C_0 \int _{r_0}^{\infty } \int _{\Sigma _r}{}^*P^q[W] r^4 \mathrm {d}r <\infty \,. \end{aligned}$$

Therefore $r^{6}f(r)<\infty$ which is statement of Prop. 5.19 below.

#### Conclusions

We conclude this Section with the main estimate for the energy flux associated to the solution $\tilde{{\mathcal {L}}}_{N}W$ of (5.6). The proof will also make use of the main estimate of Section 6.

##### Proposition 5.19

Assume (**BA:I**), (**BA:II**) and (**BA:III**.i,ii) hold for some $C_0>0$. Then there exists a constant $C>0$ so that for all solutions *W* to (1.27), we have

5.75 $$\begin{aligned} r^{6}\int _{\Sigma _r}{}^*P^q[\tilde{{\mathcal {L}}}_{N}W] + r^{6}\int _{\Sigma _r}{}^*P^q[W] \le C \int _{\Sigma _{r_0}}{}^*P^q[\tilde{{\mathcal {L}}}_{N}W] + C \int _{\Sigma _{r_0}}{}^*P^q[W]\nonumber \\ \end{aligned}$$

for all $$r\ge r_0$$.

##### Proof

We apply the energy identity of Prop. 4.1 to the current $P^q[\tilde{{\mathcal {L}}}_{N}W]$ on a domain (4.11). The “bulk term” contains on one hand

$$\begin{aligned} \int _{{\mathcal {D}}}K^q[\tilde{{\mathcal {L}}}_{N}W]\mathrm {d}\mu _{g}\ge 6\int \frac{\mathrm {d}r}{r}\int _{\Sigma _r}\bigl (1-C_0\Omega ^{-1}\bigr )^3{}^*P^q[\tilde{{\mathcal {L}}}_{N}W] \end{aligned}$$

where we applied Lemma 4.14 — which is independent of the “Weyl field” and only an algebraic property of the compatible current $P^q$ — and on the other hand the “divergence term”

$$\begin{aligned} \int _{\mathcal {D}}\bigl ({{\,\mathrm{div}\,}}&Q[\tilde{{\mathcal {L}}}_{N}W]\bigr )(M_q,M_q,M_q)\mathrm {d}\mu _{g}\\&\quad =\,\int \mathrm {d}r\int _{\Sigma _r}\phi \bigl ( {{\,\mathrm{div}\,}}Q[\tilde{{\mathcal {L}}}_{N}W] \bigr )(M_q,M_q,M_q)\mathrm {d}\mu _{{\overline{g}}_{r}} \end{aligned}$$

and by our Prop. 5.11,

$$\begin{aligned}&\int _{\Sigma _r} \phi \bigl ({{\,\mathrm{div}\,}}Q[\tilde{{\mathcal {L}}}_{N}W]\bigr )(M_q,M_q,M_q) \mathrm {d}\mu _{{\overline{g}}_{r}} \\&\quad \ge -\frac{C}{r}\int _{\Sigma _r}\frac{C_0}{\Omega }\Bigl [{}^*P^q[\tilde{{\mathcal {L}}}_{N}W]+{}^*P^q[W]\Bigr ] -\frac{C}{r}\int _{\Sigma _r}\frac{C_0}{\Omega }\Omega ^2{P\!\!\!\!/\,}^q\mathrm {d}\mu _{{\overline{g}}_{r}}\,. \end{aligned}$$

Let us now invoke (${\varvec{BA:III}}.i$), namely the assumption that $\Omega$ and *r* are comparable. Then by Corollary 6.5 below — cf. (5.24) for the definition of ${P\!\!\!\!/\,}$, and Section 6 for the defintions of *E*, *H* —

$$\begin{aligned}&\int _{\Sigma _r}\frac{1}{\Omega }\Omega ^2{P\!\!\!\!/\,}^q\mathrm {d}\mu _{{\overline{g}}_{r}}\le C_0\frac{1}{r}\int _{\Sigma _r}\frac{\Omega ^2}{(2\Omega )^3}\Bigl [|\nabla \!\!\!\!/{\underline{\alpha }}_q |^2 \\&\quad +\, |\nabla \!\!\!\!/{\underline{\beta }}_q |^2 + |\nabla \!\!\!\!/\rho |^2 + |\nabla \!\!\!\!/\sigma |^2 + |\nabla \!\!\!\!/\beta _q|^2 +|\nabla \!\!\!\!/\alpha _q|^2\Bigr ]\\&\quad \le C_0\frac{1}{r}\frac{1}{(2r)^3}\int _{\Sigma _r}|{\overline{\nabla }}(rE) |^2+|{\overline{\nabla }}(rH)|^2\mathrm {d}\mu _{{\overline{g}}_{r}}\\&\quad \le \frac{C_0}{r} \int _{\Sigma _r} \frac{1}{(2\Omega )^3}\Bigl [|{\tilde{{\underline{\alpha }}}}|^2+|{\tilde{{\underline{\beta }}}}|^2+{\tilde{\rho }}^2+{\tilde{\sigma }}^2+|{\tilde{\beta }}|^2+|{\tilde{\alpha }}|^2\Bigr ]\mathrm {d}\mu _{{\overline{g}}_{r}}\\&\quad +\,\frac{C_0}{r} \int _{\Sigma _r} \frac{1}{(2\Omega )^3}\Bigl [|{\underline{\alpha }}|^2+|{\underline{\beta }}|^2+\rho ^2+\sigma ^2+|\beta |^2+|\alpha |^2\Bigr ]\mathrm {d}\mu _{{\overline{g}}_{r}}\\&\quad \le \frac{C_0}{r}\int _{\Sigma _r}{}^*P^q[\tilde{{\mathcal {L}}}_{N}W]+{}^*P^q[W] \end{aligned}$$

Therefore

$$\begin{aligned} \int _{\Sigma _r} \phi \bigl ({{\,\mathrm{div}\,}}Q[\tilde{{\mathcal {L}}}_{N}W]\bigr )(M_q,M_q,M_q) \mathrm {d}\mu _{{\overline{g}}_{r}}\ge -\frac{C C_0^2}{r^2}\int _{\Sigma _r}{}^*P^q[\tilde{{\mathcal {L}}}_{N}W]+{}^*P^q[W] \end{aligned}$$

The energy

$$\begin{aligned} g(r) := \int _{\Sigma _r}{}^*P^q[\tilde{{\mathcal {L}}}_{N}W] \end{aligned}$$

thus satisfies the integral inequality

$$\begin{aligned} g(r_2)+\int _{r_1}^{r_2}\Bigl [\frac{\kappa (r)}{r} g(r)-\frac{C}{r^2}f(r)\Bigr ] \mathrm {d}r \le g(r_1) \end{aligned}$$

where

$$\begin{aligned}&\kappa (r):= 6\Bigl (1-\frac{C_0^2}{r}\Bigr )^3-\frac{CC_0^2}{r}\qquad \kappa _1:=6\\&f(r) := \int _{\Sigma _r}{}^*P^q[W]\,. \end{aligned}$$

Since also by Prop. 4.15, $r^{6}f(r)\le C r_0^{6}f(r_0)$, the argument of Sections 5.4.1 applies, because here

$$\begin{aligned} H(r,r_0)=\int _{r_0}^r\frac{1}{r} f(r) r^5 \mathrm {d}r \le r_0^5 f(r_0) \end{aligned}$$

and the statement of the Proposition follows. $\square$

## Electromagnetic decomposition

The electromagnetic formalism is a decomposition of the Weyl curvature — and more generally of any “Weyl field” — into “electric” and “magnetic” parts relative to a space-like hypersurface $\Sigma$.^44^ The decomposition with respect to $\Sigma _r$ — which we use in addition to the null decomposition of *W* introduced in Section 2.4 — occurs naturally in the redshift estimate of Sections 4.3, 5, and is also necessary to control the remaining error that we have seen in Section 5.3. The electromagnetic decomposition has been used in [10], which can serve as a reference for some of the results that we shall discuss.

In Section 6.1 we discuss the from of the Bianchi equations relative to the electromagnetic decomposition, in Section 6.2 its relation to the null decomposition, and in Section 6.3 an elliptic estimate for the resulting system on $\Sigma _r$.

### Bianchi equations

We consider a domain foliated by the spacelike level sets of the area radius *r*:

6.1 $$\begin{aligned} {\mathcal {D}}=\bigcup \Sigma _r \end{aligned}$$

where each leaf $\Sigma _r$ is diffeomorphic to a cylinder ${\mathbb {R}}\times {\mathbb {S}}^2$. We denote by *n* the time-like unit normal to $\Sigma _r$, and by $\phi$ the associated lapse function of the foliation, cf. Section 2.5.

The *electromagnetic decomposition* of a Weyl field *W* consists of a pair of symmetric trace-less $\Sigma _r$-tangent 2-tensors

6.2a $$\begin{aligned}&E[W]:=ii_n[W] \qquad H[W]:=ii_n[{}^*W] \end{aligned}$$

6.2b $$\begin{aligned}&E[W](X,Y)=W(n,X,n,Y)\quad H[W](X,Y)={}^*W(n,X,n,Y)\,; \end{aligned}$$

we refer the reader to Section 4 of [9] for precise definitions — see in particular (4.4) therein — and for a more detailed discussion of this decomposition. In particular, we recall that *E*[*W*] and *H*[*W*] *together completely determine* the Weyl field *W* as can be seen from the formula (4.3) in [9]. Furthermore it follows directly from (4.22) in [9] that with *E*[*W*] and *H*[*W*] as defined in (6.2), and *n* given by (4.74), we have

6.3 $$\begin{aligned} |E[W]|^2+|H[W]|^2=\frac{1}{8}|{\underline{\alpha }}_q|^2+|{\underline{\beta }}_q|^2+\frac{3}{2}\rho ^2+\frac{3}{2}\sigma ^2+|\beta _q|^2+\frac{1}{8}|\alpha _q|^2\,. \end{aligned}$$

Following Chapter 7.2 in [10] we will derive that *E*, and *H*, satisfy — *in the frame discussed in Section* 2.5 — the following equations:

6.4a $$\begin{aligned}&{{\,\mathrm{div}\,}}E=H\wedge k \end{aligned}$$

6.4b $$\begin{aligned}&\widehat{{\mathcal {L}}_nH}+{{\,\mathrm{curl}\,}}E={\overline{\nabla }}\log \phi \wedge E-\frac{1}{2}k\times H \end{aligned}$$

6.4c $$\begin{aligned}&{{\,\mathrm{div}\,}}H= -E\wedge k \end{aligned}$$

6.4d $$\begin{aligned}&-\widehat{{\mathcal {L}}_nE}+{{\,\mathrm{curl}\,}}H={\overline{\nabla }}\log \phi \wedge H+\frac{1}{2}k\times E \end{aligned}$$

where $\widehat{{\mathcal {L}}_nH}_{ij}={\mathcal {L}}_nH_{ij}+\frac{2}{3}(k\cdot H){{\overline{g}}_{}}_{ij}$, *k* is the second fundamental form of $\Sigma _r$, ‘$\times$’ is the product defined in (4.4.17) in [10] and ‘$\wedge$’ in (7.2.2e-) therein. More explicitly, (6.4b) can also be expressed as:

6.4e $$\begin{aligned} \frac{1}{\phi }\frac{\partial H_{ij}}{\partial r}+({{\,\mathrm{curl}\,}}E)_{ij}=({\overline{\nabla }}\log \phi \wedge E)_{ij}-\frac{1}{2}(H\times k)_{ij}+\frac{2}{3}(H\cdot k) {\overline{g}}_{ij} \end{aligned}$$

Note that, cf. (7.2.2) in [10],

$$\begin{aligned} \nabla _{k}W_{i0j0}= & {} {\overline{\nabla }}_k E_{ij}+\Bigl (\epsilon _{il}^{m} H_{mj}-\epsilon _{jl}^{m}H_{mi} \Bigr )k^l_k\\ \nabla _{k}{}^*W_{i0j0}= & {} {\overline{\nabla }}_k H_{ij}-\Bigl (\epsilon _{il}^{m} E_{mj}+\epsilon _{jl}^{m}E_{mi} \Bigr )k^l_k \end{aligned}$$

The equations (6.4a) and (6.4c) then follow directly by taking the trace and using (2.31). Moreover^45^

$$\begin{aligned} \nabla _0 W_{kij0}= & {} -\epsilon _{ki}^{l}\frac{1}{\phi }\frac{\partial H_{lj}}{\partial r}-{{\,\mathrm{tr}\,}}k \epsilon _{ki}^{ l} H_{lj} \\&\quad +\,\epsilon _{li}^{ m} k_k^l H_{mj}+\epsilon _{kl}^{ m} k_i^l H_{mj}+\epsilon _{ki}^{ m}k_j^l H_{ml}\\&\quad +\,({\overline{\nabla }}_k\log \phi ) E_{ij}-({\overline{\nabla }}_i \log \phi )E_{kj}-({\overline{\nabla }}^l\log \phi ) W_{kijl}\\ \nabla _k W_{i0j0}+\nabla _i W_{0kj0}= & {} \epsilon _{ki}^{l}\frac{1}{\phi }\frac{\partial H_{lj}}{\partial r}+{{\,\mathrm{tr}\,}}k \epsilon _{ki}^{ l} H_{lj} \\&\quad -\,\epsilon _{li}^{ m} k_k^l H_{mj}-\epsilon _{kl}^{ m} k_i^l H_{mj}-\epsilon _{ki}^{ m}k_j^l H_{ml}\\&\quad -\,({\overline{\nabla }}_k\log \phi ) E_{ij}+({\overline{\nabla }}_i \log \phi )E_{kj}+({\overline{\nabla }}^l\log \phi ) W_{kijl}\\ 2\epsilon _{n}^{ ki}\nabla _k W_{i0j0}= & {} 2\frac{1}{\phi }\frac{\partial H_{nj}}{\partial r} -2\epsilon _{n}^{ ki}({\overline{\nabla }}_k\log \phi ) E_{ij}-2\epsilon _{j}^{ lm}({\overline{\nabla }}_l\log \phi )E_{mn} \end{aligned}$$

From (7.2.2f):

$$\begin{aligned} \epsilon _{n}^{ ki}\nabla _k W_{i0j0}+\epsilon _{j}^{ ki}\nabla _k W_{i0n0}=-2({{\,\mathrm{curl}\,}}E)_{nj}-(H\times k)_{nj}+\frac{4}{3}(H\cdot k )g_{nj} \end{aligned}$$

This yields (6.4e), and thus (6.4b). Similarly for (6.4d).

### Relation to null decomposition

In this Section we discuss the relation between the electromagnetic and null decompositions. In particular, when (6.4) is viewed as a Hodge system on $\Sigma _r$,

6.7a $$\begin{aligned} {{\,\mathrm{div}\,}}E= & {} \rho _E\qquad {{\,\mathrm{curl}\,}}E=\sigma _H \end{aligned}$$

6.7b $$\begin{aligned} {{\,\mathrm{div}\,}}H= & {} \rho _H\qquad {{\,\mathrm{curl}\,}}H=\sigma _E \end{aligned}$$

we establish that the “source terms” coincide — *including the lower order terms* — with components of the modified Lie derivative $\tilde{{\mathcal {L}}}_{N}W$. This is a non-trivial consequence of our choice of *N* in (5.4), and is important for the application of the elliptic estimate of Section 6.3.

Recall first

$$\begin{aligned}n=\frac{1}{2}(e_3+e_4)\,,\end{aligned}$$

and also define

6.8 $$\begin{aligned} X=\frac{1}{2}\bigl (e_3-e_4\bigr ) \end{aligned}$$

then

$$\begin{aligned}e_3=n+X\qquad e_4=n-X\,.\end{aligned}$$

We record, cf. (7.3.3e) in [10],

6.9a $$\begin{aligned} E_{XX}= & {} \rho \qquad H_{XX}=\sigma \end{aligned}$$

6.9b $$\begin{aligned} E_{AX}= & {} \frac{1}{2}{\underline{\beta }}_A+\frac{1}{2}\beta _A\qquad H_{AX}=\frac{1}{2}{}^*{\underline{\beta }}_A-\frac{1}{2}{}^*\beta _A \end{aligned}$$

6.9c $$\begin{aligned} E_{AB}= & {} \frac{1}{4}\alpha _{AB}+\frac{1}{4}{\underline{\alpha }}_{AB}-\frac{1}{2}\rho g\!\!\!/_{AB}\qquad H_{AB}=-\frac{1}{4}{}^*\alpha _{AB}+\frac{1}{4}{}^*{\underline{\alpha }}_{AB}-\frac{1}{2}\sigma g\!\!\!/_{AB}\nonumber \\ \end{aligned}$$

We now compare the components of $\widehat{{\mathcal {L}}_nE}$, $\widehat{{\mathcal {L}}_nH}$ to the null components of the modified Lie derivative of *W* with respect to $N=\Omega n$.

#### Lemma 6.1

For any Weyl field *W* decomposed into electric and magnetic parts *E*, and *H* with respect to the normal *n*, we have the following relations to the null decomposition of $\tilde{{\mathcal {L}}}_{N}W$ where $N=\Omega n$:

6.10a $$\begin{aligned}&\Bigl |\widehat{{\mathcal {L}}_nE}(X,X)+\frac{1}{2}k\times E - \frac{1}{\Omega } \rho [\tilde{{\mathcal {L}}}_{N}W] \Bigr |\le C \bigl |\hat{{\underline{L}}}q\nonumber \\&\quad +\,\hat{{L}}q^{-1}+q(2{\hat{{\underline{\omega }}}}-{{\,\mathrm{tr}\,}}{\underline{\chi }})+q^{-1}(2{\hat{\omega }}-{{\,\mathrm{tr}\,}}\chi )\bigr ||\rho |\nonumber \\&\quad +\,C|\bigl (\zeta +\mathrm {d}\!\!\!/\log q,\beta +{\underline{\beta }}\bigr )|+C|\Bigl ( q{\hat{{\underline{\chi }}}}+q^{-1}{\hat{\chi }},\alpha +{\underline{\alpha }}\Bigr )|\end{aligned}$$

6.10b $$\begin{aligned}&\Bigl |\widehat{{\mathcal {L}}_nE}(e_A,X)+\frac{1}{2}(k\times E)(e_A,X)-\frac{1}{2\Omega }\Bigl ({\underline{\beta }}_q[\tilde{{\mathcal {L}}}_{N}W]+\beta _q[\tilde{{\mathcal {L}}}_{N}W]\Bigr ) \Bigr |\nonumber \\&\quad \le \bigl |\hat{{\underline{L}}}q+\hat{{L}}q^{-1}\bigr |\bigl (|{\underline{\beta }}|+|\beta |\bigr )\nonumber \\&\quad +2\bigl (|\eta |+|{\underline{\eta }}|+|\mathrm {d}\!\!\!/\log q|\bigr )|\rho |+\bigl (|\alpha |+|{\underline{\alpha }}|\bigr ) \bigl (|\eta |+|{\underline{\eta }}|+|\mathrm {d}\!\!\!/\log q|\bigr )\nonumber \\&\quad +\,\bigl (q|{\hat{{\underline{\chi }}}}|+q^{-1}|{\hat{\chi }}|\bigr )\bigl (|{\underline{\beta }}|+|\beta |\bigr ) \end{aligned}$$

6.10c $$\begin{aligned}&\Bigl |\widehat{{\mathcal {L}}_nE}+\frac{1}{2}(k\times E)+\frac{1}{2}\bigl ( \widehat{{\mathcal {L}}_nE}(X,X)+\frac{1}{2}(k\times E)(X,X) \bigr ) g\!\!\!/\nonumber \\&\quad -\,\frac{1}{4\Omega }\bigl (\alpha _q[\tilde{{\mathcal {L}}}_{N}W]+{\underline{\alpha }}_q[\tilde{{\mathcal {L}}}_{N}W]\bigr )\Bigr |_{g\!\!\!/}\le \nonumber \\&\quad \le q\bigl |2{\hat{{\underline{\omega }}}}-{{\,\mathrm{tr}\,}}{\underline{\chi }}\bigr | |\rho |+q^{-1}\bigl |2{\hat{\omega }}-{{\,\mathrm{tr}\,}}\chi \bigr | |\rho |+|\hat{{\underline{L}}}q+\hat{{L}}q^{-1} ||\rho |\nonumber \\&\quad +\,\bigl (q|{\hat{{\underline{\chi }}}}|+q^{-1}|{\hat{\chi }}|\bigr )|\rho |\nonumber \\&\quad +\Bigl (q\bigl |{{\,\mathrm{tr}\,}}{\underline{\chi }}-2{\hat{{\underline{\omega }}}}\bigr |+q^{-1}\bigl |{{\,\mathrm{tr}\,}}\chi -2{\hat{\omega }}\bigr |+|\hat{{\underline{L}}}q|+|\hat{{L}}q^{-1}|+q|{\hat{{\underline{\chi }}}}|+q^{-1}|{\hat{\chi }}|\Bigr )\nonumber \\&\quad \bigl (|\alpha _q|+|{\underline{\alpha }}_q|\bigr )\nonumber \\&\quad +2\bigl (|\zeta |+|\mathrm {d}\!\!\!/\log q|\bigr )\bigl (|\beta _q|+|{\underline{\beta }}_q|\bigr ) \end{aligned}$$

Similarly for the magnetic part *H*.

#### Proof

Note first

$$\begin{aligned} g(X,X)=1\qquad g(X,e_A)=0 \end{aligned}$$

Define

$$\begin{aligned} {\overline{g}}_{r}(E^\sharp \cdot Y,Z)=E(Y,Z) \end{aligned}$$

then

$$\begin{aligned} E^\sharp \cdot X= & {} \rho X+\frac{1}{2}{\underline{\beta }}_Ae_A+\frac{1}{2}\beta _A e_A\\ E^\sharp \cdot e_A= & {} \frac{1}{2}\bigl ({\underline{\beta }}_A+\beta _A) X+\frac{1}{4}\bigl (\alpha ^{\sharp B}_{A}+{\underline{\alpha }}^{\sharp B}_{A}\bigr )\cdot e_B-\frac{1}{2}\rho e_A \end{aligned}$$

Moreover

$$\begin{aligned} k^\sharp \cdot X= & {} \frac{1}{2}\bigl (q{\hat{{\underline{\omega }}}}+q^{-1}{\hat{\omega }}+\hat{{\underline{L}}}q+\hat{{L}}q^{-1}\bigr )X +\frac{1}{2}\eta ^\sharp -\frac{1}{2}{\underline{\eta }}^\sharp \\ k^\sharp \cdot e_A= & {} \frac{1}{2}\bigl (\eta _A-{\underline{\eta }}_A\bigr )X+\frac{1}{2}q{\underline{\chi }}^{\sharp B}_{A}e_B+\frac{1}{2}q^{-1}\chi ^{\sharp B}_{A}e_B \end{aligned}$$

because

$$\begin{aligned} k(X,X)= & {} g(\nabla _X n,X)=\frac{1}{2}\bigl (q{\hat{{\underline{\omega }}}}+q^{-1}{\hat{\omega }}+\hat{{\underline{L}}}q+\hat{{L}}q^{-1}\bigr )\\ k(X,e_A)= & {} \frac{1}{2}\eta _A-\frac{1}{2}{\underline{\eta }}_A\\ k(e_A,e_B)= & {} \frac{1}{2}q{\underline{\chi }}_{AB}+\frac{1}{2}q^{-1}\chi _{AB} \end{aligned}$$

Thus

$$\begin{aligned} g(k^\sharp \cdot X, E^\sharp \cdot X)= & {} \frac{1}{2}\bigl (q{\hat{{\underline{\omega }}}}+q^{-1}{\hat{\omega }}+\hat{{\underline{L}}}q+\hat{{L}}q^{-1}\bigr )\rho +\frac{1}{4}\bigl (\eta -{\underline{\eta }},\beta +{\underline{\beta }}\bigr ) \\ g(k^\sharp \cdot e_A, E^\sharp \cdot e_B\bigr )= & {} \frac{1}{4}\bigl (\eta _A-{\underline{\eta }}_A\bigr )\bigl (\beta _B+{\underline{\beta }}_B)+\frac{1}{8}\bigl (\alpha +{\underline{\alpha }}\bigr )_{B}^{\sharp C}\bigl (q{\hat{{\underline{\chi }}}}_{AC}+q^{-1}{\hat{\chi }}_{AC}\bigr )\\&-\frac{1}{4}\bigl (q{\hat{{\underline{\chi }}}}+q^{-1}{\hat{\chi }}\bigr )_{AB}\rho +\frac{1}{16}\bigl (q{{\,\mathrm{tr}\,}}{\underline{\chi }}+q^{-1}{{\,\mathrm{tr}\,}}\chi \bigr )\bigl (\alpha +{\underline{\alpha }}\bigr )_{AB} \\&\quad -\,\frac{1}{8}\bigl (q{{\,\mathrm{tr}\,}}{\underline{\chi }}+q^{-1}{{\,\mathrm{tr}\,}}\chi \bigr ) g\!\!\!/_{AB}\rho \\ g(k^\sharp \cdot e_A,E^\sharp \cdot X)= & {} \frac{1}{2}\bigl (\eta _A-{\underline{\eta }}_A\bigr )\rho +\frac{1}{4}\bigl (q{\underline{\chi }}+q^{-1}\chi \bigr )_A^\sharp \cdot \bigl ({\underline{\beta }}+\beta \bigr ) \\ g(k^\sharp \cdot X,E^\sharp \cdot e_A)= & {} \frac{1}{4}\bigl (q{\hat{{\underline{\omega }}}}+q^{-1}{\hat{\omega }}+\hat{{\underline{L}}}q+\hat{{L}}q^{-1}\bigr )\bigl ({\underline{\beta }}_A+\beta _A)\\&\quad +\,\frac{1}{8}\bigl (\alpha _A^\sharp +{\underline{\alpha }}_A^\sharp \bigr )\cdot \bigl (\eta -{\underline{\eta }}\bigr )-\frac{1}{4}\bigl (\eta _A-{\underline{\eta }}_A\bigr )\rho \\ k\cdot E= & {} k^{ij}E_{ij}=k(X,X)E(X,X)+g\!\!\!/^{AB}k(X,e_A)E(X,e_B)\\&\quad +\,g\!\!\!/^{AC}g\!\!\!/^{BD}k_{CD}E_{AB}\\= & {} \frac{1}{4}\bigl (2q{\hat{{\underline{\omega }}}}+2q^{-1}{\hat{\omega }}-q{{\,\mathrm{tr}\,}}{\underline{\chi }}-q^{-1}{{\,\mathrm{tr}\,}}\chi +2\hat{{\underline{L}}}q+2\hat{{L}}q^{-1}\bigr )\rho \\&\quad +\,\frac{1}{4}\bigl (\eta -{\underline{\eta }},\beta +{\underline{\beta }}\bigr )+\frac{1}{8}\Bigl ( q{\hat{{\underline{\chi }}}}+q^{-1}{\hat{\chi }},\alpha +{\underline{\alpha }}\Bigr ) \end{aligned}$$

and by Lemma 4.4.2 in [10]

$$\begin{aligned} \frac{1}{2}(k\times E)(X,X)= & {} g(k^\sharp \cdot X,E^\sharp \cdot X)-\frac{1}{3}(k\cdot E) g(X,X)\\= & {} \frac{1}{2}\frac{1}{6}\bigl (4q{\hat{{\underline{\omega }}}}+4q^{-1}{\hat{\omega }}+q{{\,\mathrm{tr}\,}}{\underline{\chi }}+q^{-1}{{\,\mathrm{tr}\,}}\chi +4\hat{{\underline{L}}}q+4\hat{{L}}q^{-1}\bigr )\rho \\&\quad +\,\frac{1}{6}\bigl (\eta -{\underline{\eta }},\beta +{\underline{\beta }}\bigr )\\&-\frac{1}{3}\frac{1}{8}\Bigl ( q{\hat{{\underline{\chi }}}}+q^{-1}{\hat{\chi }},\alpha +{\underline{\alpha }}\Bigr ) \\ (k\times E)(e_A,X)= & {} \frac{1}{4}\bigl (\eta _A-{\underline{\eta }}_A\bigr )\rho +\frac{1}{4}\bigl (q{\hat{{\underline{\chi }}}}+q^{-1}{\hat{\chi }}\bigr )_A^\sharp \cdot \bigl ({\underline{\beta }}+\beta \bigr )\\&+\frac{1}{8}\bigl (2q{\hat{{\underline{\omega }}}}+2q^{-1}{\hat{\omega }}+q{{\,\mathrm{tr}\,}}{\underline{\chi }}+q^{-1}{{\,\mathrm{tr}\,}}\chi +2\hat{{\underline{L}}}q\\&\quad +\,2\hat{{L}}q^{-1}\bigr )\bigl ({\underline{\beta }}_A+\beta _A)+\frac{1}{8}\bigl (\alpha _A^\sharp +{\underline{\alpha }}_A^\sharp \bigr )\cdot \bigl (\eta -{\underline{\eta }}\bigr ) \\ (k\times E)(e_A,e_B)= & {} \frac{1}{4}\bigl (\eta -{\underline{\eta }}\bigr ){\hat{\otimes }}\bigl (\beta +{\underline{\beta }}\bigr )_{AB}+\frac{1}{12}\bigl (\eta -{\underline{\eta }},\beta +{\underline{\beta }}\bigr )g\!\!\!/_{AB}\\&+\frac{1}{24}\bigl (\alpha +{\underline{\alpha }},q{\hat{{\underline{\chi }}}}+q^{-1}{\hat{\chi }}\bigr )g\!\!\!/_{AB} -\frac{1}{2}\bigl (q{\hat{{\underline{\chi }}}}+q^{-1}{\hat{\chi }}\bigr )_{AB}\rho \\&\quad +\,\frac{1}{8}\bigl (q{{\,\mathrm{tr}\,}}{\underline{\chi }}+q^{-1}{{\,\mathrm{tr}\,}}\chi \bigr )\bigl (\alpha +{\underline{\alpha }}\bigr )_{AB}\\&-\frac{1}{3}\frac{1}{4}\bigl (4q{\hat{{\underline{\omega }}}}+4q^{-1}{\hat{\omega }}+q{{\,\mathrm{tr}\,}}{\underline{\chi }}+q^{-1}{{\,\mathrm{tr}\,}}\chi \!+\!4\hat{{\underline{L}}}q\!+\!4\hat{{L}}q^{-1}\bigr )g\!\!\!/_{AB}\rho \end{aligned}$$

Finally since

$$\begin{aligned} {[}n,X]= & {} -\frac{1}{2}\Bigl (2\eta -2{\underline{\eta }}-\bigl (q{\hat{{\underline{\omega }}}}+\hat{{\underline{L}}}q\bigr )e_4+\bigl (q^{-1}{\hat{\omega }}+\hat{{L}}q^{-1}\bigr )e_3\Bigr )\\ {[}n,e_A]= & {} \frac{1}{2}\bigl (\nabla \!\!\!\!/_3e_A+\nabla \!\!\!\!/_4 e_A\bigr )+\frac{1}{2}\eta _A e_3+\frac{1}{2}{\underline{\eta }}_A e_4\\&\quad -\,\frac{1}{2}\bigl (q{\underline{\chi }}+q^{-1}\chi \bigr )_A^\sharp -\bigl (\zeta +\mathrm {d}\!\!\!/\log q\bigr )_AX \end{aligned}$$

we have

$$\begin{aligned} {\mathcal {L}}_nE(X,X)= & {} n\rho +\bigl (\eta -{\underline{\eta }},\beta +{\underline{\beta }}\bigr )+\bigl (q{\hat{{\underline{\omega }}}}+q^{-1}{\hat{\omega }}+\hat{{\underline{L}}}q+\hat{{L}}q^{-1}\bigr )\rho \\ {\mathcal {L}}_nE(e_A,X)= & {} \frac{1}{4}\frac{1}{\Omega }\Bigl ({\underline{D}}{\underline{\beta }}+{\underline{D}}\beta +D{\underline{\beta }}+D\beta \Bigr )\\&\quad +\,\frac{1}{4}\bigl (q{\hat{{\underline{\omega }}}}+q^{-1}{\hat{\omega }}+\hat{{\underline{L}}}q+\hat{{L}}q^{-1}\bigr )\bigl ({\underline{\beta }}_A+\beta _A\bigr )\\&+\frac{1}{2}\bigl (\zeta +\mathrm {d}\!\!\!/\log q-2\eta +2{\underline{\eta }}\bigr )_A\rho +\frac{1}{4}\bigl (\alpha +{\underline{\alpha }}\bigr )_A^\sharp \cdot \bigl (\eta -{\underline{\eta }}\bigr )\\ {\mathcal {L}}_nE(e_A,e_B)= & {} \frac{1}{8}\frac{1}{\Omega }\Bigl ({\hat{{\underline{D}}}}\alpha +{\hat{{\underline{D}}}}{\underline{\alpha }}+{\hat{D}}\alpha +{\hat{D}}{\underline{\alpha }}\bigr )\\&\quad -\,\frac{1}{4}\Bigl (2 n \rho +q{{\,\mathrm{tr}\,}}{\underline{\chi }}\rho +q^{-1}{{\,\mathrm{tr}\,}}\chi \rho \Bigr )g\!\!\!/_{AB}\\&\quad -\,\frac{1}{4}\bigl (\eta _A-{\underline{\eta }}_A-4\zeta -4\mathrm {d}\!\!\!/\log q\bigr )\bigl ({\underline{\beta }}_B+\beta _B\bigr ) \\&\quad -\,\frac{1}{4}\bigl (\eta _B-{\underline{\eta }}_B-4\zeta -4\mathrm {d}\!\!\!/\log q\bigr )\bigl ({\underline{\beta }}_A+\beta _A\bigr )\\&\quad +\,\frac{1}{8}\Bigl (q{\hat{{\underline{\chi }}}}+q^{-1}{\hat{\chi }},{\underline{\alpha }}+\alpha \Bigr )g\!\!\!/_{AB} \end{aligned}$$

and thus

$$\begin{aligned} \widehat{{\mathcal {L}}_nE}(X,X)= & {} {\mathcal {L}}_nE(X,X)+\frac{2}{3}(k\cdot E)g(X,X)\\&=n\rho +\frac{7}{6}\bigl (\eta -{\underline{\eta }},\beta +{\underline{\beta }}\bigr )+\frac{1}{3}\frac{1}{4}\Bigl ( q{\hat{{\underline{\chi }}}}+q^{-1}{\hat{\chi }},\alpha +{\underline{\alpha }}\Bigr )\\&+\frac{1}{3}\frac{1}{2}\bigl (8q{\hat{{\underline{\omega }}}}+8q^{-1}{\hat{\omega }}-q{{\,\mathrm{tr}\,}}{\underline{\chi }}-q^{-1}{{\,\mathrm{tr}\,}}\chi +8\hat{{\underline{L}}}q+8\hat{{L}}q^{-1}\bigr )\rho \\ \widehat{{\mathcal {L}}_nE}(e_A,e_B)= & {} {\mathcal {L}}_nE(e_A,e_B)+\frac{2}{3}(k\cdot E)g_{AB}=\frac{1}{8}\frac{1}{\Omega }\Bigl ({\hat{{\underline{D}}}}\alpha +{\hat{{\underline{D}}}}{\underline{\alpha }}+{\hat{D}}\alpha +{\hat{D}}{\underline{\alpha }}\bigr )_{AB}\\&-\frac{1}{2} n \rho g\!\!\!/_{AB} +\frac{1}{3}\frac{1}{4}\bigl (4q{\hat{{\underline{\omega }}}}+4q^{-1}{\hat{\omega }}\\&\quad -\,5q{{\,\mathrm{tr}\,}}{\underline{\chi }}-5q^{-1}{{\,\mathrm{tr}\,}}\chi +4\hat{{\underline{L}}}q+4\hat{{L}}q^{-1}\bigr )g\!\!\!/_{AB}\rho \\&-\frac{1}{4}\bigl (\eta -{\underline{\eta }}\bigr ){\hat{\otimes }}\bigl ({\underline{\beta }}+\beta \bigr )_{AB} \\&\quad +\,\bigl (\zeta _A+\mathrm {d}\!\!\!/_A\log q\bigr )\bigl ({\underline{\beta }}_B+\beta _B\bigr ) +\bigl (\zeta _B+\mathrm {d}\!\!\!/_B\log q\bigr )\bigl ({\underline{\beta }}_A+\beta _A\bigr )\\&+\frac{5}{24}\Bigl (q{\hat{{\underline{\chi }}}}+q^{-1}{\hat{\chi }},{\underline{\alpha }}+\alpha \Bigr )g\!\!\!/_{AB} -\frac{1}{12}\bigl (\eta -{\underline{\eta }},\beta +{\underline{\beta }}\bigr )g\!\!\!/_{AB} \end{aligned}$$

Therefore

$$\begin{aligned}&\widehat{{\mathcal {L}}_nE}(X,X)+\frac{1}{2}(k\times E)(X,X)=n\rho +\frac{4}{3}\bigl (\eta -{\underline{\eta }},\beta +{\underline{\beta }}\bigr )\\&\quad +\,\frac{1}{3}\frac{1}{8}\Bigl ( q{\hat{{\underline{\chi }}}}+q^{-1}{\hat{\chi }},\alpha +{\underline{\alpha }}\Bigr )\\&\quad +\,\frac{1}{6}\frac{1}{2}\bigl (20q{\hat{{\underline{\omega }}}}+20q^{-1}{\hat{\omega }}-q{{\,\mathrm{tr}\,}}{\underline{\chi }}-q^{-1}{{\,\mathrm{tr}\,}}\chi +20\hat{{\underline{L}}}q+20\hat{{L}}q^{-1}\bigr )\rho \\&\quad \widehat{{\mathcal {L}}_nE}(e_A,X)+\frac{1}{2}(k\times E)(e_A,X)=\frac{1}{4}\frac{1}{\Omega }\Bigl ({\underline{D}}{\underline{\beta }}+{\underline{D}}\beta +D{\underline{\beta }}+D\beta \Bigr )\\&\quad +\,\frac{1}{16}\bigl (6q{\hat{{\underline{\omega }}}}+6q^{-1}{\hat{\omega }}+q{{\,\mathrm{tr}\,}}{\underline{\chi }}+q^{-1}{{\,\mathrm{tr}\,}}\chi +6\hat{{\underline{L}}}q+6\hat{{L}}q^{-1}\bigr )\bigl ({\underline{\beta }}_A+\beta _A\bigr )\\&\quad +\,\frac{1}{8}\bigl (4\zeta +4\mathrm {d}\!\!\!/\log q-7\eta +7{\underline{\eta }}\bigr )_A\rho +\frac{5}{16}\bigl (\alpha _A^\sharp +{\underline{\alpha }}_A^\sharp \bigr )\cdot \bigl (\eta -{\underline{\eta }}\bigr )\\&\quad +\,\frac{1}{8}\bigl (q{\hat{{\underline{\chi }}}}+q^{-1}{\hat{\chi }}\bigr )_A^\sharp \cdot \bigl ({\underline{\beta }}+\beta \bigr ) \\&\quad \widehat{{\mathcal {L}}_nE}(e_A,e_B)+\frac{1}{2}(k\times E)(e_A,e_B)+ \frac{1}{2}\widehat{{\mathcal {L}}_nE}(X,X)g\!\!\!/_{AB}+\frac{1}{4}(k\times E)(X,X) g\!\!\!/_{AB}\\&\quad =\frac{1}{8}\frac{1}{\Omega }\Bigl ({\hat{{\underline{D}}}}\alpha +{\hat{{\underline{D}}}}{\underline{\alpha }}+{\hat{D}}\alpha +{\hat{D}}{\underline{\alpha }}\bigr ) +\frac{1}{2}\bigl (2q{\hat{{\underline{\omega }}}}+2q^{-1}{\hat{\omega }}-q{{\,\mathrm{tr}\,}}{\underline{\chi }}\\&\quad -\,q^{-1}{{\,\mathrm{tr}\,}}\chi +2\hat{{\underline{L}}}q+2\hat{{L}}q^{-1}\bigr )g\!\!\!/_{AB}\rho \\&\quad -\,\frac{1}{8}\bigl (\eta -{\underline{\eta }}\bigr ){\hat{\otimes }}\bigl ({\underline{\beta }}+\beta \bigr )_{AB}\\&\quad +\,\bigl (\zeta _A+\mathrm {d}\!\!\!/_A\log q\bigr )\bigl ({\underline{\beta }}_B+\beta _B\bigr ) +\bigl (\zeta _B+\mathrm {d}\!\!\!/_B\log q\bigr )\bigl ({\underline{\beta }}_A+\beta _A\bigr )\\&\quad +\,\frac{1}{4}\Bigl (q{\hat{{\underline{\chi }}}}+q^{-1}{\hat{\chi }},{\underline{\alpha }}+\alpha \Bigr )g\!\!\!/_{AB} +\frac{5}{8}\bigl (\eta -{\underline{\eta }},\beta +{\underline{\beta }}\bigr )g\!\!\!/_{AB}\\&\quad -\,\frac{1}{4}\bigl (q{\hat{{\underline{\chi }}}}+q^{-1}{\hat{\chi }}\bigr )_{AB}\rho +\frac{1}{16}\bigl (q{{\,\mathrm{tr}\,}}{\underline{\chi }}+q^{-1}{{\,\mathrm{tr}\,}}\chi \bigr )\bigl (\alpha +{\underline{\alpha }}\bigr )_{AB} \end{aligned}$$

and in comparison to Lemma 5.8:

$$\begin{aligned}&\widehat{{\mathcal {L}}_nE}(X,X)+\frac{1}{2}(k\times E)(X,X)= \frac{1}{\Omega } \rho [\tilde{{\mathcal {L}}}_{N}W]+\frac{4}{3}\bigl (\hat{{\underline{L}}}q+\hat{{L}}q^{-1}\bigr )\rho \\&+\frac{1}{6}\bigl (4\zeta -3\mathrm {d}\!\!\!/\log q,\beta +{\underline{\beta }}\bigr )+\frac{1}{3}\frac{1}{8}\Bigl ( q{\hat{{\underline{\chi }}}}+q^{-1}{\hat{\chi }},\alpha +{\underline{\alpha }}\Bigr )\\&\quad +\,\frac{11}{24}\bigl (q(2{\hat{{\underline{\omega }}}}-{{\,\mathrm{tr}\,}}{\underline{\chi }})+q^{-1}(2{\hat{\omega }}-{{\,\mathrm{tr}\,}}\chi )\bigr )\rho \\&4\widehat{{\mathcal {L}}_nE}(e_A,X)+2(k\times E)(e_A,X)=\frac{2}{\Omega }\Bigl ({\tilde{{\underline{\beta }}}}_q+{\tilde{\beta }}_q\Bigr )+\frac{5}{4}\bigl (\hat{{\underline{L}}}q+\hat{{L}}q^{-1}\bigr )\bigl ({\underline{\beta }}_A+\beta _A\bigr )\\&+\frac{1}{2}\bigl (\eta -{\underline{\eta }}+10\mathrm {d}\!\!\!/\log q\bigr )_A\rho +\frac{1}{4}\bigl (\alpha _A^\sharp +{\underline{\alpha }}_A^\sharp \bigr )\cdot \bigl (3\eta -3{\underline{\eta }}-2\mathrm {d}\!\!\!/\log q\bigr )\\&\quad +\,\frac{3}{2}\bigl (q{\hat{{\underline{\chi }}}}+q^{-1}{\hat{\chi }}\bigr )_A^\sharp \cdot \bigl ({\underline{\beta }}+\beta \bigr ) \\&8\widehat{{\mathcal {L}}_nE}(e_A,e_B)+4(k\times E)(e_A,e_B)+ 4\widehat{{\mathcal {L}}_nE}(X,X)g\!\!\!/_{AB}+2(k\times E)(X,X) g\!\!\!/_{AB}\\&=\frac{2}{\Omega }\Bigl ({\tilde{\alpha }}_q+{\tilde{{\underline{\alpha }}}}_q\bigr )_{AB} +4\bigl (2q{\hat{{\underline{\omega }}}}+2q^{-1}{\hat{\omega }}-q{{\,\mathrm{tr}\,}}{\underline{\chi }}-q^{-1}{{\,\mathrm{tr}\,}}\chi +2\hat{{\underline{L}}}q+2\hat{{L}}q^{-1}\bigr )g\!\!\!/_{AB}\rho \\&+\frac{1}{4}\bigl (3q{{\,\mathrm{tr}\,}}{\underline{\chi }}+3q^{-1}{{\,\mathrm{tr}\,}}\chi -6q{\hat{{\underline{\omega }}}}-6q^{-1}{\hat{\omega }}-7\hat{{\underline{L}}}q+\hat{{L}}q^{-1}\bigr )\alpha _{AB}\\&+\frac{1}{4}\bigl (3q{{\,\mathrm{tr}\,}}{\underline{\chi }}+3q^{-1}{{\,\mathrm{tr}\,}}\chi -6q^{-1}{\hat{\omega }}-6q{\hat{{\underline{\omega }}}}-7\hat{{L}}q^{-1}+\hat{{\underline{L}}}q\bigr ){\underline{\alpha }}_{AB}\\&+10\bigl (\zeta +\mathrm {d}\!\!\!/\log q\bigr ){\hat{\otimes }}\bigl ({\underline{\beta }}+\beta \bigr )_{AB}+2\mathrm {d}\!\!\!/\log q{\hat{\otimes }}\bigl ({\underline{\beta }}+\beta \bigr )_{AB}\\&+2\bigl (\zeta -4\mathrm {d}\!\!\!/\log q,\beta +{\underline{\beta }}\bigr )g\!\!\!/_{AB}+2\Bigl (q{\hat{{\underline{\chi }}}}+q^{-1}{\hat{\chi }},{\underline{\alpha }}+\alpha \Bigr )g\!\!\!/_{AB} -2\bigl (q{\hat{{\underline{\chi }}}}+q^{-1}{\hat{\chi }}\bigr )_{AB}\rho \end{aligned}$$

$$\square $$

Also note that

6.11 $$\begin{aligned} (E\wedge k)(X)=\frac{1}{4}\bigl ({}^*\eta -{}^*{\underline{\eta }},{\underline{\beta }}+\beta \bigr )+\frac{1}{8}\bigl (\alpha +{\underline{\alpha }}\bigr )\wedge \bigl (q{\hat{{\underline{\chi }}}}+q^{-1}{\hat{\chi }}\bigr ) \end{aligned}$$

contains no terms linear in $q{{\,\mathrm{tr}\,}}{\underline{\chi }}$, $q^{-1}{{\,\mathrm{tr}\,}}\chi$ due to the antisymmetry of the product. Similarly for the other components.

### Elliptic estimate for Maxwell system

We quote the following result from Section 4.4 in [10] for symmetric Hodge systems.

#### Lemma 6.2

(Corollary 4.4.2.1 in [10]) Let *E* and *H* be symmetric traceless tensors on a 3-dimensional Riemannian manifold $(\Sigma ,{\overline{g}}_{})$ which satisfy the system (6.7). Then there is a constant $C>0$ such that

6.12 $$\begin{aligned} \int _\Sigma \Bigl (|\nabla E|^2+|\nabla H|^2\Bigr )\mathrm {d}\mu _{{\overline{g}}_{}}\le & {} C \int _{\Sigma } |\rho _E|^2+|\rho _H|^2+|\sigma _E|^2+|\sigma _H|^2 \mathrm {d}\mu _{{\overline{g}}_{}}\nonumber \\&\quad +\,C\int _{\Sigma }|{{\,\mathrm{Ric}\,}}({\overline{g}}_{})|\bigl (|E|^2+|H|^2\bigr )\mathrm {d}\mu _{{\overline{g}}_{}} \end{aligned}$$

Here we will make for simplicity an assumption directly on the Ricci curvature of $\Sigma _r$: 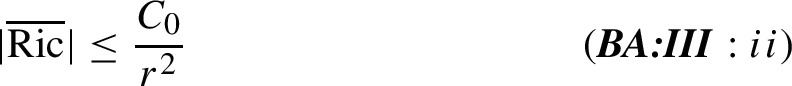 We note however that in view of the Gauss equations of the embedding of $\Sigma _r$ in $({\mathcal {D}},g)$ we can reduce this condition to an assumption on the scalar curvature of $\Sigma _r$.

#### Lemma 6.3

Suppose $(\Sigma ,{\overline{g}}_{r})$ is embedded as a spacelike hypersurface in $({\mathcal {M}},g)$, which is a solution to (1.1). Assume (**BA:I**.ii,iv-vii) hold for some $C_0>0$. Then there is a constant $C>0$,

6.13 $$\begin{aligned} |{\overline{{{\,\mathrm{Ric}\,}}}} |^2 \le C\Bigl ( \frac{1}{\Omega ^2}+{\overline{R}}^2+|W|^2\Bigr ) \end{aligned}$$

#### Remark 6.4

While this estimate shows that the assumption (${{\varvec{BA:III}}:ii}$) is consistent with (${\varvec{BA:III}}.i$), and the decay properties of the Weyl curvature, we point out that the assumption (${{\varvec{BA:III}}:ii}$) is not redundant in the context of the assumptions we have already made. In fact, to retrieve (${{\varvec{BA:III}}:ii}$) directly from the assumptions on the null second fundamental form we would have to make the stronger assumption that

6.14 $$\begin{aligned} \Omega ^2 \bigl (2{\hat{\omega }}-{{\,\mathrm{tr}\,}}\chi \bigr )\le C\qquad \Omega ^2\bigl (2{\hat{{\underline{\omega }}}}-{{\,\mathrm{tr}\,}}{\underline{\chi }}\bigr )\le C \end{aligned}$$

which reflects the behavior in (Schwarzschild-)de Sitter, but goes beyond the assumptions already made in (${{\varvec{BA:I}}.ii}$).

#### Proof

Recall the Gauss equation

$$\begin{aligned} {\overline{{{\,\mathrm{Ric}\,}}}}_{ij}+{{\,\mathrm{tr}\,}}k k_{ij}-k_{i}^{m}k_{mj}={{\,\mathrm{Ric}\,}}_{ij}+R_{0i0j} \end{aligned}$$

From the Einstein equations

$$\begin{aligned} {{\,\mathrm{Ric}\,}}_{ij}=\Lambda g_{ij} \end{aligned}$$

and with (2.5)

$$\begin{aligned} R_{0i 0j} = W_{0i 0j}-\frac{\Lambda }{3}g_{ij}\,. \end{aligned}$$

Then using the formulas obtained in the proof of Lemma 6.1,

$$\begin{aligned} {{\,\mathrm{tr}\,}}k= & {} \frac{1}{2}\bigl (q{\hat{{\underline{\omega }}}}+q^{-1}{\hat{\omega }}+q{{\,\mathrm{tr}\,}}{\underline{\chi }}+q^{-1}{{\,\mathrm{tr}\,}}\chi +\hat{{\underline{L}}}q+\hat{{L}}q^{-1}\bigr )\\ |k|^2= & {} \frac{1}{4}\bigl (q{\hat{{\underline{\omega }}}}+q^{-1}{\hat{\omega }}+\hat{{\underline{L}}}q+\hat{{L}}q^{-1}\bigr )^2+\frac{1}{4}|q{\hat{{\underline{\chi }}}}+q^{-1}{\hat{\chi }}|^2+\frac{1}{8}\bigl (q{{\,\mathrm{tr}\,}}{\underline{\chi }}+q^{-1}{{\,\mathrm{tr}\,}}\chi \bigr )^2 \end{aligned}$$

we have

$$\begin{aligned} {\overline{{{\,\mathrm{Ric}\,}}}}(X,X)= & {} -{{\,\mathrm{tr}\,}}k\, k(X,X)+g(k^\sharp \cdot X,k^\sharp \cdot X)+\frac{2}{3}\Lambda +E_{XX}\\ {\overline{{{\,\mathrm{Ric}\,}}}}(e_A,X)= & {} -{{\,\mathrm{tr}\,}}k\, k(X,e_A)+g(k^\sharp \cdot e_A,k^\sharp \cdot X)+E_{AX}\\ {\overline{{{\,\mathrm{Ric}\,}}}}_{AB}= & {} -{{\,\mathrm{tr}\,}}k\, k_{AB}+g(k^\sharp \cdot e_A,k^\sharp \cdot e_B)+\frac{2}{3}\Lambda g\!\!\!/_{AB}+E_{AB} \end{aligned}$$

Now the Gauss equation tells us

$$\begin{aligned} {\overline{R}}+({{\,\mathrm{tr}\,}}k)^2-|k|^2=2\Lambda \end{aligned}$$

so we can write

$$\begin{aligned} {\overline{{{\,\mathrm{Ric}\,}}}}(X,X)= & {} \frac{1}{3}{{\,\mathrm{tr}\,}}k\bigl ({{\,\mathrm{tr}\,}}k-3 k(X,X)\bigr )+\frac{1}{3}\bigl (3g(k^\sharp \cdot X,k^\sharp \cdot X)-|k|^2\bigr )+\frac{1}{3}{\overline{R}}+\rho \\ {\overline{{{\,\mathrm{Ric}\,}}}}(e_A,X)= & {} -{{\,\mathrm{tr}\,}}k\, k(X,e_A)+g(k^\sharp \cdot e_A,k^\sharp \cdot X)+\frac{1}{2}\bigl ({\underline{\beta }}+\beta \bigr )_A\\ {\overline{{{\,\mathrm{Ric}\,}}}}_{AB}= & {} \frac{1}{3}{{\,\mathrm{tr}\,}}k \bigl ({{\,\mathrm{tr}\,}}kg\!\!\!/_{AB}-3 k_{AB}\bigr )+\frac{1}{3}\bigl (3g(k^\sharp \cdot e_A,k^\sharp \cdot e_B)\\&\quad -\,|k|^2g\!\!\!/_{AB}\bigr )+\frac{1}{3}{\overline{R}}g\!\!\!/_{AB}+E_{AB} \end{aligned}$$

Since

$$\begin{aligned}&{{\,\mathrm{tr}\,}}k-3k(X,X)=\frac{1}{2}\bigl (q{{\,\mathrm{tr}\,}}{\underline{\chi }}+q^{-1}{{\,\mathrm{tr}\,}}\chi -2q{\hat{{\underline{\omega }}}}-2q^{-1}{\hat{\omega }}-2\hat{{\underline{L}}}q-2\hat{{L}}q^{-1}\bigr )\\&\quad 3g(k^\sharp \cdot X,k^\sharp \cdot X)-|k|^2= \frac{1}{8}\bigl (2q{\hat{{\underline{\omega }}}}+2q^{-1}{\hat{\omega }}-q{{\,\mathrm{tr}\,}}{\underline{\chi }}-q^{-1}{{\,\mathrm{tr}\,}}\chi \\&\quad +\,2\hat{{\underline{L}}}q+2\hat{{L}}q^{-1}\bigr )\times \\&\quad \times \bigl (2q{\hat{{\underline{\omega }}}}+2q^{-1}{\hat{\omega }}+q{{\,\mathrm{tr}\,}}{\underline{\chi }}+q^{-1}{{\,\mathrm{tr}\,}}\chi +2\hat{{\underline{L}}}q+2\hat{{L}}q^{-1}\bigr ) +3|\zeta |^2 \\&g(k^\sharp \cdot e_A,k^\sharp \cdot X)=\frac{1}{8}\bigl (2q{\hat{{\underline{\omega }}}}+2q^{-1}{\hat{\omega }}+q{{\,\mathrm{tr}\,}}{\underline{\chi }}+q^{-1}{{\,\mathrm{tr}\,}}\chi \\&\quad +\,2\hat{{\underline{L}}}q+2\hat{{L}}q^{-1}\bigr )\bigl (\eta _A-{\underline{\eta }}_A\bigr )\\&\quad +\,\frac{1}{4}\bigl (q{\hat{{\underline{\chi }}}}_A^\sharp +q^{-1}{\hat{\chi }}_A^\sharp \bigr )\cdot \bigl (\eta -{\underline{\eta }}\bigr ) \\&{{\,\mathrm{tr}\,}}kg\!\!\!/_{AB}-3 k_{AB}=\frac{1}{4}\bigl (2q{\hat{{\underline{\omega }}}}+2q^{-1}{\hat{\omega }}-q{{\,\mathrm{tr}\,}}{\underline{\chi }}-q^{-1}{{\,\mathrm{tr}\,}}\chi +2\hat{{\underline{L}}}q+2\hat{{L}}q^{-1}\bigr )g\!\!\!/_{AB}\\&\quad -\,\frac{3}{2}q{\hat{{\underline{\chi }}}}_{AB}+\frac{3}{2}q^{-1}{\hat{\chi }}_{AB} \\&3g(k^\sharp \cdot e_A,k^\sharp \cdot e_B)-|k|^2g\!\!\!/_{AB}= +\frac{3}{4}\bigl (q{{\,\mathrm{tr}\,}}{\underline{\chi }}+q^{-1}{{\,\mathrm{tr}\,}}\chi \bigr )\bigl (q{\hat{{\underline{\chi }}}}+q^{-1}{\hat{\chi }}\bigr )_{AB}\\&\quad +\,\frac{3}{4}\bigl (\eta _A-{\underline{\eta }}_A\bigr )\bigl (\eta _B-{\underline{\eta }}_B\bigr )+\frac{1}{8}|q{\hat{{\underline{\chi }}}}+q^{-1}{\hat{\chi }}|^2g\!\!\!/_{AB}\\&\quad +\,\frac{1}{16}\bigl (q{{\,\mathrm{tr}\,}}{\underline{\chi }}+q^{-1}{{\,\mathrm{tr}\,}}\chi -2q{\hat{{\underline{\omega }}}}-2q^{-1}{\hat{\omega }}-2\hat{{\underline{L}}}q-2\hat{{L}}q^{-1}\bigr )\times \\&\quad \times \bigl (q{{\,\mathrm{tr}\,}}{\underline{\chi }}+q^{-1}{{\,\mathrm{tr}\,}}\chi +2q{\hat{{\underline{\omega }}}}+2q^{-1}{\hat{\omega }}+2\hat{{\underline{L}}}q+2\hat{{L}}q^{-1}\bigr )g\!\!\!/_{AB} \end{aligned}$$

the result follows from

$$\begin{aligned} |{\overline{{{\,\mathrm{Ric}\,}}}} |^2 = \bigl ({\overline{{{\,\mathrm{Ric}\,}}}}(X,X)\bigr )^2+2g\!\!\!/^{AB}{\overline{{{\,\mathrm{Ric}\,}}}}(X,e_A){\overline{{{\,\mathrm{Ric}\,}}}}(X,e_B)+g\!\!\!/^{AC}g\!\!\!/^{BD}{\overline{{{\,\mathrm{Ric}\,}}}}_{AB}{\overline{{{\,\mathrm{Ric}\,}}}}_{CD}\,. \end{aligned}$$

$$\square $$

#### Corollary 6.5

Let *W* be a solution to (1.27), and *E* and *H* the electric and magnetic parts of *W* relative to $\Sigma _r$. Assume (**BA:I**,ii,iv-vi) and (${{\varvec{BA:III}}:ii}$) hold for some $C_0>0$. Then

6.15 $$\begin{aligned}&\int _{\Sigma _r}|{\overline{\nabla }}E|^2+|{\overline{\nabla }}H|^2\mathrm {d}\mu _{{\overline{g}}_{r}}\le C \int _{\Sigma _r} \frac{1}{\Omega ^2}\Bigl [|{\tilde{{\underline{\alpha }}}}|^2+|{\tilde{{\underline{\beta }}}}|^2+{\tilde{\rho }}^2+{\tilde{\sigma }}^2+|{\tilde{\beta }}|^2+|{\tilde{\alpha }}|^2\Bigr ]\mathrm {d}\mu _{{\overline{g}}_{r}}\nonumber \\&\quad +\,C\int _{\Sigma _r}\Bigl (\frac{1}{r^2}+\frac{1}{\Omega ^2}\Bigr )\Bigl (|E|^2+|H|^2\Bigr )\mathrm {d}\mu _{{\overline{g}}_{r}} \end{aligned}$$

#### Proof

Since *W* solves the Bianchi equations, *E*, and *H* solve (6.7) — cf. (6.4) — with

$$\begin{aligned} \rho _E= & {} H\wedge k\qquad \sigma _E={\overline{\nabla }}\log \phi \wedge E-\frac{1}{2}k\times H-\widehat{{\mathcal {L}}_nH}\\ \rho _H= & {} -E\wedge k\qquad \sigma _H={\overline{\nabla }}\log \phi \wedge H+\frac{1}{2}k\times E+\widehat{{\mathcal {L}}_nE} \end{aligned}$$

By Lemma 6.1, and the assumptions (${{\varvec{BA:I}},ii,iv-vi}$) we have

$$\begin{aligned}&| \widehat{{\mathcal {L}}_nE}+\frac{1}{2}k\times E |^2 + | \widehat{{\mathcal {L}}_nH}+\frac{1}{2}k\times H |^2\\&\quad \le \frac{1}{\Omega ^2}\bigl [{\tilde{\rho }}^2+{\tilde{\sigma }}^2+|{\tilde{{\underline{\beta }}}}|^2+|{\tilde{\beta }}|^2+|{\tilde{{\underline{\alpha }}}}|^2+|{\tilde{\alpha }}|^2\\&\quad +\,\rho ^2+\sigma ^2+|{\underline{\beta }}_q|^2+|\beta _q|^2+|{\underline{\alpha }}_q|^2+|\alpha _q|^2\bigr ] \end{aligned}$$

Moreover by (6.11), and the assumptions (${{\varvec{BA:I}},ii,iv-vi}$),

$$\begin{aligned} | E\wedge k |^2 + | H \wedge k |^2 \le \frac{C}{\Omega ^2}\bigl [ |{\underline{\alpha }}_q|^2+|{\underline{\beta }}_q|^2+\rho ^2+\sigma ^2+|\beta _q|^2+|\alpha _q|^2\bigr ] \end{aligned}$$

The statement of the Corollary then follows immediately from Lemma 6.2, and (6.9). $\square$

## Sobolev inequalties

We will prove a Sobolev trace inequality on the spacelike hypersurfaces $\Sigma _r$ to relate the bounds on the energy fluxes obtained in Sections 4-5 to $\mathrm {L}^p$ estimates on the spheres.

### Preliminaries

Here $(\Sigma _r,{\overline{g}}_{r})$ are 3-dimensional Riemannian manifolds diffeomorphic to a cylinder ${\mathbb {R}}\times {\mathbb {S}}^2$, and there exists a differentiable function $u:\Sigma _r\rightarrow {\mathbb {R}}$, namely the restriction of the null coordinate *u* to $\Sigma _r$, such that the level sets of $u|_{\Sigma _r}$ are spheres $S_{u,r}$ diffeomorphic to ${\mathbb {S}}^2$, and *u* has no critical points. In fact, in Lemma 2.2 we proven that the metric on $\Sigma _r$ in $(u,\vartheta ^1,\vartheta ^2)$ coordinates, takes the canonical form

7.1 $$\begin{aligned} {\overline{g}}_{r}=q^{-2}\Omega ^2\mathrm {d}u^2+g\!\!\!/_{AB}\mathrm {d}\vartheta ^1\mathrm {d}\vartheta ^2\,. \end{aligned}$$

We also found that the volume form is given by

7.2 $$\begin{aligned} \mathrm {d}\mu _{{\overline{g}}_{r}}=q^{-1}\Omega \,\mathrm {d}u\wedge \mathrm {d}\mu _{g\!\!\!/}=q^{-1}\Omega \sqrt{\det g\!\!\!/}\,\mathrm {d}u \wedge \mathrm {d}\vartheta ^1\wedge \mathrm {d}\vartheta ^2 \end{aligned}$$

The “lapse function” of the foliation of $\Sigma _r$ by spheres $S_u:=S_{u,r}$ is thus given by

7.3 $$\begin{aligned} a=\frac{\Omega }{q} \end{aligned}$$

Recall that in Section 6.2 we introduced *X* as the unit tangent vector to $\Sigma _r$, orthogonal to $S_u$. Thus we have $aX f=\partial _u f$, for any function $f(u,\vartheta ^1,\vartheta ^2)$ on $\Sigma _r$. We denote by $\theta$ the second fundamental form of the surfaces $S_u$ embedded in $\Sigma _r$. Viewed as a tensor on $\Sigma _r$ we can write

7.4 $$\begin{aligned} \theta (Y,Z)=g\!\!\!/({\overline{\nabla }}_{\Pi Y} X,\Pi Z) \end{aligned}$$

where $\Pi$ denotes the projection to the tangent space of $S_u$, and thus

$$\begin{aligned} \theta _{ij}=\nabla _i X_j+X_i\nabla \!\!\!\!/_j \log a \end{aligned}$$

because $${\overline{\nabla }}_X X=-\nabla \!\!\!\!/\log a$$. Therefore

7.5 $$\begin{aligned} {{\,\mathrm{tr}\,}}_{{\overline{g}}_{}}\theta ={\overline{g}}_{}^{ij}\theta _{ij}={\overline{\nabla }}^i X_i={\overline{{{\,\mathrm{div}\,}}}}X\,. \end{aligned}$$

We also have the first variational formula

$$\begin{aligned} \theta _{AB}=\frac{1}{2a}\partial _u \gamma _{AB}\qquad \theta _{AB}=\theta (\partial _{\vartheta ^A},\partial _{\vartheta ^B}) \end{aligned}$$

which gives

$$\begin{aligned} {{\,\mathrm{tr}\,}}\theta = \frac{1}{2 a }\partial _u\log \det ( g\!\!\!/_{AB} ) \end{aligned}$$

Consequently,

7.6 $$\begin{aligned} \frac{\mathrm {d}}{\mathrm {d}u}A(u)=\int _{S_u}a{{\,\mathrm{tr}\,}}\theta \mathrm {d}\mu _{g\!\!\!/} \end{aligned}$$

and more generally, for any function *f*,

7.7 $$\begin{aligned} \frac{\mathrm {d}}{\mathrm {d}u}\int _{S_u} f\mathrm {d}\mu _{g\!\!\!/}=\int _{S_u}a \bigl \{ X f+ f {{\,\mathrm{tr}\,}}\theta \bigr \}\mathrm {d}\mu _{g\!\!\!/} \end{aligned}$$

#### Remark 7.1

In the spherically symmetric setting, $\det g\!\!\!/= r^4$ is constant on $\Sigma _r$, leading to the *vanishing of the mean curvature* ${{\,\mathrm{tr}\,}}\theta =0$. Thus it cannot be expected that in the present setting ${{\,\mathrm{tr}\,}}\theta$ has a sign.

### Isoperimetric Sobolev Inequalities

We can adapt the proof of the following Sobolev inequality of Proposition 3.2.1 in [10]. There the geometric setting is different, the manifold $\Sigma$ being diffeomorphic to ${\mathbb {R}}^3$, and *u* being a radial function whose level sets have *positive* mean curvature. However, as already remarked there, the proof carries over more generally, if the constant is allowed to depend on the mean curvature. Here we require that

7.8 $$\begin{aligned} r|{{\,\mathrm{tr}\,}}\theta |\le C \end{aligned}$$

Since

7.9 $$\begin{aligned} \theta _{AB}=\frac{1}{2}q{\underline{\chi }}-\frac{1}{2}q^{-1}\chi _{AB} \end{aligned}$$

the assumption amounts to 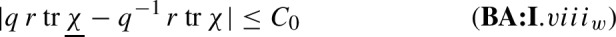


which follows from (${{\varvec{BA:I}}:vii,viii}$); in fact (${{\textbf {BA:I}}.viii}_w$) is *weaker* than (${{\varvec{BA:I}}:viii}$).

Furthermore we will assume that the lapse function of the foliation of $\Sigma _r$ by level sets of *u* is bounded above and below: 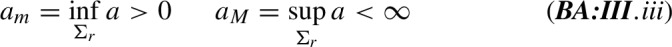 The Sobolev inequality on $\Sigma _r$ stated below relies on the isoperimetric inequalities on the spheres $S_u$, and we thus need some control on the *isoperimetric constant* *I*(*u*) of each sphere $S_u$: 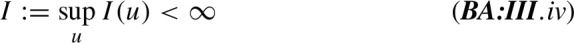


#### Remark 7.2

We note here that in the context of the recovery of the assumptions $({\textbf {BA}})$, the bounds on the isoperimetric constants of $S_{u,v}$ are derived from corresponding assumptions on the initial data — which imply that the isoperimetric constants of the spheres foliating ${\mathcal {C}}\cup \overline{{\mathcal {C}}}$ are bounded — and the validity of the assumptions $({\textbf {BA:I}})$. We refer the reader to Lemma 6.1 in [39], where (${\varvec{BA:III}}{} .iv$) is derived in a simplified setting from (${\varvec{BA:I}}.iii$) using Lemma 5.12 in [39]. The role of the isoperimetric constants of $S_{u,v}$ is also discussed more broadly in Chapter 5.2 in [8].

#### Lemma 7.3

Assume (**BA:III**.iii,iv) hold. Moreover assume (${{\textbf {BA:I}}.viii}_w$) for some $C_0>0$. Then for any $S_u$-tangent tensorfield $\theta$ we have

7.10a $$\begin{aligned} \Bigl (\int _{\Sigma _r} r^6|\theta |^6 \Bigr )^\frac{1}{6}\le C\Bigl (\int _{\Sigma _r}|\theta |^2+r^2|{\overline{\nabla }}\theta |^2 \Bigr )^\frac{1}{2} \end{aligned}$$

7.10b $$\begin{aligned} \sup _u\Bigl (\int _{S_u} r^4|\theta |^4\Bigr )^\frac{1}{4}\le C\Bigl (\int _{\Sigma _r}|\theta |^2+r^2|{\overline{\nabla }}\theta |^2\Bigr )^\frac{1}{2} \end{aligned}$$

where *C* depends on $a_M/a_m$, *I*, and

7.11 $$\begin{aligned} h=\sup _{\Sigma _r}|r{{\,\mathrm{tr}\,}}\theta |<\infty \,. \end{aligned}$$

#### Proof

Recall the isoperimetric inequality on the sphere:

$$\begin{aligned} \int _{S_u}\bigl (\Phi -{\overline{\Phi }}\bigr )^2\mathrm {d}\mu _{g\!\!\!/}\le I(u)\Bigl (\int _{S_u}|\nabla \!\!\!\!/\Phi |\mathrm {d}\mu _{g\!\!\!/}\Bigr )^2 \end{aligned}$$

Apply to $\Phi =|\theta |^3$ to obtain

$$\begin{aligned} \int _{S_u}|\theta |^6\lesssim _ I\Bigl (r^{-2}\int _{S_u}|\theta |^4\Bigr )\Bigl (\int _{S_u}|\theta |^2+r^2|\nabla \!\!\!\!/\theta |^2\Bigr ) \end{aligned}$$

and therefore:

$$\begin{aligned}&\int _{\Sigma }r^6 |\theta |^6=\int \int _{S_u}r^6|\theta |^6a\mathrm {d}\mu _{g\!\!\!/}\mathrm {d}u \lesssim _ I\int \Bigl (r^4\int _{S_u}|\theta |^4\Bigr )\Bigl (\int _{S_u}|\theta |^2+r^2|\nabla \!\!\!\!/\theta |^2\Bigr )a_M(u)\mathrm {d}u\\&\quad \lesssim _I \frac{a_M}{a_m}\sup _u\Bigl (r^4\int _{S_u}|\theta |^4\Bigr )\int _\Sigma |\theta |^2+r^2|\nabla \!\!\!\!/\theta |^2 \end{aligned}$$

Now by the divergence theorem on^46^

$$\begin{aligned} \Sigma ^u=\cup _{l\ge u} S_l \end{aligned}$$

we obtain

$$\begin{aligned} \int _{S_u}r^4|\theta |^4= & {} -\int _{\Sigma ^u}{\overline{{{\,\mathrm{div}\,}}}}\bigl (r^4|\theta |^4 X) \le \int _{\Sigma ^u}r^4|\theta |^4 |{\overline{{{\,\mathrm{div}\,}}}}X | \!+\!4 r^4|\theta |^3|{\overline{\nabla }}_X\theta |+4r^3|X r||\theta |^4\\&\quad \le \int _{\Sigma ^u} r^4 |\theta |^4\bigl ( |{{\,\mathrm{tr}\,}}\theta | +\frac{2}{a}|\overline{a{{\,\mathrm{tr}\,}}\theta } |\bigr )+4r^3|\theta ^3|r|{\overline{\nabla }}_X\theta |\\&\quad \lesssim \frac{a_M}{a_m} \sup _{\Sigma ^u}|r{{\,\mathrm{tr}\,}}\theta | \Bigl (\int _{\Sigma ^u}r^6|\theta |^6\Bigr )^\frac{1}{2}\Bigl (\int _{\Sigma _u}|\theta |^2\Bigr )^\frac{1}{2}\\&\quad +\,4 \Bigl (\int _{\Sigma ^u}r^6|\theta |^6\Bigr )^\frac{1}{2}\Bigl (\int _{\Sigma _u}r^2|{\overline{\nabla }}_X\theta |^2\Bigr )^\frac{1}{2} \end{aligned}$$

where we used (7.5) and

$$\begin{aligned} X r=\frac{r}{2a}\overline{r{{\,\mathrm{tr}\,}}\theta }\,. \end{aligned}$$

Inserting this above yields (7.10a):

$$\begin{aligned} \int _{\Sigma }r^6 |\theta |^6\lesssim _{I,\frac{a_M}{a_m},h} \Bigl (\int _\Sigma |\theta |^2+r^2|{\overline{\nabla }}\theta |^2\Bigr )^3 \end{aligned}$$

Finally inserting this again above then also yields (7.10b):

$$\begin{aligned} \int _{S_u}r^4|\theta |^4\lesssim _{I,\frac{a_M}{a_m},h}\Bigl (\int _\Sigma |\theta |^2+r^2|{\overline{\nabla }}\theta |^2\Bigr )^2 \end{aligned}$$

$$\square $$

See also Chapter 2 in [10] for a broader discussion.

### Applications

Let $\theta$ be any of the null components of the Weyl curvature *W*:

$$\begin{aligned} \theta =\Bigl \{{\underline{\alpha }},{\underline{\beta }},\rho ,\sigma ,\beta ,\alpha \Bigr \} \end{aligned}$$

Note then $\theta$ is a function, or a 1-, or 2-covariant tensorfield on $S_{u}$. Consider the dimensionless $\mathrm {L}^4$ norm^47^ of $\theta$ on a sphere $S_u$:

7.14 $$\begin{aligned} \Vert \!\!\!\!-\theta \Vert \!\!\!\!-_{\mathrm {L}^{4}(S)}:= \Bigl (\frac{1}{4\pi r^2}\int _{S_u}|\theta |^4_{g\!\!\!/}\Bigr )^\frac{1}{4} \end{aligned}$$

Let us now assume the validity of all “bootstrap assumptions” (***BA:I-III***). Then by the Sobolev inequality of Lemma 7.3 above

7.15 $$\begin{aligned} \Vert \!\!\!\!-\theta \Vert \!\!\!\!-_{\mathrm {L}^{4}(S)}\lesssim \frac{1}{r^\frac{3}{2}}\Bigl (\int _{\Sigma _r}|\theta |^2+r^2|{\overline{\nabla }}\theta |^2\Bigr )^\frac{1}{2} \end{aligned}$$

Moreover by Corollary 6.5 in view of (${\varvec{BA:III}}.i$)

7.12 $$\begin{aligned}&\int _{\Sigma _r}r^2|{\overline{\nabla }}\theta |^2 \mathrm {d}\mu _{{\overline{g}}_{r}}\lesssim \int _{\Sigma _r}|{\tilde{{\underline{\alpha }}}}|^2+|{\tilde{{\underline{\beta }}}}|^2+{\tilde{\rho }}^2+{\tilde{\sigma }}^2+|{\tilde{\beta }}|^2+|{\tilde{\alpha }}|^2\mathrm {d}\mu _{{\overline{g}}_{r}}\nonumber \\&\quad +\,\int _{\Sigma _r}|{\underline{\alpha }}|^2+|{\underline{\beta }}|^2+\rho ^2+\sigma ^2+|\beta |^2+|\alpha |^2\mathrm {d}\mu _{{\overline{g}}_{r}} \end{aligned}$$

Finally by the main results of Proposition 5.19

$$\begin{aligned}&\int _{\Sigma _r}|{\tilde{{\underline{\alpha }}}}|^2+|{\tilde{{\underline{\beta }}}}|^2+{\tilde{\rho }}^2+{\tilde{\sigma }}^2+|{\tilde{\beta }}|^2+|{\tilde{\alpha }}|^2\mathrm {d}\mu _{{\overline{g}}_{r}}\lesssim r^3\int _{\Sigma _r}{}^*P^q[\tilde{{\mathcal {L}}}_{N}W]\lesssim \frac{1}{r^{3}}\\&\int _{\Sigma _r}|{\underline{\alpha }}|^2+|{\underline{\beta }}|^2+\rho ^2+\sigma ^2+|\beta |^2+|\alpha |^2\mathrm {d}\mu _{{\overline{g}}_{r}}\lesssim r^3\int _{\Sigma _r}{}^*P^q[W]\lesssim \frac{1}{r^{3}} \end{aligned}$$

which implies

7.16 $$\begin{aligned} \Vert \!\!\!\!-\theta \Vert \!\!\!\!-_{\mathrm {L}^{4}(S)}\lesssim \frac{1}{r^{3}}\,. \end{aligned}$$

This concludes the proof of the theorem.

## Conformal geometry

In this appendix we recall a relationship between the Weyl curvature of the ambient spacetime $({\mathcal {M}},g)$ and the intrinsic conformal geometry of $\Sigma _r$. More precisely we recall that the *Bach tensor* — which provides a local characterisation of conformal flatness — of $\Sigma _r$ is related to the magnetic part of the Weyl curvature. The fact that in our main theorem *all components* of the Weyl curvature are on equal footing — in particular that the magnetic part decays at the *same rate* as the electric part indicates that in general $\Sigma _r$ is *not* conformally flat.^48^

Recall the *Bach tensor* of a 3-dimensional Riemannian manifold $(\Sigma ,{\overline{g}}_{})$:

A.1 $$\begin{aligned} B={{\,\mathrm{curl}\,}}{\hat{S}} \end{aligned}$$

here ${\hat{S}}$ is the trace-free part of the Ricci curvature *S* of $\Sigma$,

A.2 $$\begin{aligned} {\hat{S}}_{ij}=S_{ij}-\frac{1}{3}{{\overline{g}}_{}}_{ij}{{\,\mathrm{tr}\,}}S \end{aligned}$$

and ${{\,\mathrm{curl}\,}}$ denotes the *symmetric curl*.^49^

*The fundamental property of* *B* *is that its vanishing locally identifies conformal flatness.*

Here we will prove a formula that relates *B* to the magnetic part of the ambient Weyl curvature *W* relative to $\Sigma _r$:

### Proposition A.1

Let *B* be the Bach tensor of $(\Sigma _r,{\overline{g}}_{r})$, and $(\Sigma _r,{\overline{g}}_{r},k)$ be a hypersurface in (*M*, *g*), which satisfies the Einstein equations ${{\,\mathrm{Ric}\,}}(g)=\Lambda g$. Then *B* is given in terms of *H* by

A.3 $$\begin{aligned} B=-\widehat{{\mathcal {L}}_nH}+{\overline{\nabla }}\log \phi \wedge E -\frac{1}{3}{\overline{\nabla }}{{\,\mathrm{tr}\,}}k\wedge {\hat{k}} - \frac{1}{2} {\hat{k}}\wedge {\overline{\nabla }}{\hat{k}} - {\hat{k}}\times H \end{aligned}$$

We recall the Gauss-Codazzi equations of the embedding of $\Sigma _r\subset {\mathcal {M}}$, and express the curvature that appears on the right hand sides of (2.70a) and (2.71) in terms of the electric and magnetic parts *E*, *H* of the ambient Weyl curvature *W*:

A.4 $$\begin{aligned}&{\overline{\nabla }}_i k_{jm}-{\overline{\nabla }}_j k_{im}=R_{m0ij}=-\epsilon _{ij}^{k}H_{km} \end{aligned}$$

A.5 $$\begin{aligned}&{\overline{{{\,\mathrm{Ric}\,}}}}_{ij}+{{\,\mathrm{tr}\,}}k k_{ij}-k_{i}^{m}k_{mj}={{\,\mathrm{Ric}\,}}_{ij}+R_{0i0j}=E_{ij}+\frac{2}{3}\Lambda g_{ij} \end{aligned}$$

A.6 $$\begin{aligned}&{\overline{R}}+({{\,\mathrm{tr}\,}}k)^2-|k|^2=2\Lambda \end{aligned}$$

We can now relate the Bach tensor *B* of $\Sigma _r$ to the Weyl curvature *W* of the ambient spacetime; here we always denote

$$\begin{aligned}S:=S_r:={\overline{{{\,\mathrm{Ric}\,}}}}:={{\,\mathrm{Ric}\,}}({\overline{g}}_{r})\end{aligned}$$

### Lemma A.2

The traceless part of the Ricci curvature of $\Sigma _r\subset {\mathcal {M}}$ is given by

A.7 $$\begin{aligned} {\hat{S}}=E+\frac{1}{2}{\hat{k}}\times {\hat{k}}-\frac{1}{3}{{\,\mathrm{tr}\,}}k {\hat{k}} \end{aligned}$$

### Proof

We have already seen in (2.71) that the Codazzi equations read

A.8 $$\begin{aligned} (S_r)_{ij}+{{\,\mathrm{tr}\,}}k k_{ij}-k_{i}^{m}k_{mj}={{\,\mathrm{Ric}\,}}_{ij}(g)+R_{0i0j}=\Lambda ({\overline{g}}_{r})_{ij}+R_{0i0j} \end{aligned}$$

and

A.9 $$\begin{aligned} {{\,\mathrm{tr}\,}}_{{\overline{g}}_{r}} S=-({{\,\mathrm{tr}\,}}k)^2+|k|^2+2\Lambda \end{aligned}$$

by the Einstein equations, and thus

A.10 $$\begin{aligned} {\hat{S}}_{ij}={\hat{k}}_i^{m}{\hat{k}}_{mj}-\frac{1}{3}|{\hat{k}}|^2({\overline{g}}_{r})_{ij}-\frac{1}{3}{{\,\mathrm{tr}\,}}k {\hat{k}}_{ij}+\frac{1}{3}\Lambda ({\overline{g}}_{r})_{ij}+R_{0i0j} \end{aligned}$$

We have also seen in (6.2) that the electric part of the Weyl curvature relates to the relevant component of the Riemann curvature in view of (2.5) as follows:

A.11 $$\begin{aligned} E_{ij}=W_{0i0j}=R_{0i0j}+\frac{\Lambda }{3}({\overline{g}}_{r})_{ij} \end{aligned}$$

Therefore

A.12 $$\begin{aligned} {\hat{S}}_{ij}=E_{ij}+{\hat{k}}_i^{m}{\hat{k}}_{mj}-\frac{1}{3}|{\hat{k}}|^2({\overline{g}}_{r})_{ij}-\frac{1}{3}{{\,\mathrm{tr}\,}}k {\hat{k}}_{ij} \end{aligned}$$

which yields the formula of the Lemma, in view of the fact

A.13 $$\begin{aligned} ({\hat{k}}\times {\hat{k}})_{ij} = 2{\hat{k}}_i^{m}{\hat{k}}_{mj}-\frac{2}{3}{\hat{k}}\cdot {\hat{k}}g_{ij} \end{aligned}$$

$$\square $$

For the curl of ${\hat{S}}$ we then have

A.14 $$\begin{aligned} {{\,\mathrm{curl}\,}}{\hat{S}}={{\,\mathrm{curl}\,}}E+\frac{1}{2}{{\,\mathrm{curl}\,}}{\hat{k}}\times {\hat{k}}-\frac{1}{6}{\overline{\nabla }}{{\,\mathrm{tr}\,}}k\wedge {\hat{k}}-\frac{1}{3}{{\,\mathrm{tr}\,}}k {{\,\mathrm{curl}\,}}{\hat{k}} \end{aligned}$$

and it remains to calculate the curl of

A.15 $$\begin{aligned} \bigl ({\hat{k}}\times {\hat{k}}\bigr )_{ij}=\epsilon _i^{ab}\epsilon _j^{cd}{\hat{k}}_{ac}{\hat{k}}_{bd}+\frac{1}{3}|{\hat{k}}|^2 g_{ij} \end{aligned}$$

Since

A.16 $$\begin{aligned} \epsilon _i^{ab}\epsilon _b^{mn}=\delta ^m_i\delta ^{an}-\delta ^n_i\delta ^{am} \end{aligned}$$

we obtain

A.17 $$\begin{aligned} \begin{aligned} ({{\,\mathrm{curl}\,}}{\hat{k}}\times {\hat{k}})_{ij}&=\epsilon _i^{cd}\nabla ^a({\hat{k}}_{ad}{\hat{k}}_{jc}\bigr )+\epsilon _j^{cd}\nabla ^a({\hat{k}}_{ad}{\hat{k}}_{ic}\bigr )\\&=-\bigl ({{\,\mathrm{div}\,}}{\hat{k}}\wedge {\hat{k}}\bigr )_{ij}+\epsilon _i^{cd}{\hat{k}}_{ad}\nabla ^a{\hat{k}}_{jc}+\epsilon _j^{cd}{\hat{k}}_{ad}\nabla ^a{\hat{k}}_{ic} \end{aligned} \end{aligned}$$

Note that the Codazzi equation (A.4) implies that

A.18a $$\begin{aligned}&\epsilon _{ab}^{j}{\overline{\nabla }}_i k_{jm}=\epsilon _{ab}^{j}{\overline{\nabla }}_j k_{im}-\delta _{ai}H_{bm}+\delta _{bi}H_{am} \end{aligned}$$

A.18b $$\begin{aligned}&({{\,\mathrm{div}\,}}k)_j-{\overline{\nabla }}_j{{\,\mathrm{tr}\,}}k=0 \end{aligned}$$

or alternatively for the trace-free parts

A.19a $$\begin{aligned}&\epsilon _{ab}^{j}{\overline{\nabla }}_i {\hat{k}}_{jm}=-\frac{1}{3}\epsilon _{amb}{\overline{\nabla }}_i {{\,\mathrm{tr}\,}}k+\epsilon _{ab}^{j}{\overline{\nabla }}_j {\hat{k}}_{im}\nonumber \\&\quad +\,\frac{1}{3}\epsilon _{ab}^{j}g_{im}{\overline{\nabla }}_j {{\,\mathrm{tr}\,}}k-\delta _{ai}H_{bm}+\delta _{bi}H_{am} \end{aligned}$$

A.19b $$\begin{aligned}&({{\,\mathrm{div}\,}}{\hat{k}})_j=\frac{2}{3}{\overline{\nabla }}_j{{\,\mathrm{tr}\,}}k \end{aligned}$$

which then implies that

A.20 $$\begin{aligned}&\epsilon _i^{cd}{\hat{k}}_{ad}{\overline{\nabla }}^a{\hat{k}}_{jc}+\epsilon _j^{cd}{\hat{k}}_{ad}{\overline{\nabla }}^a{\hat{k}}_{ic}=\nonumber \\&\quad =\epsilon _{i}^{cd}{\hat{k}}_{ad}{\overline{\nabla }}_c {\hat{k}}^a_{j}+\epsilon _{j}^{cd}{\hat{k}}_{ad}{\overline{\nabla }}_c {\hat{k}}^a_{i}\nonumber \\&\quad +\,\frac{1}{3}\epsilon _{i}^{cd}{\hat{k}}_{jd}{\overline{\nabla }}_c {{\,\mathrm{tr}\,}}k+\frac{1}{3}\epsilon _{j}^{cd}{\hat{k}}_{id}{\overline{\nabla }}_c {{\,\mathrm{tr}\,}}k-{\hat{k}}_{id}H_{j}^d-{\hat{k}}_{jd}H_{i}^d\nonumber \\&\quad =- \bigl ({\hat{k}}\wedge {\overline{\nabla }}{\hat{k}}\bigr )_{ij}+\frac{2}{3}{\hat{k}}\cdot H g_{ij}+\frac{1}{3}\bigl ({\overline{\nabla }}{{\,\mathrm{tr}\,}}k\wedge {\hat{k}}\bigr )_{ij}\nonumber \\&\quad -\,({\hat{k}}\times H)_{ij}-\frac{2}{3}({\hat{k}}\cdot H) g_{ij} \end{aligned}$$

where we introduced the notation:

A.21 $$\begin{aligned} ({\hat{k}}\wedge {\overline{\nabla }}{\hat{k}})_{ij}=\epsilon _{i}^{cd}{\hat{k}}_{ca}{\overline{\nabla }}_d {\hat{k}}^a_{j}+\epsilon _{j}^{cd}{\hat{k}}_{ca}{\overline{\nabla }}_d {\hat{k}}^a_{i}-\frac{2}{3}\Bigl (\epsilon ^{mcd}{\hat{k}}_{ca}{\overline{\nabla }}_d {\hat{k}}^a_{m}\Bigr )g_{ij} \end{aligned}$$

and observed that by the Codazzi equations

A.22 $$\begin{aligned}&\epsilon ^{jcd}{\hat{k}}_{ca}{\overline{\nabla }}_d {\hat{k}}^a_{j}=-\epsilon ^{jcd}{\hat{k}}_{ca}{\overline{\nabla }}_d {\hat{k}}^a_{j}+\epsilon ^{jcd}{\hat{k}}_{ca}\epsilon _{jd}^{m}H_{m}^{a}\nonumber \\&\quad \epsilon ^{jcd}{\hat{k}}_{ca}{\overline{\nabla }}_d {\hat{k}}^a_{j} = -\frac{2}{2}\delta ^{cm}{\hat{k}}_{ca}H_m^{a}=-{\hat{k}}\cdot H \end{aligned}$$

In conclusion,

A.23 $$\begin{aligned} {{\,\mathrm{curl}\,}}{\hat{k}}\times {\hat{k}} = -\frac{1}{3} {\overline{\nabla }}{{\,\mathrm{tr}\,}}k\wedge {\hat{k}} - {\hat{k}}\wedge {\overline{\nabla }}{\hat{k}} -{\hat{k}}\times H \end{aligned}$$

and we have proven that

A.24 $$\begin{aligned} \begin{aligned} {{\,\mathrm{curl}\,}}{\hat{S}}&={{\,\mathrm{curl}\,}}E+\frac{1}{2}{{\,\mathrm{curl}\,}}{\hat{k}}\times {\hat{k}}-\frac{1}{6}{\overline{\nabla }}{{\,\mathrm{tr}\,}}k\wedge {\hat{k}}-\frac{1}{3}{{\,\mathrm{tr}\,}}k {{\,\mathrm{curl}\,}}{\hat{k}}\\&={{\,\mathrm{curl}\,}}E -\frac{1}{3}{\overline{\nabla }}{{\,\mathrm{tr}\,}}k\wedge {\hat{k}} - \frac{1}{2} {\hat{k}}\wedge {\overline{\nabla }}{\hat{k}} - \frac{1}{2} {\hat{k}}\times H +\frac{1}{3}{{\,\mathrm{tr}\,}}k H \end{aligned} \end{aligned}$$

because again by (A.4),

A.25 $$\begin{aligned} {{\,\mathrm{curl}\,}}k=-H \end{aligned}$$

The proof of Prop. A.1 is now immediate in view of the electromagnetic decomposition of the Bianchi equations (6.4), in particular (6.4b),

$$\begin{aligned} {{\,\mathrm{curl}\,}}E=-\widehat{{\mathcal {L}}_nH}+{\overline{\nabla }}\log \phi \wedge E-\frac{1}{2}k\times H \end{aligned}$$

Indeed, it follows from (A.24) that

$$\begin{aligned} B=-\widehat{{\mathcal {L}}_nH}+{\overline{\nabla }}\log \phi \wedge E -\frac{1}{3}{\overline{\nabla }}{{\,\mathrm{tr}\,}}k\wedge {\hat{k}} - \frac{1}{2} {\hat{k}}\wedge {\overline{\nabla }}{\hat{k}} - {\hat{k}}\times H \,. \end{aligned}$$

## Assumptions

We list here in three tables the assumptions on the metric and connection coefficients that are made in this paper under the label that they are referred to along with the page numbers where they are first introduced and further discussed in the text. Here

B.1 $$\begin{aligned} q=\sqrt{\frac{\overline{\Omega {{\,\mathrm{tr}\,}}\chi }}{\overline{\Omega {{\,\mathrm{tr}\,}}{\underline{\chi }}}}} \end{aligned}$$

and $C_0>0$ is a fixed constant.

## Footnotes

1 The equations (1.1) in its more general form including sources of matter can be thought of as a general relativistic version of the classical analogue $\triangle \psi -\Lambda \psi =4\pi \rho$, namely a modification of Newton’s law which Einstein considered to model homogeneous mass densities $\rho$ in space [18].

2 Neither was this solution discovered in this form, nor was it immediately understood that this “universe” is expanding. This was realized only later by Lemaître [30]. For a historical discussion of the confusions and controversies surrounding its discovery see e.g. [31].

3 In particular our choice of gauge is informed by the behavior of solutions to the eikonal equation on de Sitter spacetime; see [39].

4 Here we simply mean that given any two time-like geodesics $\Gamma _1$, $\Gamma _2$ in ${\mathcal {R}}^+$ (with different “end points” on ${\mathcal {I}}^+$) parametrized by arc length, we can find proper times $s_1$, and $s_2$ such that the causal futures of $\Gamma _1(s_1)$ and $\Gamma _2(s_2)$ have *empty* intersection.

5 Here ${\mathcal {I}}^+$ can be identified with the endpoints all outgoing null geodesics from ${\mathcal {S}}$ along which $r\rightarrow \infty$, and is thus still referred to as *null infinity*. It being spacelike (as a surface in a suitably extended space-time) marks an important difference to the asymptotically flat setting, and is prototypical for $\Lambda >0$.

6 It is expected that the analysis in [12] (adapted to the $\Lambda >0$ setting) can be combined with the results of [27] to prove the $C^0$-stability of the interior up to the Cauchy horizon, and to *disprove unconditionally* the $C^0$-formulation of strong cosmic censorship. However it is an open question to understand in which sense strong cosmic censorship may hold in this setting; see Section 1.6 in [12].

7 Here ${\mathcal {S}}$ is of course not defined with the help of ${\mathcal {I}}^+$, but instead found dynamically as the stationary region of the Kerr de Sitter geometry that the solution asymptotes to, and the solution is controlled in a slightly larger domain, as indicated in Fig. 3.

8 Strictly speaking [27] does not construct the cosmological horizon as a null hypersurface, but this is a minor point given that one expects to obtain ${\mathcal {C}}$ in their setting by an application of the stable manifold theorem. In any case, in this paper — in particular in the main theorem of Section 1.3 — we actually do *not* use the exponential decay along the cosmological horizons. While the results in this paper hold under broader assumptions, one could impose these stronger assumptions to derive the behaviour of all geometric quantities not only in *r*(*u*, *v*) but also track the behaviour as a function of “retarded time” *u*; see Section 1.1 for definitions. The detailed analysis of the characteristic constraint equations under the exponential decay assumption, and the investigation of finer asymptotics towards $\iota ^+$ are not included in this paper; see also Remark 1.5 below.

9 As we shall see the geometry *is* expected to be asymptotically de Sitter *locally* in the past of any time-like geodesic, however it cannot be expected to agree *globally*. Indeed the stability results in Appendix C of [27] for the static model of de Sitter space are of a local nature, in the sense that they apply to the past of a point $p\in {\mathbb {S}}^3$ on the future boundary. If the cosmological region of the Kerr de Sitter solution is stable then we could choose a point $p\in {\mathcal {I}}^+$, and find than in a suitable small neighborhood of *p* as depicted in Figure 4 the past of *p* is a perturbation of the static region of de Sitter spacetime, to which Theorem C.4 in [27] applies. However, convergence of the metric to de Sitter as the point *p* on the future boundary is approached can only be achieved *in a gauge that depends on* *p*. In fact, their proof proceeds in by an iteration scheme which eliminates growing modes of the linearised equations *by a suitable gauge choice which depends on the solution in the past of* *p*. This behaviour of the solutions at late times observed *locally* also runs under the name of “cosmic no-hair”, and has been proven *in spherical symmetry* in [11].

10 In general a spacelike hypersurface is conformally flat if the Bach tensor *B* vanishes identically, where *B* is related to the magnetic part of the ambient Weyl curvature *W*; see Appendix A. Here future null infinity ${\mathcal {I}}^+$ is a not an embedded hypersurface but rather the boundary of a conformally changed spacetime, and the statement that ${\mathcal {I}}^+$ is conformally flat refers to the vanishing of the Bach tensor of the *rescaled* Weyl curvature $r^3 W$. Thus the conformal flatness of ${\mathcal {I}}^+$ is related to the vanishing of certain components of the *rescaled* Weyl curvature, and our setting is compatible with all components of $r^3 W$ to be supported, and ${\mathcal {I}}^+$ *not* to be conformally flat.

11 The “functional degrees of freedom at infinity” referred to here already appear in the context of Friedrich’s proof of the stability of de Sitter spacetime [20], and are precisely captured by the free data for the “conformal constraint equations”. In Section 2.3 of [21] it is pointed out that Friedrich’s conformal constraint equations reduce to ${\tilde{\nabla }}\cdot D=0$, where *D* is the electric part of the *rescaled* conformal Weyl curvature, and ${\tilde{\nabla }}$ is the connection associated the intrinsic metric on $({\mathcal {I}}^+,h)$; see also Section 6 for the definition of electromagnetic decomposition. The statement that ${\mathcal {I}}^+$ is not intrinsically locally conformally flat is here naturally related to the non-vanishing of the Cotton tensor of the metric *h*; cf. the discussion of the related Bach tensor in Appendix A..

12 de Sitter space is in fact *conformally flat*. The passing from *R* to *W* can thus be thought of as a renormalisation of the curvature by its de Sitter values. Note also that for $\Lambda =0$ the Weyl and Riemann curvature coincide, and thus play a notably different role only in the cosmological setting.

13 Connection coefficients are on the level of *one* derivative of the metric. Alternatively these assumptions can be thought of as conditions on the geometric properties of a chosen *foliation*, or as conditions on the deformation tensors of a number of relevant vectorfields.

14 In a simplified setting this is precisely what is achieved in [39]. Therein a global double null foliation of de Sitter spacetime — namely under the assumption that the Weyl curvature vanishes identically — is constructed by a suitable choice of spheres on a bifurcate null hypersurface.

15 Characteristic initial data will in particular be prescribed such that *along* the cosmological horizons the geometry converges exponentially fast to the geometry induced by Kerr de Sitter. The detailed geometric setup is not discussed in this paper. For a general discussion of *characteristic* initial data we refer to Chapter 2 in [8].

16 Interestingly, in a different context, namely in the study of relativistic compressible fluids, a similar issue appears for the characterisation of the “singular hypersurface” in the formation of shocks; see Chapter 15 in [7].

17 This gauge — and its very general geometric formulation suitable for the study of the Einstein equations outside spherical symmetry — has been introduced by Christodoulou [6] and used notably in his [8], whose conventions we follow closely in this paper.

18 Essentially, any assumption that requires the smallness of the deviation of a quantity $f(u,v,\vartheta ^1,\vartheta ^2)$ from its average ${\overline{f}}(u,v)$ on the sphere $S_{u,v}$ cannot be satisfied if the area radius of $S_{u,v}$ diverges while a subset of points $q\in S_{u,v}$ remain in the spacetime.

19 The coordinates $(\vartheta ^1,\vartheta ^2)$ are chosen arbitrarily on a domain of $S={\mathcal {C}}\cap \overline{{\mathcal {C}}}$, and then transported first along the null geodesics generating $\overline{{\mathcal {C}}}$, and then those of ${\underline{C}}_v$.

20 Here we choose for simplicity coordinates on $\Sigma _r$ which are transported along the normal geodesics, which justifies the absence of a shift term.

21 In Section 4 we will consider the “energy flux” of the Weyl curvature through $\Sigma _r$, and the form (1.24) is particularly well adapted to the application of the *co-area formula* for integration on domains foliated by $\Sigma _r$. Also the Sobolev inequality of Section 7 is applied on the manifold $\Sigma _r$.

22 In Schwarzschild de Sitter there is really only *one* non-vanishing component of the Weyl curvature. In this result all components of the Weyl curvature are on an equal footing, and at present there is no indication of any “peeling” behavior. This is consistent with the expectations formulated in [3], and we will revisit this point in Section 6 in the context of the electro-magnetic decomposition of the Weyl curvature.

23 This is an insight gained in [4], which has provided a significant simplification of the original proof of the non-linear stability of Minkowski space in [10]. It eliminates in particular the need to work with *second* derivatives of the curvature, but comes of course at the cost of less refined asymptotics; see [4] and discussion therein. Further to the discussion of the conformal constraint equations in footnote 10, we remark that the present results are at the level of $D\in \mathrm {H^1({\mathcal {I}}^+)}$. Note however that the Theorem holds under minimal regularity assumptions on $\Omega$, which plays the role of the conformal factor. In view of Sections 2.2 & 7.3 in [39] we expect to close the estimates with $\Omega$, $D\Omega$, ${\underline{D}}\Omega$ merely in $\mathrm {W}_2^4(S_{u,v})$.

24 The assumption that *both* null expansions are positive fails in ${\mathcal {S}}$, where ${{\,\mathrm{tr}\,}}{\underline{\chi }}<0$. Similarly in the study of *asymptotically flat* black hole exteriors, with $\Lambda =0$, one has ${{\,\mathrm{tr}\,}}{\underline{\chi }}<0$. In these settings the construction of *positive currents* is much more subtle, and in particular sensitive to the presence of “trapped” null geodesics; see [14] and references therein.

25 Much like the redshift observed in black hole spacetimes, the effect is due to the presence of horizons: An observer *A* who stays away from a black hole perceives a signal sent from an observer *B* who crosses the *event horizon* as redshifted, because *B* leaves the past of *A* in finite proper time; see e.g. [13]. In the cosmological region, where all observers are drifting away from each other (due to the *expansion* of space), each observer has its *own* cosmological horizon, which all other observers (in his past) cross in finite proper time. This effect is already present in the de Sitter cosmology, where each time-like geodesic has a cosmological horizon with positive surface gravity; see [38, 39].

26 This is an idea due to Dafermos and Rodnianski that already appeared in the study of the linear wave equation on Schwarzschild spacetimes, related to the redshift effect on the event horizon; see [13]. Similarly to Theorem 3.2 therein one might also consider *multiple* commutations by *N*, with the aim of obtaining ${\overline{\nabla }}^k W\in \mathrm {L}^2(\Sigma _r)$, but in view of the complexity that we encounter already at the level of *one* commutation — see Section 5 — we do not expect that a “higher order global redshift effect” can be established easily. Note that a *second* commutation by *N*, and a suitable version of (1.45) for ${\tilde{{\mathcal {L}}}}_N^2 W$, would give pointwise estimates for $W\in \mathrm {L}^\infty$.

27 The cancellations occur as a result of a delicate choice of the commutation vectorfield *N*, rather than particularly strict geometric assumptions. In other words, if the spacetime satisfies the assumptions (**BA:I,II**) introduced below (see also Appendix B) then a commutator can be construced as in Section 5, and thus the cancellations are stable within this class of spacetimes.

28 In problems where this circle of ideas — the treatment of Bianchi equations using energies constructed from the Bel-Robinson tensor — has been applied, these terms are usually treated as “error terms”. Notably for the “stability of Minkowski space” they have only been written out schematically in Chapter 8 in [10]. Although they are also treated as error terms in proof of the “formation of black holes” in [8], their precise nature, and “scaling”, is much more important therein, and we take great advantage in this paper of the fact that at least in special cases precise algebraic expressions for the “divergence terms” have already been provided in Chapter 12-14 in [8].

29 The analysis of these systems has been developed in some generality in Chapter 2-4 of [10].

30 This in turn is motivated by the insight that some of the main difficulties in obtaining a global double null foliation with the desired properties already occur in de Sitter space, and are less related to the specific decay properties of the Weyl curvature.

31 This restriction is of some interest for the full problem, too: While in the formulation of page 5 non-trivial data is posed on both cosmological horizons (and this is certainly the relevant setting for the non-linear stability problem) the physically relevant setting — namely the specific setting in which physical meaning can be ascribed to the asymptotic quantities — is the special case when a *no incoming radiation condition* is imposed on $\overline{{\mathcal {C}}}^+$; see also [1].

32 We already see that the Weyl curvature on the right hand side is *not* the slowest decaying term.

33 While the result is very relevant for this paper, the ideas of their proof are entirely different: In the stationary region ${\mathcal {S}}$ a “resonance expansion” is available for solutions to the linearized equations, and the authors succeed in relating all growing modes to pure gauge solutions; cf. Section 4 in [27].

34 The author is not aware of paper where this procedure is carried out in detail, but one would expect that the outlined truncation/gluing steps can be achieved using known techniques; see e.g. [5].

35 Also [33] stops short of relating the “asymptotic profiles” to the details of the initial data, which is largely an open problem; see also [32].

36 Albeit in [36] a constant mean curvature gauge is used “to synchronize the singularity”, while in this work we rely on a double null gauge to “parametrize null infinity”.

37 Note the frame is *not* “Fermi transported”.

38 For a broader discussion of *non-*spherically symmetric foliations in this setting see [39].

39 We caution that this frame is not regular on the horizon, so the values of ${{\,\mathrm{tr}\,}}\chi$, and ${{\,\mathrm{tr}\,}}\chi$ have no meaning on ${\mathcal {C}}\cup \overline{{\mathcal {C}}}$.

40 One reason to expect that this vectorfield produces the correct lower order terms can already be inferred without computing the terms in (5.3): The modified Lie derivative is an expression of the form (5.5). Given control on $\tilde{{\mathcal {L}}}_{N}W$, we only obtain control on all tangential derivatives by the elliptic estimate of Section 6, if the lower order term in the expression for $\tilde{{\mathcal {L}}}_{N}W$ match precisely the lower order terms in the “Maxwell equations” (6.4) of the electro-magnetic decomposition on $\Sigma _r$, cf. Lemma 6.1. This gives a condition on ${{\,\mathrm{tr}\,}}{}^{(N)}\pi$ which can motivate the rescaling by $\Omega ^2$ in (5.4).

41 In this paper we denote the Weyl current defined in (5.10b) by *d*, and not *q* as in [8], because the notation *q* is already used in Lemma 2.1.

42 Note specifically that this Lemma does *not* require the stricter version (${{\varvec{BA:I}}}.iv^{\prime },vi^{\prime }$) to hold.

43 The appearance of such *borderline error integrals* may be familiar already from [8]. Indeed terms of precisely this form play a prominent role in Chapter 14.2, see e.g. (14.32, 14.34, 14.37) in [8], which also require more precise estimates.

44 One may think of the resulting fields as electric and magnetic parts in the frame of reference of an observer with 4-velocity *n*, the normal to $\Sigma$.

45 This formula differs from (7.2.2h) in [10] because $\Sigma _r$ is *not* maximal, and the frame *not* Fermi transported.

46 We can think of the cylinder $\Sigma$ being divided in two components by $S_u$, $\Sigma _u$ being one component, a 3-dimensional manifold with boundary $S_u$.

47 These norms will play a prominent role in the “recovery” of the assumptions (***BA***), see already Section 7 in [39]. Here they are introduced for convenience, but they also provide the correct “scaling” in comparison to the $\mathrm {L}^\infty (S_{u,v})$ norm, in the sense that under suitable assumptions the following Sobolev inequality holds for any *S*-tangent tensorfield $\xi$:

7.13 $$\begin{aligned} \Vert \xi \Vert _{L^\infty (S)}\lesssim \Vert \!\!\!\!-\xi \Vert \!\!\!\!-_{\mathrm {L}^{4}(S)}+\Vert \!\!\!\!-r\nabla \!\!\!\!/\xi \Vert \!\!\!\!-_{\mathrm {L}^{4}(S)}\end{aligned}$$

In particular, if the right hand side decays as a function of *r*(*S*), then $\xi$ decays in $\mathrm {L}^\infty (S)$ at the same rate. We refer the reader to Section 6 of [39] for the proof of this inequality in the current setting, and to Chapter 5 in [8] for Sobolev inequalities on spheres more generally.

## References

[1] Ashtekar, A., Bonga, B., Kesavan, A.: Asymptotics with a positive cosmological constant: I. Basic framework, Classical Quantum Gravity **32**(2):025004, 41 (2015)

[2] Ashtekar, A., Bonga, B., Kesavan, A.: Asymptotics with a positive cosmological constant. II. Linear fields on de Sitter spacetime, Phys. Rev. D **92**(4), 044011, 14 (2015)

[3] Ashtekar A, Bonga B, Kesavan A (2016). Gravitational waves from isolated systems: Surprising consequences of a positive cosmological constant. Phys. Rev. Lett..

[4] Bieri L, Zipser N (2009). Extensions of the Stability Theorem of the Minkowski Space in General Relativity, AMS/IP Studies in Advanced Mathematics.

[5] Choquet-Bruhat Y, Isenberg J, Pollack D (2007). The constraint equations for the Einstein-scalar field system on compact manifolds. Class. Quant. Gravity.

[6] Christodoulou, D.: Notes on the geometry of null hypersurfaces (1991)

[7] Christodoulou, D.: The formation of shocks in 3-dimensional fluids, EMS Monographs in Mathematics. European Mathematical Society (EMS), Zürich (2007)

[8] Christodoulou, D.: The formation of black holes in general relativity, EMS Monographs in Mathematics. European Mathematical Society (EMS), Zürich (2009)

[9] Christodoulou D, Klainerman S (1990). Asymptotic properties of linear field equations in Minkowski space. Commun. Pure Appl. Math..

[10] Christodoulou D, Klainerman S (1993). The Global Nonlinear Stability of the Minkowski Space, Princeton Mathematical Series.

[11] Costa JL, Natário J, Oliveira P (2019). Cosmic no-hair in spherically symmetric black hole spacetimes. Ann. Henri Poincaré.

[12] Dafermos, M., Luk, J.: The interior of dynamical vacuum black holes i: The c0-stability of the kerr cauchy horizon, arXiv:1710.01722 [gr-qc] (2017)

[13] Dafermos, M., Rodnianski, I.: Lectures on Black Holes and Linear Waves, Evolution Equations, Clay Math. Proc, vol. 17, pp. 97–205. Amer. Math. Soc., Providence (2013)

[14] Dafermos, M., Holzegel, G., Rodnianski, I.: The linear stability of the Schwarzschild solution to gravitational perturbations, arXiv:1601.06467, (2016)

[15] de Sitter W (1917). On Einstein’s theory of gravitation and its astronomical consequences. Mon. Not. R. Astron. Soc..

[16] Dyatlov S (2011). Exponential energy decay for Kerr-de Sitter black holes beyond event horizons. Math. Res. Lett..

[17] Dyatlov S (2011). Quasi-normal modes and exponential energy decay for the Kerr-de Sitter black hole. Commun. Math. Phys..

[18] Einstein, A.: Kosmologische Betrachtungen zur allgemeinen Relativitätstheorie, Sitzungsberichte der Preußischen Akad. d. Wissenschaften, 142–152 (1917), http://einsteinpapers.press.princeton.edu/vol6-doc/568

[19] Fefferman, C., Robin Graham, C.: Conformal invariants, no. Numéro Hors Série, 1985, The mathematical heritage of Élie Cartan (Lyon, 1984), pp. 95–116

[20] Friedrich H (1986). On the existence of $$n$$-geodesically complete or future complete solutions of Einstein’s field equations with smooth asymptotic structure. Commun. Math. Phys..

[21] Gasperín E, Valiente Kroon JA (2017). Perturbations of the asymptotic region of the Schwarzschild-de Sitter spacetime. Ann. Henri Poincaré.

[22] Gibbons GW, Hawking SW (1977). Cosmological event horizons, thermodynamics, and particle creation. Phys. Rev. D.

[23] Hadžić M, Speck J (2015). The global future stability of the FLRW solutions to the dust-Einstein system with a positive cosmological constant. J. Hyperbolic Differ. Equ..

[24] Hintz P (2016). Global analysis of quasilinear wave equations on asymptotically de Sitter spaces. Ann. Inst. Fourier (Grenoble).

[25] Hintz P, Vasy A (2014). Non-trapping estimates near normally hyperbolic trapping. Math. Res. Lett..

[26] Hintz P, Vasy A (2016). Global analysis of quasilinear wave equations on asymptotically kerr-de sitter spaces. Int. Math. Res. Not..

[27] Hintz P, Vasy A (2018). The global non-linear stability of the Kerr-de Sitter family of black holes. Acta Math..

[28] Klainerman, S., Szeftel, J.: Effective results on uniformization and intrinsic gcm spheres in perturbations of kerr (2019)

[29] Kottler F (1918). Über die physikalischen Grundlagen der Einsteinschen Gravitationstheorie. Ann. Phys..

[30] Lemaître, G.: Un univers homogène de masse constante et de rayon croissant, redant compte de la vitesse radiale des nébuleuses extra-galactiques. Ann. Soc. Sci. Bruxelles **47**(A), 49–59 (1927)

[31] Nussbaumer H, Bieri L (2009). Discovering the Expanding Universe.

[32] Rendall AD (2004). Asymptotics of solutions of the Einstein equations with positive cosmological constant. Ann. Henri Poincaré.

[33] Ringström H (2008). Future stability of the Einstein-non-linear scalar field system. Invent. Math..

[34] Rodnianski I, Speck J (2013). The nonlinear future stability of the FLRW family of solutions to the irrotational Euler-Einstein system with a positive cosmological constant. J. Eur. Math. Soc. (JEMS).

[35] Rodnianski, I., Speck, J.: A regime of linear stability for the Einstein-scalar field system with applications to nonlinear big bang formation. Ann. Math. (2) **187**(1), 65–156 (2018)

[36] Rodnianski, I., Speck, J.: Stable big bang formation in near-FLRW solutions to the Einstein-scalar field and Einstein-stiff fluid systems. Selecta Math. (N.S.) **24**(5), 4293–4459 (2018)

[37] Schlue, V.: General relativity, Lecture notes, available at https://blogs.unimelb.edu.au/volker-schlue/home-page/teaching/ (2014)

[38] Schlue V (2015). Global results for linear waves on expanding Kerr and Schwarzschild de Sitter cosmologies. Commun. Math. Phys..

[39] Schlue, V.: Optical functions in de Sitter, J. Math. Phys. **62**(8), Paper No. 082501, 67 (2021)

[40] Speck, J.: The nonlinear future stability of the FLRW family of solutions to the Euler-Einstein system with a positive cosmological constant. Selecta Math. (N.S.) **18**(3), 633–715 (2012)

[41] Speck J (2013). The stabilizing effect of spacetime expansion on relativistic fluids with sharp results for the radiation equation of state. Arch. Ration. Mech. Anal..

[42] Vasy A (2013). Microlocal analysis of asymptotically hyperbolic and Kerr-de Sitter spaces (with an appendix by Semyon Dyatlov). Invent. Math..

[43] Weyl H (1919). Über die statischen kugelsymmetrischen Lösungen von Einsteins kosmologischen Gravitationsgleichungen. Phys. Z..

